# Photocatalytic generation of alkoxysulfonium ions for selective oxidation of benzylic/allylic halides to carbonyls under base-free conditions

**DOI:** 10.1039/d5sc07057k

**Published:** 2025-11-21

**Authors:** Yuanzhen Mao, Xiaofang Zhang, Wei-Yu Shi, Hongyu Guo, Rong Zhou

**Affiliations:** a College of Chemistry and Chemical Engineering, Taiyuan University of Technology Taiyuan 030024 P. R. China zhourong@tyut.edu.cn; b State Key Laboratory of Elemento-Organic Chemistry, Nankai University Tianjin 300071 China

## Abstract

Selective oxidation of organic molecules to carbonyls is a highly desirable but still challenging transformation in both synthetic chemistry and the pharmaceutical industry. Herein, we report a visible-light photocatalytic oxidation of benzylic/allylic halides to carbonyl compounds enabled by a consecutive photoinduced electron transfer (ConPET) and HAT catalysis under base-free conditions. By synergistic combination of an organophotocatalyst 4CzIPN and a thiol HAT reagent in dimethyl sulfoxide (DMSO), a broad range of halides including benzylic, allylic, and even aliphatic halides were smoothly converted into their corresponding aldehydes/ketones in moderate to excellent yields with good functional group tolerance. The robustness of this protocol is further strengthened by selective oxidation of polyhalo compounds and hybrid halo-hydroxyl compounds and controllable oxidation of bioactive molecules. Mechanistic investigation unveils a photocatalytic generation of alkoxylsulfonium ion with DMSO followed by a photoredox assisted decomposition of this species into carbonyl.

## Abstract 2

A base-free photocatalytic oxidation of benzylic/allylic halides to carbonyls is described, which allows selective oxidation of polyhalo compounds and hybrid halo-hydroxyl compounds, as well as controllable oxidation of bioactive molecules.

## Graphical abstract

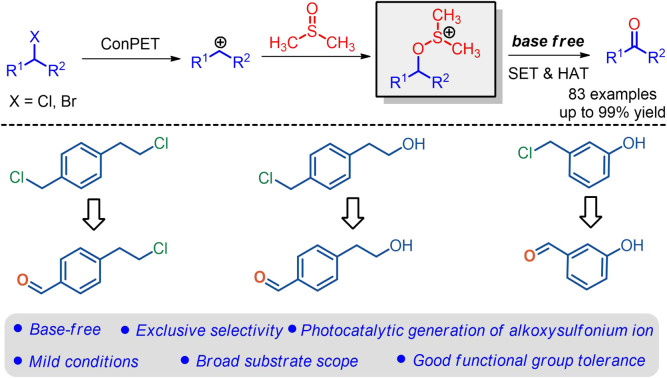

## Introduction

**Scheme 1:**
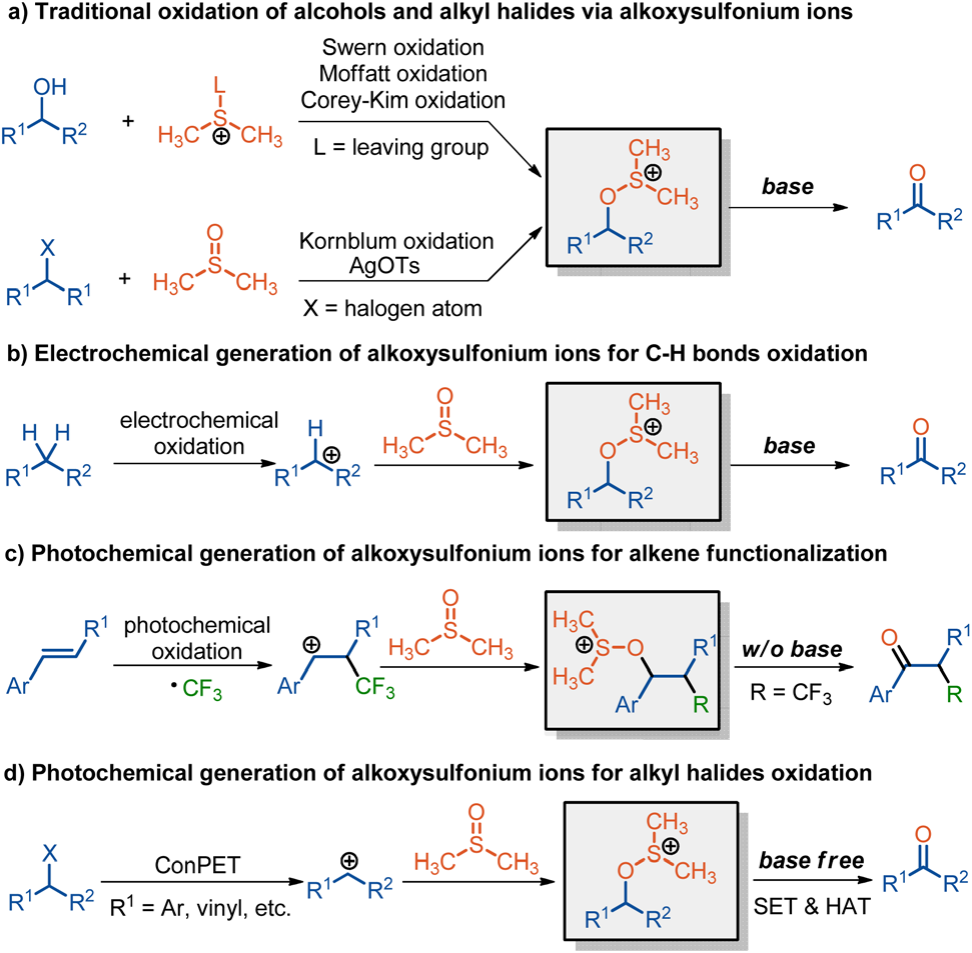
Generation of alkoxysulfonium ions for oxidation.

The controllable oxidation of organic molecules to carbonyl compounds such as aldehydes and ketones is one of the most fundamental transformations in both synthetic chemistry and the pharmaceutical industry due to their versatility in organic synthesis and ubiquity in natural products and pharmaceuticals.^1–3^ In this context, alcohol oxidation serves as a prevailing protocol to access carbonyls,^4^ and a plethora of strategies including traditional named reactions such as Swern oxidation, Corey–Kim oxidation, Moffatt oxidation, and Dess–Martin oxidation have been well established.^5–8^ Alternatively, the direct transformation of alkyl halides into carbonyls has also attracted considerable attention from synthetic community since many organohalides are industrially manufactured through Friedel–Crafts chloromethylation of aromatic compounds.^9–11^ Among both alcohol and alkyl halide oxidations, alkoxysulfonium ions are crucial intermediates in facilitating these transformations as witnessed in Swern oxidation,^5^ Corey–Kim oxidation,^6^ and Kornblum oxidation^12,13^ (Scheme 1a). Upon treatment with bases, the alkoxysulfonium ions can be smoothly converted into carbonyls and thus complete the oxidations. Generally, the alkoxysulfonium ions are formed from either alcohols *via* nucleophilic attack on activated DMSO (*e.g.*, Swern, Corey–Kim oxidations) or benzylic halides, first converted to tosylates using silver tosylate and then reacted with DMSO upon heating (Kornblum oxidation). However, the limitations including use of stoichiometric amounts of activated reagents such as trifluoroacetic anhydride (TFAA), oxalyl chloride, *N*-chlorosuccinimide (NCS), dicyclohexylcarbodiimide (DCC), silver tosylate, or harsh reaction conditions undermine their practical applications.^5–7,12^ Furthermore, the requirement of base additives to decompose the alkoxysulfonium ions into final carbonyls also brings about the alkene byproduct by elimination and functional group tolerance issue. In 2011, Yoshida and co-workers developed a mild electrochemical generation of alkoxysulfonium ions through *in situ* trapping of the electrochemically generated carbocation by DMSO, leading to the oxidation of both benzylic C–H bonds and alkenes to carbonyls (Scheme 1b).^14,15^ However, bases such as triethylamine are still indispensable to convert the resulting alkoxysulfonium ions into carbonyls.

Photoredox catalysis has witnessed dramatic developments over the past decade, enabling the discovery and invention of numerous unique and valuable transformations *via* open-shell radical species.^16–21^ In this regard, the photocatalytic generation of alkoxysulfonium ions for oxidative transformation has also been reported sporadically. In 2014, Akita and co-workers pioneered a photocatalytic keto-trifluoromethylation of aromatic alkenes, where an alkoxysulfonium ion, obtained by DMSO trapping of the photocatalytically generated β–CF_3_–substituted carbocation, serves as a key intermediate to produce the desired α-trifluoromethylated ketones (Scheme 1c).^22^ Soon after that, the photocatalytic keto-difluoroalkylation and carboxylation of aromatic alkenes were also reported by employing a similar strategy *via* an alkoxysulfonium ion intermediate.^23–27^ Despite their efficiency in oxidative functionalization of alkenes, the photocatalytic generation of alkoxysulfonium ion for direct transformation of alkyl halides into carbonyls has never been realized. Although photo-induced aerobic oxidation of benzylic halides has been reported occasionally, these approaches suffer from low selectivity, the requirement of base additives, limited substrate scope, and confinement primarily to benzylic bromides.^28–31^

In 2021, we reported a ConPET process to reduce challenging electron-rich aryl chlorides to aryl radicals for aromatic C–B, C–P, and C–C bond formation.^32^ Building on this foundation, we envisioned that combining a ConPET with a single-electron oxidation would offer a carbocation from alkyl halides. This intermediate is then trapped by DMSO, forming an alkoxysulfonium ion and ultimately enabling the carbonylation of alkyl halides (Scheme 1d). Herein, we report the realization of this hypothesis in detail.

## Results and discussion

### Optimization of reaction conditions

We initiated the study with 4-methoxybenzyl chloride (1) in the presence of an organic photocatalyst, 4CzIPN (PC 1), and a catalytic amount of ethyl 2-mercaptopropionate (HAT cat 1) in MeCN with 20 eq. H_2_O under the illumination of a blue LED. Delightfully, the desired oxidative dehalogenation product 4-methoxybenzaldehyde (2) was afforded in 59% isolated yield, along with hydrogen evolution as detected by GC analysis (Table 1, entry 1). This indicates the possible generation of a benzyl alcohol intermediate, which subsequently undergoes photocatalytic acceptorless dehydrogenation to deliver the desired aldehyde as we previously reported.^33^ A couple of reaction parameters were investigated in order to further improve the reaction efficiency. Several organic photocatalysts were investigated, with 4CzIPN outperforming alternatives including the analogous cyanoarene-based catalyst PC 2, triarylpyranium salts PC 3 and 4, and 9-mesityl-10-methylacridinium perchlorate (PC 5) (entries 2–5). Solvent screening indicated that dimethyl sulfoxide (DMSO) was the best reaction medium, whereas other solvents such as THF, 1,4-dioxane, and DMF led to inferior yields (entries 6–9). Interestingly, reducing the loading of H_2_O to 5 eq. or even in the absence of H_2_O, the reaction still occurred smoothly albeit in reduced yields, indicating H_2_O as a co-solvent with DMSO (entries 10 and 11). It is worth noting that no hydrogen gas was detected with DMSO as the solvent, highlighting an alternative reaction pathway. Other sulfur-centered HAT reagents such as *tert*-dodecylmercaptan (HAT cat 2), 1-butanethiol (HAT cat 3), triisopropylsilylthiol (HAT cat 4), 2,4,6-triisopropylbenzenethiol (HAT cat 5) all resulted in decline in yields (entries 12–15). Control experiments indicated that both the photocatalyst and light are essential for a successful transformation (entry 16). The absence of the external HAT catalyst also brought about 44% yield (entry 17), most likely due to the dissociated Cl^−^ from methylbenzyl chloride serving as a HAT reagent; this is supported by the observation that the external addition of KCl also afforded the product in 74% yield (entry 18).

**Table 1:** Optimization of the model reaction conditions^a^

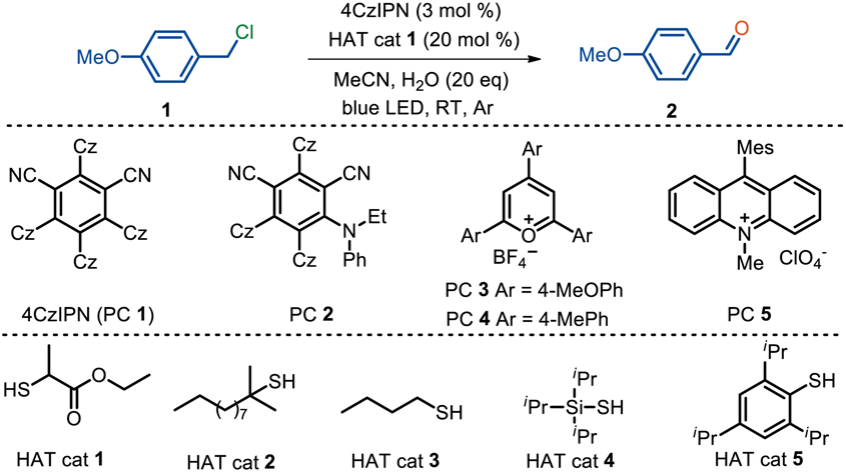		
Entry	Deviation from the model conditions	Yield of 2^b^ (%)
1	None	59
2	PC 2 instead of PC 1	7
3	PC 3 instead of PC 1	56
4	PC 4 instead of PC 1	23
5	PC 5 instead of PC 1	26
6	THF instead of MeCN	26
7	1,4-Dioxane instead of MeCN	28
8	DMF instead of MeCN	33
9	DMSO instead of MeCN	88
10^c^	DMSO instead of MeCN	77
11^d^	DMSO instead of MeCN	70
12^e^	HAT cat 2 instead of 1	38
13^e^	HAT cat 3 instead of 1	24
14^e^	HAT cat 4 instead of 1	14
15^e^	HAT cat 5 instead of 1	10
16^e^	Without PC or light	N.D.
17^e^	Without HAT cat	44
18^e^^,^^f^	KCl instead of HAT cat	74

a Typical procedure: 1 (0.20 mmol), H_2_O (20 eq), 4CzIPN (3.0 mol%), HAT cat. 1 (20 mol%), 24 W blue LED, Ar, RT, 48 h.

b Isolated yield.

c H_2_O (5 eq.) was added.

d Without H_2_O.

e DMSO was used as the solvent.

f KCl (1 eq.) was added.

### Scope of the dehalocarbonylation reaction

**Scheme 2:**
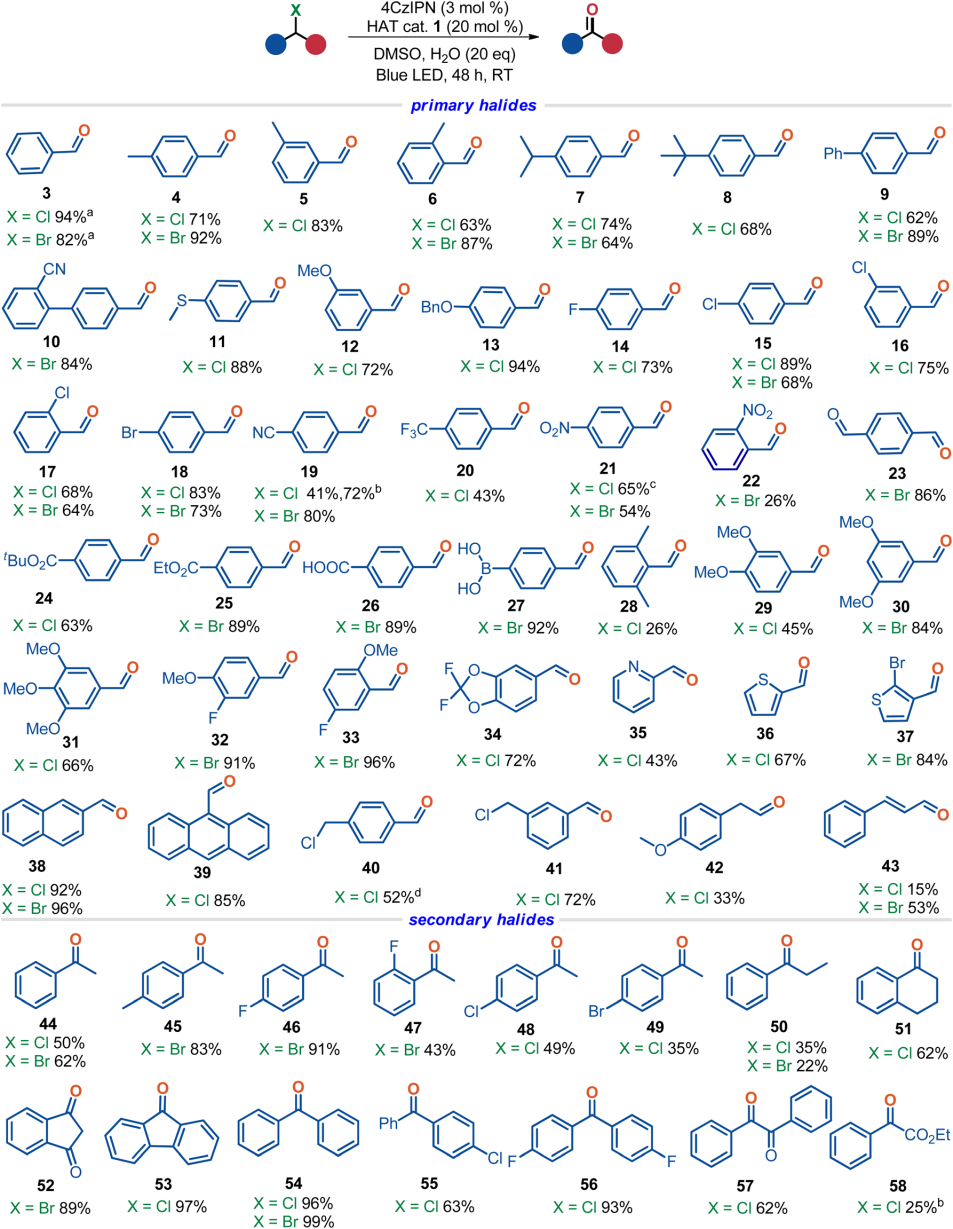
Scope of the dehalocarbonylation reaction. Standard procedure: halide (0.20 mmol), H_2_O (20 eq), 4CzIPN (0.006 mmol, 3 mol%), HAT cat 1 (0.04 mmol, 20 mol%), DMSO (2 mL), 24 W blue LED, Ar, RT, 48 h, isolated yields. ^a^Yields were determined by analysis of the crude ^1^H NMR spectra using 1,3,5-trimethoxybenzene as an internal standard. ^b^KBr (1 eq.) was used instead of HAT cat 1. ^c^The reaction time is 24 h. ^d^Terephthalaldehyde was isolated in 15% yield accompanied by 40.

With the optimal conditions in hand, we sought to explore the generality of the oxidative dehalogenation reaction (Scheme 2). A plethora of primary benzyl halides including both benzyl chlorides and bromides bearing either electron-neutral, electron-rich, or electron-poor aromatic rings participated in the reaction smoothly to afford their corresponding aldehydes in moderate to excellent yields (3–27). Functionalities such as cyano (10 and 19), thioether (11), methoxy (12), benzyloxy (13), halogen (14–18), trifluoromethyl (20), nitro (21 and 22), acyl (23), ester (24 and 25), carboxylic acid (26), and even boronic acid (27) groups were well tolerated. Notably, the tolerance of acidic functional groups renders this protocol extremely attractive because these groups are fragile under traditional basic Kornblum oxidation conditions. Di- and tri-substituted substrates were also capable of delivering their products irrespective of the position of the substituents on the phenyl rings (28–34). Heteroarenes (35–37) and sterically hindered arenes (38 and 39) on the benzyl halides were readily accommodated as well. Interestingly, bi-chlorides such as 1,4-bis(chloromethyl)benzene (40) and 1,3-bis(chloromethyl)benzene (41) predominantly afforded the mono-dechlorocarbonylation products, with one benzyl chloride moiety intact for further transformation.^34^ It is worth noting that the challenging aliphatic halides and allylic halides were also viable substrates, leading to the formation of their corresponding aldehydes in moderate yields (42 and 43). However, alkyl iodides such as benzyl iodide and 4-methoxyphenethyl iodide failed to afford the desired carbonyls under the standard photocatalytic conditions.

We then turned our attention to the secondary halides. As depicted in Scheme 2, aryl–alkyl hybrid secondary benzyl halides bearing either electron-rich or electron-poor aryl rings were compatible, readily affording their acetophenone derivatives in moderate to excellent yields (44–49). A longer aliphatic chain was detrimental to the reaction, resulting in an inferior yield (50), while cyclic benzyl halides such as 1-chlorotetralin (51) and 3-bromo-1-indanone (52) underwent the oxidation smoothly to deliver their ketone products in high yields. Diaryl-substituted benzyl halides were examined as well, which uniformly furnished their corresponding products in good to excellent yields (53–56). Aryl α-halo ketones such as 2-chloro-1,2-diphenylethanone (57) and α-halo esters like ethyl α-chlorophenylacetate (58) were amenable to this dehalocarbonylation reaction as well. Remarkably, as witnessed in the above results, this protocol is typically effective towards the challenging alkyl chlorides, which is different from the traditional Kornblum oxidation and other reported synthetic methodologies where alkyl chlorides were challenging substrates.^12,13,29–31^

### Synthetic application

**Scheme 3:**
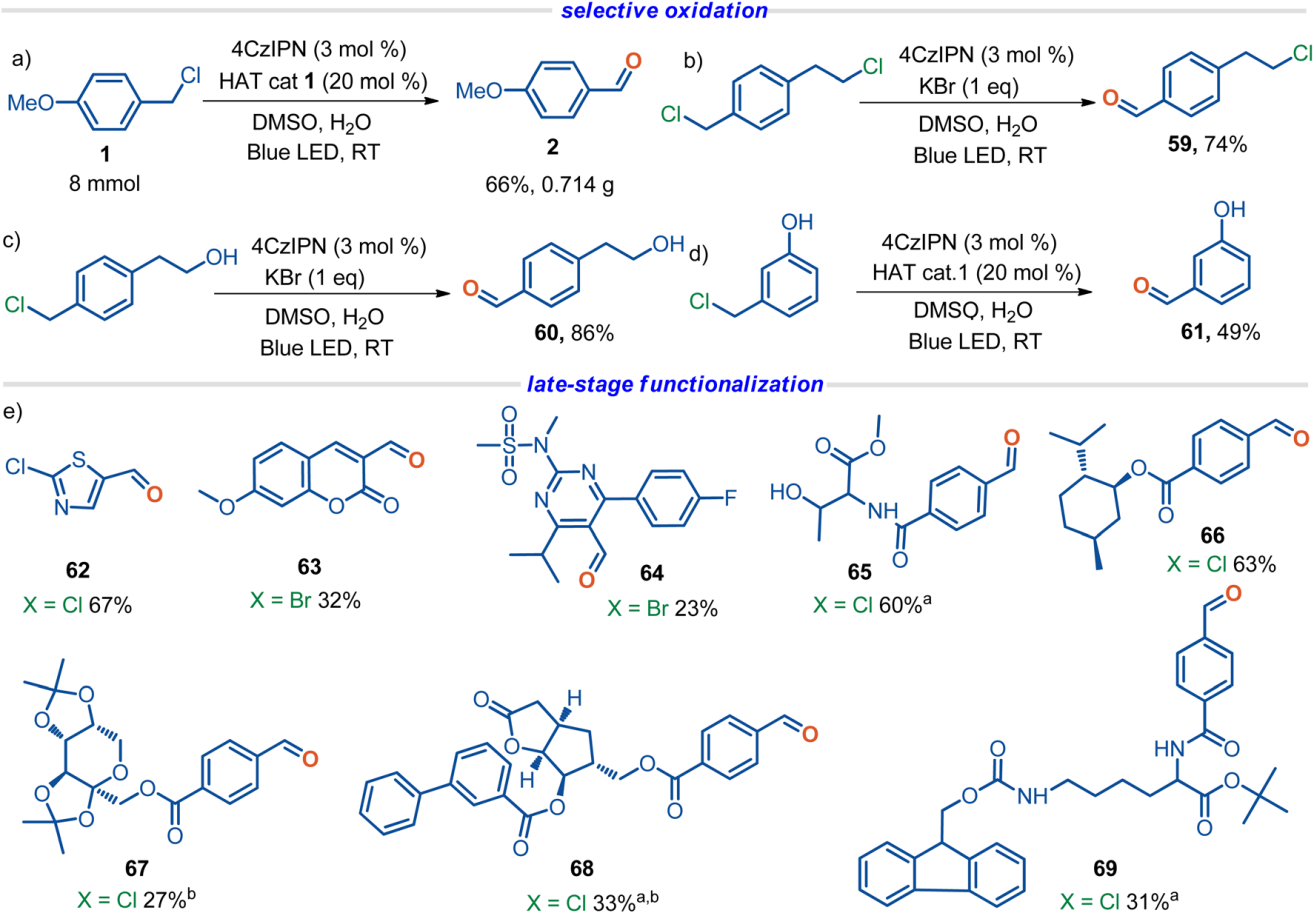
Synthetic application. Standard procedure: halide (0.20 mmol), H_2_O (20 eq.), 4CzIPN (3.0 mol%), HAT cat 1 (0.04 mmol, 20 mol%), DMSO (2 mL), 24 W blue LED, Ar, RT, 48 h, isolated yields. ^a^NaBr (2 eq.) instead of HAT cat 1 was used, and the reaction time is 3 d. ^b^Without H_2_O.

The synthetic utility of the protocol was further demonstrated by its feasibility to scale-up to large scale with high efficiency (Scheme 3a). Furthermore, selective oxidation was realized by this protocol as well. When the benzyl halides tethered with either an aliphatic halide (59), an aliphatic alcohol (60), or a phenol group (61) were subjected to the standard conditions, only the benzylic halide moiety was oxidized, with other fragile functional groups intact (Scheme 3b–d). The robustness of this methodology was further strengthened by late-stage functionalization of drugs and bioactive molecules (Scheme 3e), including the neonicotinoid insecticide—thiamethoxam precursor (62), coumarin derivative (63), rosuvastatin precursor (64), l-threonine and l-lysine derivatives (65 and 69), l-menthol derivative (66), d-fructopyranose derivative (67), and biphenyl Corey lactone (68).

### Mechanistic investigation

**Scheme 4:**
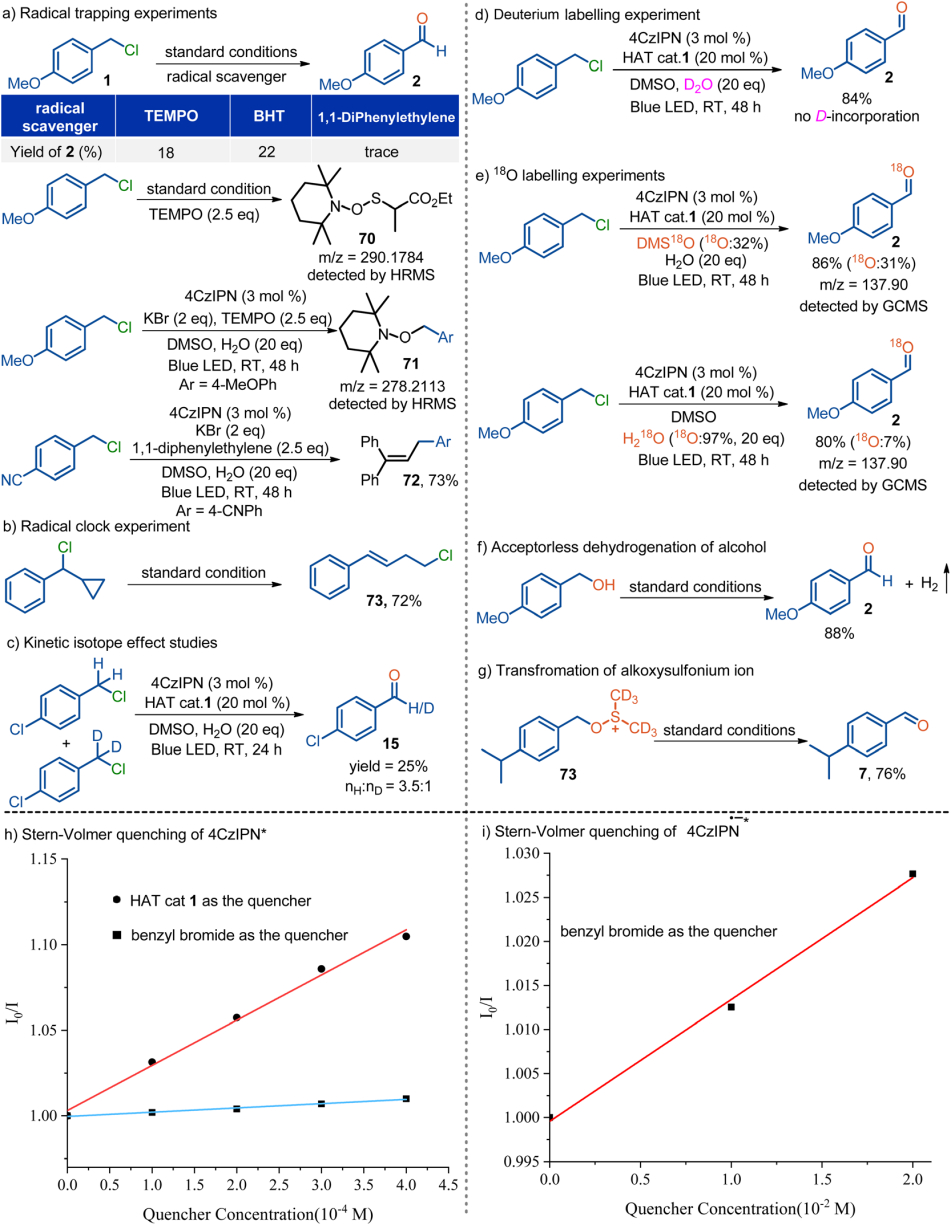
Mechanistic investigation.

A series of control experiments were conducted to elucidate the mechanism of this dehalocarbonylation process (Scheme 4 and also see the SI). (1) Radical inhibition experiments indicated that radical scavengers such as TEMPO and BHT significantly inhibited the reaction, whereas 1,1-diphenylethylene blocked the reaction completely. Furthermore, the intermediate 70 was detected by high-resolution mass spectrometry (HRMS) when TEMPO was used as the radical scavenger under the standard photocatalytic conditions. In addition, the intermediate 71 was also detected by HRMS when using KBr as the HAT reagent under the otherwise identical conditions. Notably, the trapped product 72 was isolated in 73% yield in the presence of KBr and 1,1-diphenylethylene (Scheme 4a). These results strongly support the involvement of radical species and the presence of both the thiyl radical and the benzylic radical in the reaction. (2) Radical clock experiment using (chloro(cyclopropyl)methyl)benzene under the standard photocatalytic conditions furnished the ring-opening product 73 in 72% yield, which further supports the involvement of a benzylic radical (Scheme 4b).^35^ (3) When equal moles of 1-chloro-4-(chloromethyl)benzene and 1-chloro-4-(chloromethyl-*d*_2_)benzene were subjected to the standard photocatalytic conditions, the H/D ratios in product 15 were calculated to be *n*_H_/*n*_D_ = 3.5 : 1, implying that the C–H abstraction step might be the rate determining step (Scheme 4c). Furthermore, we also conducted the reaction using D_2_O instead of H_2_O under the otherwise standard conditions, and no deuterium incorporation was detected in the isolated product 15, ruling out the possible formation of a benzylic carbanion during the reaction (Scheme 4d). (4) In order to investigate the origin of the oxygen, we synthesized ^18^O labelled DMS^18^O (abundance of ^18^O in DMS^18^O: 32%).^36^ When DMS^18^O was used as the solvent, the desired ^18^O labelled product 2 was obtained in 86% yield with the expected ^18^O content (abundance of ^18^O: 31%). Meanwhile, H_2_^18^O instead of H_2_O was added to the reaction leading to the formation of product 2 in 80% yield with inferior ^18^O content (abundance of ^18^O: 7%). These results, together with the result in Table 1 entry 11, indicate that DMSO acts as both the solvent and oxygen source (Scheme 4e). (5) In accordance with our previous photocatalytic acceptorless dehydrogenation of alcohols,^33^ treatment of alcohols such as 4-methoxybenzyl alcohol under the standard conditions uneventfully furnished the corresponding aldehyde 2 in good yield, along with hydrogen evolution simultaneously (Scheme 4f). However, as illustrated in Table 1, entry 9, when 4-methoxybenzyl chloride (1) was subjected to the standard conditions, the aldehyde product 2 was smoothly afforded with no detectable formation of hydrogen gas. These results indicate that the alcohol is presumably not the active species of the dehalogenative carbonylation reaction. (6) The alkoxysulfonium salt 73 was synthesized and subjected to the standard photocatalytic conditions, affording the desired aldehyde 7 in 76% yield.^37^ This result suggests that the alkoxysulfonium salt is most likely an intermediate of the reaction (Scheme 4g). (7) Stern–Volmer quenching experiments demonstrate that thiols such as HAT cat 1 can quench the excited PC 1*, whereas benzyl halides such as benzyl bromide cannot quench it (Scheme 4h). Furthermore, we prepared the reduced-state photocatalyst PC 1˙^−^ according to our previous study and subjected it to the quenching experiment.^32^ It clearly revealed that the excited PC 1˙^−^* can be quenched by benzyl halides such as benzyl bromide (Scheme 4i). These results strongly support a ConPET process in the reduction of the benzyl halide. (8) Light on–off experiments revealed the interruption of the reaction progress in the dark and recuperation of reactivity on further irradiation. Together with the calculated quantum yield with a value of *Φ* = 0.53, it indicates a non-chain process (see the SI).

**Scheme 5:**
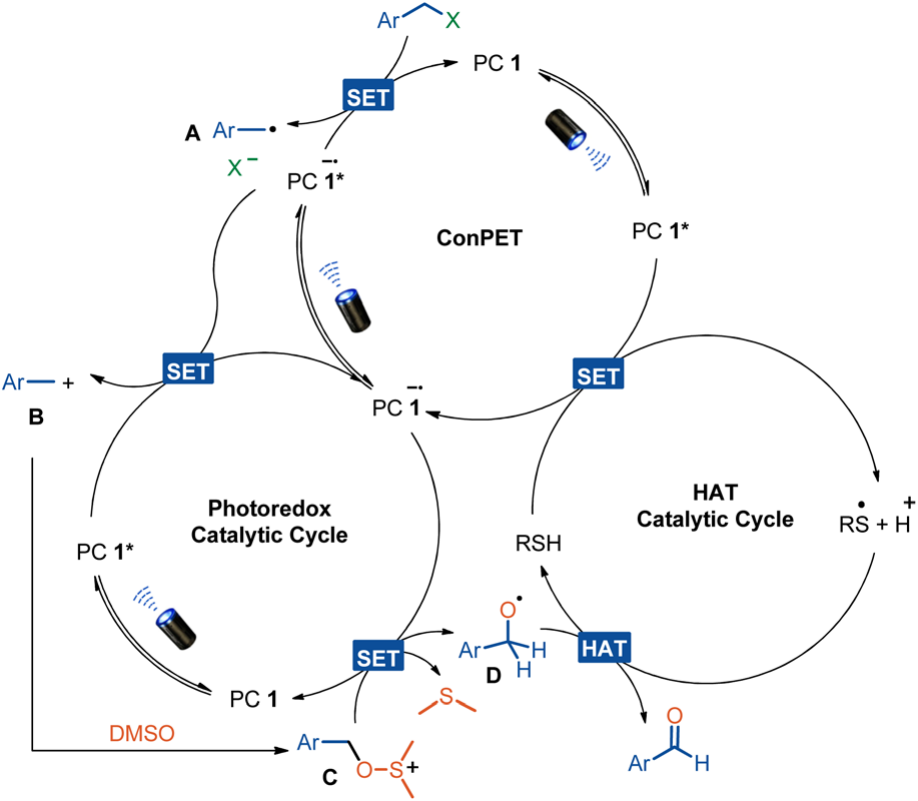
Proposed mechanism.

On the basis of the experimental results, a reasonable account of the formation of the carbonyl compound is depicted in Scheme 5. Photoexcitation of the catalyst 4CzIPN generates its excited state PC 1* [*E*_1/2_(*P/P^−^) = +1.35 V *vs.* SCE in MeCN], which is reductively quenched by thiols such as HAT cat 1,^38^ producing the thiyl radical after deprotonation and the reduced-state photocatalyst PC 1˙^−^. The reduced photocatalyst PC 1˙^−^ is then excited by photo irradiation again to give the excited state PC 1˙^−^*, which is reductive enough to reduce alkyl halides such as benzyl chloride (*E*^red^_1/2_ = −2.25 V *vs.* SCE), generating the benzylic radical A with recovery of the photocatalyst.^39,40^ Single electron oxidation of radical A by the excited PC 1* affords its cation intermediate B. DMSO trapping of cation intermediate B results in the formation of alkoxysulfonium ion C (*E*_1/2_ = −0.69 V *vs.* SCE in MeCN),^22^ which undergoes single electron reduction by the reduced photocatalyst PC 1˙^−^ [*E*_1/2_(P/P^−^) = −1.21 V *vs.* SCE in MeCN] generating the radical intermediate D along with the release of dimethyl sulfide. Finally, a HAT between the thiyl radical and intermediate D furnishes the carbonyl product and turns over the catalytic cycle.^41^

## Conclusions

In summary, a visible light photocatalytic oxidation of benzylic/allylic halides to carbonyl compounds enabled by a ConPET and HAT catalysis has been developed. A broad range of halides including benzylic, allylic, and aliphatic chlorides as well as bromides were facilely converted into aldehydes/ketones in moderate to excellent yields. Furthermore, this protocol is also applicable to site-selective oxidation of polyhalo and hybrid halo-hydroxy compounds and controllable oxidation of bioactive compounds. Mechanistic investigation supports photocatalytic generation of an alkoxysulfonium ion followed by a photoredox assisted decomposition of it to carbonyl. The merits of this methodology such as base-free, mild conditions, broad substrate scope, good functional group tolerance, and exclusive selectivity render it a highly attractive and practical approach for oxidation of alkyl halides.

## Author contributions

R. Z. conceived and directed the project. Y. M. and X. Z. performed the experiments. W.-Y. S., H.G. and R. Z. discussed the results. Y. M. and R. Z. wrote the manuscript.

## Conflicts of interest

There are no conflicts to declare.

## Supplementary Material

SC-017-D5SC07057K-s001

## Data Availability

The data supporting this article have been included as part of the supporting information (SI). Supplementary information is available. See DOI: https://doi.org/10.1039/d5sc07057k.
