# Supplementary material for: Photocatalytic generation of alkoxysulfonium ions for selective oxidation of benzylic/allylic halides to carbonyls under base-free conditions
Source: Chem Sci. 2025 Nov 21;17(3):1771–7. doi: 10.1039/d5sc07057k (PMC12666653; doi:10.1039/d5sc07057k)

Supplementary Information (SI) for Chemical Science.
This journal is © The Royal Society of Chemistry 2025

Supporting Information

Photocatalytic Generation of Alkoxysulfonium Ions for Selective Oxidation of Benzylic/Allylic Halides to Carbonyls under Base-Free Conditions

Yuanzhen Mao,‡[a] Xiaofang Zhang,‡[a] Wei-Yu Shi,[a] Hongyu Guo,[a] and Rong Zhou*[a,b]

[a] College of Chemistry and Chemical Engineering, Taiyuan University of Technology, Taiyuan, 030024, China.

[b] State Key Laboratory of Elemento-Organic Chemistry, Nankai University, Tianjin, 300071, China.

‡ These authors contributed equally to this work.

Table of Contents

1. General information S2
2. Optimization of reaction conditions S3
3. Preparation of raw materials S6
   3.1 Preparation of chlorides S6
   3.2 Preparation of 4-Chlorobenzyl chloride-d_2 .... S6
   3.3 Modification of bioactive molecules by amidation S7
   3.4 Modification of bioactive molecules by esterification S7
   3.5 Preparation of DMS^{18}O S8
   3.6 Preparation of alkoxylsulfonium salt S8
4. General procedure for the oxidation of halides S9
5. Scale-Up synthesis of compound 2 .... S10
6. Mechanistic investigations S10
   6.1 Radical trapping experiments S10
   6.2 Radical clock experiment S11
   6.3 Stern-Volmer fluorescence quenching study S11
   6.4 Quantum yield experiment S12
   6.5 Light on-off experiments S14
   6.6 Kinetic isotope effect studies S14
   6.7 ^{18}O labelled experiments S15
   6.8 Transformation of alkoxylsulfonium salt intermediate S17
   6.9 Deuterium labeling experiment S17
   6.10 Detection of benzyl sulfide S18
   6.11 Plausible mechanism for the dehalocarbonylation reaction in other solvents instead of DMSO 19
7. Analytical data of the products 21
8. References 43
9. ^{1}H and ^{13}C NMR Spectra of the products 48

1. General information

Unless otherwise noted, all reagents were purchased from commercial suppliers and used without further purification. Column chromatography purification was performed using 200-300 mesh silica gel. ^{1}H NMR and ^{13}C NMR spectra were recorded on a Bruker AV-Ⅲ400 (400 MHZ) spectrometer. ^{1}H and ^{13}C NMR spectra were recorded in $CDCl_3$ (δ 7.26, 77.0 ppm) or DMSO-d_6 (δ 7.16, 128.0 ppm) with tetramethylsilane (TMS) as the internal standard. Multiplicity was indicated as follows: s (singlet), d (doublet), t (triplet), q (quartet), m (multiplet), dd (doublet of doublet). All high resolution mass spectra (HRMS) were obtained by ESI mode with the mass analyzer of TOF used. The blue LED strips (2.0 meter, 24 W, maximum emission at around 440 nm) were purchased from Shenzhen Lingke Lighting Co., Ltd (China). The distance between the Schlenk tube and the light strips is about 3-4 cm. All the photocatalysts are known compounds, which are either commercially available or prepared by reported procedures. The PC **1**, [1] PC **2**,[2] PC **3**, PC **4** [3] and PC **5** [4] were prepared according to literature procedures.

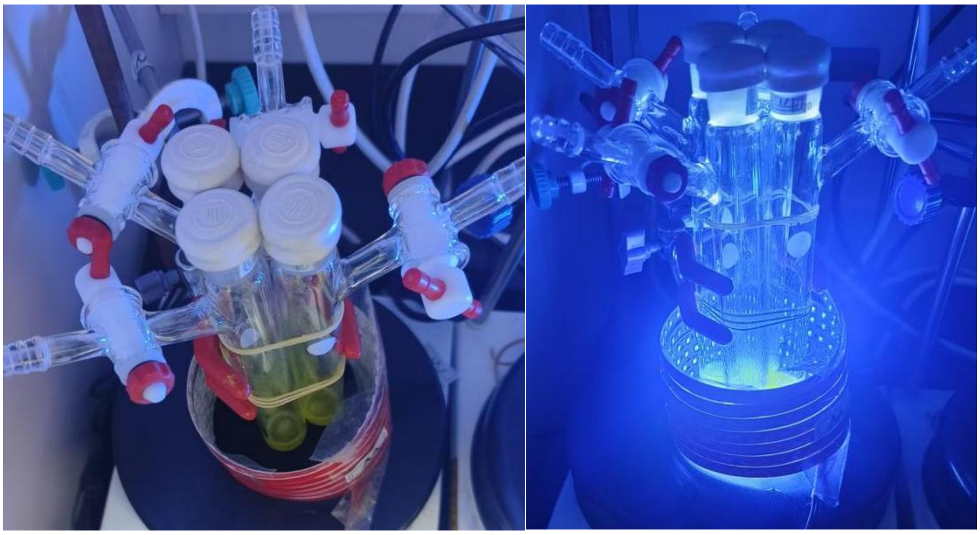

Figure S1. The reaction setup

2. Optimization of reaction conditions

Table S1. Optimization of photocatalysts

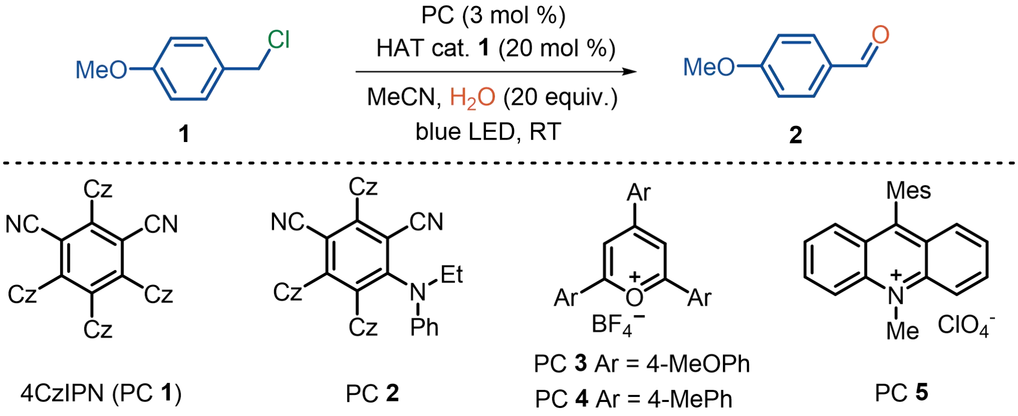

Entry	PC	Yield of **2** (%)[a]
**1**	**PC 1**	**59**
2	PC **2**	7
3	PC **3**	none
4	PC **4**	56
5	PC **5**	23

Reaction conditions: **1** (0.2 mmol), PC (3.0 mol %), HAT cat. **1** (20 mol %), H_2O (20 equiv.), MeCN (2 mL), 24 W blue LED, RT, 48 h. [a]Isolated yield.

Table S2. Optimization of solvents

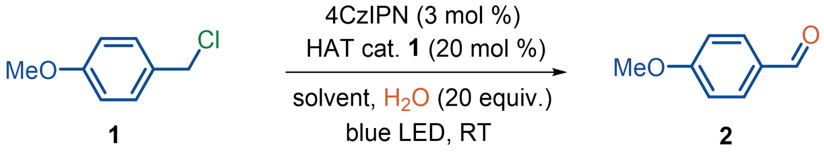

Entry	Solvent	Yield of **2** (%)[a]
1	MeCN	59
2	MeOH	22
3	THF	26
4	1,4-Dioxane	28
**5**	**DMSO**	**88**
6	DMF	33

Reaction conditions: **1** (0.2 mmol), PC **1** (3.0 mol %), HAT cat.**1** (20 mol %), H_2O (20 equiv.), solvent (2 mL), 24 W blue LED, RT, 48 h. [a]Isolated yield.

Table S3. Optimization of HAT catalysts

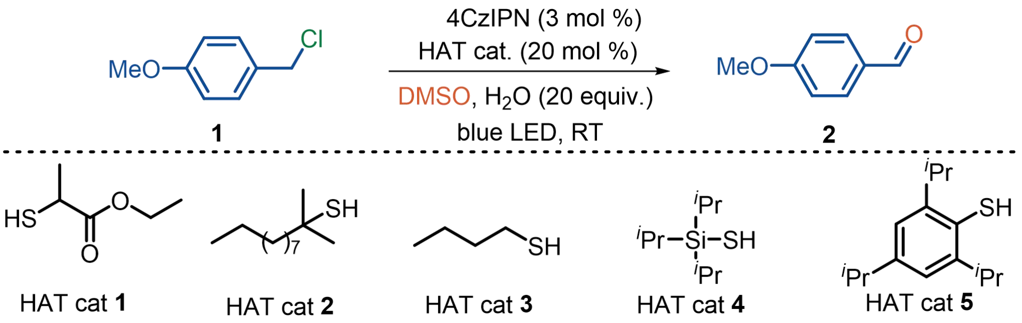

Entry	HAT cat.	Yield of **2** (%)[c]
**1**	**HAT cat. 1**	**88**
2	HAT cat. **2**	38
3	HAT cat. **3**	24
4	HAT cat. **4**	14
5	HAT cat. **5**	10
6[a]	KCl	74
7[b]	KBr	88

Reaction conditions: **1** (0.20 mmol), 4CzIPN (3.0 mol %), HAT cat. (20 mol %), H_2O (20 equiv.), DMSO (2 mL), 24 W blue LED, RT, 48 h. [a]KCl (1 equiv.). [b]KBr (1 equiv.). [c]Isolated yield.

Table S4. Optimization of the amount of H_2O

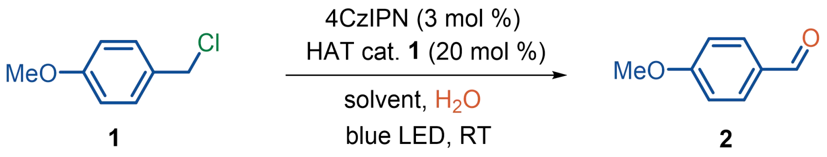

Entry	Amount of H_2O (equiv.)	Yield of **2** (%)[a]
1	0	70
2	5	77
3	15	83
**4**	**20**	**88**
5	30	88

Reaction conditions: **1** (0.20 mmol), 4CzIPN (3.0 mol %), HAT cat.**1** (20 mol %), DMSO (2 mL), 24 W blue LED, RT, 48 h. [a]Isolated yield.

Table S5. Optimization of the amount of HAT reagent

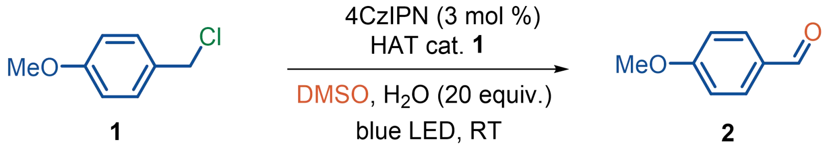

Entry	Amount of HAT cat. (mol %)	Yield of **2** (%)[a]
1	0	44
2	10	73
3	15	77
**4**	**20**	**88**
5	25	88

Reaction conditions: **1** (0.20 mmol), 4CzIPN (3.0 mol %), HAT cat.**1**, H_2O (20 equiv.), DMSO (2 mL), 24 W blue LED, RT, 48 h. [a]Isolated yield.

Table S6. Control experiments

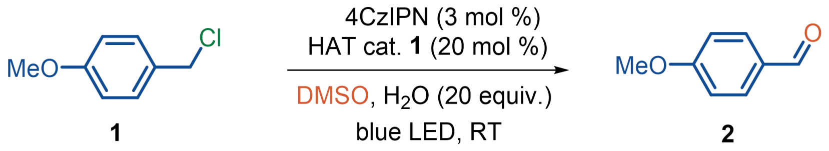

Entry	Deviation from the model conditions	Yield of **2** (%)[a]
1	None	88
2	Without HAT cat.	44
3	Without PC or Light	N.D.
4	Without H_2O	70
5[a]	0.2 M	74
6	Under air	4

Reaction conditions: **1** (0.20 mmol), 4CzIPN (3.0 mol %), HAT cat. **1** (20 mol %), H_2O (20 equiv.), DMSO (2 mL), 24 W blue LED, RT, 48 h. [a]Isolated yield.

3. Preparation of raw materials

3.1 Preparation of chlorides

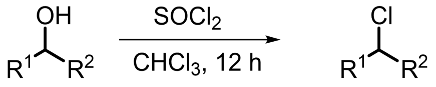

In an oven-dried 25 mL 3-neck round bottom flask, Benzyl alcohol (9.25 mmol, 1.10 equiv.) was dissolved in 7.00 mL $CHCl_3$ under a positive N_2 pressure, with an outlet leading into sat. aq. $NaHCO_3$. A solution of 1.00 g $SOCl_2$ (8.41 mmol, 1.00 equiv.) in 3.00 mL $CHCl_3$ was added dropwise via syringe, and the resulting mixture was stirred at room temperature for 12 h. To quench the reaction, 15.0 mL sat. aq. $NaHCO_3$ were added, and the mixture was extracted with $CHCl_3$ (3 × 20.0 mL). The combined organic layers were dried over $MgSO_4$ and the solvent was removed in vacuo. The product was purified by column chromatography using neutralized silica gel (netutralized w/ ca. 3% Et_3N in hexane) and eluting with hexane.[5]

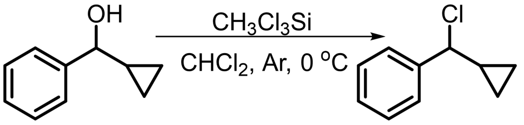

(Preparation of (chlorocyclopropylmethyl)benzene: Methyltrichlorosilane (2.6 mL, 30 mmol) was added dropwise to a dichloromethane solution of cyclopropylbenzyl alcohol (6 mmol), and the reaction was carried out under argon at 0 °C for 1 h. At the end of the reaction, the reaction was burst with H_2O and the reaction was extracted with dichloromethane (3 × 20 mL). The organic layer was collected, dried with anhydrous $MgSO_4$, concentrated in vacuum and purified by column chromatography to obtain (chlorocyclopropylmethyl)benzene.[6]

3.2 Preparation of 4-Chlorobenzyl chloride-d_2

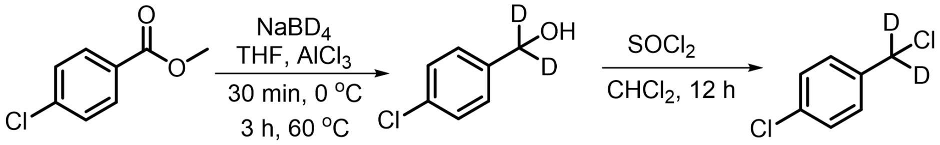

A solution of methyl 4-chlorobenzoate (0.85 g, 5 mmol) in THF (10 mL) was cooled to 0 °C. $AlCl_3$ (0.67 g, 5 mmol) and $NaBD_4$ (0.58 g, 15 mmol) was added portion-wise over 30 min. The reaction was heated to 60 °C with stirring. After 3 h, the mixture was cooled to 0 °C and quenched by careful addition of HCl (1 M) followed by extraction with ethyl acetate (50 mL). The collected organic layers were

dried with anhydrous Na_2SO_4 and concentrated, then the crude material was purified by flash column chromatography on silica gel (PE: EtOAc =5:1) to afford 4-Chlorobenzyl alcohol-*d*$_2$ as a white solid.[7]

In an oven-dried 25 mL 3-neck round bottom flask, 4-Chlorobenzyl alcohol-*d*$_2$ (4.6 mmol) was dissolved in 3.5 mL $CHCl_3$ under a positive N_2 pressure, with an outlet leading into sat. aq. $NaHCO_3$. A solution of 1.00 g $SOCl_2$ (4.2 mmol) in 1.5 mL $CHCl_3$ was added dropwise via syringe, and the resulting mixture was stirred at room temperature for 12 h. To quench the reaction, 7.5 mL sat. aq. $NaHCO_3$ were added, and the mixture was extracted with $CHCl_3$ (3 × 10.0 mL). The combined organic layers were dried over $MgSO_4$ and the solvent was removed in vacuo. The product was purified by column chromatography using neutralized silica gel (netutralized w/ ca. 3% Et_3N in hexane) to afford 4-Chlorobenzyl chloride-*d*$_2$.[5]

3.3 Modification of bioactive molecules by amidation

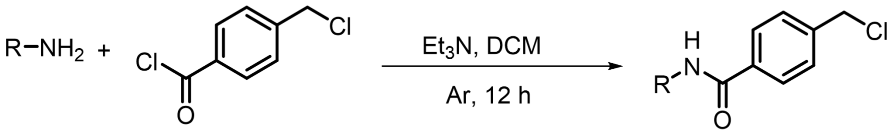

To another oven-dried 100 mL three-necked flask, amino acids (20 mmol, 1.3 equiv.), Et_3N (4.1 mL, 30 mmol, 2 equiv.) and DCM (30 mL) were added. A solution of *p*-chloromethylbenzoyl chloride (2.25 mL, 14 mmol) in DCM (10 mL) was added dropwise to the solution at 0 °C, and the solution was then warmed to room temperature. After stirring overnight, the reaction system was quenched with sat. aq. $NaHCO_3$ (30 mL) and the organic layer was separated. The aqueous layer was extracted with DCM (2 × 15 mL). The combined organic layers were washed with 1 M HCl aq. (30 mL) and brine (30 mL), dried over $MgSO_4$, filtered and evaporated in vacuo. The obtained crude amide was purified by column chromatography on silica gel (hexane/EtOAc = 10/1) to afford the desired amide as a white solid.[8]

3.4 Modification of bioactive molecules by esterification

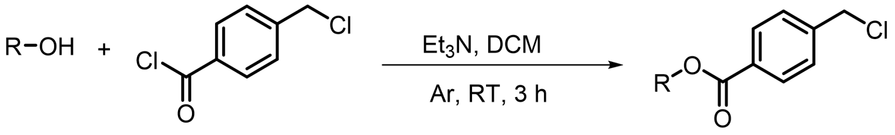

A dry two-necked round-bottom flask was added clcohol (5 mmol) and triethylamine (1.5 equiv.) in DCM (anhydrous) (0.25 M). Then *p*-chloromethylbenzoyl chloride (891 µL, 5.5 mmol) was added dropwise at 0 °C.

After stirring for 30 minutes, the reaction was warm to room temperature and stirred at RT until the completion of the reaction. The reaction was then quenched with H_2O and extracted with ethyl acetate. The combined organic layers were washed with brine, dried over Na_2SO_4 and concentrated. The crude residue was purified by flash column chromatography.[9]

3.5 Preparation of DMS^{18}O

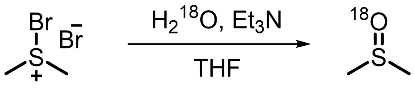

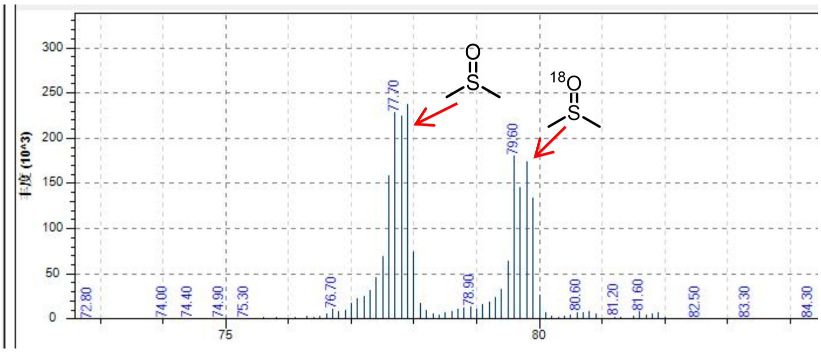

Figure S2. GCMS analysis of ^{18}O incorporated DMS^{18}O

Solid dimethylsulfur dibromide (5.0 g, 22.5 mmol) was added portion wise over 15 min to a vigorously stirred solution of triethylamine (6.3 mL, 45 mmol, freshly distilled from sodium hydroxide) and ^{18}O-labeled H_2O (97%, ^{18}O) (0.20 mL, 11 mmol) in 15 mL of tetrahydrofuran (freshly distilled from sodium metal). The temperature of the reaction was maintained below 50 °C by occasional cooling in ice. The precipitate of triethylamine hydrobromide was removed by centrifugation and washed twice with ether. The combined yellow supernatant and washings were dried on high vacuum pressure pump at room temperature (15 mm) to remove the solvent and the tan residue was distilled in a short path distillation to get ^{18}O-labeled DMSO (^{18}O: 32%), as show in **Figure S2**.[10]

3.6 Preparation of alkoxylsulfonium salt

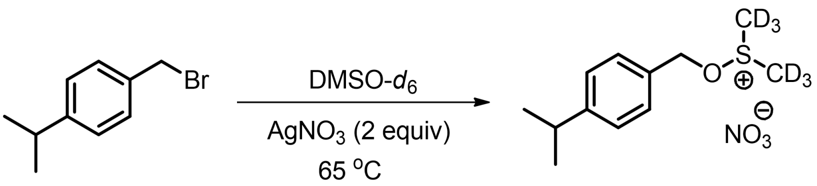

To a 25 mL Schlenk tube equipped with a magnetic stir bar was added $AgNO_3$ (67 mg, 2 equiv.). The Schlenk tube was sealed and degassed via vacuum evacuation

and subsequently backfilled with argon three times. After that DMSO-d_6 (2 mL), 1-(bromomethyl)-4-isopropylbenzene (0.2 mmol) were added sequentially by means of syringe. The reaction was warmed to 65 °C with stirring for 1.5 h. After filtration, the resulting mixture was used for the reaction directly. The corresponding alkoxylsulfonium salt was generated quantitatively according to ^{1}H NMR analysis of the crude mixture (Figure S3).[11] ^{1}H NMR (400 MHz, DMSO) δ 7.39 (d, J = 8.2 Hz, 2H), 7.30 (d, J = 8.1 Hz, 2H), 5.53 (s, 2H), 2.93 – 2.85 (m, 1H), 1.20 (d, J = 6.9 Hz, 6H).

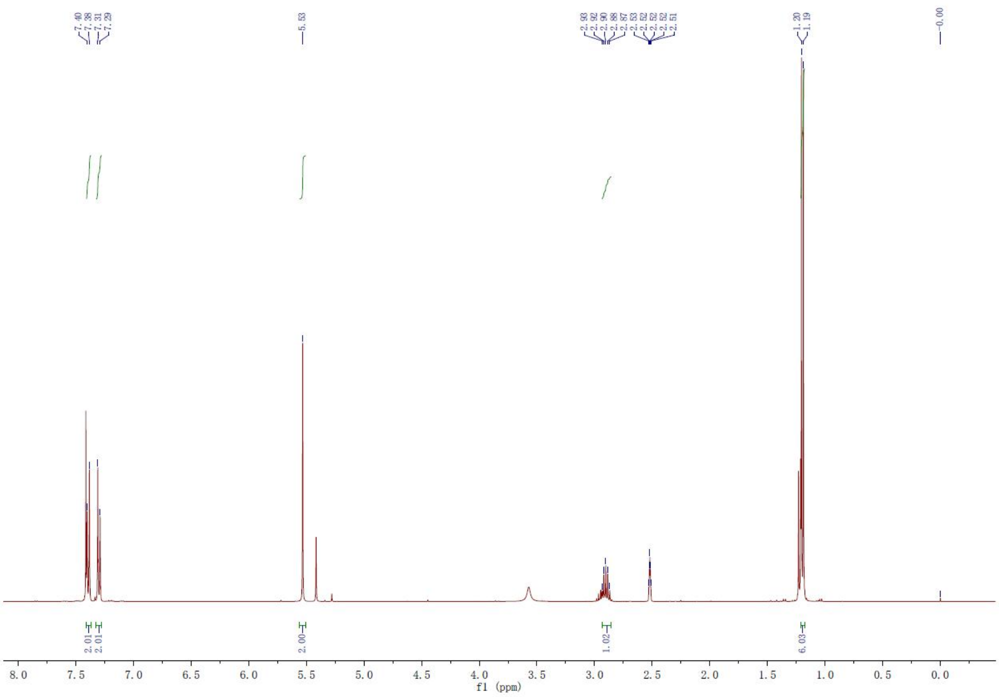

Figure S3. ^{1}H NMR of the crude alkoxylsulfonium salt

4. General procedure for the oxidation of halides

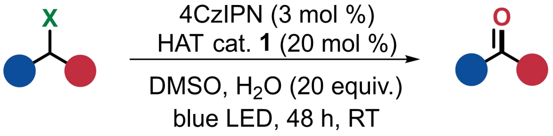

To a 25 mL Schlenk tube equipped with a magnetic stir bar was added the 4CzIPN (PC **1**, 4.7 mg, 0.006 mmol). The Schlenk tube was sealed and degassed via vacuum evacuation and subsequently backfilled with argon three times. After that anhydrous DMSO (2 mL), halide (0.2 mmol), H_2O (72 μL, 20 equiv.), ethyl 2-mercaptopropanoate (HAT cat. **1**, 5.2 μL, 0.02 mmol) were added sequentially by means of syringe. Then the reaction was placed under blue LED irradiated for 48 h.

After that, saturated brine (5 mL) was added and the mixture was extracted with ethyl acetate (3 × 5 mL). The combined organic layer was dried with anhydrous $MgSO_4$. The solvent was removed on a rotary evaporator under reduced pressure. Purification by column chromatography on silica gel gave the desired pure product.

5. Scale-Up synthesis of compound **2**

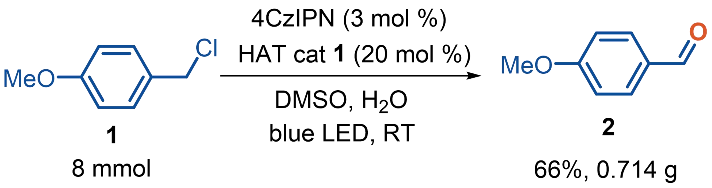

To a 100 mL eggplant shaped flask equipped with a magnetic stir bar was added the 4CzIPN (PC **1**, 188 mg, 0.24 mmol). The eggplant shaped flask was sealed and degassed via vacuum evacuation and subsequently backfilled with argon three times. After that anhydrous DMSO (60 mL), *p*-methoxybenzyl chloride (1160 μL, 8 mmol), H_2O (2.5 mL), ethyl 2-mercaptopropanoate (HAT cat. **1**, 208 μL, 1.6 mmol) were added sequentially by means of syringe. Then the reaction was placed under blue LED irradiated for 3 d. After that, saturated brine (100 mL) was added and the mixture was extracted with ethyl acetate (3 × 50 mL). The combined organic layer was dried with anhydrous $MgSO_4$. The solvent was removed on a rotary evaporator under reduced pressure. Purification by column chromatography on silica gel gave the desired pure product **2** (0.714 g) in 66% yield.

6. Mechanistic investigations

6.1 Radical trapping experiments

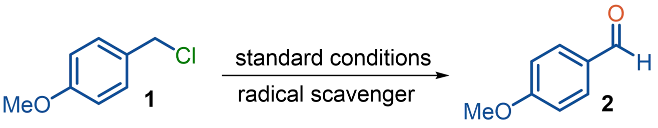

radical scavenger	TEMPO	BHT	1,1-DiPhenylethylene
Yield of **2** (%)	18	22	trace

To a 25 mL Schlenk tube equipped with a magnetic stir bar was added the 4CzIPN (PC **1**, 4.7 mg, 0.006 mmol) and the free radical inhibitor (2.5 equiv., 0.5 mmol). The Schlenk tube was sealed and degaswatersed via vacuum evacuation and subsequently backfilled with argon three times. After that anhydrous DMSO (2 mL), *p*-methoxybenzyl chloride (29 μL, 0.2 mmol), (72 μL, 20 equiv.), ethyl

2-mercaptopropanoate (HAT cat. **1**, 5.2 μL, 0.02 mmol) were added sequentially. Then the reaction was placed under blue LED irradiated for 48 h. After that, saturated brine (5 mL) was added and the mixture was extracted with ethyl acetate (3 × 5 mL). The combined organic layer was dried with anhydrous $MgSO_4$. The solvent was removed on a rotary evaporator under reduced pressure. Purification by column chromatography on silica gel gave the desired pure product **2**.

6.2 Radical clock experiment

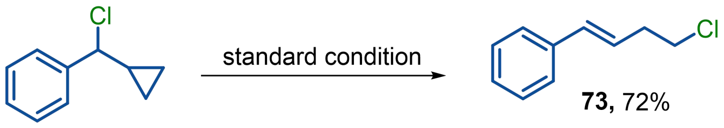

To a 25 mL Schlenk tube equipped with a magnetic stir bar was added the 4CzIPN (PC **1**, 4.7 mg, 0.006 mmol). The Schlenk tube was sealed and degassed via vacuum evacuation and subsequently backfilled with argon three times. After that anhydrous DMSO (2 mL), chlorocyclopropylmethylbenzene (0.2 mmol), H_2O (72 μL, 20 equiv.), ethyl 2-mercaptopropanoate (HAT cat. **1**, 5.2 μL, 0.02 mmol) were added sequentially by means of syringe. Then the reaction was placed under blue LED irradiated for 48 h. After that, saturated brine (5 mL) was added and the mixture was extracted with ethyl acetate (3 × 5 mL). The combined organic layer was dried with anhydrous $MgSO_4$. The solvent was removed on a rotary evaporator under reduced pressure. Purification by column chromatography on silica gel gave the desired pure product **73**.

6.3 Stern-Volmer fluorescence quenching study

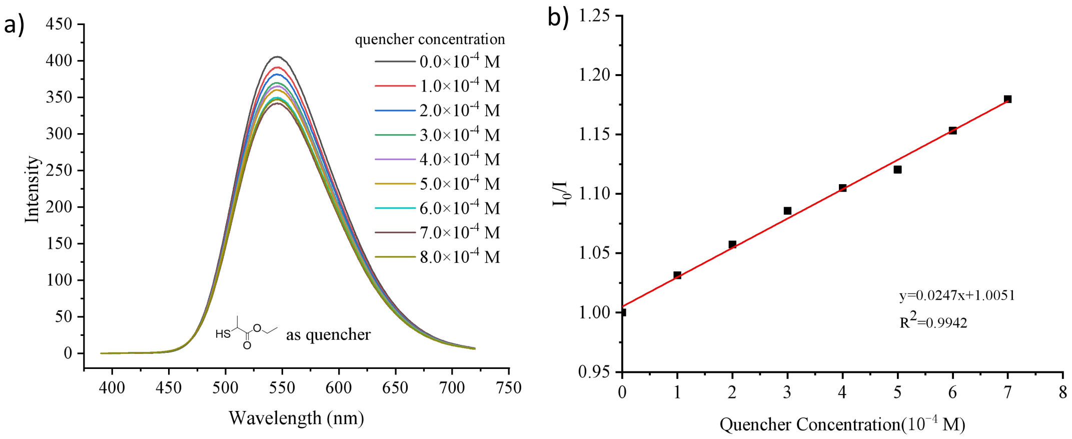

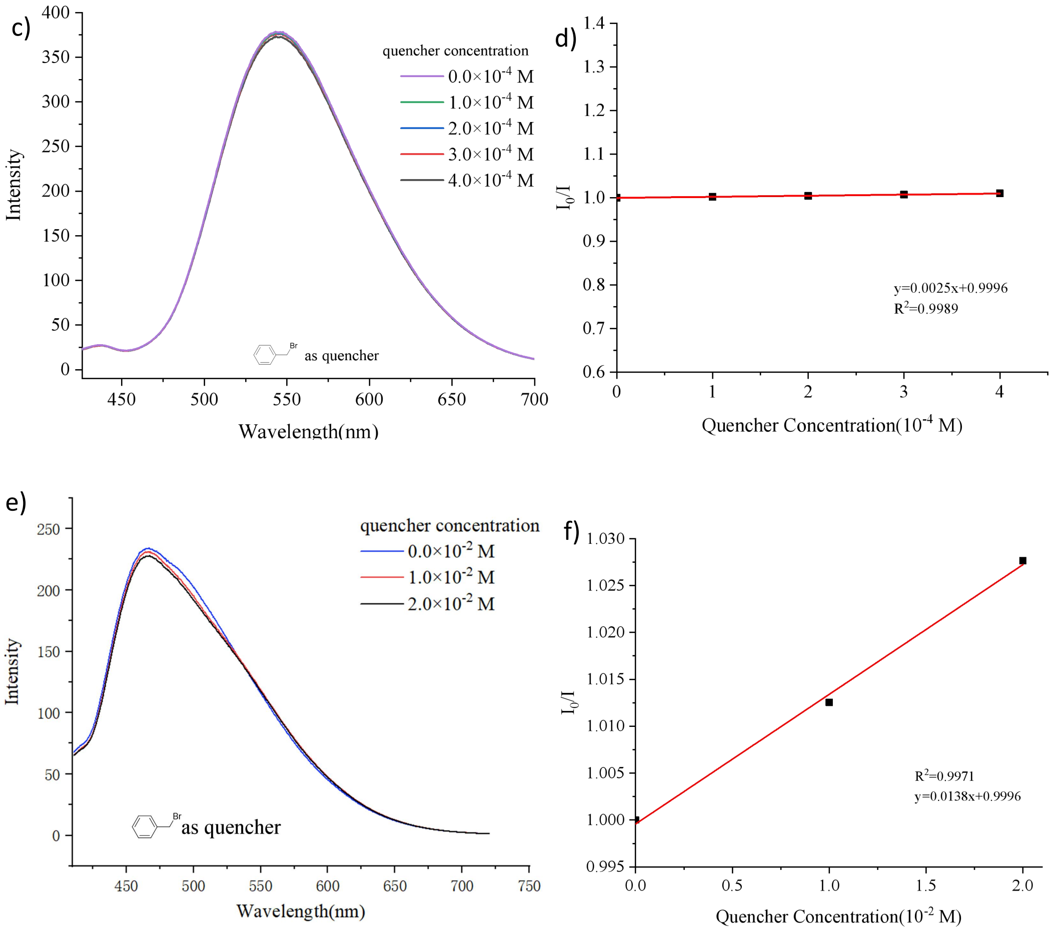

Figure S4. Stern-Volmer fluorescence quenching study

In a typical experiment, a solution of 4CzIPN in anhydrous DMSO (1.25×10^{-4} M) was added with an appropriate amount of quencher in a quartz cuvette. Then the emission of the sample was collected. The emission intensity at 554 nm was collected with excited wavelength of 361 nm, as show in **Figure S4a-d**.

A mixture solution of 4CZIPN (3.2×10^{-5} M) and DIPEA (20 μL) in DMSO in a 1 cm quartz cuvette under argon atmosphere was irradiated with a 440 nm blue lamp (24 W) for 10 min to ensure the full conversion of 4CzIPN. An appropriate amount of quencher, the (bromomethyl)benzene, was added to the irradiated solution via syringe. The emission intensity at 462 nm was collected with excited wavelength of 361 nm, as show in **Figure S4e** and **f**.

6.4 Quantum yield experiment

Determination of the light intensity at 470 nm: Following Yoon's procedure,[12] the photon flux of the spectrophotometer was determined by standard ferrioxalate actinometry. A 0.15 M solution of ferrioxalate was prepared by dissolving 2.21 g of potassium ferrioxalate hydrate in 30 mL of 0.05 M H_2SO_4. A buffered solution of

phenanthroline was prepapred by dissoving 50 mg of phenanthroline and 11.25 g of sodium aceate in 50 mL of 0.5 M H_2SO_4. Both solutions were stored in the dark. To determine the photon flux of the spectrophotometer, 2.0 mL of the ferrioxalate solution was placed solution was placed in a cuvette and irradiated for 90.0 seconds at λ = 470 nm with an emssion slit width at 10.0 nm. After irradiation, 0.35 mL of the phenanthroline solution was added to the cuvette. The solution was then allowed to rest for 1 h to allow the ferrous ions to completely coordinate to the phenanthroline. The absorbance of the solution was measured at 510 nm. A non-irradinated sample was also prepared and the absorbance at 510 nm measured. Conversion was calculates using eq (1).

Where *v* is the total volume (0.00235 L) of the solution after of phenanthroline, *ΔA* is the difference in absorbance at 510 nm between the irradiated and non-irradiated solution, l is the path length (1.000 cm), and ε is the molar absorptivity at 510 nm (11,100 $L \cdot mol^{-1} \cdot cm^{-1}$). The photon flux can be calculated using eq (2).

Where φ is the quantum yield for the ferrioxalate actinometer (0.92 for a 0.15 M solution at λ = 468 nm), t is the time (90.0 s), and f is the fraction of light absorbed at λ = 440 nm. The photo flux was calculated (average of three experiments) to be 4.705×10^{-9} $einstein^{-1}$.

$$molFe^{2+} = \frac{v \bullet \Delta A}{l \bullet \varepsilon} \quad (1)$$

$$photo\ flux = \frac{molFe^{2+}}{\varphi \bullet t \bullet f} \quad (2)$$

$$molFe^{2+} = \frac{0.00235\mathrm{L} \times (1.363 - 0.762)}{1.000\mathrm{cm} \times 11100\mathrm{L}/(\mathrm{mol} \bullet \mathrm{cm})} = 1.245 \times 10^{-7}\mathrm{mol} \quad (3)$$

$$photo\ flux = \frac{1.245 \times 10^{-7}\,\mathrm{mol}}{0.92 \times 90\mathrm{s} \times 0.648} = 2.321 \times 10^{-9}\,\mathrm{mol} \quad (4)$$

Determine the quantum yield:

To a 25 mL Schlenk tube equipped with a magnetic stir bar was added the 4CzIPN (PC **1**, 4.7 mg, 0.006 mmol). The Schlenk tube was sealed and degassed via vacuum evacuation and subsequently backfilled with argon three times. After that anhydrous DMSO-d_3 (2 mL), *p*-methoxybenzyl chloride (29 μL, 0.2 mmol), H_2O (72 μL, 20 equiv.), ethyl 2-mercaptopropanoate (HAT cat. **1**, 5.2 μL, 0.02 mmol) were added sequentially by means of syringe. Then the reaction was placed under blue

LED irradiated for 12 h. The yield of product **2** was determined as 27% by crude ^{1}H NMR based on a 1,3,5-trimethoxybenzene standard. The quantum yield was determined using eq (5). Essentially all incident light (f > 0.999, vide infra) is absorbed by the 4CzIPN at the reaction conditions described above φ = 0.531.

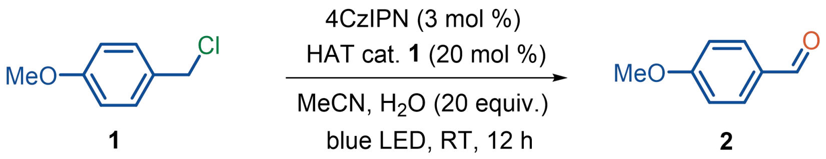

$$\varphi = \frac{\text{mol product}}{\textit{photo flux} \bullet t \bullet f} \tag{5}$$

$$\varphi = \frac{5.4 \times 10^{-5}\,\text{mol}}{2.356 \times 10^{-9} \times 43200 \times 1.00} = 0.531 \tag{6}$$

6.5 Light on-off experiments

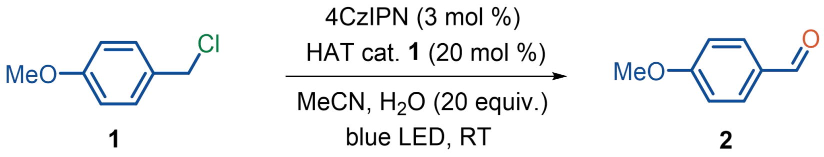

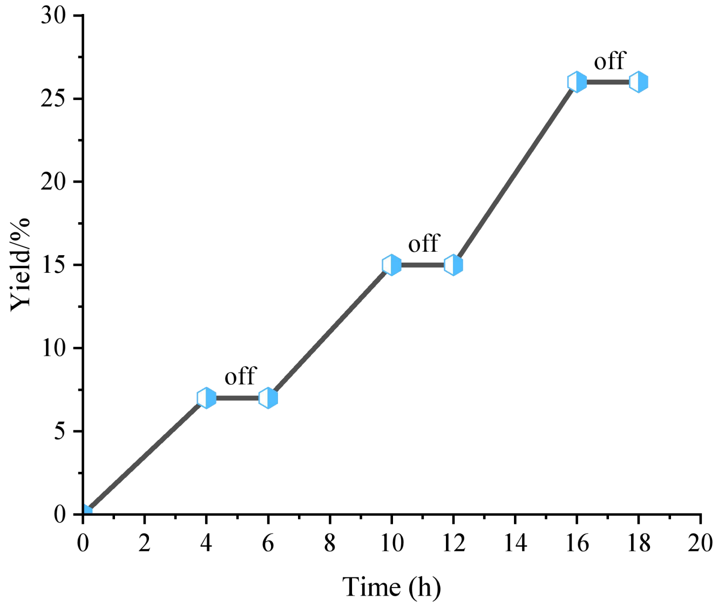

Figure S5. Light on-off Experiment.

6.6 Kinetic isotope effect studies

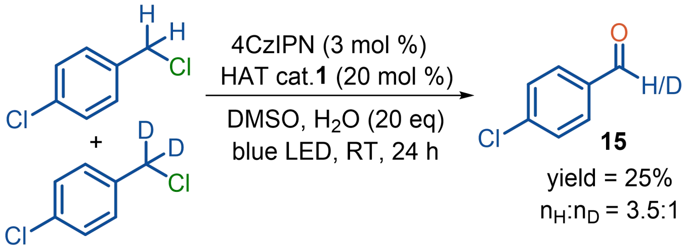

To a 25 mL Schlenk tube equipped with a magnetic stir bar was added the 4CzIPN (PC **1**, 4.7 mg, 0.006 mmol), *p*-chlorobenzyl chloride (16 mg, 0.1 mmol) and *p*-chlorobenzyl chloride-d_2 (99%-D incorporation) (18 mg, 0.1 mmol),. The Schlenk tube was sealed and degassed via vacuum evacuation and subsequently backfilled with argon three times. After that anhydrous DMSO (2 mL), H_2O (72 μL, 20 equiv.), ethyl 2-mercaptopropanoate (HAT cat. **1**, 5.2 μL, 0.02 mmol) were added sequentially by means of syringe. Then the reaction was placed under blue LED irradiated for 24 h. After that, saturated brine (5 mL) was added and the mixture was extracted with ethyl acetate (3 × 5 mL). The combined organic layer was dried with anhydrous $MgSO_4$. The solvent was removed on a rotary evaporator under reduced pressure. Purification by column chromatography on silica gel gave the desired pure product **15**. The C−H/D ratio (3.5 : 1) was obtained in the product **15** according to ^1H NMR analysis, as shown in **Figure S6**.

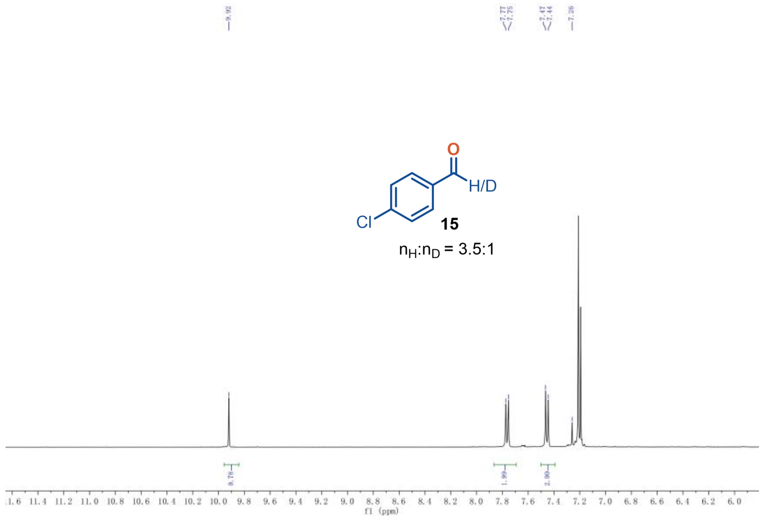

Figure S6. ^1H NMR spectra of the product ($n_H : n_D = 3.5:1$)

6.7 ^{18}O labelled experiments

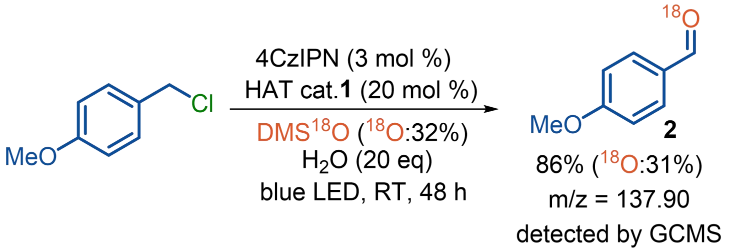

DMS[18]O was used as a solvent: To a 25 mL Schlenk tube equipped with a magnetic stir bar was added the 4CzIPN (PC **1**, 4.7 mg, 0.006 mmol). The Schlenk tube was sealed and degassed via vacuum evacuation and subsequently backfilled with argon three times. After that anhydrous ^{18}O-labeled dimethyl sulfoxide (DMSO) (2 mL), *p*-methoxybenzyl chloride (29 μL, 0.2 mmol), H_2O (72 μL, 20 equiv.), ethyl 2-mercaptopropanoate (HAT cat. **1**, 5.2 μL, 0.02 mmol) were added sequentially by means of syringe. Then the reaction was placed under blue LED irradiated for 24 h. After that, saturated brine (5 mL) was added and the mixture was extracted with ethyl acetate (3 × 5 mL). The combined organic layer was dried with anhydrous $MgSO_4$. The solvent was removed on a rotary evaporator under reduced pressure. The crude reaction mixture was analyzed by GC-MS (**Figure S7a**). Purification by column chromatography on silica gel gave the desired pure product in 86% yield.

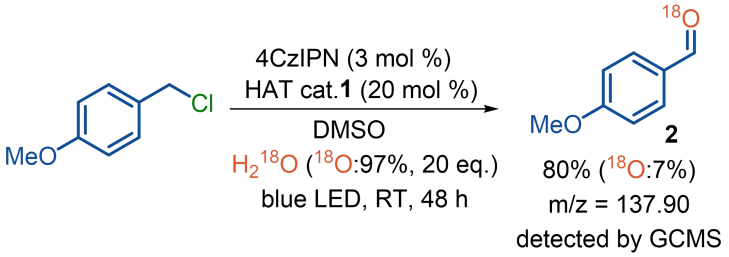

$H_2{}^{18}O$ as co-solvent: To a 25 mL Schlenk tube equipped with a magnetic stir bar was added the 4CzIPN (PC **1**, 4.7 mg, 0.006 mmol). The Schlenk tube was sealed and degassed via vacuum evacuation and subsequently backfilled with argon three times. After that anhydrous DMSO (2 mL), *p*-methoxybenzyl chloride (29 μL, 0.2 mmol), $H_2{}^{18}O$ (72 μL, 20 equiv.), ethyl 2-mercaptopropanoate (HAT cat. **1**, 5.2 μL, 0.02 mmol) were added sequentially by means of syringe. Then the reaction was placed under blue LED irradiated for 24 h. After that, saturated brine (5 mL) was added and the mixture was extracted with ethyl acetate (3 × 5 mL). The combined organic layer was dried with anhydrous $MgSO_4$. The solvent was removed on a rotary evaporator under reduced pressure. The crude reaction mixture was analyzed by GC-MS (**Figure S7b**). Purification by column chromatography on silica gel gave the desired pure product in

80% yield.

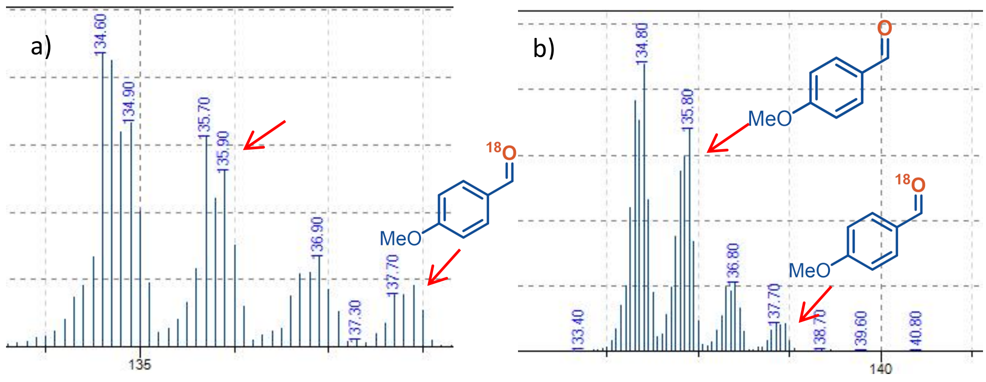

Figure S7. GCMS analysis of ^{18}O labeling experiment.

6.8 Transformation of alkoxylsulfonium salt intermediate

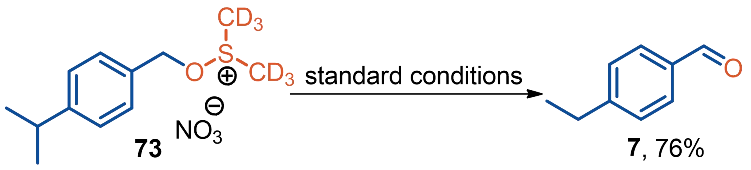

To a 25 mL Schlenk tube equipped with a magnetic stir bar was added the 4CzIPN (PC **1**, 4.7 mg, 0.006 mmol). The Schlenk tube was sealed and degassed via vacuum evacuation and subsequently backfilled with argon three times. After that the alkoxylsulfonium ion solution in DMSO (0.1 M, 2 mL), ethyl 2-mercaptopropanoate (HAT cat. **1**, 5.2 μL, 0.02 mmol) were added sequentially by means of syringe. Then the reaction was placed under blue LED irradiated for 24 h. After that, saturated brine (5 mL) was added and the mixture was extracted with ethyl acetate (3 × 5 mL). The combined organic layer was dried with anhydrous $MgSO_4$. The solvent was removed on a rotary evaporator under reduced pressure. Purification by column chromatography on silica gel gave the desired pure product **7**.

6.9 Deuterium labeling experiment

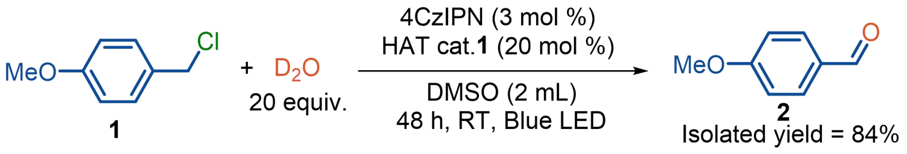

To a 25 mL Schlenk tube equipped with a magnetic stir bar was added the 4CzIPN (PC **1**, 4.7 mg, 0.006 mmol). The Schlenk tube was sealed and degassed via vacuum evacuation and subsequently backfilled with argon three times. After that anhydrous DMSO (2 mL), *p*-methoxybenzyl chloride (29 μL, 0.2 mmol), D_2O (72 μL, 20 equiv.), ethyl 2-mercaptopropanoate (HAT cat. **1**, 5.2 μL, 0.02 mmol) were added

sequentially by means of syringe. Then the reaction was placed under blue LED irradiated for 48 h. After that, saturated brine (5 mL) was added and the mixture was extracted with ethyl acetate (3 × 5 mL). The combined organic layer was dried with anhydrous $MgSO_4$. The solvent was removed on a rotary evaporator under reduced pressure. No deuterium incorporation was observed in the isolated product **2** according to ^1H NMR analysis.

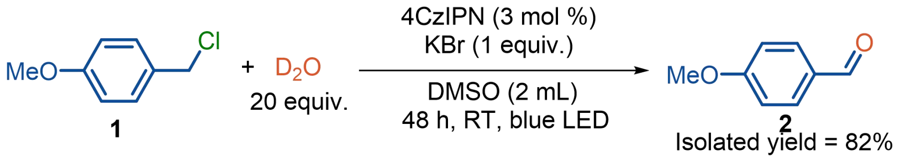

To a 25 mL Schlenk tube equipped with a magnetic stir bar was added the 4CzIPN (PC **1**, 4.7 mg, 0.006 mmol), KBr (23 mg, 1 equiv.). The Schlenk tube was sealed and degassed via vacuum evacuation and subsequently backfilled with argon three times. After that anhydrous DMSO (2 mL), *p*-methoxybenzyl chloride (29 μL, 0.2 mmol), D_2O (72 μL, 20 equiv.) were added sequentially by means of syringe. Then the reaction was placed under blue LED irradiated for 48 h. After that, saturated brine (5 mL) was added and the mixture was extracted with ethyl acetate (3 × 5 mL). The combined organic layer was dried with anhydrous $MgSO_4$. The solvent was removed on a rotary evaporator under reduced pressure. No deuterium incorporation was observed in the isolated product **2** according to ^1H NMR analysis.

6.10 Detection of benzyl sulfide

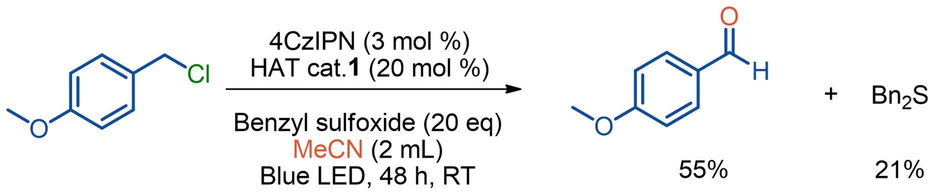

To a 25 mL Schlenk tube equipped with a magnetic stir bar was added the 4CzIPN (PC **1**, 4.7 mg, 0.006 mmol) and benzyl sulfoxide (921.3 mg, 4 mmol). The Schlenk tube was sealed and degassed via vacuum evacuation and subsequently backfilled with argon three times. After that MeCN (2 mL), 4-methoxybenzyl chloride (0.2 mmol), ethyl 2-mercaptopropanoate (HAT cat. **1**, 5.2 μL, 0.02 mmol) were added sequentially by means of syringe. Then the reaction was placed under blue LED irradiated for 48 h. After that, the solvent was removed on a rotary evaporator under reduced pressure. GCMS measurement indicates the generation of the aldehyde, the benzyl sulfide and dibenzyl disulphide. Purification by column chromatography on silica gel gave the aldehyde and the benzyl sulfide in 55% and 21% yields,

respectively.

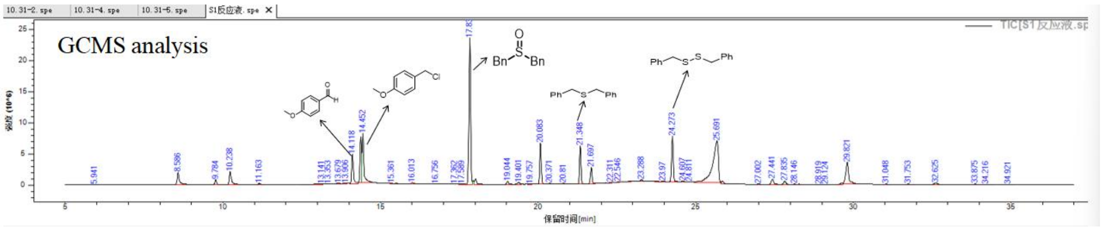

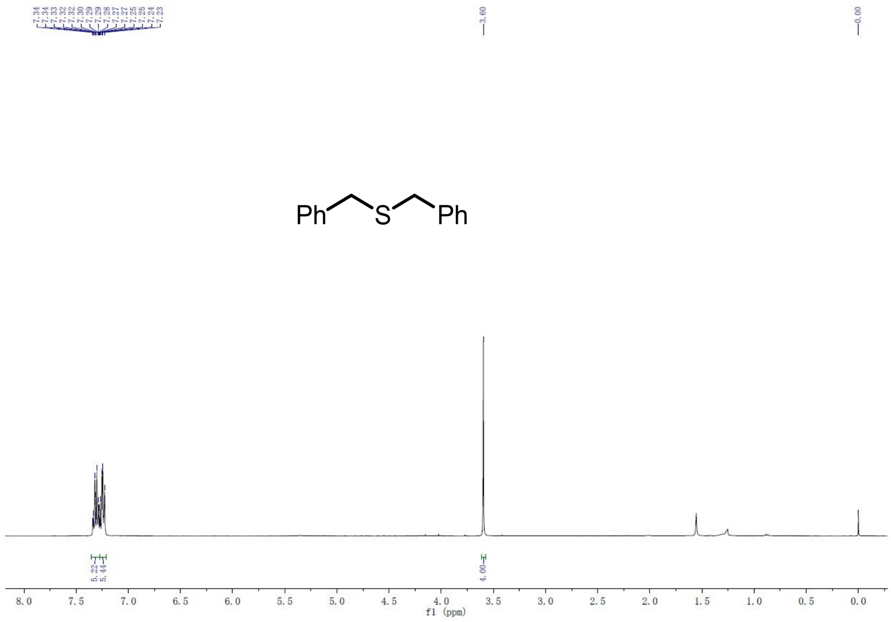

6.11 Plausible mechanism for the dehalocarbonylation reaction in other solvents instead of DMSO

Based on the experimental results and our previous studies on photocatalytic acceptorless dehydrogenation of alcohols,[15] a plausible mechanism for the dehalocarbonylation reaction of the alkyl halides such as 4-methoxybenzyl chloride (**1**) in other solvents instead of DMSO as depicted in Table 1 is illustrated in Figure S8.

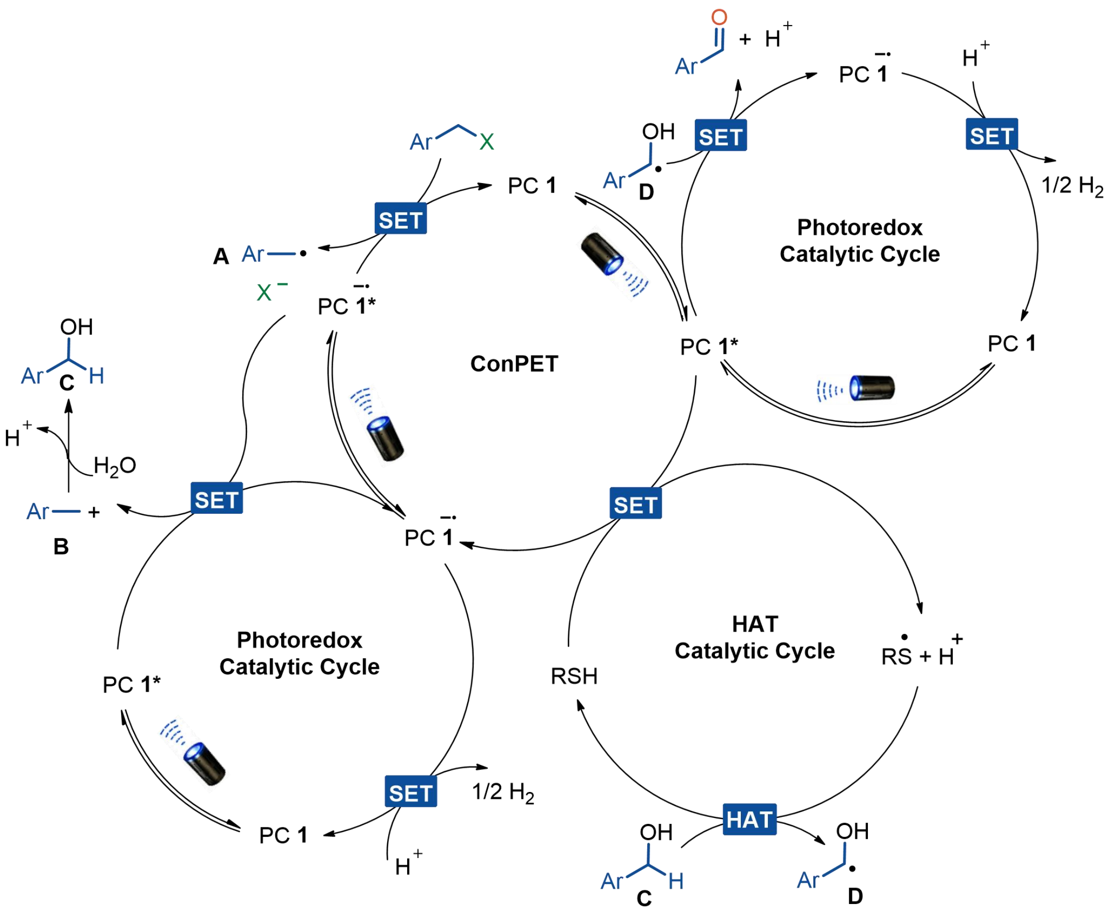

Figure S8. Plausible mechanism for the dehalocarbonylation in other solvents instead of DMSO.

7. Analytical data of the products

4-methoxybenzyl bromide (2)

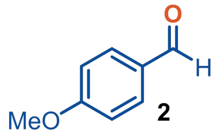

2

Purification by column chromatography (petroleum ether/ethyl acetate = 20:1-7:1) and isolated in 88% yield as a pale yellow oil. **^{1}H NMR** (400 MHz, Chloroform-*d*) δ 9.88 (s, 1H), 7.83 (d, *J* = 8.7 Hz, 2H), 7.00 (d, *J* = 8.7 Hz, 2H), 3.88 (s, 3H) ppm. **^{13}C NMR** (151 MHz, Chloroform-*d*) δ 190.6, 164.4, 131.8, 129.8, 114.1, 55.4 ppm. A known compound and the characterization data are in accordance with the literature.[13]

4-methylbenzaldehyde (4)

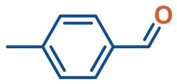

4

Purification by column chromatography (petroleum ether/ethyl acetate = 20:1-10:1) and isolated in 71% yield as a pale yellow oil. **^{1}H NMR** (400 MHz, Chloroform-*d*) δ 9.96 (s, 1H), 7.77 (d, *J* = 7.9 Hz, 2H), 7.33 (d, *J* = 7.8 Hz, 2H), 2.44 (s, 3H) ppm. **^{13}C NMR** (101 MHz, Chloroform-*d*) δ 191.9, 145.4, 134.1, 129.7, 129.6, 21.8 ppm. A known compound and the characterization data are in accordance with the literature.[13]

3-methylbenzaldehyde (5)

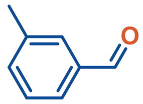

5

Purification by column chromatography (petroleum ether/ethyl acetate = 20:1-10:1) and isolated in 83% yield as a colorless oil. **^{1}H NMR** (400 MHz, Chloroform-*d*) δ 9.98 (s, 1H), 7.68 (d, *J* = 7.5 Hz, 2H), 7.42 (d, *J* = 7.7 Hz, 2H), 2.43 (s, 3H) ppm. **^{13}C NMR** (101 MHz, Chloroform-*d*) δ 192.5, 138.8, 136.3, 135.2, 129.9, 128.7, 127.1, 21.1 ppm.A known compound and the characterization data are in accordance with the literature.[14]

2-methylbenzaldehyde (6)

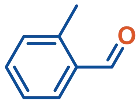

6

Purification by column chromatography (petroleum ether/ethyl acetate = 20:1-10:1) and isolated in 63% yield from 1-(chloromethyl)-2-methylbenzene and 87% yield from 1-(bromomethyl)-2-methylbenzene as a pale yellow oil. ^{1}**H NMR** (400 MHz, Chloroform-*d*) δ 10.25 (s, 1H), 7.79 (d, J = 7.7 Hz, 1H), 7.49 – 7.43 (m, 1H), 7.38 – 7.32 (m, 1H), 7.25 (d, J = 7.6 Hz, 1H), 2.66 (s, 3H) ppm. ^{13}**C NMR** (101 MHz, Chloroform-*d*) δ 192.7, 140.5, 134.0, 133.5, 131.9, 131.6, 126.2, 19.5 ppm. A known compound and the characterization data are in accordance with the literature.[13]

4-isopropylbenzaldehyde (7)

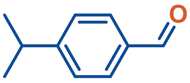

7

Purification by column chromatography (petroleum ether/ethyl acetate = 20:1-10:1) and isolated in 74% yield from 1-(chloromethyl)-4- isopropylbenzene and 64% yield from 1-(bromomethyl)-4-isopropylbenzene as a colorless oil. ^{1}**H NMR** (400 MHz, Chloroform-*d*) δ 9.97 (s, 1H), 7.81 (d, *J* = 7.9 Hz, 2H), 7.39 (d, *J* = 7.9 Hz, 2H), 2.99 (p, *J* = 6.9 Hz, 1H), 1.28 (d, *J* = 7.0 Hz, 6H) ppm. ^{13}**C NMR** (101 MHz, Chloroform-*d*) δ 191.9, 156.1, 134.4, 129.9, 127.0, 34.4, 23.5 ppm. A known compound and the characterization data are in accordance with the literature.[13]

4-(*tert*-butyl)benzaldehyde (8)

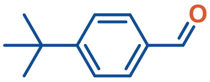

8

Purification by column chromatography (petroleum ether/ethyl acetate = 20:1-10:1) and isolated in 68% yield as a colorless oil. ^{1}**H NMR** (400 MHz, Chloroform-*d*) δ 9.98 (s, 1H), 7.82 (d, *J* = 8.4 Hz, 2H), 7.55 (d, *J* = 8.3 Hz, 2H), 1.35 (s, 9H) ppm. ^{13}**C NMR** (151 MHz, Chloroform-*d*) δ 191.9, 158.3, 134.0, 129.6, 125.9, 35.3, 31.0 ppm. A known compound and the characterization data are in accordance with the literature.[13]

[1,1'-biphenyl]-4-carbaldehyde (9)

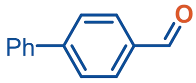

9

Purification by column chromatography (petroleum ether/ethyl acetate = 20:1-7:1) and isolated in 62% yield from 4-(chloromethyl)-1,1' -biphenyl and 89% yield from 4-(bromomethyl)-1,1'-biphenyl as a white solid. **^{1}H NMR** (400 MHz, Chloroform-*d*) δ 10.04 (s, 1H), 8.02 – 7.88 (m, 2H), 7.84 – 7.69 (m, 2H), 7.63 (d, *J* = 7.8 Hz, 2H), 7.47 (t, *J* = 7.6 Hz, 2H), 7.43 – 7.38 (m, 1H) ppm. **^{13}C NMR** (101 MHz, Chloroform-*d*) δ 191.8, 147.0, 139.6, 135.1, 130.1, 128.9, 128.4, 127.5, 127.2 ppm. A known compound and the characterization data are in accordance with the literature.[15]

4'-formyl-[1,1'-biphenyl]-2-carbonitrile (10)

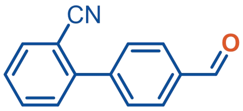

10

Purification by column chromatography (petroleum ether/ethyl acetate = 20:1-5:1) and isolated in 84% yield as a yellow solid. **^{1}H NMR** (400 MHz, Chloroform-*d*) δ 10.10 (s, 1H), 8.02 (d, *J* = 8.3 Hz, 2H), 7.82 (d, *J* = 8.5 Hz, 1H), 7.74 (d, *J* = 8.2 Hz, 2H), 7.69 (d, *J* = 7.8 Hz, 1H), 7.55 (d, *J* = 8.0 Hz, 1H), 7.52 (d, *J* = 8.8 Hz, 1H) ppm. **^{13}C NMR** (151 MHz, Chloroform-*d*) δ 191.6, 143.9, 143.8, 136.1, 133.8, 133.0, 130.0, 129.9, 129.5, 128.4, 118.1, 111.2 ppm. A known compound and the characterization data are in accordance with the literature.[16]

4-(methylthio)benzaldehyde (11)

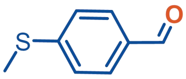

11

Purification by column chromatography (petroleum ether/ethyl acetate = 20:1-7:1) and isolated in 88% yield as a pale yellow oil. **^{1}H NMR** (400 MHz, Chloroform-*d*) δ 9.92 (s, 1H), 7.77 (d, *J* = 8.5 Hz, 2H), 7.32 (d, *J* = 8.4 Hz, 2H), 2.53 (s, 3H) ppm. **^{13}C NMR** (101 MHz, Chloroform-*d*) δ 191.1, 147.8, 132.8, 129.9, 125.1, 14.6 ppm. A known compound and the characterization data are in accordance with the literature.[13]

3-methoxybenzaldehyde (12)

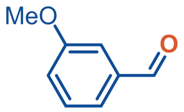

12

Purification by column chromatography (petroleum ether/ethyl acetate = 20:1-7:1) and isolated in 74% yield as a colorless oil. **^{1}H NMR** (400 MHz, Chloroform-*d*) δ 9.98 (s, 1H), 7.48 – 7.44 (m, 2H), 7.40 (d, *J* = 2.0 Hz, 1H), 7.21 – 7.06 (m, 1H), 3.87 (s, 3H) ppm. **^{13}C NMR** (101 MHz, Chloroform-*d*) δ 192.1, 160.1, 137.7, 130.0, 123.5, 121.5, 111.9, 55.4 ppm. A known compound and the characterization data are in accordance with the literature.[13]

4-(benzyloxy)benzaldehyde (13)

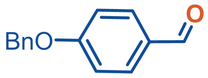

13

Purification by column chromatography (petroleum ether/ethyl acetate = 20:1-7:1) and isolated in 94% yield as a pale yellow solid. **^{1}H NMR** (400 MHz, Chloroform-*d*) δ 9.88 (s, 1H), 7.84 (d, *J* = 8.8 Hz, 2H), 7.58 – 7.31 (m, 5H), 7.08 (d, *J* = 8.7 Hz, 2H), 5.15 (s, 2H) ppm. **^{13}C NMR** (151 MHz, Chloroform-*d*) δ 190.6, 163.7, 135.9, 131.9, 128.7, 128.3, 127.4, 115.1, 70.2 ppm. A known compound and the characterization data are in accordance with the literature.[13]

4-fluorobenzaldehyde (14)

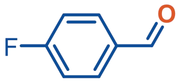

14

Purification by column chromatography (petroleum ether/ethyl acetate = 15:1-7:1) and isolated in 73% yield as a pale yellow solid. **^{1}H NMR** (400 MHz, Chloroform-*d*) δ 9.98 (s, 1H), 8.23 – 7.82 (m, 2H), 7.22 (t, *J* = 8.5 Hz, 2H) ppm. **^{13}C NMR** (101 MHz, Chloroform-*d*) δ 196.3, 165.6 (d, *J* = 254.6 Hz), 133.4 (d, *J* = 3.0 Hz), 130.8 (d, *J* = 9.4 Hz), 115.5 (d, *J* = 22.0 Hz), 26.4 ppm. **^{19}F NMR** (376 MHz, Chloroform-*d*) δ -102.4 ppm. A known compound and the characterization data are in accordance with the literature.[17]

4-chlorobenzaldehyde (15)

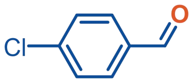

15

Purification by column chromatography (petroleum ether/ethyl acetate = 15:1-5:1) and isolated in 89% yield from 1-chloro-4-(chloromethyl)benzene and 63% yield from 1-(bromomethyl)-4-chlorobenzene as a pale yellow solid. **^{1}H NMR** (400 MHz, Chloroform-*d*) δ 9.98 (s, 1H), 7.82 (d, *J* = 8.5 Hz, 2H), 7.51 (d, *J* = 8.5 Hz, 2H) ppm. **^{13}C NMR** (151 MHz, Chloroform-*d*) δ 190.7, 140.8, 134.6, 130.8, 129.3 ppm. A known compound and the characterization data are in accordance with the literature.[13]

3-chlorobenzaldehyde (16)

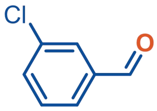

16

Purification by column chromatography (petroleum ether/ethyl acetate = 15:1-5:1) and isolated in 75% yield as a colorless oil. **^{1}H NMR** (400 MHz, Chloroform-*d*) δ 9.98 (s, 1H), 7.85 (d, *J* = 2.1 Hz, 1H), 7.77 (d, *J* = 7.6 Hz, 1H), 7.60 (dd, *J* = 8.2, 1.9 Hz, 1H), 7.49 (t, *J* = 7.8 Hz, 1H) ppm. **^{13}C NMR** (101 MHz, Chloroform-*d*) δ 190.7, 137.6, 135.3, 134.2, 130.2, 129.1, 127.9 ppm. A known compound and the characterization data are in accordance with the literature.[15]

2-chlorobenzaldehyde (17)

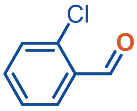

17

Purification by column chromatography (petroleum ether/ethyl acetate = 15:1-5:1) and isolated in 68% yield from 1-chloro-2-(chloromethyl)benzene and 64% yield from 1-(bromomethyl)-2-chlorobenzeneae as a colorless oil. **^{1}H NMR** (400 MHz, Chloroform-*d*) δ 10.47 (s, 1H), 7.91 (dd, J = 7.8, 1.8 Hz, 1H), 7.59 – 7.48 (m, 1H), 7.44 (d, J = 7.0 Hz, 1H), 7.38 (t, J = 7.5 Hz, 1H) ppm. **^{13}C NMR** (101 MHz, Chloroform-d) δ 189.7, 137.8, 135.0, 132.3, 130.5, 129.2, 127.2 ppm. A known compound and the characterization data are in accordance with the literature.[13]

4-bromobenzaldehyde (18)

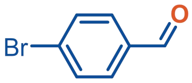

18

Purification by column chromatography (petroleum ether/ethyl acetate = 15:1-5:1) and isolated in 83% yield from 1-bromo-4- (chloromethyl)benzene and 73% yield from 1-bromo-4-(bromomethyl)benzene as a white solid. **^{1}H NMR** (400 MHz,

Chloroform-*d*) δ 9.98 (s, 1H), 7.75 (d, *J* = 8.5 Hz, 2H), 7.69 (d, *J* = 8.4 Hz, 2H) ppm. **^{13}C NMR** (101 MHz, Chloroform-*d*) δ 191.0, 135.0, 132.4, 130.9, 129.7 ppm. A known compound and the characterization data are in accordance with the literature.[15]

4-formylbenzonitrile (19)

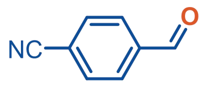

19

Purification by column chromatography (petroleum ether/ethyl acetate = 15:1-3:1) and isolated in 72% yield from 4-(chloromethyl)benzonitrile and 80% yield from 4-(bromomethyl)benzonitrileas as a white solid. **^{1}H NMR** (400 MHz, Chloroform-*d*) δ 10.12 (s, 1H), 8.02 (d, *J* = 8.3 Hz, 2H), 7.87 (d, *J* = 8.2 Hz, 2H) ppm. **^{13}C NMR** (151 MHz, Chloroform-*d*) δ 190.5, 138.6, 132.8, 129.7, 117.6, 117.4 ppm. A known compound and the characterization data are in accordance with the literature.[13]

4-(trifluoromethyl)benzaldehyde (20)

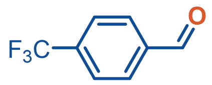

20

Purification by column chromatography (petroleum ether/ethyl acetate = 15:1-5:1) and isolated in 43% yield as a colorless oil. **^{1}H NMR** (400 MHz, Chloroform-*d*) δ 10.11 (s, 1H), 8.02 (d, *J* = 8.0 Hz, 2H), 7.82 (d, *J* = 8.2 Hz, 2H) ppm. **^{13}C NMR** (101 MHz, Chloroform-*d*) δ 201.0 – 184.3 (m), 138.6 – 138.5 (m), 135.5 (q, *J* = 32.7 Hz), 129.9, 126.0 (q, *J* = 3.8 Hz), 123.4 (d, *J* = 272.9 Hz) ppm. **^{19}F NMR** (376 MHz, Chloroform-*d*) δ -63.4 ppm. A known compound and the characterization data are in accordance with the literature.[18]

4-nitrobenzaldehyde (21)

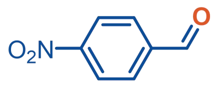

21

Purification by column chromatography (petroleum ether/ethyl acetate = 15:1-3:1) and isolated in 65% yield from 1-(chloromethyl)-4-nitrobenzene and 54% yield from 1-(bromomethyl)-4-nitrobenzene as a pale yellow solid. **^{1}H NMR** (400 MHz, Chloroform-*d*) δ 10.18 (s, 1H), 8.41 (d, *J* = 7.5 Hz, 2H), 8.10 (d, *J* = 7.5 Hz, 2H) ppm. **^{13}C NMR** (101 MHz, Chloroform-*d*) δ 190.2, 151.0, 139.9, 130.4, 124.2 ppm. A

known compound and the characterization data are in accordance with the literature.[13]

2-nitrobenzaldehyde (22)

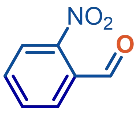

22

Purification by column chromatography (petroleum ether/ethyl acetate = 15:1-3:1) and isolated in 26% yield as a yellow solid. **^{1}H NMR** (400 MHz, Chloroform-*d*) δ 10.42 (s, 1H), 8.13 (d, J = 7.6 Hz, 1H), 7.96 (d, J = 7.3 Hz, 1H), 7.89 – 7.69 (m, 2H) ppm. **^{13}C NMR** (101 MHz, Chloroform-*d*) δ 188.0, 149.4, 134.0, 133.6, 131.2, 129.5, 124.4 ppm. A known compound and the characterization data are in accordance with the literature.[19]

4-formylbenzaldehyde (23)

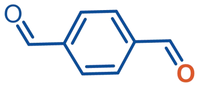

23

Purification by column chromatography (petroleum ether/ethyl acetate = 15:1-5:1) and isolated in 86% yield as a white solid. **^{1}H NMR** (400 MHz, Chloroform-*d*) δ 10.15 (s, 2H), 8.07 (s, 4H) ppm. **^{13}C NMR** (151 MHz, Chloroform-*d*) δ 191.4, 140.0, 130.1 ppm. A known compound and the characterization data are in accordance with the literature.[20]

***tert*-butyl 4-formylbenzoate (24)**

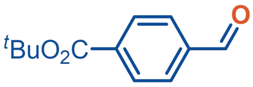

24

Purification by column chromatography (petroleum ether/ethyl acetate = 15:1-7:1) and isolated in 63% yield as a white solid. **^{1}H NMR** (400 MHz, Chloroform-*d*) δ 10.10 (s, 1H), 8.14 (d, J = 8.3 Hz, 2H), 7.93 (d, J = 8.4 Hz, 2H), 1.62 (s, 9H) ppm. **^{13}C NMR** (151 MHz, Chloroform-*d*) δ 191.7, 164.6, 138.7, 137.0, 129.9, 129.3, 82.0, 28.1 ppm. A known compound and the characterization data are in accordance with the literature.[21]

ethyl 4-formylbenzoate (25)

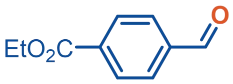

25

Purification by column chromatography (petroleum ether/ethyl acetate = 15:1-7:1) and isolated in 89% yield as a pale yellow solid. ^{1}**H NMR** (400 MHz, Chloroform-*d*) δ 10.11 (s, 1H), 8.21 (d, *J* = 8.3 Hz, 2H), 7.96 (d, *J* = 8.3 Hz, 2H), 4.42 (q, *J* = 7.1 Hz, 2H), 1.43 (t, *J* = 7.1 Hz, 3H) ppm. ^{13}**C NMR** (101 MHz, Chloroform-*d*) δ 191.68, 165.56, 139.04, 135.44, 130.12, 129.46, 61.59, 14.24 ppm. A known compound and the characterization data are in accordance with the literature.[22]

4-formylbenzoic acid (26)

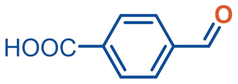

26

Purification by column chromatography (petroleum ether/ethyl acetate = 5:1-1:1) and isolated in 43% yield as a white solid. ^{1}**H NMR** (400 MHz, Chloroform-*d*) δ 10.13 (s, 1H), 8.15 (d, *J* = 8.0 Hz, 2H), 8.04 (d, *J* = 8.0 Hz, 2H) ppm. ^{13}**C NMR** (101 MHz, DMSO-d_6) δ 193.4, 167.0, 139.3, 136.1, 130.4, 130.0 ppm. A known compound and the characterization data are in accordance with the literature.[23]

(4-formylphenyl)boronic acid (27)

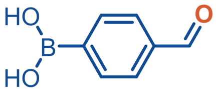

27

Purification by column chromatography (petroleum ether/ethyl acetate = 5:1-1:1) and isolated in 92% yield as a white solid. ^{1}**H NMR** (400 MHz, DMSO-d_6) δ 10.05 (d, *J* = 9.8 Hz, 1H), 8.06 (dd, *J* = 50.3, 7.7 Hz, 2H), 7.97 – 7.82 (m, 2H) ppm. ^{13}**C NMR** (101 MHz, DMSO-d_6) δ 194.0, 137.6, 135.0, 134.4, 128.8 ppm.A known compound and the characterization data are in accordance with the literature.[24]

2,6-dimethylbenzaldehyde (28)

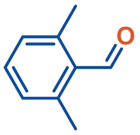

28

Purification by column chromatography (petroleum ether/ethyl acetate = 20:1-10:1) and isolated in 26% yield as a white solid. ^{1}**H NMR** (400 MHz, Chloroform-*d*) δ 10.54 (s, 1H), 7.23 (t, *J* = 7.6 Hz, 1H), 7.00 (d, *J* = 7.6 Hz, 2H), 2.53 (s, 6H) ppm. ^{13}**C NMR** (101 MHz, Chloroform-*d*) δ 193.4, 141.0, 132.9, 132.3, 129.6, 20.4 ppm. A known compound and the characterization data are in accordance with the literature.[25]

3,4-dimethoxybenzaldehyde (29)

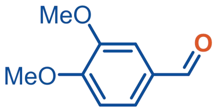

29

Purification by column chromatography (petroleum ether/ethyl acetate = 20:1-5:1) and isolated in 45% yield as a white solid. **^{1}H NMR** (400 MHz, Chloroform-*d*) δ 9.79 (s, 1H), 7.39 (dd, *J* = 8.2, 1.9 Hz, 1H), 7.34 (d, *J* = 1.9 Hz, 1H), 6.91 (d, *J* = 8.1 Hz, 1H), 3.90 (s, 3H), 3.88 (s, 3H) ppm. **^{13}C NMR** (151 MHz, Chloroform-*d*) δ 190.8, 154.4, 149.6, 130.1, 126.7, 110.3, 108.9, 56.1, 55.9 ppm. A known compound and the characterization data are in accordance with the literature.[26]

3,5-dimethoxybenzaldehyde (30)

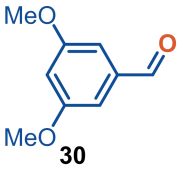

30

Purification by column chromatography (petroleum ether/ethyl acetate = 20:1-5:1) and isolated in 84% yield as a white solid. **^{1}H NMR** (400 MHz, Chloroform-*d*) δ 9.91 (s, 1H), 7.02 (d, *J* = 2.3 Hz, 2H), 6.71 (t, *J* = 2.3 Hz, 1H), 3.85 (s, 6H) ppm. **^{13}C NMR** (151 MHz, Chloroform-*d*) δ 191.9, 161.2, 138.4, 107.1 (d, *J* = 9.1 Hz), 55.6 ppm. A known compound and the characterization data are in accordance with the literature.[15]

3,4,5-trimethoxybenzaldehyde (31)

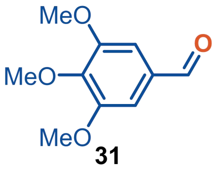

31

Purification by column chromatography (petroleum ether/ethyl acetate = 20:1-5:1) and isolated in 66% yield as a white solid. **^{1}H NMR** (400 MHz, Chloroform-*d*) δ 9.86 (s, 1H), 7.13 (s, 2H), 3.94 (d, *J* = 4.0 Hz, 9H) ppm. **^{13}C NMR** (151 MHz, Chloroform-*d*) δ 190.7, 153.3, 143.3, 131.4, 106.4, 60.6, 55.9 ppm. A known compound and the characterization data are in accordance with the literature.[27]

3-fluoro-4-methoxybenzaldehyde (32)

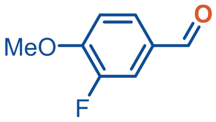

32

Purification by column chromatography (petroleum ether/ethyl acetate = 20:1-7:1) and isolated in 91% yield as a white solid. **^{1}H NMR** (400 MHz, Chloroform-*d*) δ 9.88 (d, *J* = 2.2 Hz, 1H), 7.80 (d, *J* = 9.2 Hz, 1H), 7.70 (dd, *J* = 11.4, 1.9 Hz, 1H), 7.40 (t, *J* = 8.3 Hz, 1H), 3.96 (s, 3H) ppm. **^{13}C NMR** (101 MHz, DMSO-d_6) δ 191.2, 153.1 (d, *J* = 30.5 Hz), 150.8, 130.1 (d, *J* = 5.1 Hz), 128.9 (d, *J* = 3.0 Hz), 115.5 (d, *J* = 18.1 Hz), 114.2 (d, *J* = 1.9 Hz), 56.9 ppm. **^{19}F NMR** (376 MHz, Chloroform-*d*) δ -133.4 ppm. A known compound and the characterization data are in accordance with the literature.[28]

5-fluoro-2-methoxybenzaldehyde (33)

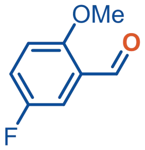

33

Purification by column chromatography (petroleum ether/ethyl acetate = 20:1-7:1) and isolated in 96% yield as a white solid. **^{1}H NMR** (400 MHz, Chloroform-*d*) δ 10.42 (s, 1H), 7.51 (dd, *J* = 8.2, 3.3 Hz, 1H), 7.26 (ddd, *J* = 9.0, 7.5, 3.3 Hz, 1H), 6.96 (dd, *J* = 9.1, 3.8 Hz, 1H), 3.93 (s, 3H) ppm. **^{13}C NMR** (151 MHz, Chloroform-*d*) δ 188.6, 157.9 (d, *J* = 68.0 Hz), 156.1, 125.5, 122.4 (d, *J* = 23.9 Hz), 114.0 (d, *J* = 23.4 Hz), 113.1 (d, *J* = 7.3 Hz), 56.1 ppm. **^{19}F NMR** (376 MHz, Chloroform-*d*) δ -122.8 ppm. A known compound and the characterization data are in accordance with the literature.[28]

2,2-difluorobenzo[*d*][1,3]dioxole-5-carbaldehyde (34)

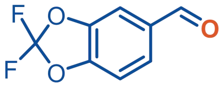

34

Purification by column chromatography (petroleum ether/ethyl acetate = 20:1-7:1) and isolated in 72% yield as a colorless oil. **^{1}H NMR** (400 MHz, Chloroform-*d*) δ 9.90 (s, 1H), 7.66 (dd, J = 8.1, 1.6 Hz, 1H), 7.58 (d, J = 1.6 Hz, 1H), 7.22 (d, J = 8.2 Hz, 1H) ppm. **^{13}C NMR** (101 MHz, Chloroform-*d*) δ 189.7, 146.3 (d,

J = 354.6 Hz), 134.1, 133.1, 131.6, 128.6, 109.2 (d, J = 101.9 Hz) ppm. **^{19}F NMR** (376 MHz, Chloroform-*d*) δ -122.8 ppm. **HRMS (ESI)** Calcd for $C_{16}H_{18}FN_3O_3S$ $[M+Na]^+$: 209.0026; Found: 209.0028.

Picolinaldehyde (35)

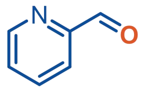

35

Purification by column chromatography (petroleum ether/ethyl acetate = 20:1-1:1) and isolated in 43% yield as a pale yellow oil. **^{1}H NMR** (400 MHz, Chloroform-*d*) δ 10.10 (s, 1H), 8.82 (s, 1H), 7.98 (d, J = 7.8 Hz, 1H), 7.90 (t, J = 7.6 Hz, 1H), 7.66 – 7.42 (m, 1H) ppm. **^{13}C NMR** (101 MHz, Chloroform-*d*) δ 193.3, 152.7, 150.1, 137.0, 127.8, 121.6 ppm. A known compound and the characterization data are in accordance with the literature.[15]

thiophene-2-carbaldehyde (36)

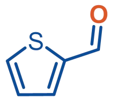

36

Purification by column chromatography (petroleum ether/ethyl acetate = 20:1-5:1) and isolated in 67% yield as a pale yellow oil. **^{1}H NMR** (400 MHz, Chloroform-*d*) δ 9.95 (s, 1H), 7.79 (dd, J = 7.6, 4.1 Hz, 2H), 7.22 (t, J = 3.8 Hz, 1H) ppm. **^{13}C NMR** (101 MHz, Chloroform-*d*) δ 182.9, 143.9, 136.2, 135.0, 128.2 ppm. A known compound and the characterization data are in accordance with the literature.[15]

2-bromothiophene-3-carbaldehyde (37)

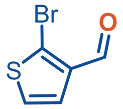

37

Purification by column chromatography (petroleum ether/ethyl acetate = 20:1-5:1) and isolated in 67% yield as a brown solid. **^{1}H NMR** (400 MHz, Chloroform-*d*) δ 9.94 (s, 1H), 7.36 (d, J = 5.8 Hz, 1H), 7.29 (d, J = 5.8 Hz, 1H) ppm. **^{13}C NMR** (101 MHz, Chloroform-*d*) δ 184.6, 138.4, 126.9, 126.2, 125.3 ppm. A known compound and the characterization data are in accordance with the literature.[29]

2-naphthaldehyde (38)

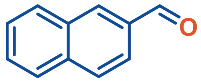

38

Purification by column chromatography (petroleum ether/ethyl acetate = 20:1-7:1) and isolated in 92% yield from 2-(chloromethyl)naphthalene 96% yield from 2-(bromomethyl)naphthalene as a white solid. **^{1}H NMR** (400 MHz, Chloroform-*d*) δ 10.14 (s, 1H), 8.36 – 8.28 (s, 1H), 7.98 (d, *J* = 8.1 Hz, 1H), 7.97 – 7.91 (m, 2H), 7.89 (d, *J* = 8.7 Hz, 1H), 7.67 – 7.60 (m, 1H), 7.60 – 7.54 (m, 1H) ppm. **^{13}C NMR** (151 MHz, Chloroform-*d*) δ 192.1, 136.3, 134.4, 134.0, 132.5, 129.4, 129.0, 129.0, 128.0, 127.0, 122.6 ppm. A known compound and the characterization data are in accordance with the literature.[15]

anthracene-9-carbaldehyde (39)

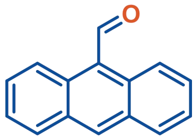

39

Purification by column chromatography (petroleum ether/ethyl acetate = 20:1-5:1) and isolated in 85% yield as a yellow solid. **^{1}H NMR** (400 MHz, Chloroform-*d*) δ 11.48 (s, 1H), 8.95 (d, *J* = 9.0 Hz, 2H), 8.63 (s, 1H), 8.02 (d, *J* = 8.5 Hz, 2H), 7.80 – 7.60 (m, 2H), 7.59 – 7.44 (m, 2H) ppm. **^{13}C NMR** (151 MHz, Chloroform-*d*) δ 192.9, 135.1, 132.0, 131.0, 129.2, 129.0, 125.6, 124.6, 123.4 ppm. A known compound and the characterization data are in accordance with the literature.[15]

4-(chloromethyl)benzaldehyde (40)

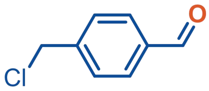

40

Purification by column chromatography (petroleum ether/ethyl acetate = 20:1-5:1) and isolated in 52% yield as a white solid. **^{1}H NMR** (400 MHz, Chloroform-*d*) δ 10.02 (s, 1H), 7.89 (d, *J* = 8.2 Hz, 2H), 7.56 (d, *J* = 8.1 Hz, 2H), 4.64 (s, 2H) ppm. **^{13}C NMR** (151 MHz, Chloroform-*d*) δ 191.5, 143.7, 136.1, 130.0, 129.0, 45.2 ppm. A known compound and the characterization data are in accordance with the literature.[30]

3-(chloromethyl)benzaldehyde (41)

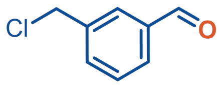

41

Purification by column chromatography (petroleum ether/ethyl acetate = 20:1-5:1) and isolated in 72% yield as a pale yellow oil. **^{1}H NMR** (400 MHz, Chloroform-*d*) δ 10.03 (s, 1H), 7.91 (s, 1H), 7.85 (d, J = 7.6 Hz, 1H), 7.67 (d, J = 7.6 Hz, 1H), 7.55 (t, J = 7.6 Hz, 1H), 4.66 (s, 2H) ppm. 13**C NMR** (101 MHz, Chloroform-*d*) δ 191.8, 138.6, 136.7, 134.4, 129.7, 129.5, 129.4, 77.3, 77.0, 76.7, 45.2 ppm. A known compound and the characterization data are in accordance with the literature.[31]

2-(4-methoxyphenyl)acetaldehyde (42)

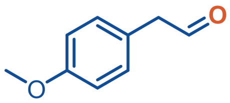

42

Purification by column chromatography (petroleum ether/ethyl acetate = 20:1-5:1) and isolated in 67% yield as a pale yellow oil. **^{1}H NMR** (400 MHz, Chloroform-*d*) δ 9.71 (s, 1H), 7.13 (d, J = 8.6 Hz, 3H), 6.90 (d, J = 8.6 Hz, 3H), 3.80 (s, 7H), 3.62 (d, J = 2.4 Hz, 4H) ppm. **^{13}C NMR** (101 MHz, Chloroform-*d*) δ 199.7, 158.8, 130.6, 123.6, 114.3, 77.3, 77.0, 76.7, 55.2, 49.6 ppm. A known compound and the characterization data are in accordance with the literature.[32]

cinnamaldehyde (43)

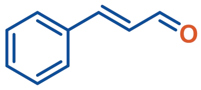

43

Purification by column chromatography (petroleum ether/ethyl acetate = 20:1-7:1) and isolated in 15% yield from (*E*)-(3-chloroprop-1-en-1-yl)benzene and 53% yield from (*E*)-(3-bromoprop-1-en-1-yl)benzene as a pale yellow oil. **^{1}H NMR** (400 MHz, Chloroform-*d*) δ 9.70 (d, *J* = 7.7 Hz, 1H), 7.57 (d, *J* = 2.0 Hz, 1H), 7.55 (d, *J* = 4.3 Hz, 1H), 7.47 (d, *J* = 16.0 Hz, 1H), 7.44 (d, *J* = 2.1 Hz, 2H), 7.42 (d, *J* = 1.8 Hz, 1H), 6.71 (dd, *J* = 15.9, 7.7 Hz, 1H) ppm. **^{13}C NMR** (151 MHz, Chloroform-*d*) δ 193.5, 152.6, 133.9, 131.1, 128.9, 128.4, 128.3 ppm. A known compound and the characterization data are in accordance with the literature.[15]

acetophenone (44)

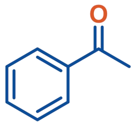

44

Purification by column chromatography (petroleum ether/ethyl acetate = 20:1-10:1) and isolated in 50% yield from (1-chloroethyl)benzene and 62% yield from (1-bromoethyl)benzene as a colorless oil. **^{1}H NMR** (400 MHz, Chloroform-*d*) δ 7.96 (d, *J* = 8.1 Hz, 2H), 7.55 (d, *J* = 7.1 Hz, 1H), 7.46 (t, *J* = 7.6 Hz, 2H), 2.60 (s, 3H) ppm. **^{13}C NMR** (101 MHz, Chloroform-*d*) δ 198.0, 136.9, 132.9, 128.4, 128.1, 26.4 ppm. A known compound and the characterization data are in accordance with the literature.[15]

1-(*p*-tolyl)ethan-1-one (45)

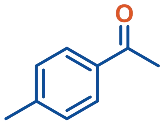

45

Purification by column chromatography (petroleum ether/ethyl acetate = 20:1-10:1) and isolated in 83% yield as a pale yellow oil. **^{1}H NMR** (400 MHz, Chloroform-*d*) δ 7.85 (d, *J* = 8.2 Hz, 2H), 7.25 (d, *J* = 8.0 Hz, 2H), 2.57 (s, 3H), 2.40 (s, 3H) ppm. **^{13}C NMR** (101 MHz, Chloroform-*d*) δ 196.9, 135.7, 131.8, 129.7, 128.2, 26.5 ppm. A known compound and the characterization data are in accordance with the literature.[15]

1-(4-fluorophenyl)ethan-1-one (46)

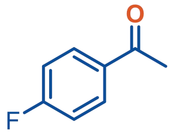

46

Purification by column chromatography (petroleum ether/ethyl acetate = 20:1-7:1) and isolated in 91% yield as a colorless oil. **^{1}H NMR** (400 MHz, Chloroform-*d*) δ 8.36 – 7.59 (m, 2H), 7.43 – 6.76 (m,2H), 2.59 (s, 3H) ppm. **^{13}C NMR** (101 MHz, Chloroform-*d*) δ 196.3, 165.6 (d, *J* = 254.6 Hz), 133.4 (d, *J* = 3.0 Hz), 130.8 (d, *J* = 9.4 Hz), 115.5 (d, *J* = 21.9 Hz), 26.4 ppm. **^{19}F NMR** (376 MHz, Chloroform-d) δ -105.4 ppm. A known compound and the characterization data are in accordance with the literature.[15]

1-(2-fluorophenyl)ethan-1-one (47)

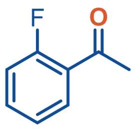

47

Purification by column chromatography (petroleum ether/ethyl acetate = 20:1-7:1) and isolated in 43% yield as a pale yellow oil. **^{1}H NMR** (400 MHz, Chloroform-*d*) δ 7.88 (td, *J* = 7.7, 1.9 Hz, 1H), 7.52 (m, *J* = 8.3, 7.1, 5.0, 1.9 Hz, 1H), 7.22 (m, *J* = 8.3, 7.4, 1.1 Hz, 1H), 7.14 (m, *J* = 11.2, 8.3, 1.1 Hz, 1H), 2.65 (d, *J* = 4.9 Hz, 3H) ppm. **^{13}C NMR** (101 MHz, Chloroform-*d*) δ 195.8 (d, *J* = 3.4 Hz), 162.1 (d, *J* = 254.9 Hz), 134.6 (d, *J* = 9.1 Hz), 130.5 (d, *J* = 2.5 Hz), 125.6 (d, *J* = 12.8 Hz), 124.2 (d, *J* = 3.3 Hz), 116.5 (d, *J* = 23.8 Hz), 31.3 (d, *J* = 7.3 Hz) ppm. **^{19}F NMR** (376 MHz, Chloroform-d) δ -109.5 ppm. A known compound and the characterization data are in accordance with the literature.[33]

1-(4-chlorophenyl)ethan-1-one (48)

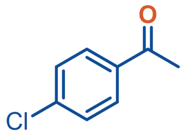

48

Purification by column chromatography (petroleum ether/ethyl acetate = 20:1-7:1) and isolated in 49% yield as a pale yellow oil. **^{1}H NMR** (400 MHz, Chloroform-*d*) δ 7.89 (d, *J* = 8.7 Hz, 2H), 7.43 (d, *J* = 8.7 Hz, 2H), 2.59 (s, 3H) ppm. **^{13}C NMR** (101 MHz, Chloroform-*d*) δ 196.7, 139.4, 135.3, 129.6, 128.8, 26.5 ppm. A known compound and the characterization data are in accordance with the literature.[15]

1-(4-bromophenyl)ethan-1-one (49)

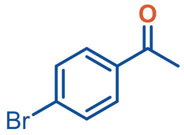

49

Purification by column chromatography (petroleum ether/ethyl acetate = 20:1-7:1) and isolated in 35% yield as a white solid. **^{1}H NMR** (400 MHz, Chloroform-*d*) δ 7.81 (d, *J* = 8.7 Hz, 2H), 7.59 (d, *J* = 8.7 Hz, 2H), 2.58 (s, 3H) ppm. **^{13}C NMR** (101 MHz, Chloroform-*d*) δ 135.7, 131.8, 129.7, 128.2, 26.5 ppm. A known compound and the characterization data are in accordance with the literature.[15]

Propiophenone (50)

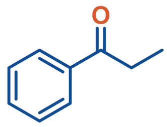

50

Purification by column chromatography (petroleum ether/ethyl acetate = 20:1-10:1) and isolated in 34% yield from (1-chloropropyl)benzene and 22% yield from (1-bromopropyl)benzene as a colorless oil. **^{1}H NMR** (400 MHz, Chloroform-*d*) δ 7.96 (d, *J* = 8.5 Hz, 2H), 7.55 (t, *J* = 7.4 Hz, 1H), 7.45 (t, *J* = 7.5 Hz, 2H), 3.01 (q, *J* = 7.2 Hz, 2H), 1.23 (t, *J* = 7.2 Hz, 3H) ppm. **^{13}C NMR** (101 MHz, Chloroform-*d*) δ 200.7, 136.8, 132.8, 128.4, 127.9, 31.7, 8.1 ppm. A known compound and the characterization data are in accordance with the literature.[34]

1H-indene-1,3(2*H*)-dione (51)

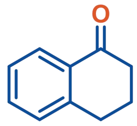

51

Purification by column chromatography (petroleum ether/ethyl acetate = 20:1-7:1) and isolated in 89% yield as a pale yellow solid. **^{1}H NMR** (400 MHz, Chloroform-*d*) δ 8.03 (d, *J* = 7.8 Hz, 1H), 7.52 – 7.42 (m, 1H), 7.30 (t, *J* = 7.6 Hz, 1H), 7.27 – 7.21 (m, 1H), 2.96 (t, *J* = 6.1 Hz, 2H), 2.65 (dd, *J* = 7.3, 5.8 Hz, 2H), 2.13 (t, *J* = 6.4 Hz, 2H) ppm. **^{13}C NMR** (101 MHz, Chloroform-*d*) δ 198.2, 144.3, 133.2, 132.5, 128.6, 127.0, 126.5, 39.0, 29.6, 23.2 ppm. A known compound and the characterization data are in accordance with the literature.[15]

3,4-dihydronaphthalen-1(2*H*)-one (52)

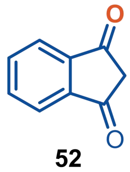

52

Purification by column chromatography (petroleum ether/ethyl acetate = 20:1-3:1) and isolated in 62% yield as a pale yellow solid. **^{1}H NMR** (400 MHz, Chloroform-*d*) δ 7.99 (dd, *J* = 5.7, 3.1 Hz, 2H), 7.86 (dd, *J* = 5.7, 3.1 Hz, 2H), 3.26 (s, 2H) ppm. **^{13}C NMR** (101 MHz, Chloroform-*d*) δ 197.4, 143.3, 135.5, 123.1, 44.9 ppm. A known compound and the characterization data are in accordance with the literature.[35]

9*H*-fluoren-9-one (53)

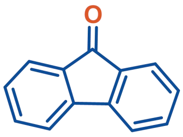

53

Purification by column chromatography (petroleum ether/ethyl acetate = 20:1-7:1) and isolated in 97% yield as a yellow solid. **^{1}H NMR** (400 MHz, Chloroform-*d*) δ 7.67 – 7.54 (m, 2H), 7.44 (m, *J* = 6.5, 1.3 Hz, 4H), 7.28 – 7.20 (m, 2H) ppm. **^{13}C NMR** (151 MHz, Chloroform-*d*) δ 193.7, 144.2, 134.5, 134.0, 128.9, 124.1, 120.1 ppm. A known compound and the characterization data are in accordance with the literature.[13]

benzophenone (54)

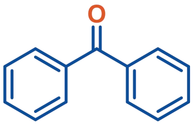

54

Purification by column chromatography (petroleum ether/ethyl acetate = 20:1-7:1) and isolated in 96% yield from (chloromethylene)dibenzene and 99% yield from (bromomethylene)dibenzene as a white solid. **^{1}H NMR** (400 MHz, Chloroform-*d*) δ 7.80 (d, *J* = 8.2 Hz, 4H), 7.58 (t, *J* = 8.0 Hz, 2H), 7.47 (t, *J* = 7.6 Hz,4H) ppm. **^{13}C NMR** (151 MHz, Chloroform-*d*) δ 196.66, 137.53, 132.35, 129.98, 128.21 ppm. A known compound and the characterization data are in accordance with the literature.[15]

(4-chlorophenyl)(phenyl)methanone (55)

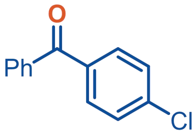

55

Purification by column chromatography (petroleum ether/ethyl acetate = 20:1-5:1) and isolated in 63% yield as a white solid. **^{1}H NMR** (400 MHz, Chloroform-*d*) δ 7.80 – 7.71 (m, 4H), 7.65 – 7.57 (m, 1H), 7.53 – 7.40 (m, 4H) ppm. **^{13}C NMR** (101 MHz, Chloroform-d) δ 195.4, 138.8, 137.1, 135.7, 132.5, 131.4, 129.8, 128.5, 128.3 ppm. A known compound and the characterization data are in accordance with the literature.[36]

bis(4-fluorophenyl)methanone (56)

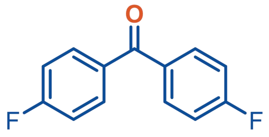

56

Purification by column chromatography (petroleum ether/ethyl acetate = 20:1-5:1) and isolated in 93% yield as a white solid. **^{1}H NMR** (400 MHz, Chloroform-*d*) δ 7.87 – 7.78 (m, 4H), 7.21 – 7.11 (m, 4H) ppm. **^{13}C NMR** (151 MHz, Chloroform-*d*) δ 193.7, 165.3 (d, *J* = 254.4 Hz), 133.6 (d, *J* = 3.0 Hz), 132.4 (d, *J* = 9.2 Hz), 115.5 (d, *J* = 21.9 Hz) ppm. **^{19}F NMR** (376 MHz, Chloroform-*d*) δ -105.7 ppm. A known compound and the characterization data are in accordance with the literature.[36]

benzil (57)

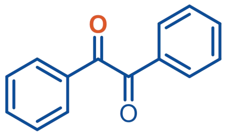

57

Purification by column chromatography (petroleum ether/ethyl acetate = 20:1-5:1) and isolated in 62% yield as a white solid. **^{1}H NMR** (400 MHz, Chloroform-*d*) δ 7.97 (dd, *J* = 8.4, 1.3 Hz, 4H), 7.65 (t, *J* = 7.4 Hz, 2H), 7.51 (t, *J* = 7.7 Hz, 4H) ppm. **^{13}C NMR** (151 MHz, Chloroform-*d*) δ 186.3, 163.7, 134.8, 132.4, 129.9, 128.8, 62.2, 14.0 ppm. A known compound and the characterization data are in accordance with the literature.[36]

ethyl 2-oxo-2-phenylacetate (58)

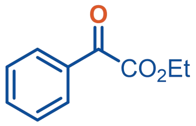

58

Purification by column chromatography (petroleum ether/ethyl acetate = 20:1-5:1) and isolated in 25% yield as a pale yellow oil. **^{1}H NMR** (400 MHz, Chloroform-*d*) δ 8.01 (d, *J* = 8.2 Hz, 2H), 7.66 (t, *J* = 7.4 Hz, 1H), 7.51 (t, *J* = 7.8 Hz, 2H), 4.46 (d, *J* = 7.1 Hz, 2H), 1.42 (t, *J* = 7.2 Hz, 3H) ppm. **^{13}C NMR** (151 MHz, Chloroform-*d*) δ 186.3, 163.7, 134.8, 132.4, 129.9, 128.8, 62.2, 14.0 ppm. A known compound and the characterization data are in accordance with the literature.[36]

4-(2-chloroethyl)benzaldehyde (59)

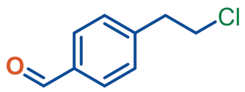

59

Purification by column chromatography (petroleum ether/ethyl acetate = 20:1-7:1) and isolated in 74% yield as a pale yellow oil. **^{1}H NMR** (400 MHz, Chloroform-*d*) δ 10.00 (s, 1H), 7.85 (d, J = 8.2 Hz, 2H), 7.40 (d, J = 8.1 Hz, 2H), 3.77 (t, J = 7.1 Hz, 2H), 3.16 (t, J = 7.1 Hz, 2H) ppm. **^{13}C NMR** (101 MHz, Chloroform-*d*) δ 191.8, 145.1, 135.1, 130.0, 129.5, 44.2, 38.9 ppm. A known compound and the characterization data are in accordance with the literature.[37]

4-(2-hydroxyethyl)benzaldehyde (60)

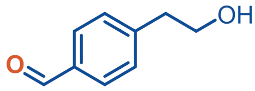

60

Purification by column chromatography (petroleum ether/ethyl acetate = 20:1-7:1) and isolated in 86% yield as a pale yellow oil. **^{1}H NMR** (400 MHz, Chloroform-*d*) δ 10.00 (s, 1H), 7.84 (d, *J* = 8.1 Hz, 2H), 7.42 (d, *J* = 8.1 Hz, 2H), 3.93 (t, *J* = 6.5 Hz, 2H), 2.97 (t, *J* = 6.5 Hz, 2H), 2.01 (s, 1H) ppm. **^{13}C NMR** (101 MHz, Chloroform-d) δ 191.9, 146.1, 134.9, 130.0, 129.7, 63.1, 39.3 ppm. A known compound and the characterization data are in accordance with the literature.[38]

3-hydroxybenzaldehyde (61)

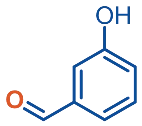

61

Purification by column chromatography (petroleum ether/ethyl acetate = 20:1-3:1) and isolated in 49% yield as a white solid. **^{1}H NMR** (400 MHz, Chloroform-*d*) δ 9.96 (s, 1H), 7.49 – 7.41 (m, 2H), 7.39 (d, *J* = 2.0 Hz, 1H), 7.16 (dt, *J* = 7.3, 2.5 Hz, 1H), 5.78 (s, 1H) ppm. **^{13}C NMR** (101 MHz, Chloroform-*d*) δ 192.4, 156.3, 137.7, 130.3, 123.4, 122.0, 114.7 ppm. A known compound and the characterization data are in accordance with the literature.[39]

2-chlorothiazole-5-carbaldehyde (62)

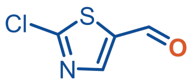

62

Purification by column chromatography (petroleum ether/ethyl acetate = 20:1-5:1) and isolated in 67% yield as a white solid. **^{1}H NMR** (400 MHz, Chloroform-*d*) δ 9.88 (s, 1H), 8.12 (s, 1H) ppm. **^{13}C NMR** (101 MHz, Chloroform-*d*) δ 180.2, 159.3, 148.5, 140.2 ppm. A known compound and the characterization data are in accordance with the literature.[40]

7-methoxy-2-oxo-2*H*-chromene-3-carbaldehyde (63)

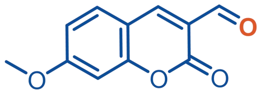

63

Purification by column chromatography (petroleum ether/ethyl acetate = 20:1-1:1) and isolated in 32% yield as a pale yellow oil. **^{1}H NMR** (400 MHz, Chloroform-*d*) δ 10.08 (s, 1H), 8.50 (d, *J* = 9.0 Hz, 1H), 6.93 (dd, *J* = 9.0, 2.5 Hz, 1H), 6.87 (d, *J* = 2.5 Hz, 1H), 6.72 (s, 1H), 3.90 (s, 3H) ppm. **^{13}C NMR** (101 MHz, Chloroform-*d*) δ 191.7, 163.3, 160.7, 156.4, 143.7, 127.3, 122.1, 113.2, 108.1, 101.0, 55.8 ppm. A known compound and the characterization data are in accordance with the literature.[41]

***N*-(4-(4-fluorophenyl)-5-formyl-6-isopropylpyrimidin-2-yl)-*N*-methylmethanesulfonamide (64)**

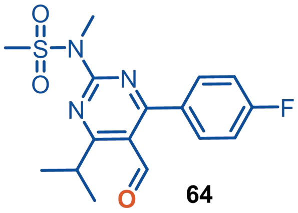

64

Purification by column chromatography (petroleum ether/ethyl acetate = 20:1-1:1) and isolated in 23% yield as a white solid. **^{1}H NMR** (400 MHz, Chloroform-*d*) δ 9.97 (s, 1H), 7.63 (dd, J = 8.6, 5.3 Hz, 2H), 7.23 (t, J = 8.6 Hz, 2H), 4.01 (p, J = 6.7 Hz, 1H), 3.64 (s, 3H), 3.56 (s, 3H), 1.32 (d, J = 6.7 Hz, 7H) ppm. **^{13}C NMR** (101 MHz, Chloroform-*d*) δ 190.5, 179.0, 169.8, 164.43 (d, *J* = 252.6 Hz), 158.8, 132.6 (d, *J* = 8.9 Hz), 132.1 (d, *J* = 3.2 Hz), 119.5, 115.9 (d, *J* = 22.0 Hz), 42.5, 33.1, 32.0, 21.7 ppm. **^{19}F NMR** (376 MHz, Chloroform-d) δ -108.6 ppm. **HRMS (ESI)** Calcd for $C_{16}H_{18}FN_3O_3S$ $[M+H]^+$: 352.1126; Found: 352.1124.

methyl 2-(4-formylbenzamido)-3-hydroxybutanoate (65)

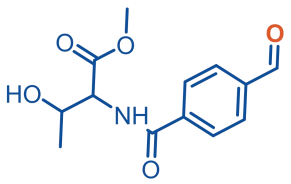

65

Purification by column chromatography (petroleum ether/ethyl acetate = 5:1-1:2) and isolated in 60% yield as a pale yellow oil. **^{1}H NMR** (400 MHz, Chloroform-*d*) δ 10.08 (d, *J* = 1.7 Hz, 1H), 8.15 – 7.85 (m, 3H), 7.12 (d, *J* = 8.8 Hz, 1H), 4.86 (ddd, *J* = 17.1, 8.1, 2.9 Hz, 1H), 4.40 (ddd, *J* = 74.1, 6.4, 2.9 Hz, 1H), 3.82 (d, *J* = 13.1 Hz, 3H), 1.30 (dd, *J* = 11.7, 6.5 Hz, 3H) ppm. **^{13}C NMR** (101 MHz, Chloroform-*d*) δ 191.4 (d, *J* = 7.0 Hz), 170.9 (d, *J* = 80.5 Hz), 166.8 (d, *J* = 6.2 Hz), 138.6 (d, *J* = 30.8 Hz), 138.3 (d, *J* = 1.8 Hz), 129.8 (d, *J* = 2.5 Hz), 127.9, 68.5 (d, *J* = 94.9 Hz), 58.2 (d, *J* = 91.9 Hz), 52.7 (d, *J* = 6.9 Hz), 19.5 (d, *J* = 111.6 Hz) ppm. **HRMS (ESI)** Calcd for $C_{13}H_{15}NO_5$ $[M+Na]^+$: 288.0842; Found: 288.0845.

(1*S*,2*R*,5*S*)-2-isopropyl-5-methylcyclohexyl 4-formylbenzoate (66)

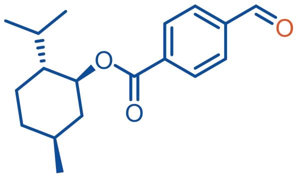

66

Purification by column chromatography (petroleum ether/ethyl acetate = 10:1-5:1) and isolated in 63% yield as a white oil. **^{1}H NMR** (400 MHz, Chloroform-*d*) δ 10.11 (s, 1H), 8.20 (d, J = 8.3 Hz, 2H), 7.96 (d, J = 8.3 Hz, 2H), 5.91 – 4.90 (m, 1H), 2.17 – 2.10 (m, 1H), 1.95 (td, J = 7.0, 2.7 Hz, 1H), 1.78 – 1.70 (m, 3H), 1.62 – 1.53 (m, 1H), 1.19 – 1.10 (m, 2H), 0.94 (dd, J = 6.8, 4.6 Hz, 7H), 0.80 (d, J = 6.9 Hz, 3H) ppm. **^{13}C NMR** (101 MHz, Chloroform-*d*) δ 191.7, 165.0, 139.0, 135.8, 130.1, 129.5, 75.6, 47.2, 40.8, 34.2, 31.4, 26.5, 23.5, 22.0, 20.7, 16.4 ppm. **HRMS (ESI)** Calcd for $C_{18}H_{24}O_3$ $[M+K]^+$: 327.1357; Found: 327.1318.

((3a*S*,5a*R*,8a*R*,8b*S*)-2,2,7,7-tetramethyltetrahydro-3a*H*-bis([1,3]dioxolo)[4,5-*b*:4',5'-*d*]pyran-3a-yl)methyl 4-formylbenzoate (67)

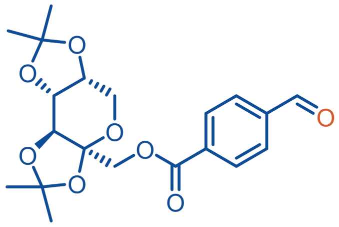

67

Purification by column chromatography (petroleum ether/ethyl acetate = 5:1-1:1) and isolated in 27% yield as a pale yellow oil. **^{1}H NMR** (400 MHz, Chloroform-*d*) δ 10.11 (s, 1H), 8.24 (d, *J* = 8.2 Hz, 2H), 7.96 (d, *J* = 8.3 Hz, 2H), 4.73 (d, *J* = 11.8 Hz, 1H), 4.65 (dd, *J* = 7.9, 2.6 Hz, 1H), 4.45 (d, *J* = 2.6 Hz, 1H), 4.36 (d, *J* = 11.8 Hz, 1H), 4.27 (d, *J* = 8.9 Hz, 1H), 3.96 (dd, *J* = 13.0, 1.9 Hz, 1H), 3.82 (d, *J* = 13.0 Hz, 1H), 1.51 (d, *J* = 39.1 Hz, 6H), 1.36 (d, *J* = 4.2 Hz, 6H) ppm. **^{13}C NMR** (101 MHz, Chloroform-*d*) δ 191.5, 164.9, 139.2, 134.8, 130.3, 129.5, 109.1, 108.9, 101.5, 77.2, 70.7, 70.6, 70.0, 65.9, 61.4, 26.5, 25.8, 25.4, 24.0 ppm. **HRMS (ESI)** Calcd for $C_{20}H_{24}O_8$ [M+H]$^+$: 393.1544; Found: 393.1542.

(3a*R*,5*R*,6*R*,6a*S*)-5-(((4-formylbenzoyl)oxy)methyl)-2-oxohexahydro-2*H*-cyclopenta[*b*]furan-6-yl [1,1'-biphenyl]-4-carboxylate (68)

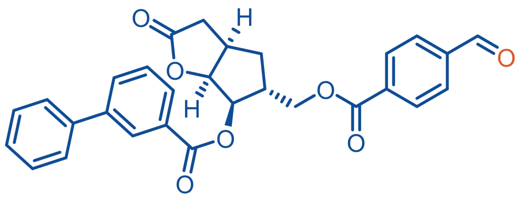

68

Purification by column chromatography (petroleum ether/ethyl acetate = 5:1-1:1) and isolated in 33% yield as a yellow oli. **^{1}H NMR** (400 MHz, Chloroform-*d*) δ 10.08 (s, 1H), 8.18 (d, J = 8.3 Hz, 2H), 8.05 (d, J = 8.4 Hz, 2H), 7.94 (d, J = 8.3 Hz, 2H), 7.65 (d, J = 8.4 Hz, 2H), 7.61 (d, J = 7.3 Hz, 2H), 7.47 (t, J = 7.4 Hz, 2H), 7.40 (t, J = 7.3 Hz, 1H), 5.55 – 5.49 (m, 1H), 5.18 – 5.13 (m, 1H), 4.52 – 4.36 (m, 2H), 3.00 (dd, J = 17.9, 10.2 Hz, 1H), 2.95 – 2.85 (m, 1H), 2.75 – 2.56 (m, 3H), 2.44 (dd, J = 15.9, 4.1 Hz, 1H) ppm. **^{13}C NMR** (101 MHz, Chloroform-*d*) δ 191.5, 176.1, 165.7, 165.3, 146.1, 139.7, 139.3, 134.3, 130.18, 130.17, 129.6, 128.9, 128.2, 127.9, 127.2, 127.1, 83.8, 77.1 64.9, 51.5, 40.5, 38.1, 35.7 ppm. **HRMS (ESI)** Calcd for $C_{29}H_{24}O_7$ [M+Na]$^+$: 507.1414; Found: 507.1408.

***tert*-butyl*N*6-(((9*H*-fluoren-9-yl)methoxy)carbonyl)-*N*2-(4-formylbenzoyl)lysinate (69)**

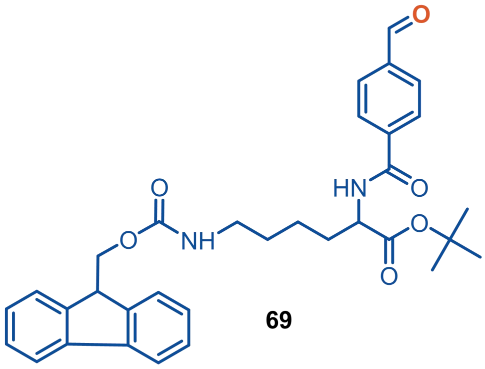

69

Purification by column chromatography (petroleum ether/ethyl acetate = 5:1-1:2) and isolated in 31% yield as a colorless oil. **^{1}H NMR** (400 MHz, Chloroform-*d*) δ 9.88 (s, 1H), 7.87 (d, *J* = 7.9 Hz, 2H), 7.76 (d, *J* = 7.9 Hz, 2H), 7.66 (d, *J* = 7.5 Hz, 2H), 7.48 – 7.39 (m, 2H), 7.30 (t, *J* = 7.4 Hz, 2H), 7.24 – 7.15 (m, 2H), 6.94 (d, *J* = 7.1 Hz, 1H), 4.98 – 4.85 (m, 1H), 4.64 – 4.53 (m, 1H), 4.30 – 4.14 (m, 2H), 4.06 (t, *J* = 6.9 Hz, 1H), 3.20 – 3.05 (m, 2H), 1.94 – 1.83 (m, 1H), 1.81 – 1.69 (m, 1H), 1.58 – 1.46 (m, 3H), 1.41 (s, 9H) ppm. **^{13}C NMR** (101 MHz, Chloroform-*d*) δ 191.5, 171.5, 165.9, 156.6, 143.8, 141.2, 139.0, 138.2, 129.7, 127.8, 127.6, 126.9, 124.9, 119.9, 82.5, 66.6, 53.1, 47.1, 40.2, 31.9, 29.5, 27.9, 22.1 ppm. **HRMS (ESI)** Calcd for $C_{33}H_{36}N_2O_6$ $[M+H]^+$: 557.2646; Found: 557.2644.

4-(3,3-diphenylallyl)benzonitrile (72)

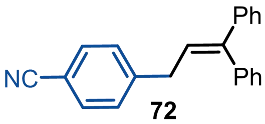

72

Purification by column chromatography (petroleum ether) and isolated in 73% yield as a pale yellow oil. **^{1}H NMR** (400 MHz, Chloroform-*d*) δ 7.58 (d, *J* = 8.3 Hz, 2H), 7.45 (d, *J* = 7.1 Hz, 1H), 7.41 (d, *J* = 6.9 Hz, 1H), 7.37 (d, *J* = 7.6 Hz, 1H), 7.34 (d, *J* = 7.6 Hz, 1H), 7.29 (d, *J* = 7.8 Hz, 3H), 7.25 (d, *J* = 4.5 Hz, 3H), 7.20 (d, *J* = 6.7 Hz, 2H), 6.20 (t, *J* = 7.6 Hz, 1H), 3.52 (d, *J* = 7.6 Hz, 2H) ppm. **^{13}C NMR** (101 MHz, Chloroform-*d*) δ 146.6, 143.9, 141.8, 139.3, 132.3, 129.6, 129.1, 128.4, 128.3, 128.1, 127.4, 127.2, 126.2, 125.4, 109.8, 35.9 ppm. A known compound and the characterization data are in accordance with the literature.[42]

(*E*)-(4-chlorobut-1-en-1-yl)benzene (73)

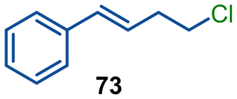

73

Purification by column chromatography (petroleum ether) and isolated in 72% yield as a colorless oil. **^{1}H NMR** (400 MHz, Chloroform-*d*) δ 7.36 (d, *J* = 7.2 Hz, 2H), 7.31 (t, *J* = 7.5 Hz, 2H), 7.26 – 7.16 (m, 1H), 6.49 (d, *J* = 15.9 Hz, 1H), 6.21 (dt, *J* = 15.8, 7.0 Hz, 1H), 3.62 (t, *J* = 6.9 Hz, 2H), 2.68 (qd, *J* = 6.9, 1.2 Hz, 2H) ppm. **^{13}C NMR** (101 MHz, Chloroform-*d*) δ 137.0, 132.7, 127.4, 126.1, 125.7, 44.0, 36.1 ppm. A known compound and the characterization data are in accordance with the literature.[43]

8. References

[1] Luo, J. & Zhang, J. Donor–acceptor fluorophores for visible-light-promoted organic synthesis: photoredox/Ni dual catalytic C(sp^3)–C(sp^2) cross-coupling. *ACS Catal*. **6**, 873–877 (2016).

[2] Xu, J. et al. Unveiling extreme photoreduction potentials of donor–acceptor cyanoarenes to access aryl radicals from aryl chlorides. *J. Am. Chem. Soc*. **143**, 13266–13273 (2021).

[3] Salfeena, C. F. et al. Synthesis of symmetrical and unsymmetrical triarylpyrylium ions via an inverse electron demand Diels–Alder reaction. *Chem. Commun*. **54**, 12463–12466 (2018).

[4] Kim, D. & Lim, H. N. Synthesis of acyl fluorides via DAST-mediated fluorinative C–C bond cleavage of activated ketones. *Org. Lett*. **22**, 7465–7469 (2020).

[5] Podhajsky, S. M. & Sigman, M. S. Coupling Pd-catalyzed alcohol oxidation to olefin functionalization: hydrohalogenation/hydroalkoxylation of styrenes. *Organometallics*. **26**, 5680–5686 (2007).

[6] Dong, X. Y. et al. A general asymmetric copper-catalysed Sonogashira C(sp^3)–C(sp) coupling. *Nature Chem*. **11**, 1158–1166 (2019).

[7] Cao, L. et al. One-Pot Synthesis of Quinazolin-4(3H)-ones through anodic oxidation and the related mechanistic studies. *Adv. Synth. Catal.* **360**, 4764–4773 (2018).

[8] Chen, C., Zeng, R., Zhang, J. & Zhao, Y. Ruthenium-catalyzed difluoroalkylation of 8-aminoquinoline amides at the C5-position. *Eur. J. Org. Chem*, **46**, 6947–6950 (2017).

[9] Qi, J. et al. Electrophotochemical synthesis facilitated trifluoromethylation of arenes using trifluoroacetic acid. *J. Am. Chem. Soc*. **145**, 24965–24971 (2023).

[10] Mupparapa, N. et al. Metal-free oxidative amidation of 2-oxoaldehydes: A facile access to *α*-ketoamides. *Org. Lett*. **16**, 1152–1155 (2014).

[11] Kornblum, N., Jones, W. J. & Anderson, G. J. A new and selective method of oxidation. The conversion of alkyl halides and alkyl tosylates to aldehydes. *J. Am. Chem. Soc.* **81**, 4113–4114 (1959).

[12] Cismesia, M. A. & Yoon, T. P. Characterizing chain processes in visible light photoredox catalysis. *Chem. Sci.* **6**, 5426–5434 (2015).

[13] Stone, I. B., Jermaks, J., MacMillan, S. N. & Lambert, T. H. The Hydrazine–O_2 redox couple as a platform for organocatalytic oxidation:

benzo[c]cinnoline-catalyzed oxidation of alkyl halides to aldehydes. *Angew. Chem. Int. Ed.* **57**, 12494–12498 (2018).

[14] Hong, B., Aganda, K. C. C. & Lee, A. Oxidative C–S bond cleavage of benzyl thiols enabled by visible-light-mediated silver (II) complexes. *Org. Lett.* **22**, 4395–4399 (2020).

[15] Yang, X. N. et al. Photochemical hydrogen atom transfer catalysis for dehydrogenation of alcohols to form carbonyls. *Org. Lett.* **25**, 5486–5491 (2023).

[16] Colleville, A. P., Horan, R. A. & Tomkinson, N. C. Aryldiazonium tetrafluoroborate salts as green and efficient coupling partners for the Suzuki–Miyaura reaction: from optimisation to mole scale. *Org. Lett.* **18**, 1128–1136 (2014).

[17] Liu, Z. et al. Rhodium-catalyzed reductive carbonylation of aryl iodides to arylaldehydes with syngas. *Beilstein J. Org. Chem.* **16**, 645–656 (2020).

[18] Pan, S., Yan, S., Osako, T. & Uozumi, Y. Controlled aerobic oxidation of primary benzylic alcohols to aldehydes catalyzed by polymer-supported triazine-based dendrimer–copper composites. *Synlett*. **29**, 1152–1156 (2018).

[19] Bazyar, Z. & Hosseini-Sarvari, M. On/Off O_2 switchable photocatalytic oxidative and protodecarboxylation of carboxylic acids. *J. Org. Chem.* **84**, 13503–13515 (2019).

[20] Zhao, B., Ren, Y.-L., Ren, F., Tian, X. & Zhao, S. Metal-free HNO_3/TEMPO-catalyzed conversion of benzyl alcohols to aromatic nitriles with oxygen molecule as the terminal oxidant. *Lett. Org. Chem.* **15**, 627–632 (2018).

[21] Kawajiri, T. et al. Chemoselective nucleophilic functionalizations of aromatic aldehydes and acetals via pyridinium salt intermediates. *J. Org. Chem.* **84**, 3853–3870 (2019).

[22] Mouselmani, R., et al. Reduction of aromatic nitriles into aldehydes using calcium hypophosphite and a nickel precursor. *Org. Biomol. Chem.* **16**, 6600–6605 (2018).

[23] Mao, Y. et al. Pd-catalyzed debenzylation and deallylation of ethers and esters with sodium hydride. *ACS Catal.* **8**, 3016–3020 (2018).

[24] Cousin, D., Mann, J., Nieuwenhuyzen, M. & van den Berg, H. A new approach to combretastatin D2. *Org. Biomol. Chem.* **4**, 54–62 (2006).

[25] Liu, Z. et al. Rhodium-catalyzed reductive carbonylation of aryl iodides to arylaldehydes with syngas. *J. Org. Chem.* **16**, 645–656 (2020).

[26] Rahimi, A., Azarpira, A., Kim, H., Ralph, J. & Stahl, S. S. Chemoselective metal-free aerobic alcohol oxidation in lignin. *J. Am. Chem. Soc.* **135**, 6415–6418 (2013).

[27] Qing, Q. et al. Merging photoredox with Brønsted acid catalysis: the cross-dehydrogenative C–O coupling for sp^3 C–H bond peroxidation. *Chem. Eur. J.* **23**, 10871–10877 (2017).

[28] Warashina, T., Matsuura, D. & Kimura, Y. Direct formylation of fluorine-containing aromatics with dichloromethyl alkyl ethers. *Chem. Pharm. Bull.* **67**, 587–593 (2019)..

[29] Nitti, A., Bianchi, G., Po, R., Swager, T. M. & Pasini, D. Domino direct arylation and cross-aldol for rapid construction of extended polycyclic π-scaffolds. *J. Am. Chem. Soc.* **139**, 8788–8791 (2017)..

[30] Fiammengo, R., Musílek, K. & Jäschke, A. Efficient Preparation of Organic Substrate− RNA Conjugates via in Vitro Transcription. *J. Am. Chem. Soc.* **127**, 9271–9276 (2005).

[31] Dai, L. & Ye, S. NHC-catalyzed ε-umpolung via p-quinodimethanes and its nucleophilic addition to ketones. *ACS Catal.* **10**, 994–998 (2019).

[32] Li, H. et al. Selective reduction of esters to aldehydes under the catalysis of well-defined NHC-iron complexes. *Angew. Chem. Int. Ed.* **52**, 8045–8049 (2013).

[33] Yousuf, M., Das, T. & Adhikari, S. Palladium catalyzed decarboxylative acylation of arylboronic acid with ethyl cyanoacetate as a new acylating agent: Synthesis of alkyl aryl ketones. *New J. Chem.* **39**, 8763–8770 (2015).

[34] Guðmundsson, A., Schlipkoeter, K. E. & Bäckvall, J. E. Iron (II)-catalyzed biomimetic aerobic oxidation of alcohols. *Angew. Chem. Int. Ed.* **59**, 5403–5406 (2020).

[35] Roscini, C., Davies, D. M., Berry, M., Orr-Ewing, A. J. & Booker-Milburn, K. I. Product selection through photon flux: laser-specific lactone synthesis. *Angew. Chem. Int. Ed.* **47**, 2283–2286 (2008).

[36] Su, Y., Zhang, L. & Jiao, N. Utilization of natural sunlight and air in the aerobic oxidation of benzyl halides. *Org. Lett.* **13**, 2168–2171 (2011)..

[37] Yang, Y. et al. Selective activation of 1, 2-dichloroethane for access to *β*-chloroethylarenes enabled by nickel-catalyzed Suzuki-type couplings. *Tetrahedron Lett.* **60**, 1130–1134 (2019).

[38] Hoover, J. M. & Stahl, S. S. Highly practical copper (I)/TEMPO catalyst system for chemoselective aerobic oxidation of primary alcohols. *J. Am. Chem. Soc.* **133**, 16901–16910 (2011).

[39] Johnson, J. A. et al. Porphyrin-metalation-mediated tuning of photoredox catalytic properties in metal–organic frameworks. *ACS Catal*. **5**, 5283–5291 (2015).

[40] Kunitski, M. et al. Double-slit photoelectron interference in strong-field ionization of the neon dimer *Nat. Commun.* **10**, 1–9 (2019).

[41] Bochkov, A. Y., Akchurin, I. O. & Traven, V. F. A new facile way for the preparation of 3-formylcoumarins. *Heterocycl. Commun.* **23**, 75–78 (2017).

[42] Yu, H., Hu, B. & Huang, H. Nickel-catalyzed benzylation of aryl alkenes with benzylamines via C–N bond activation. *J. Org. Chem.* **83**, 13922–13929 (2018).

[43] Abbott, F. S. & Haya, K. Synthesis of substituted thietanes and thiolanes from α-and *β*-chloroepoxides and their oxidation to 1, 1-dioxides. *Can. J. Chem.* **56**, 71–79 (1978).

9. ^{1}H and ^{13}C NMR Spectra of the products

^{1}H NMR spectrum of **2**

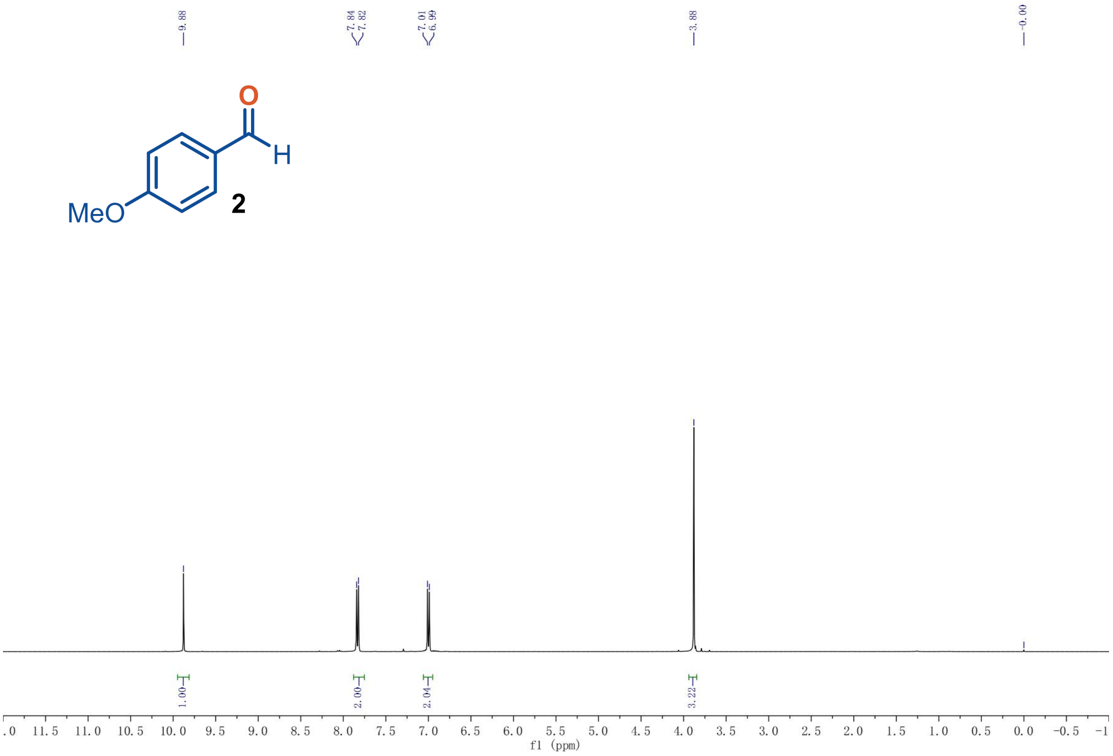

^{13}C NMR spectrum of **2**

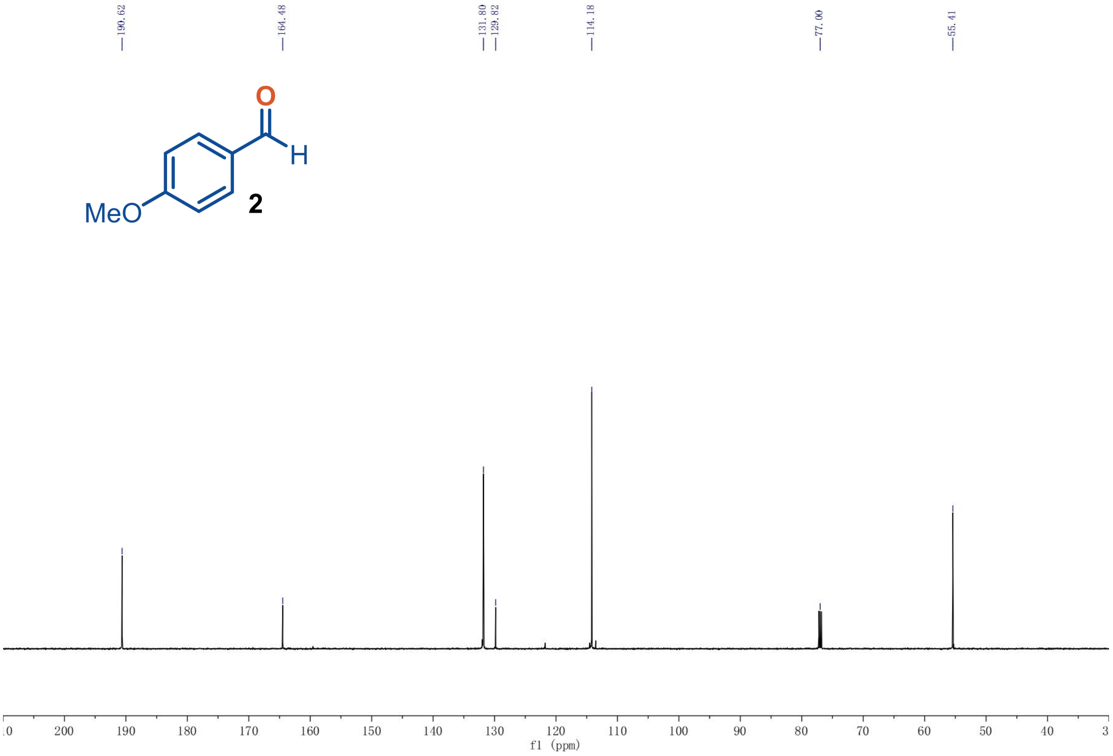

^{1}H NMR spectrum of **4**

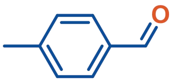

4

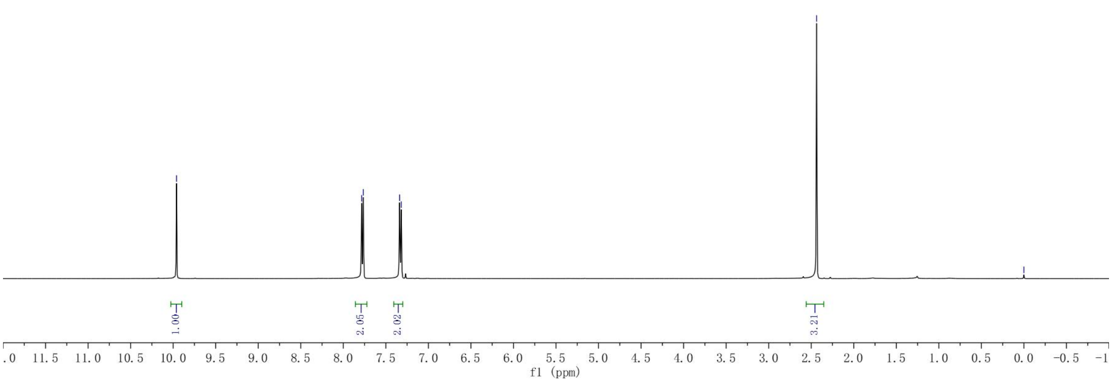

^{13}C NMR spectrum of **4**

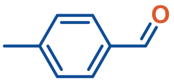

4

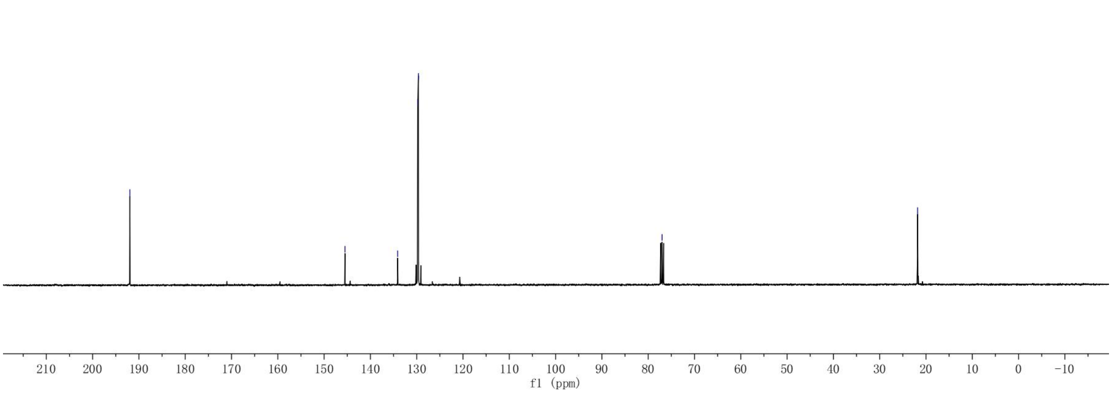

^{1}H NMR spectrum of **5**

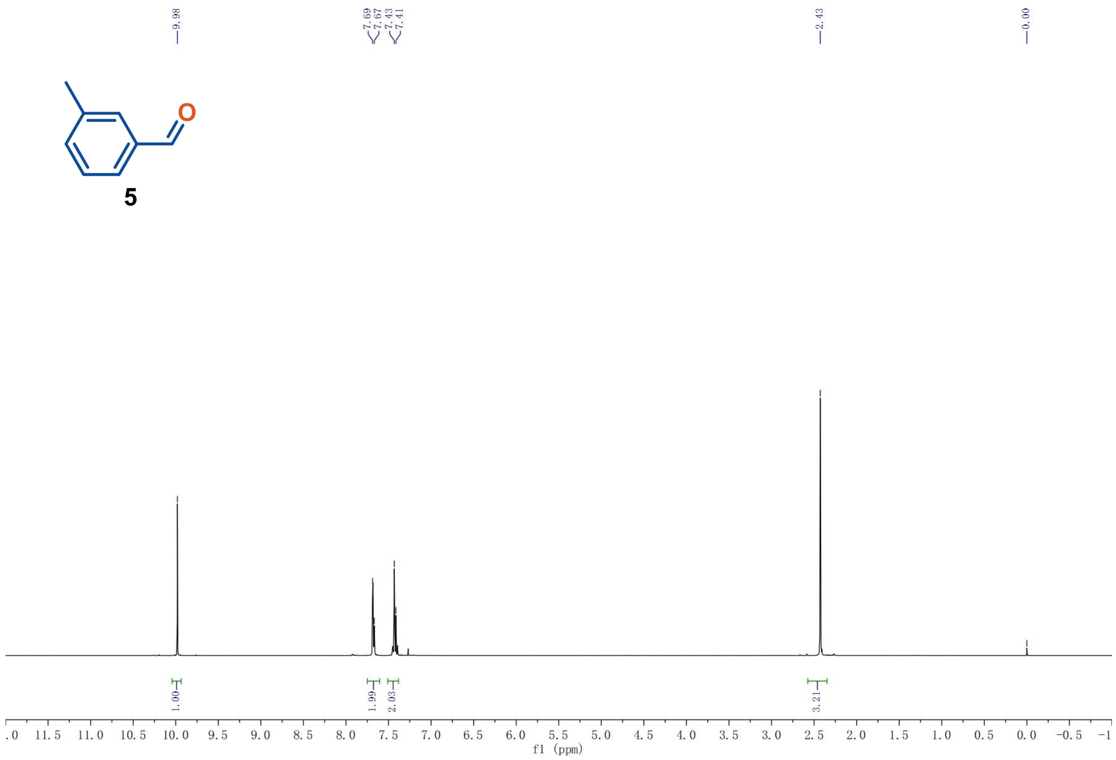

^{13}C NMR spectrum of **5**

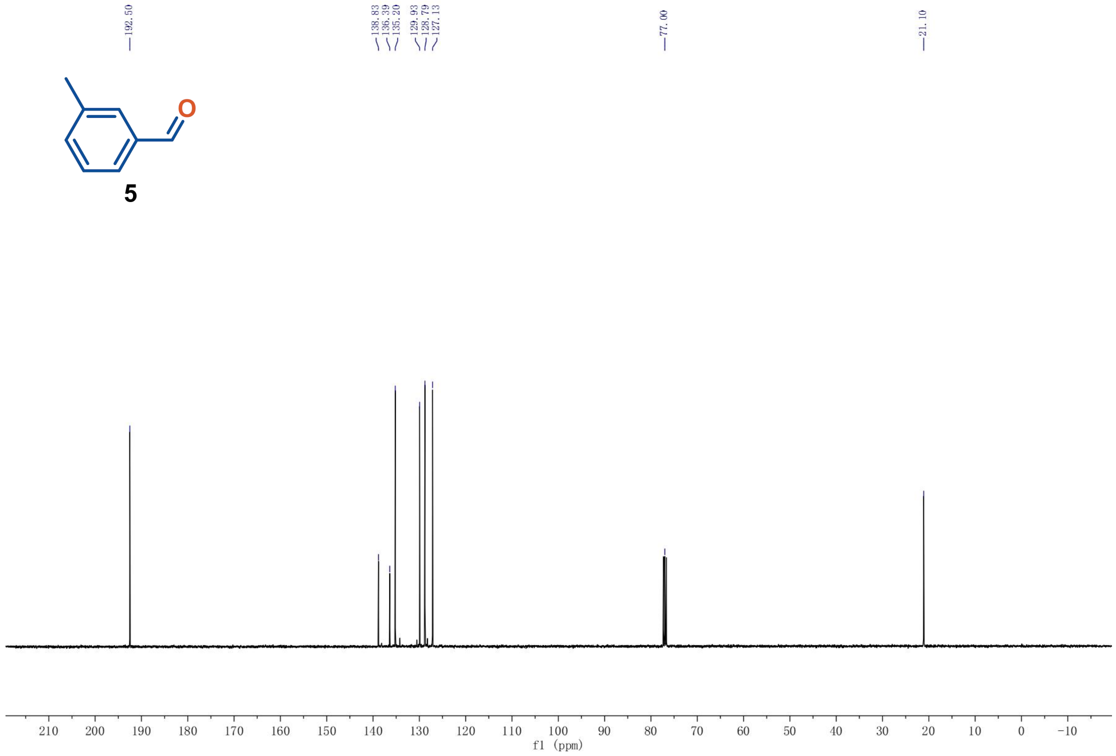

^{1}H NMR spectrum of **6**

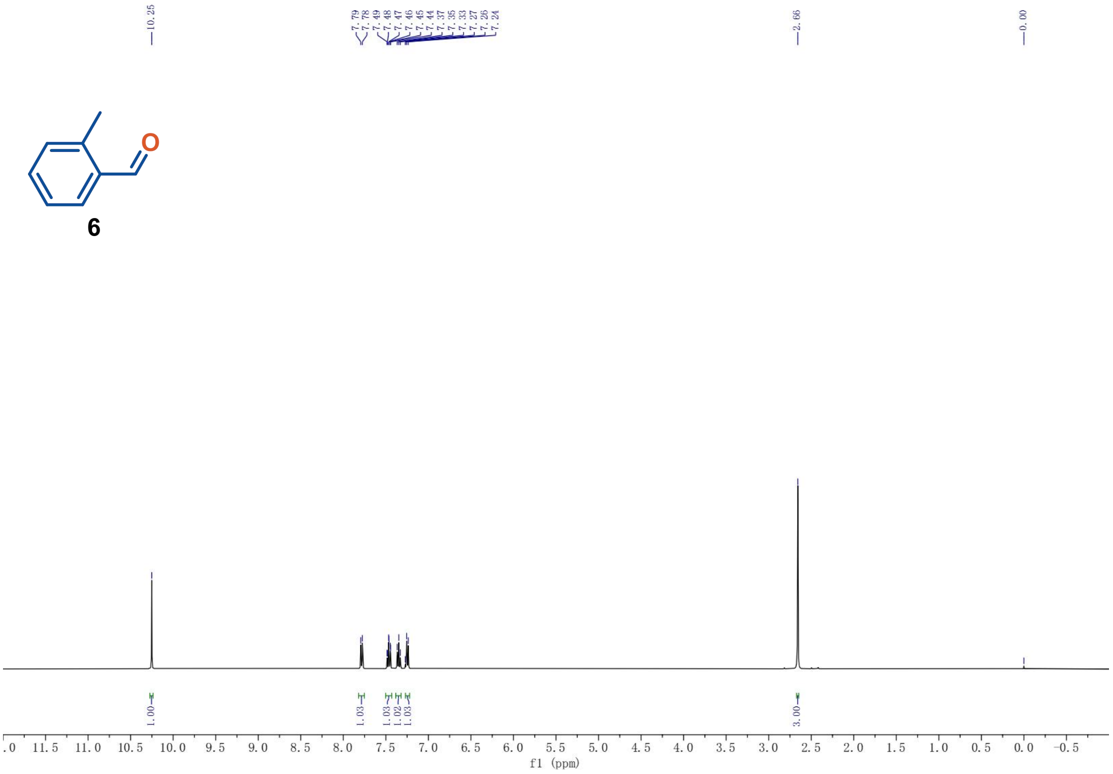

^{13}C NMR spectrum of **6**

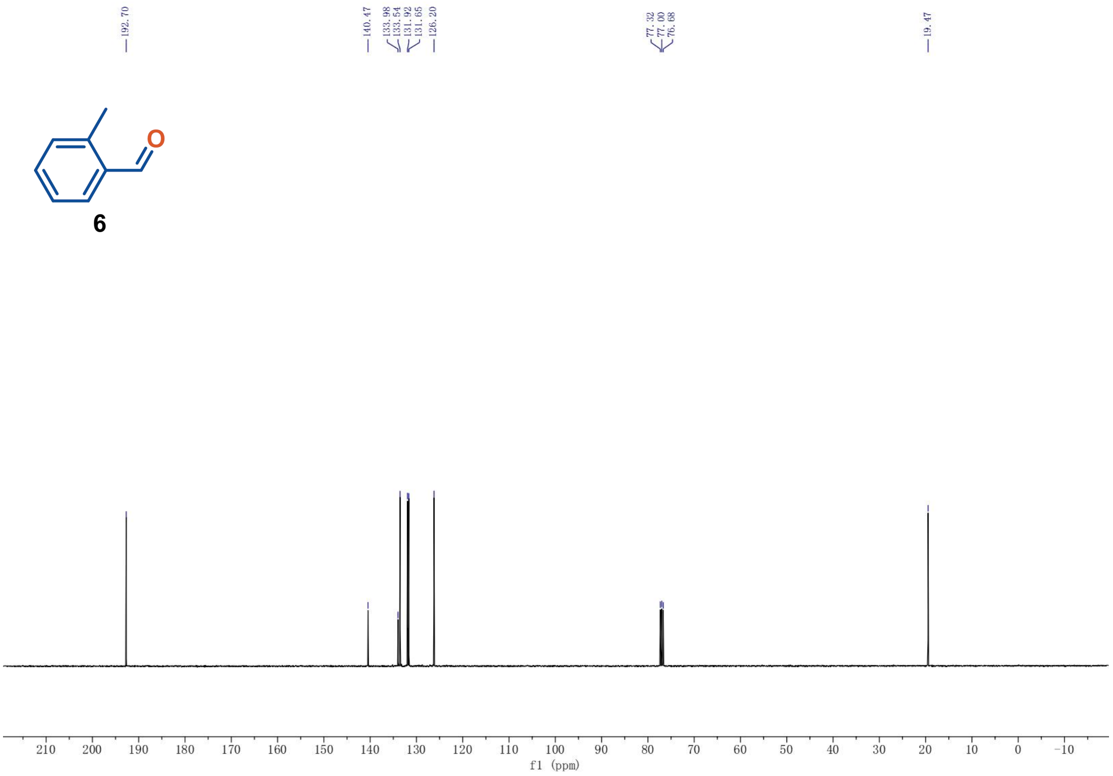

^{1}H NMR spectrum of **7**

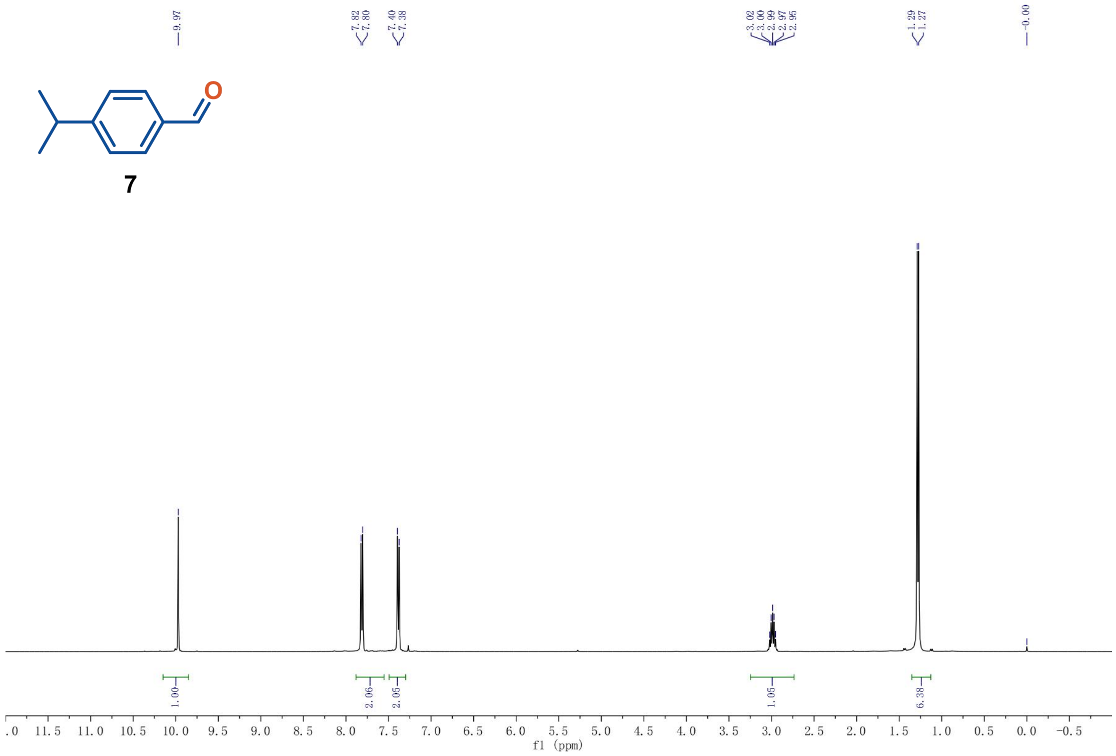

^{13}C NMR spectrum of **7**

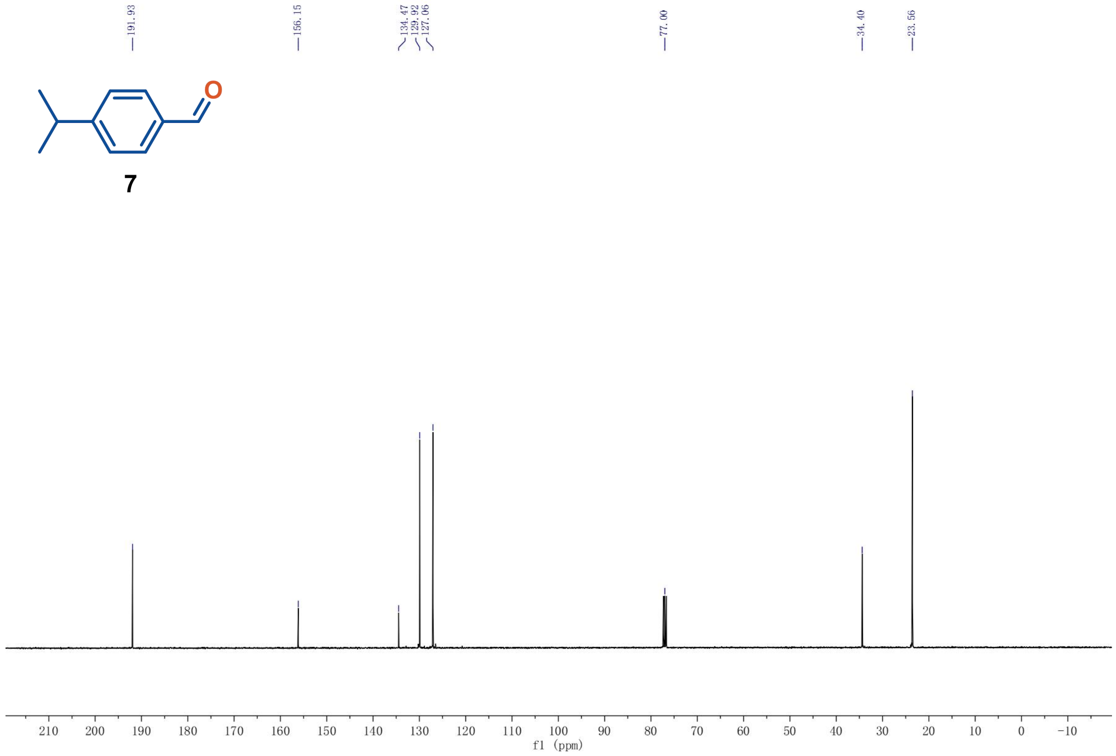

^{1}H NMR spectrum of **8**

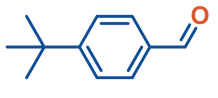

8

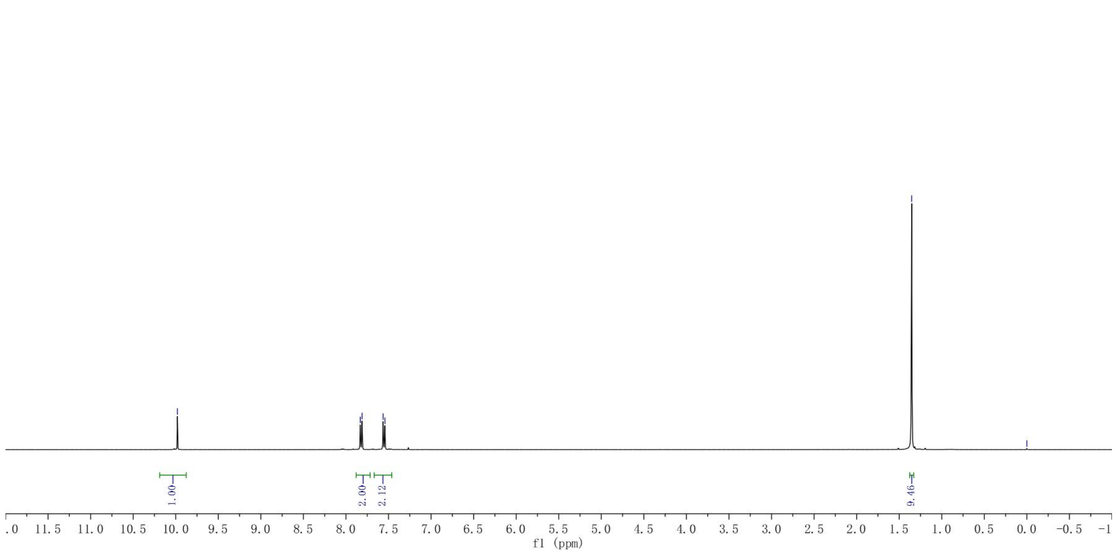

^{13}C NMR spectrum of **8**

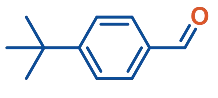

8

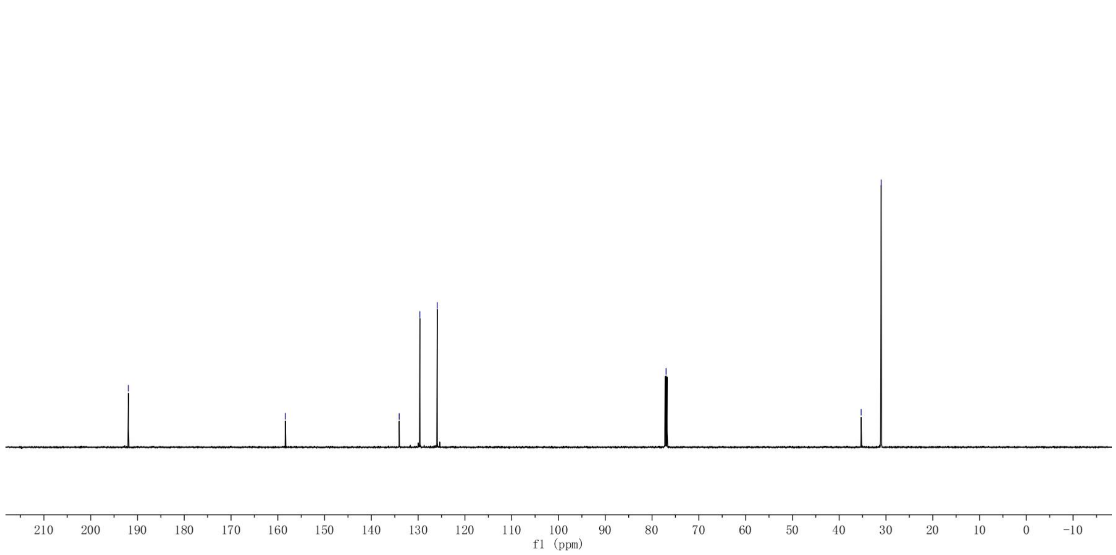

^{1}H NMR spectrum of **9**

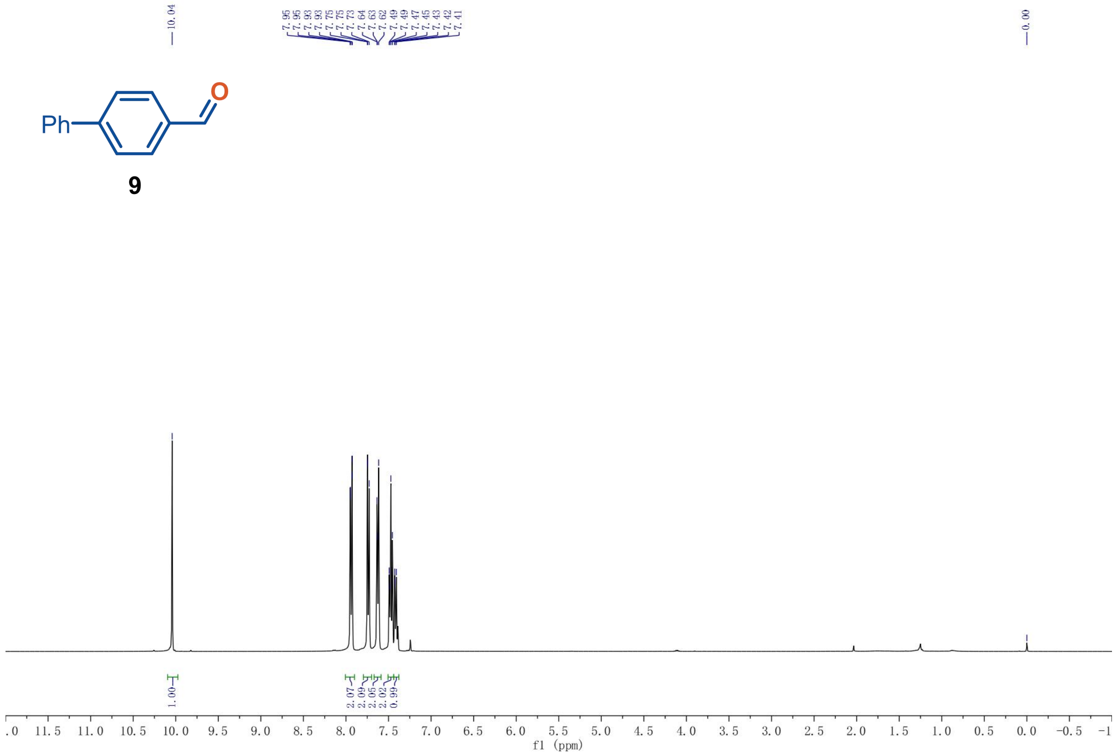

^{13}C NMR spectrum of **9**

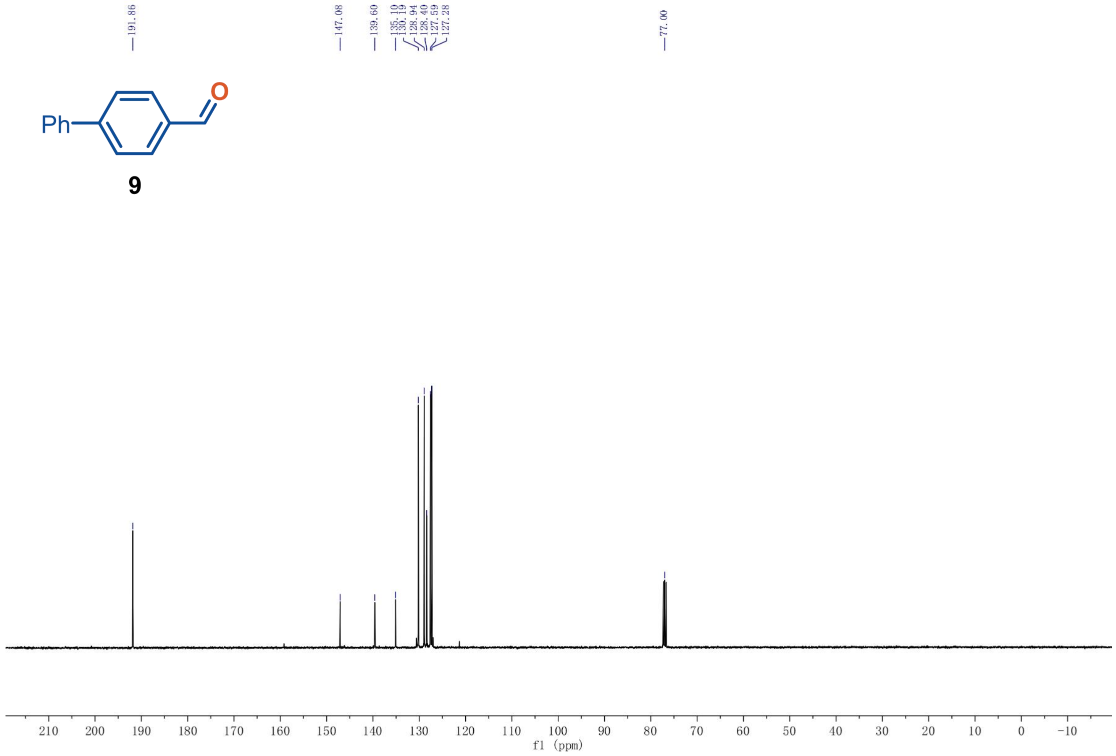

^{1}H NMR spectrum of **10**

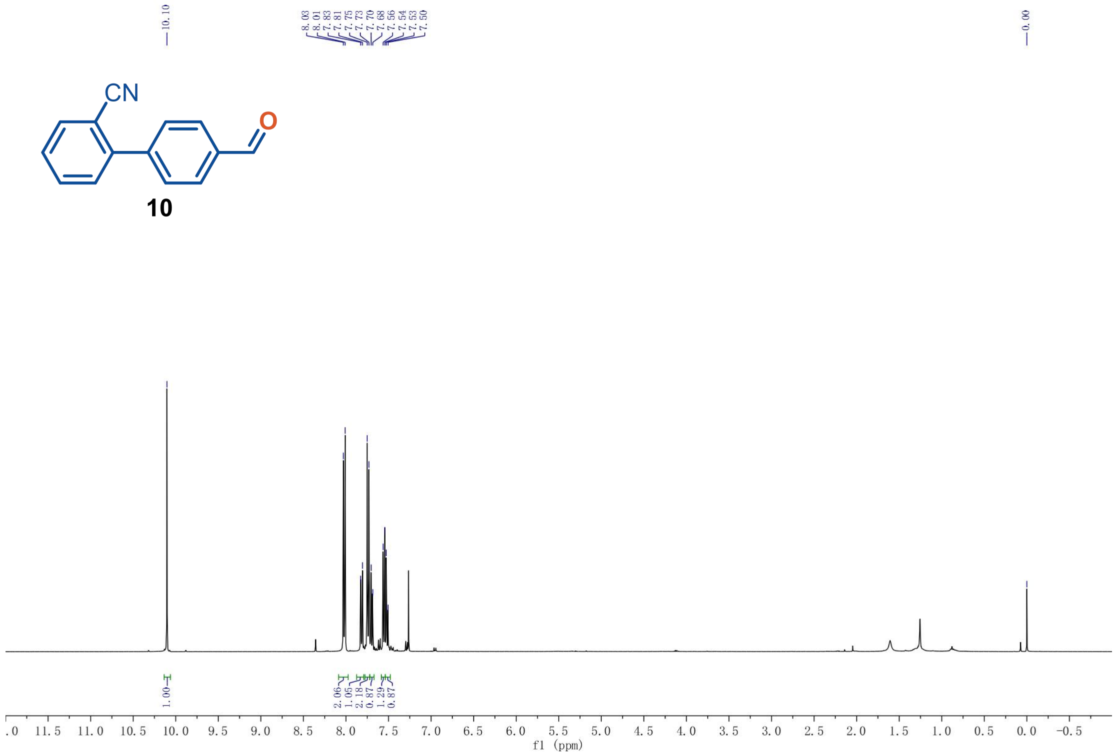

^{13}C NMR spectrum of **10**

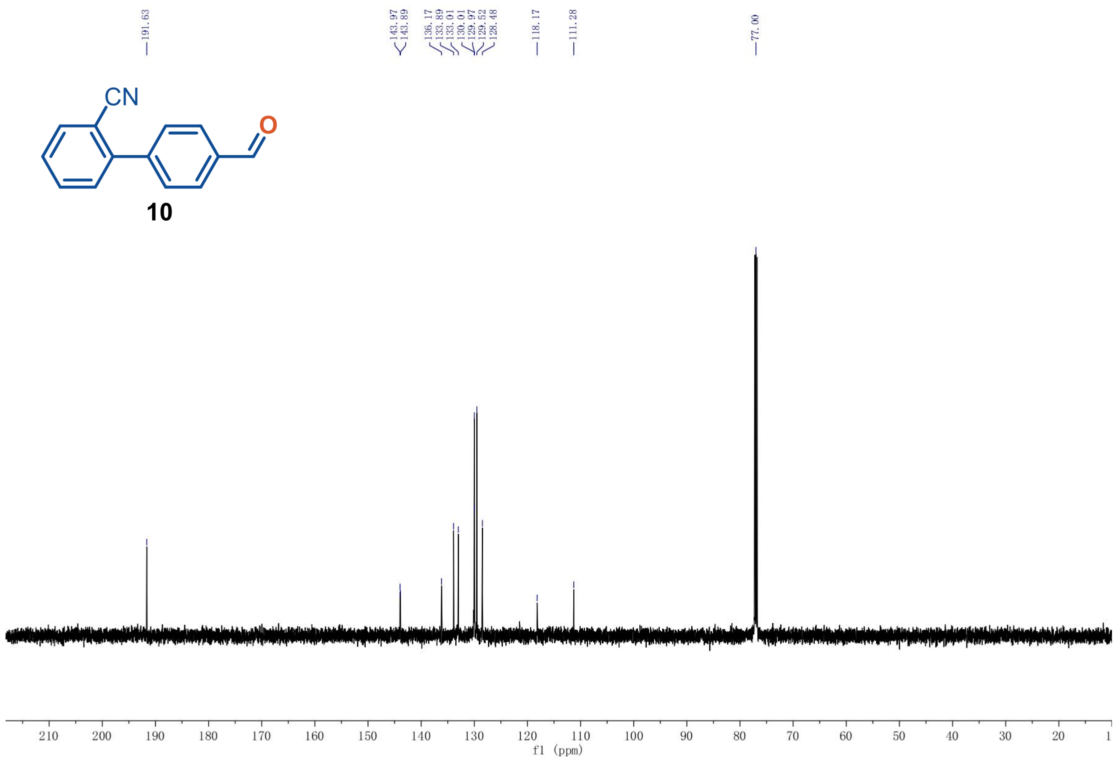

^{1}H NMR spectrum of **11**

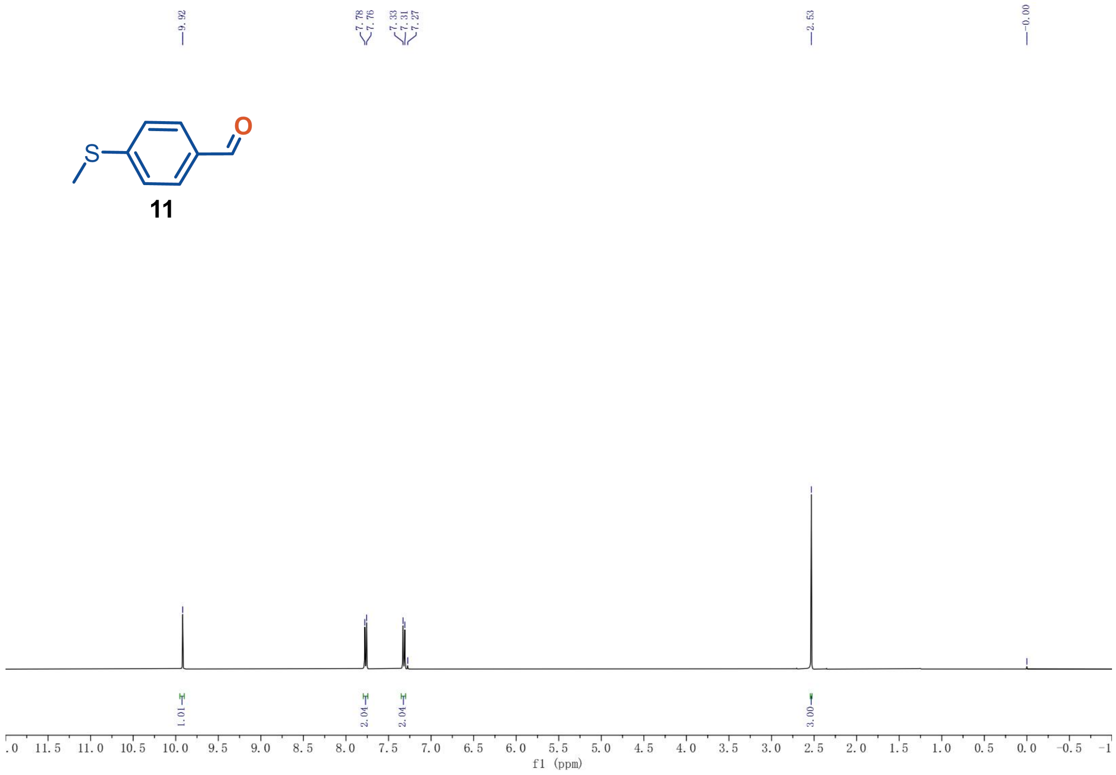

^{13}C NMR spectrum of **11**

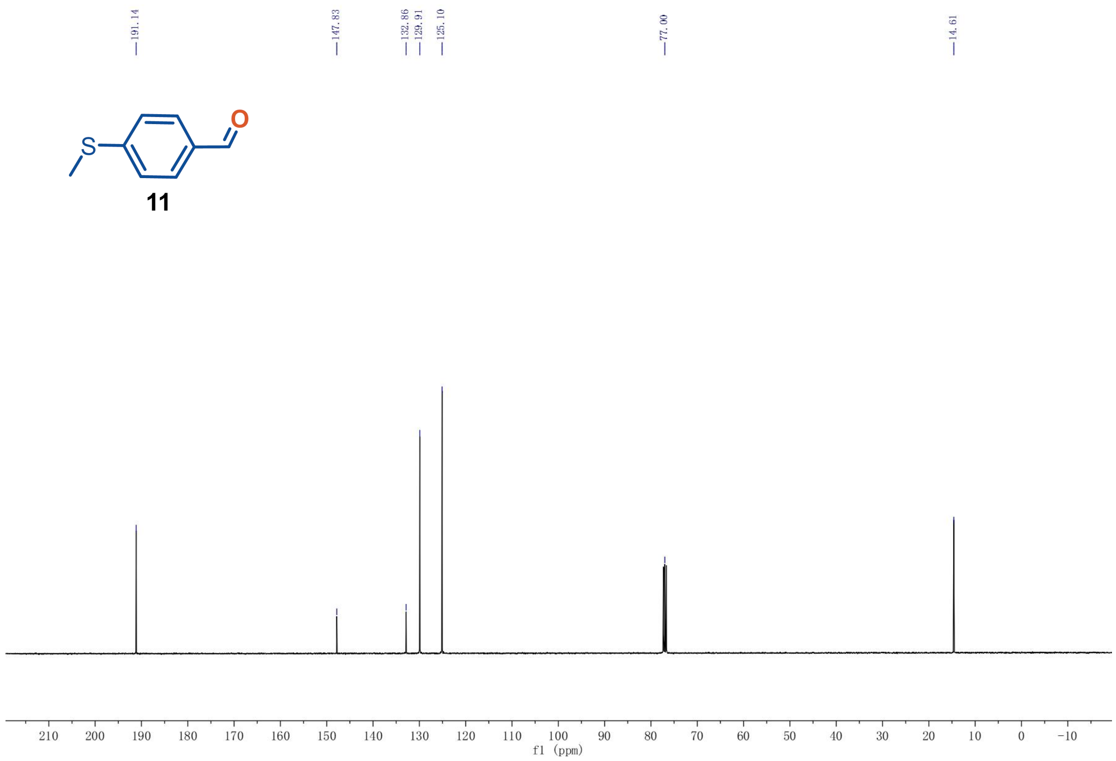

^{1}H NMR spectrum of **12**

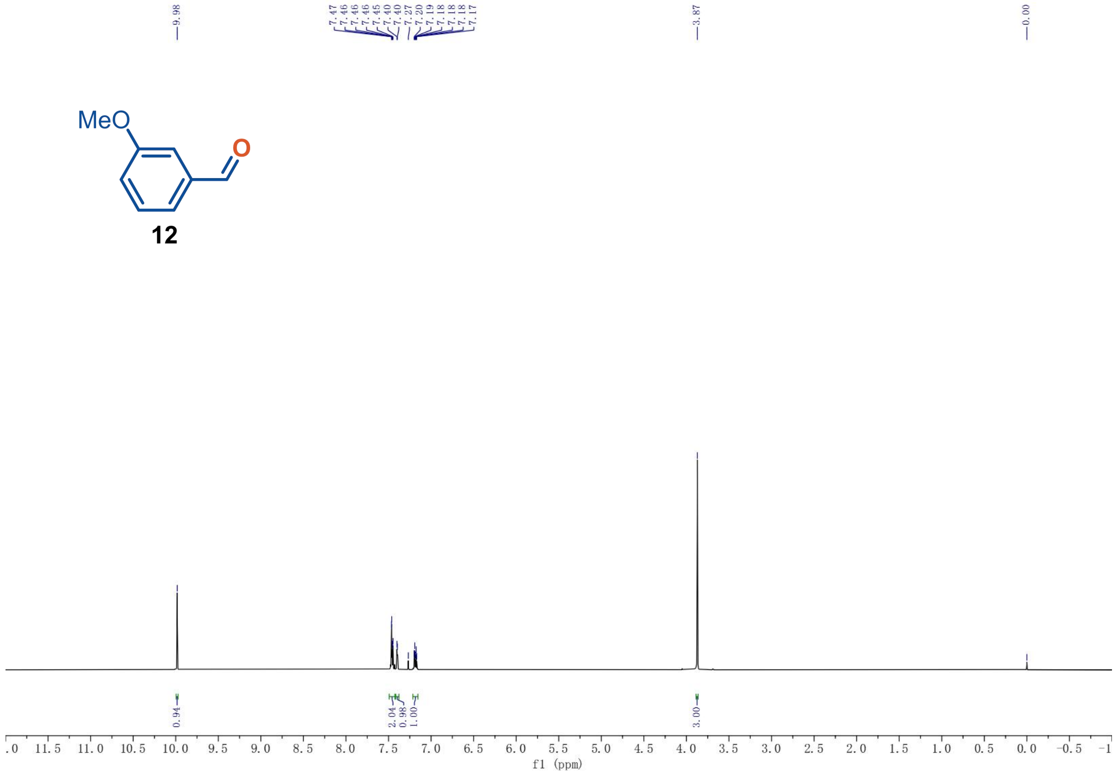

^{13}C NMR spectrum of **12**

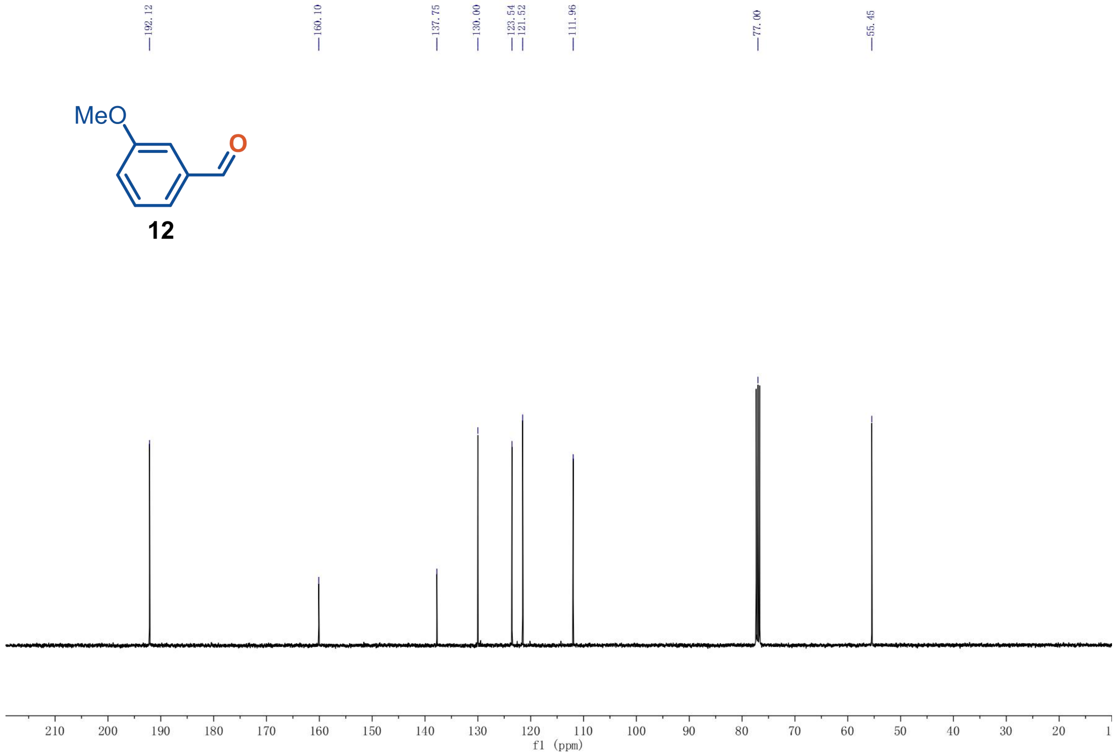

^{1}H NMR spectrum of **13**

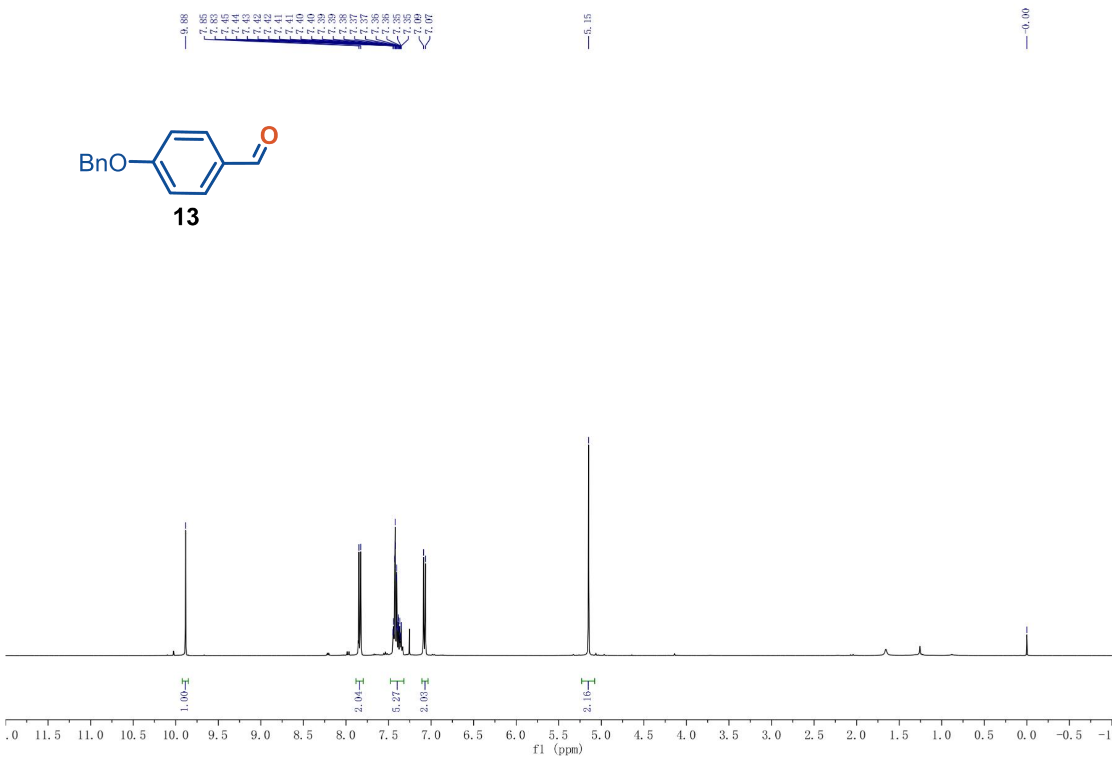

^{13}C NMR spectrum of **13**

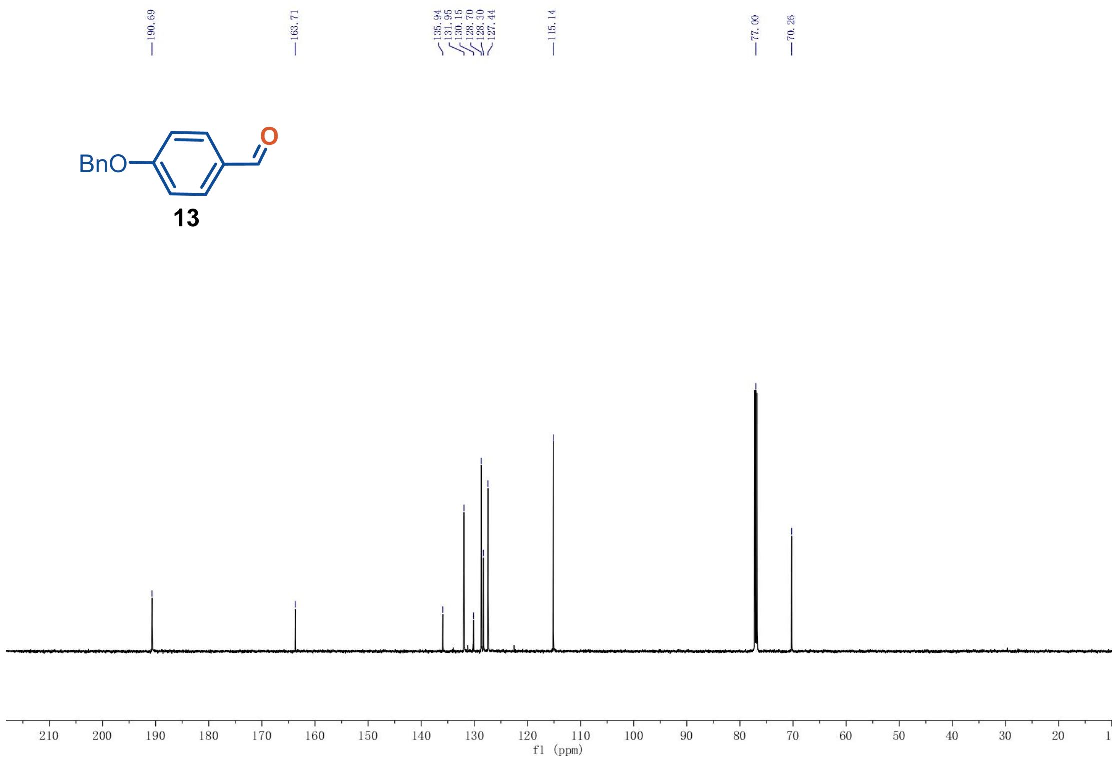

^{1}H NMR spectrum of **14**

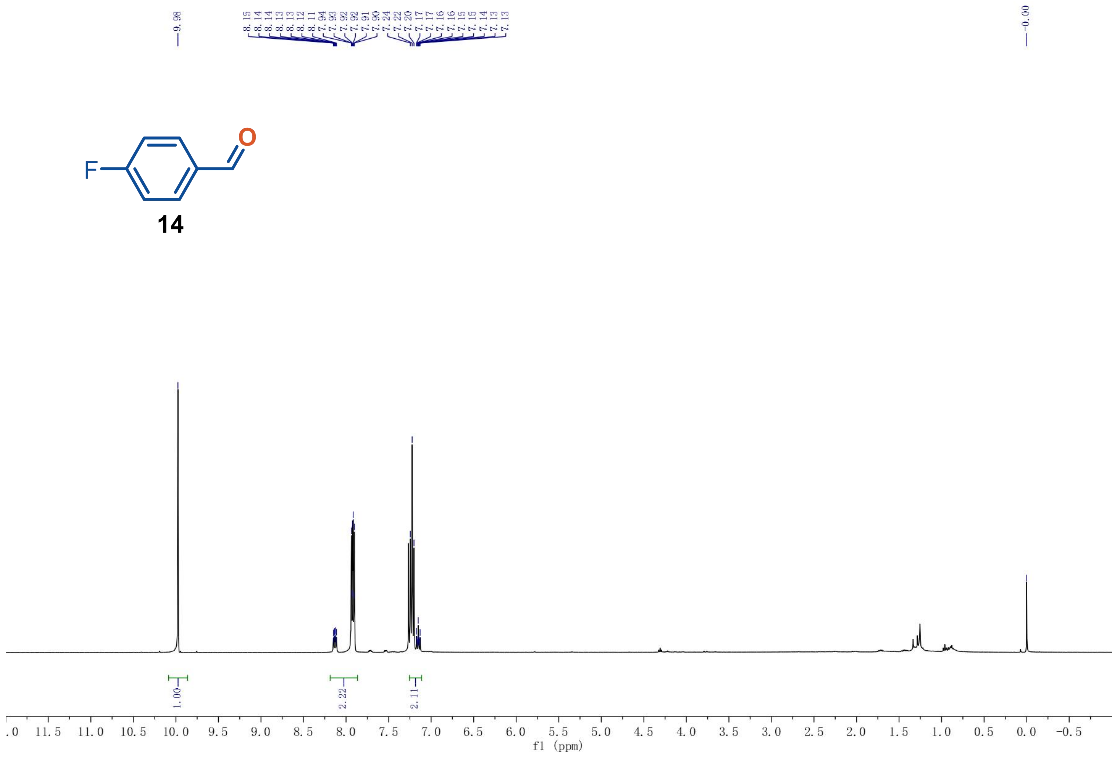

^{13}C NMR spectrum of **14**

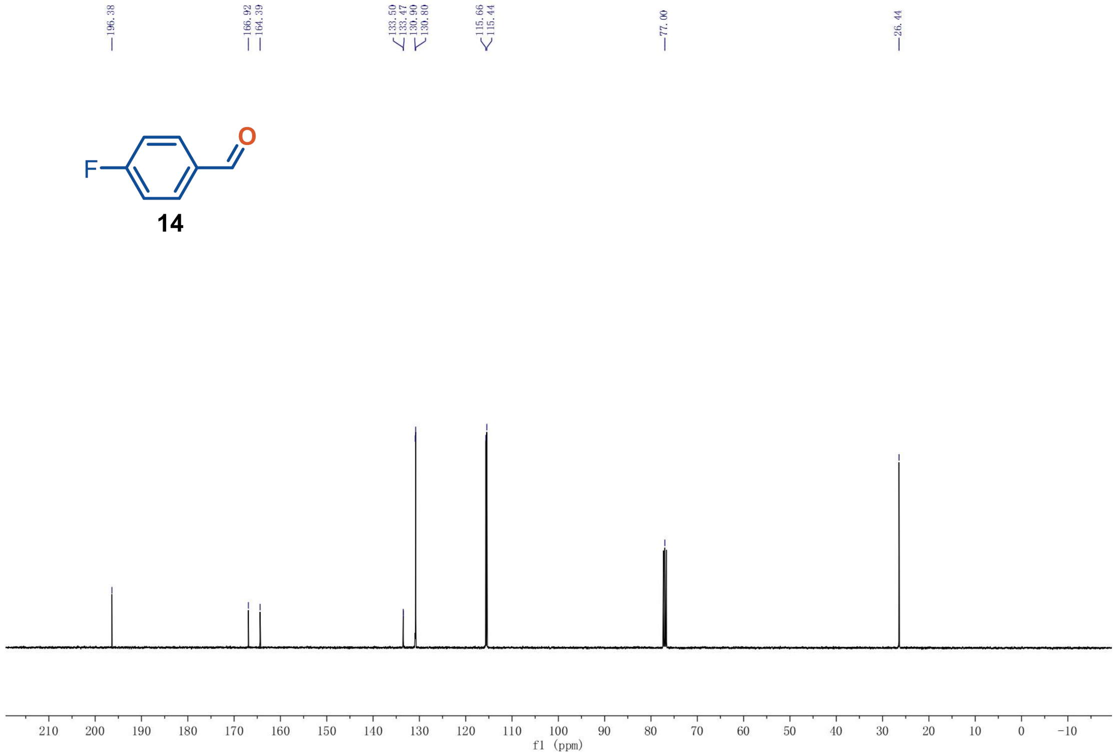

^{19F} NMR spectrum of **14**

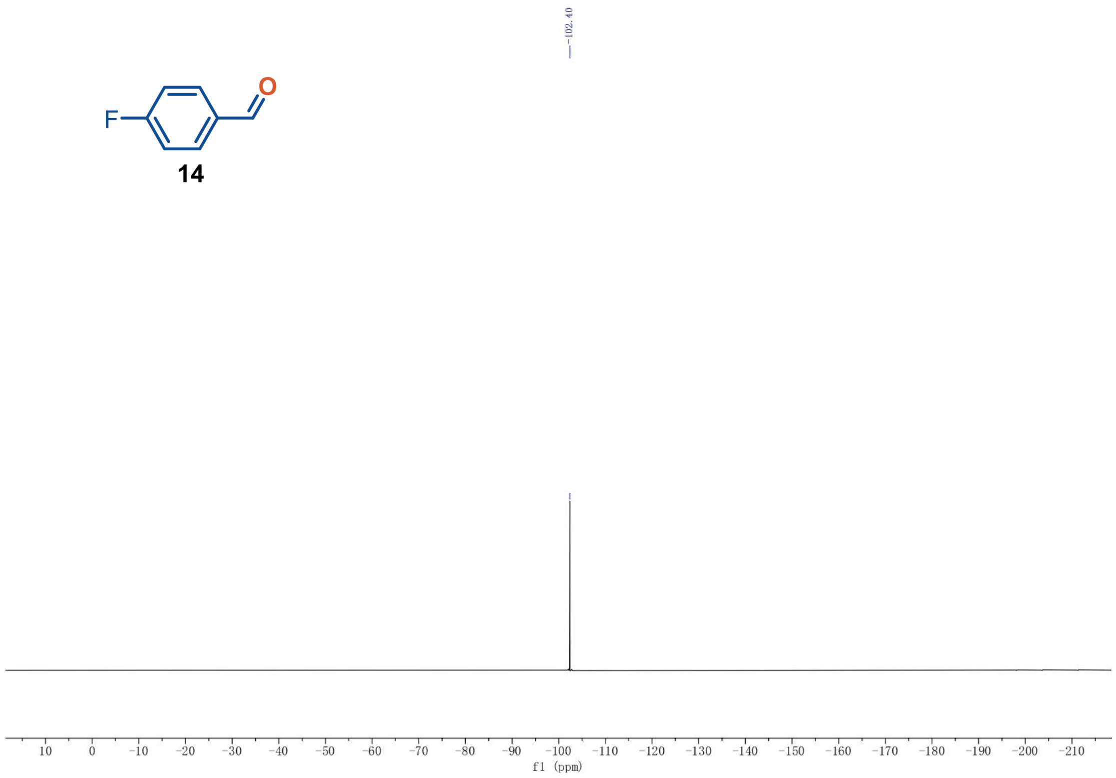

^{1}H NMR spectrum of **15**

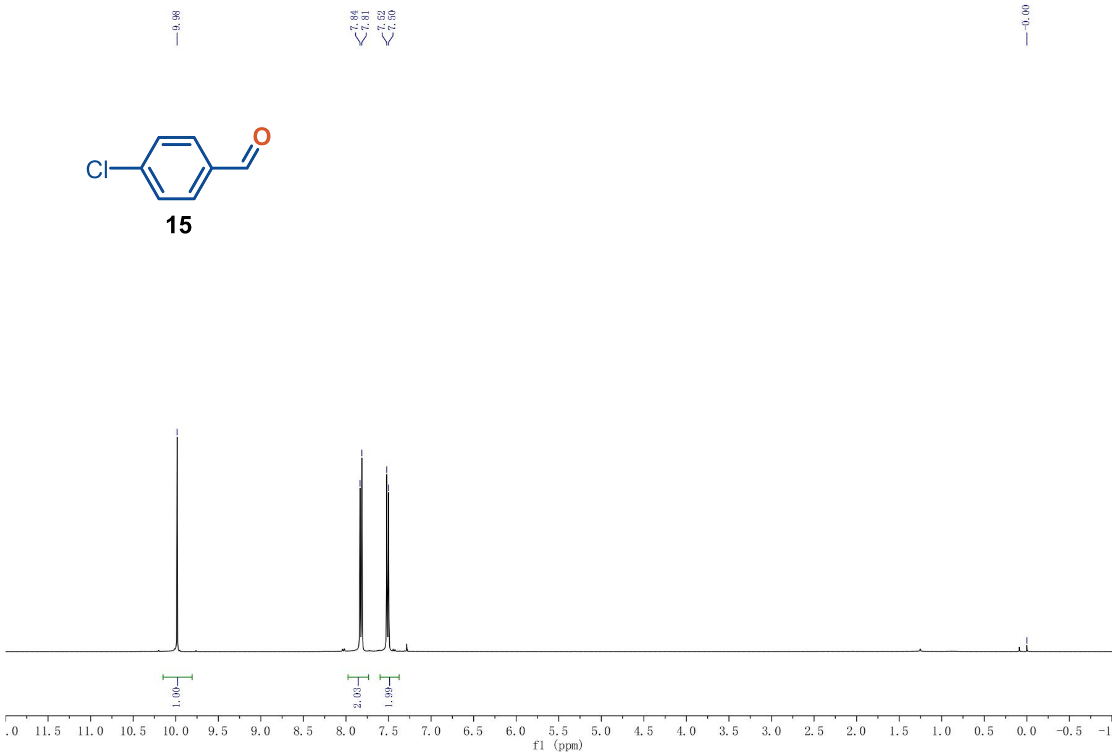

^{13}C NMR spectrum of **15**

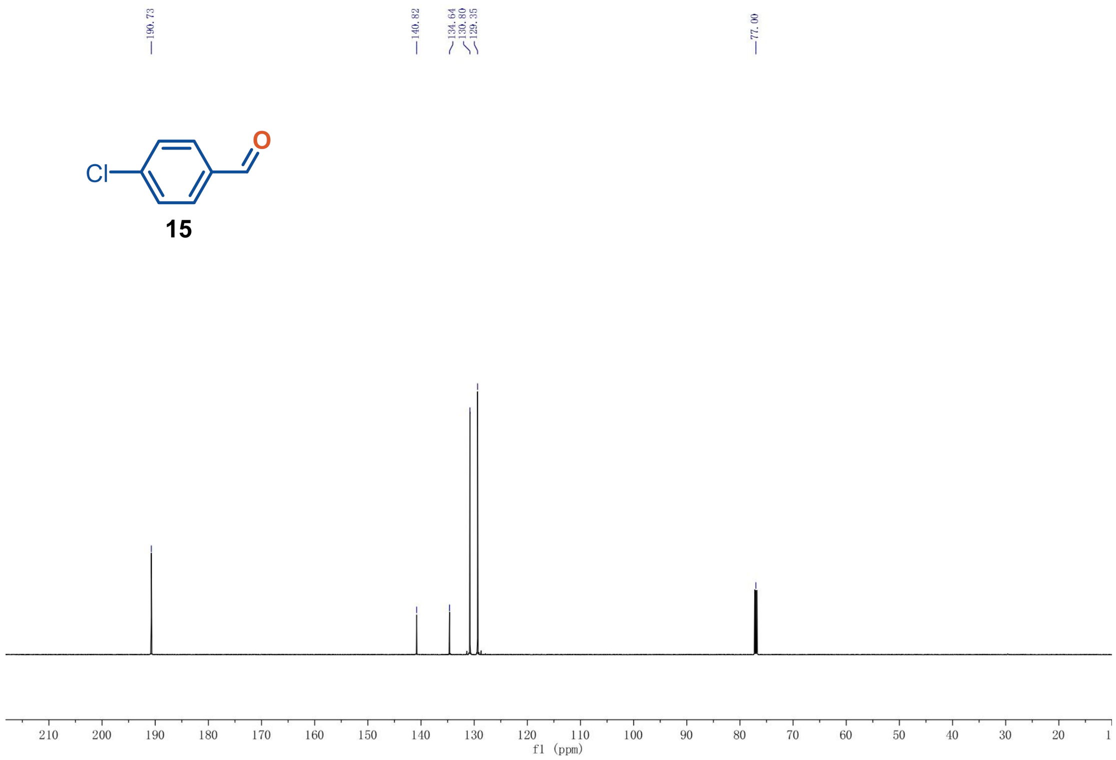

^{1}H NMR spectrum of **16**

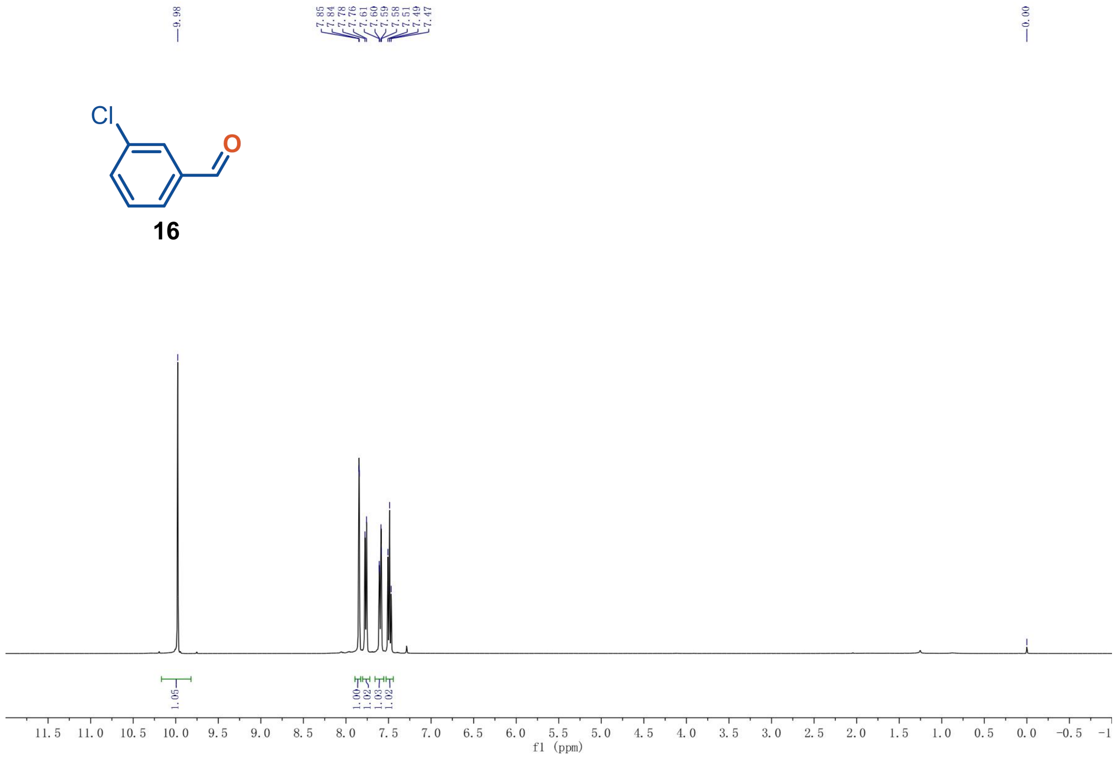

^{13}C NMR spectrum of **16**

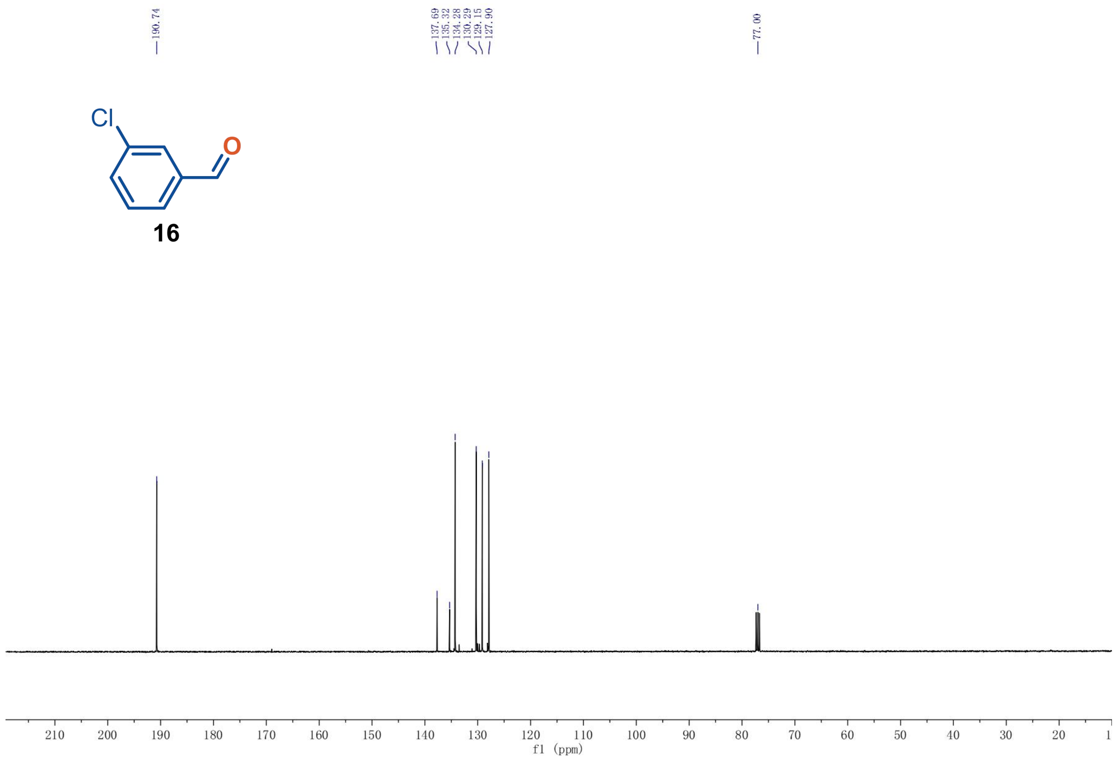

^{1}H NMR spectrum of **17**

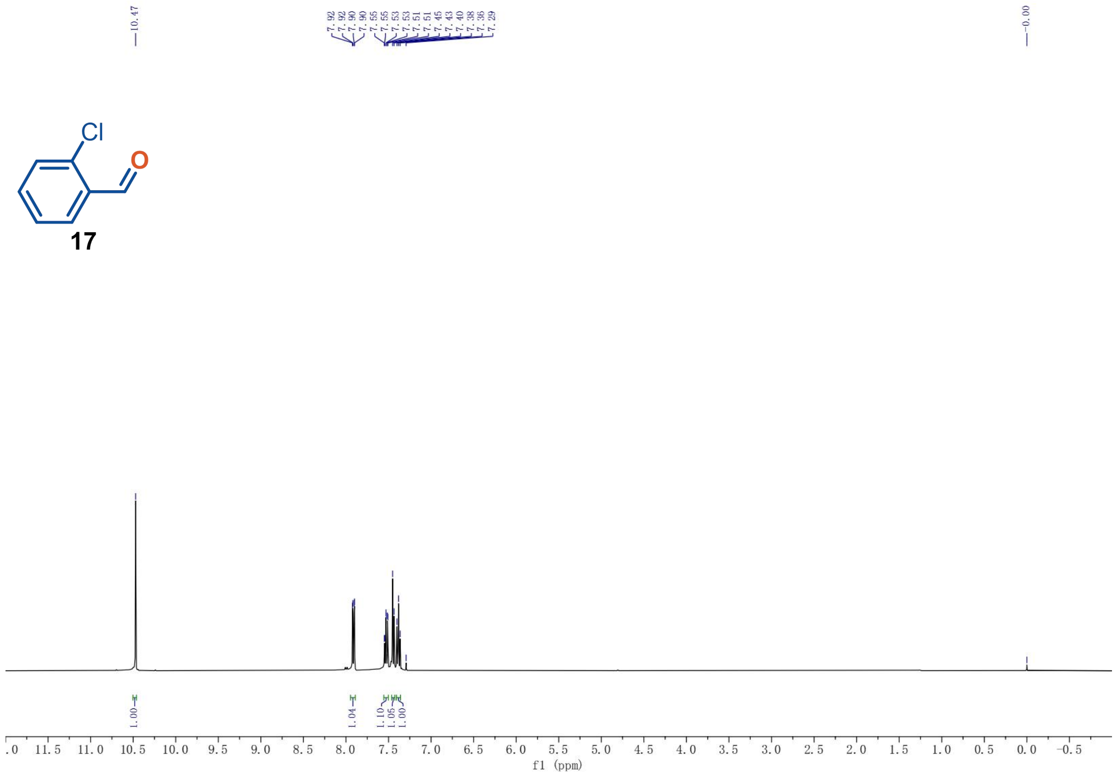

^{13}C NMR spectrum of **17**

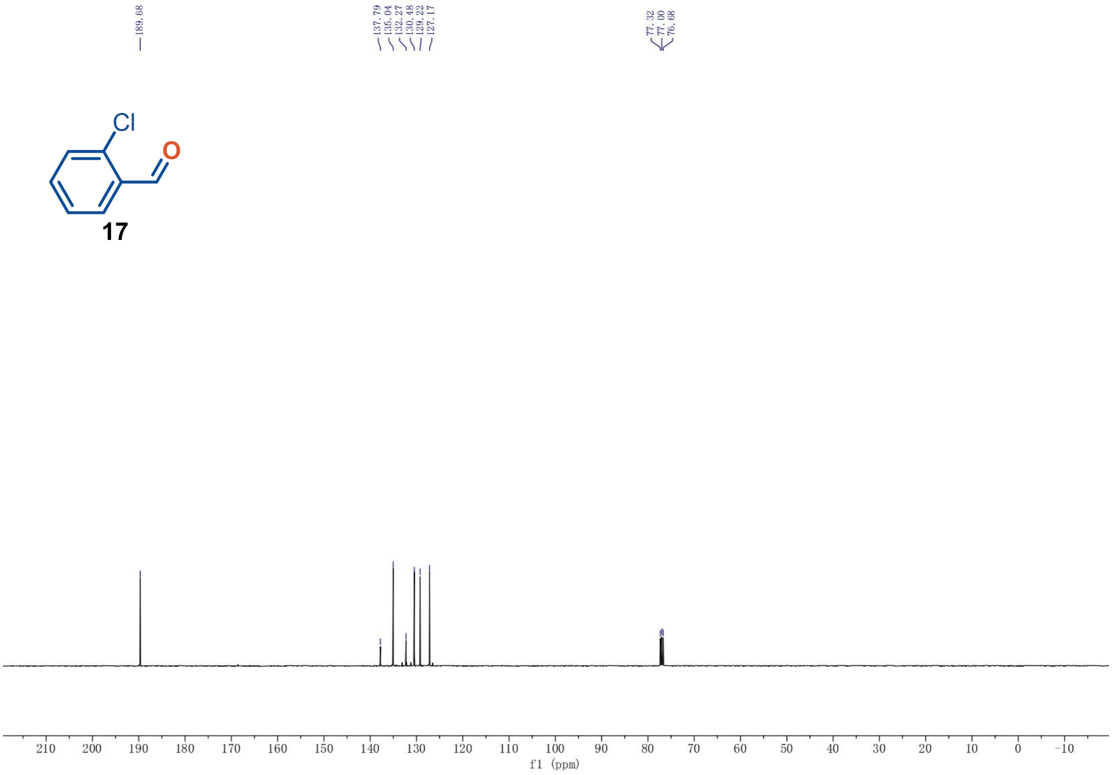

^{1}H NMR spectrum of **18**

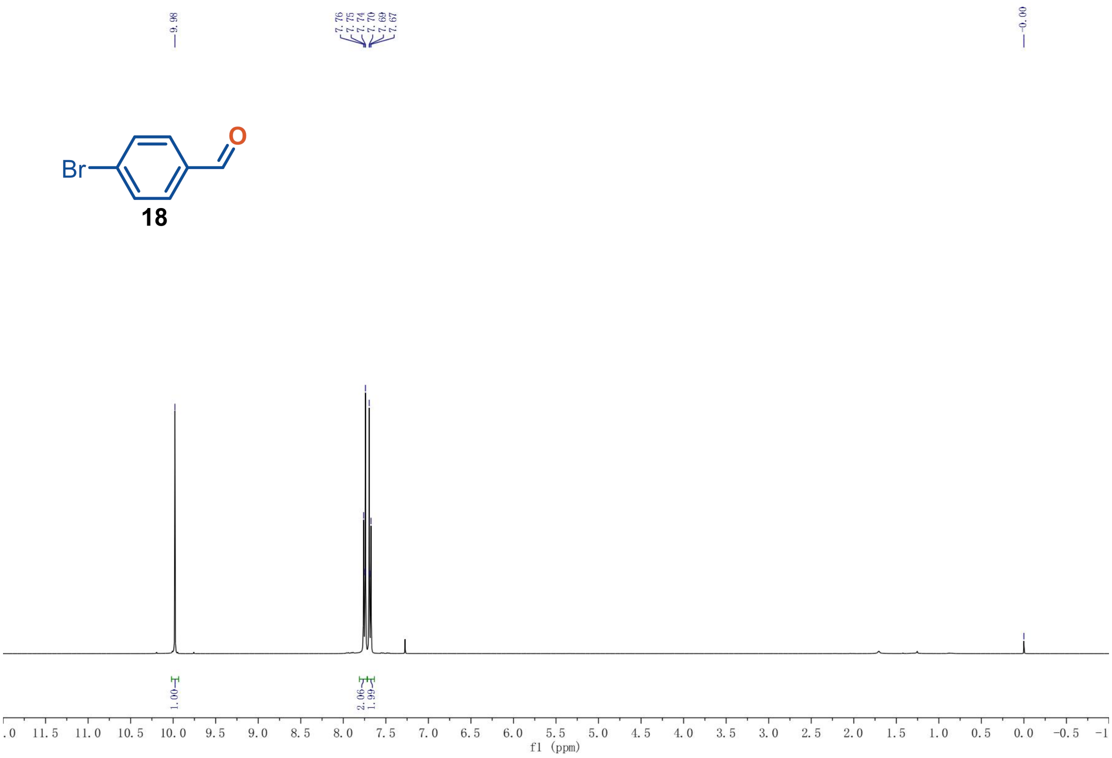

^{13}C NMR spectrum of **18**

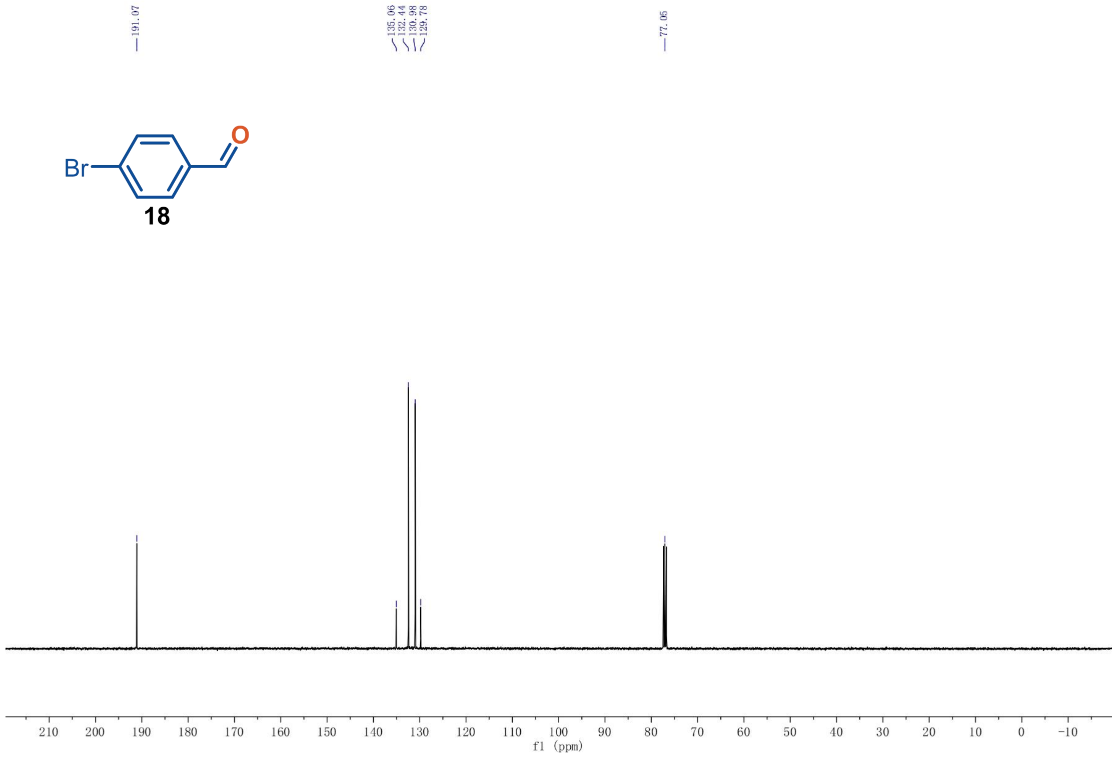

^{1}H NMR spectrum of **19**

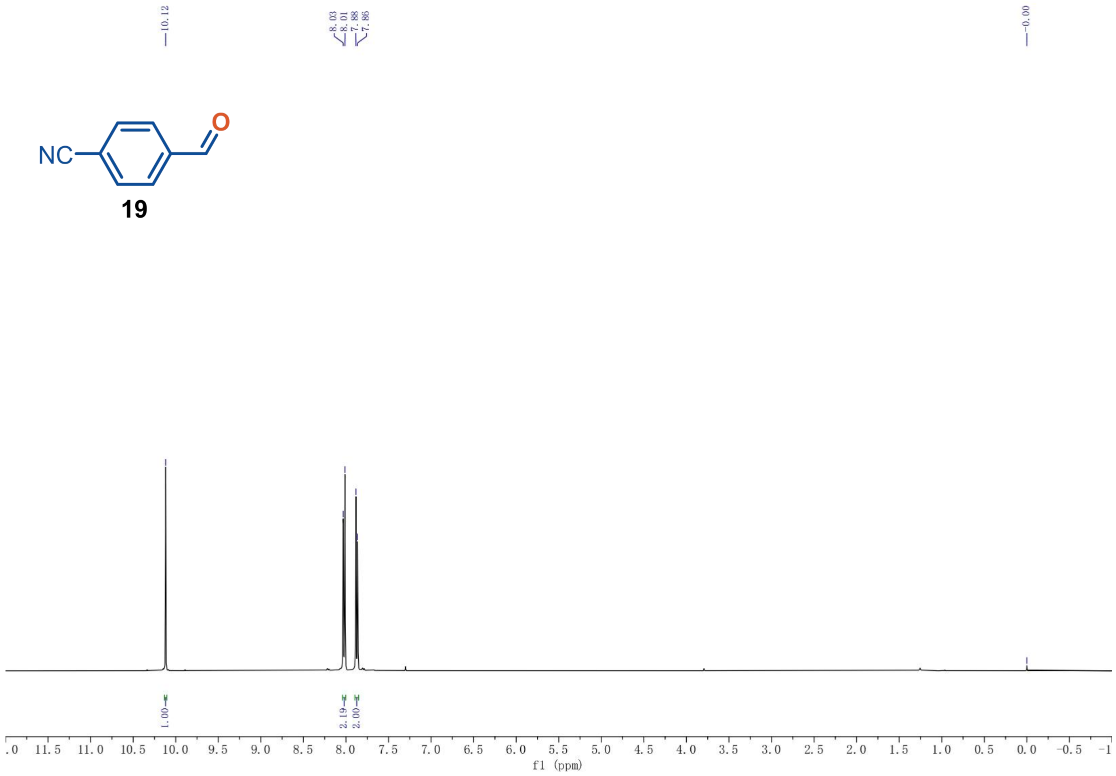

^{13}C NMR spectrum of **19**

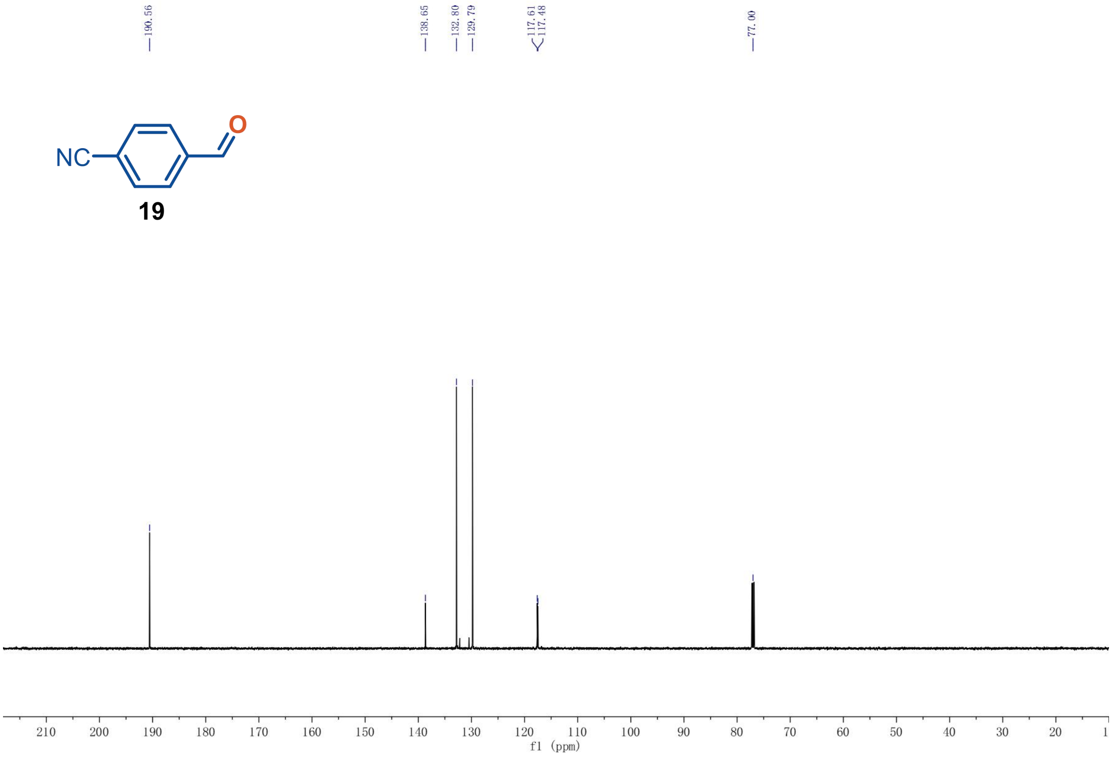

^{1}H NMR spectrum of **20**

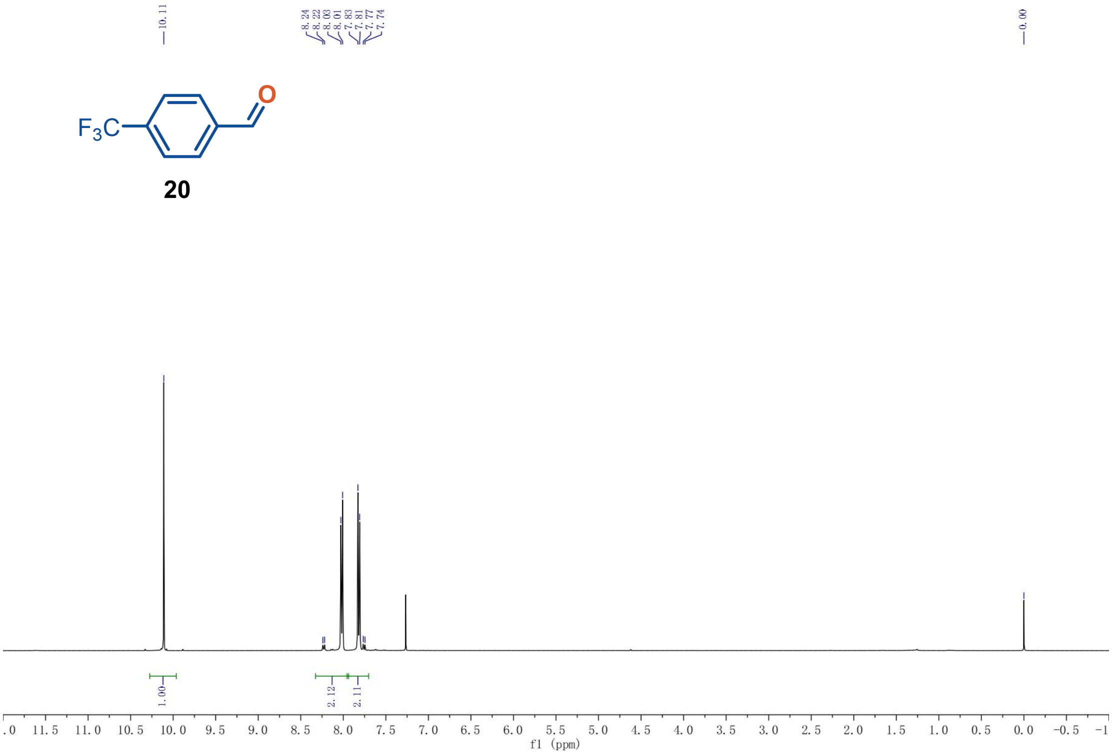

^{13}C NMR spectrum of **20**

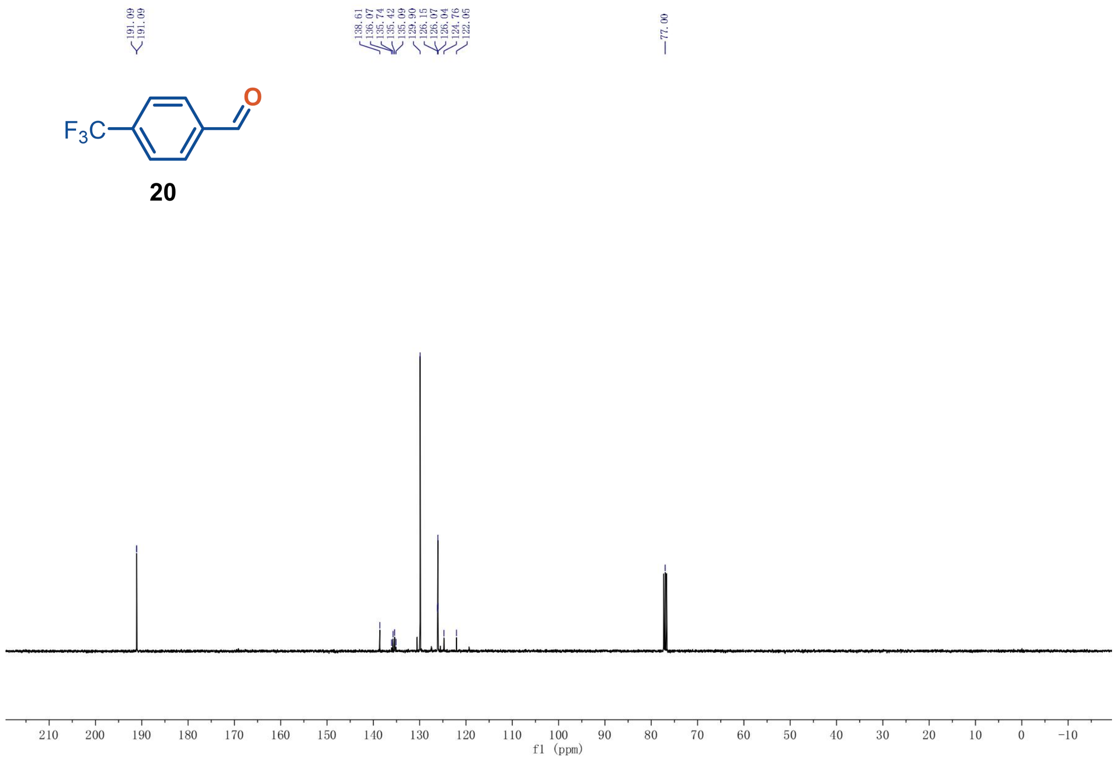

^{19}F NMR spectrum of **20**

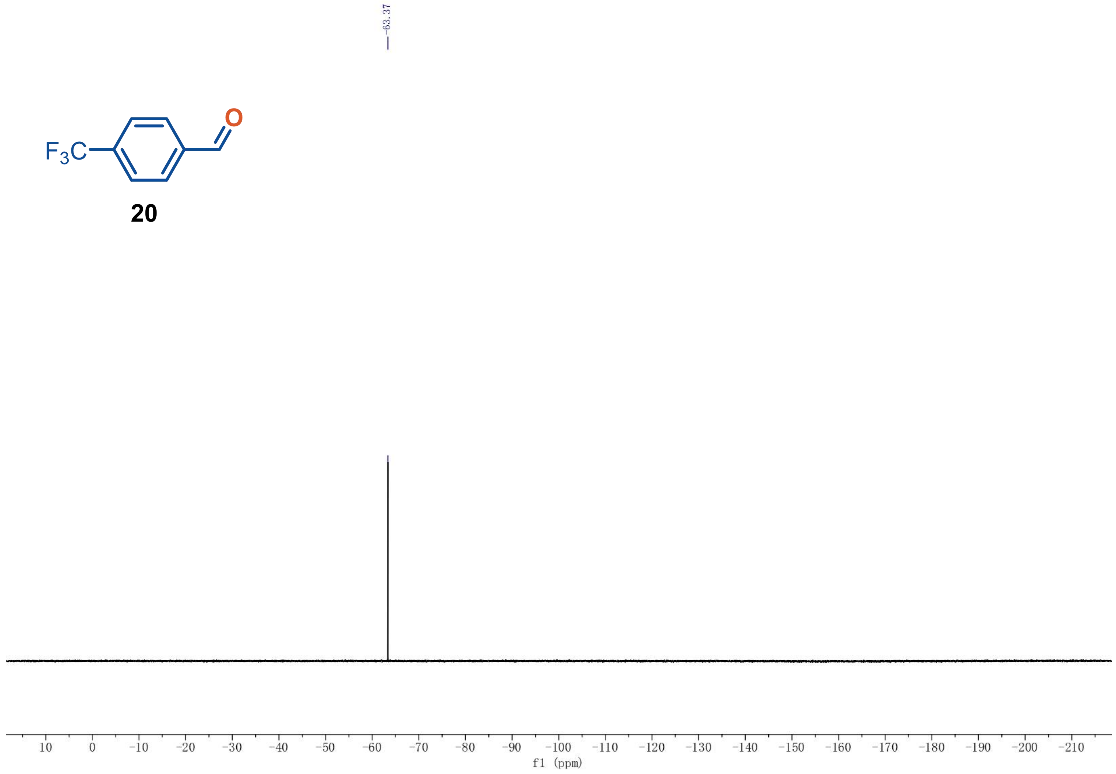

^{1}H NMR spectrum of **21**

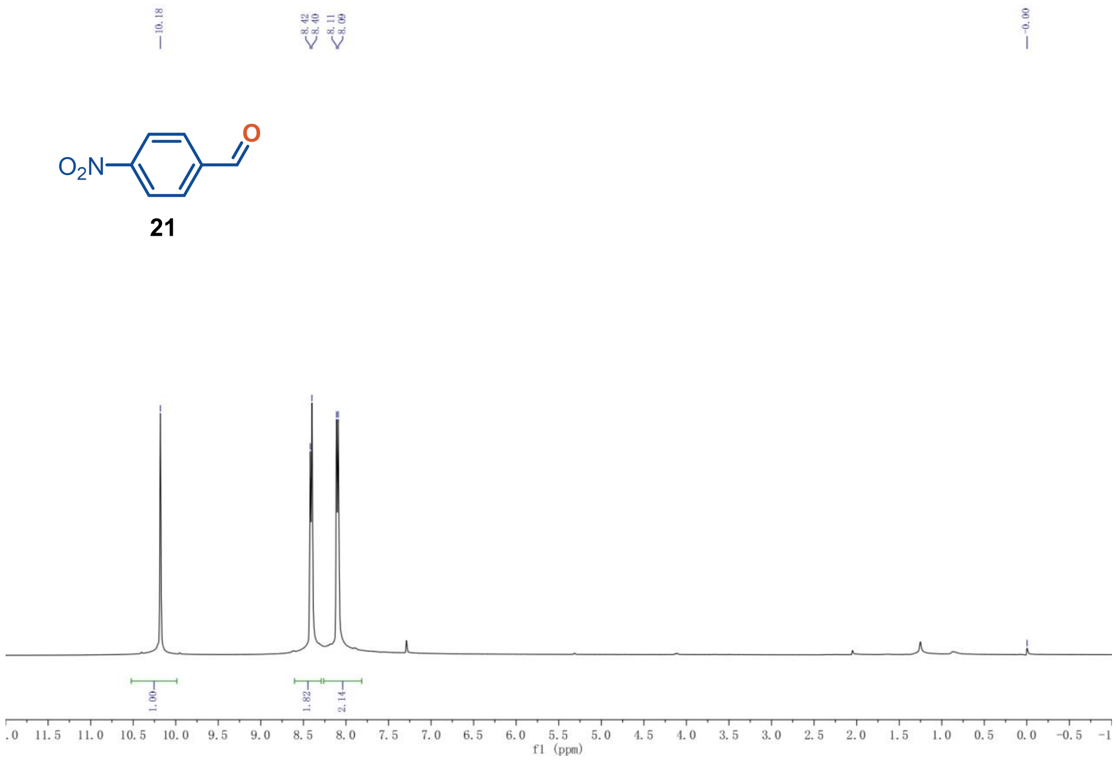

^{13}C NMR spectrum of **21**

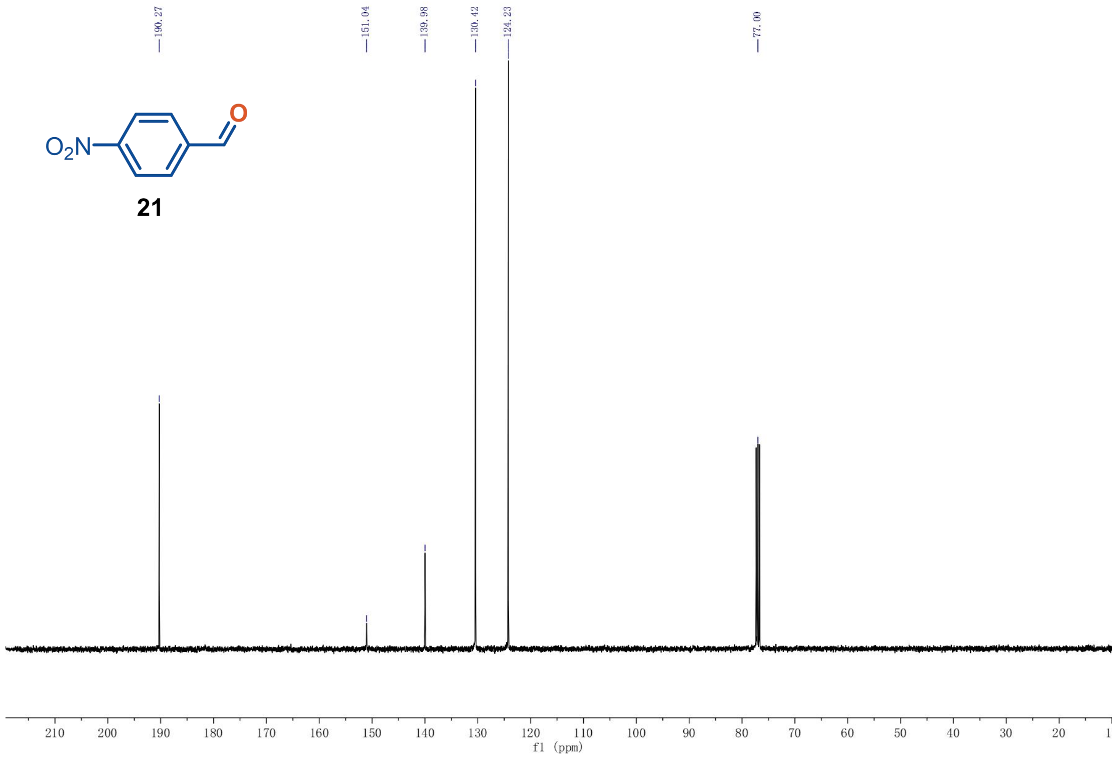

^{1}H NMR spectrum of **22**

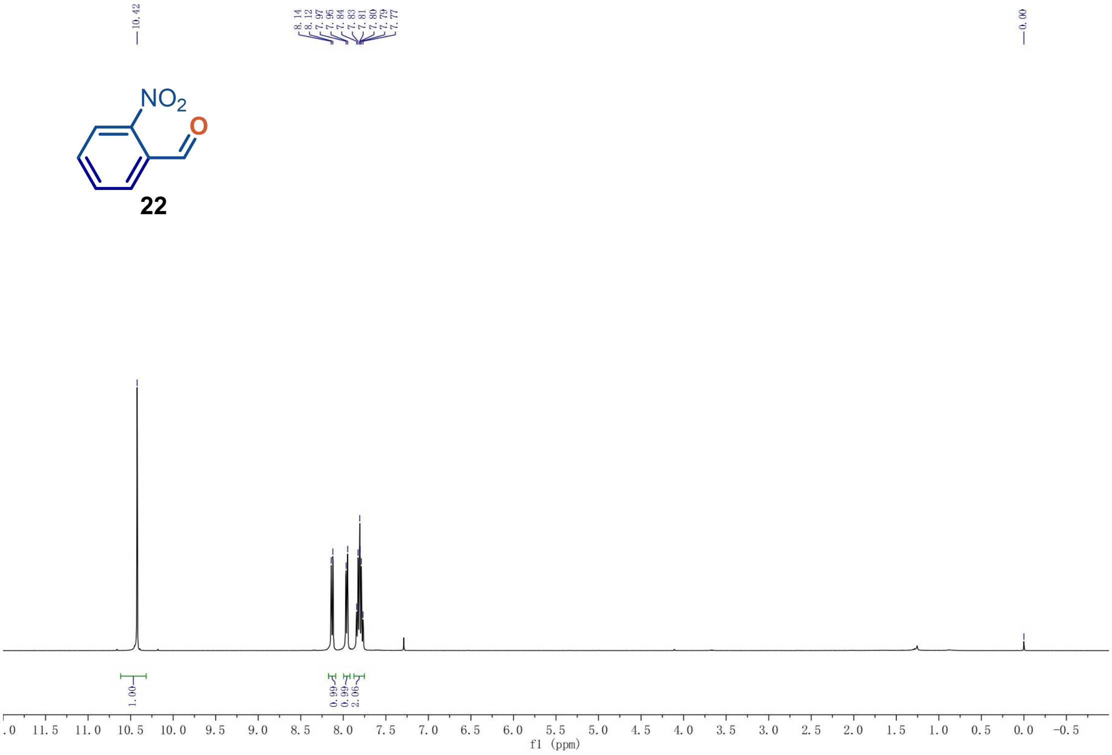

^{13}C NMR spectrum of **22**

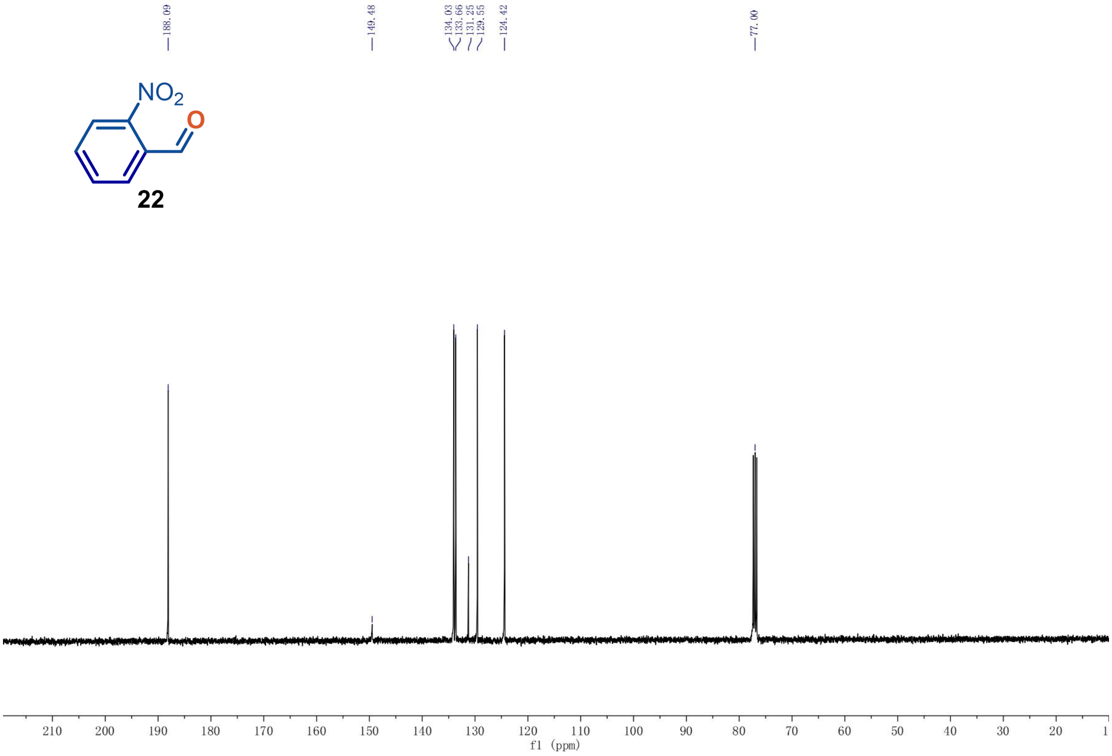

^{1}H NMR spectrum of **23**

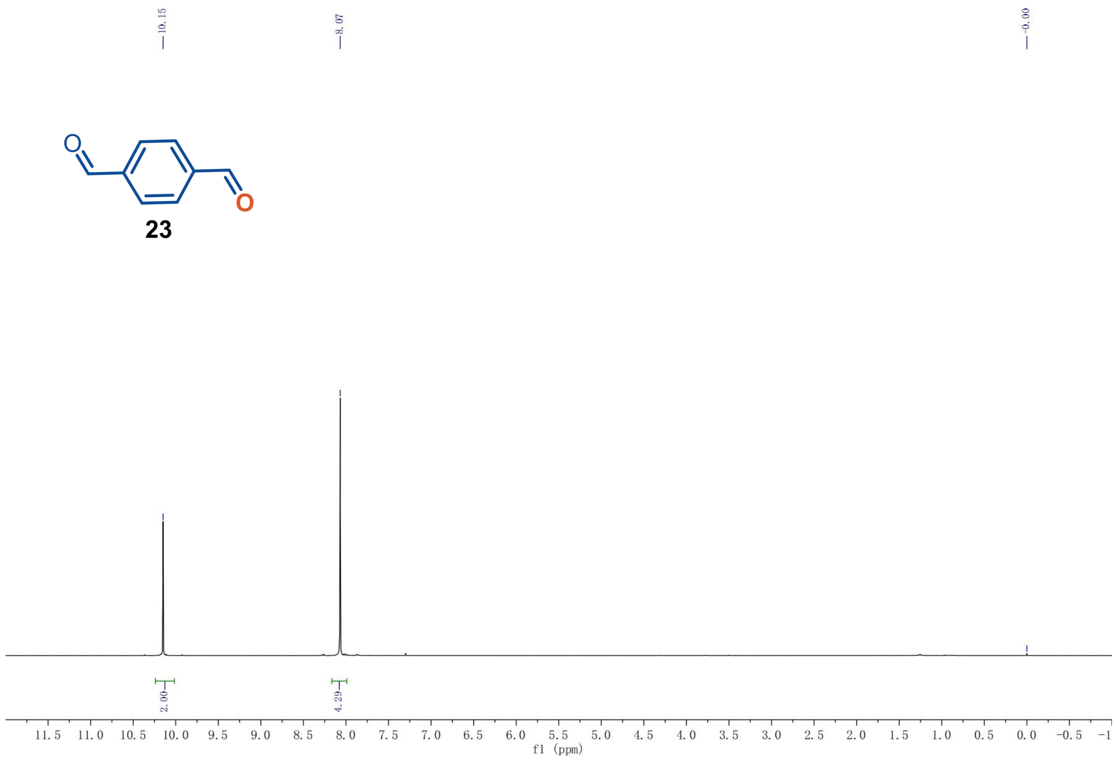

^{13}C NMR spectrum of **23**

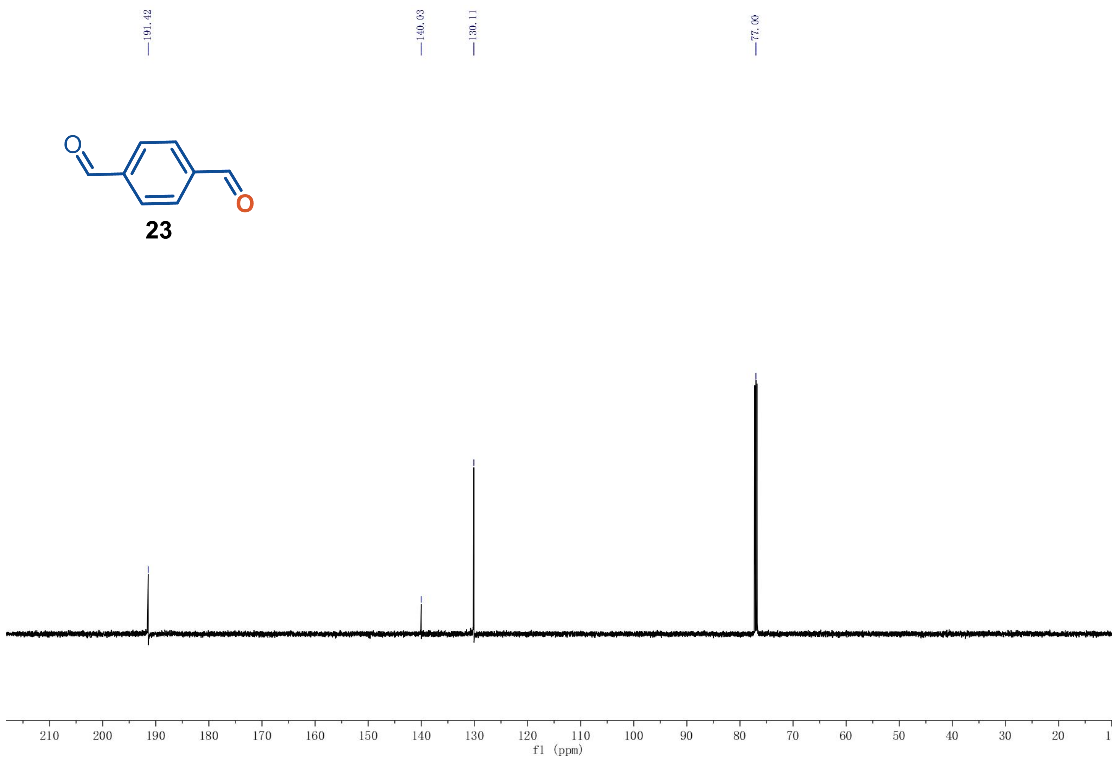

^{1}H NMR spectrum of **24**

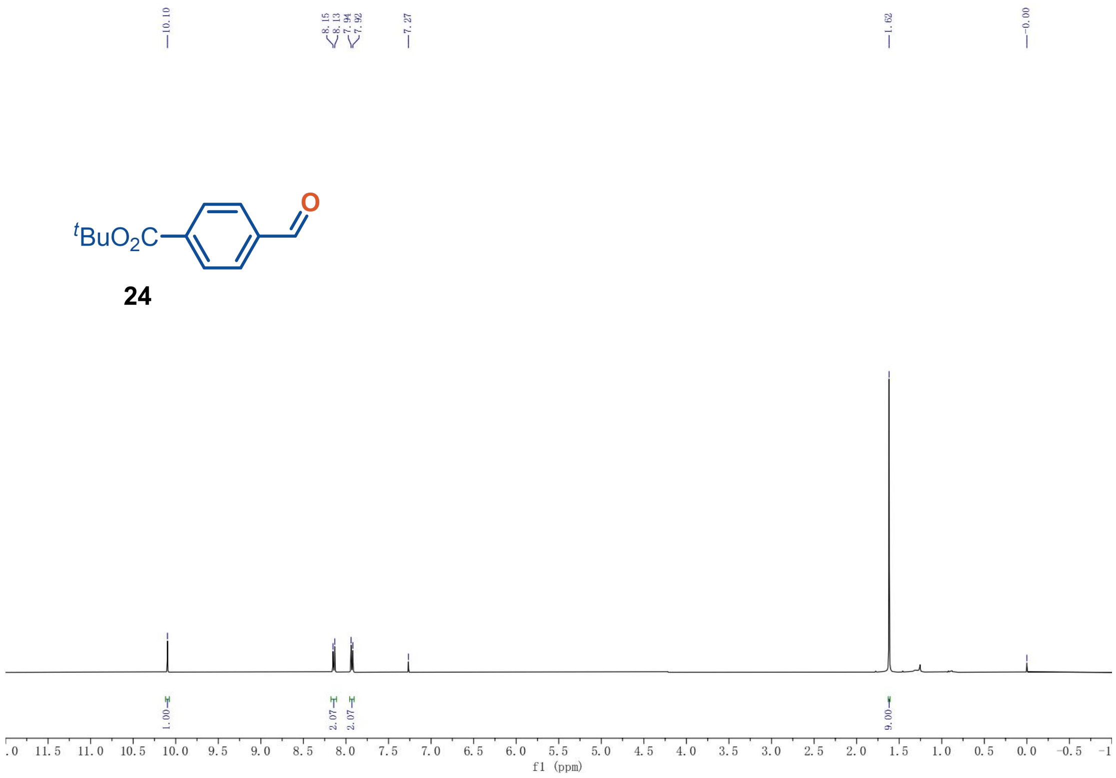

^{13}C NMR spectrum of **24**

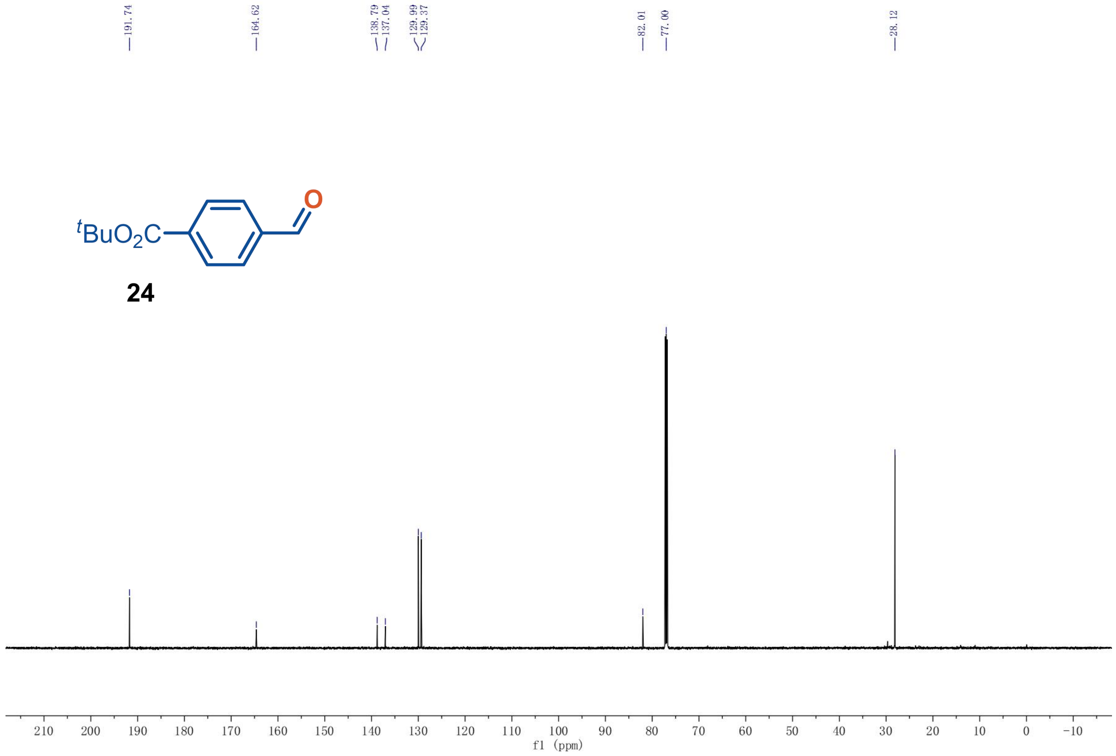

^{1}H NMR spectrum of **25**

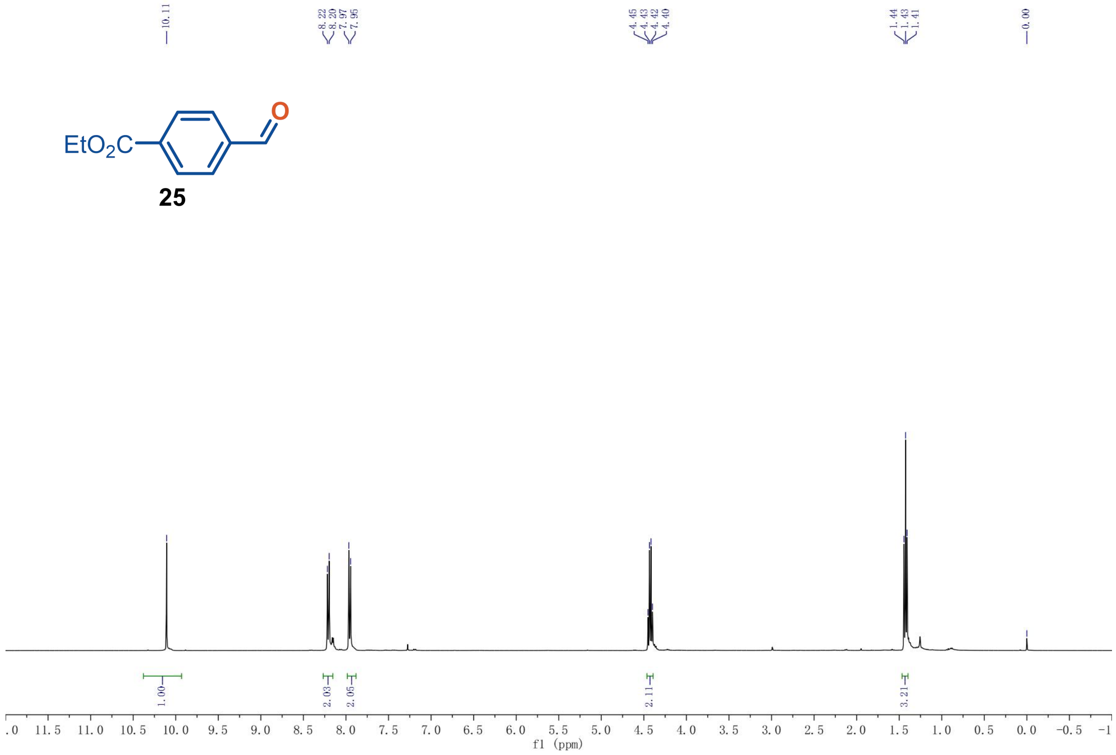

^{13}C NMR spectrum of **25**

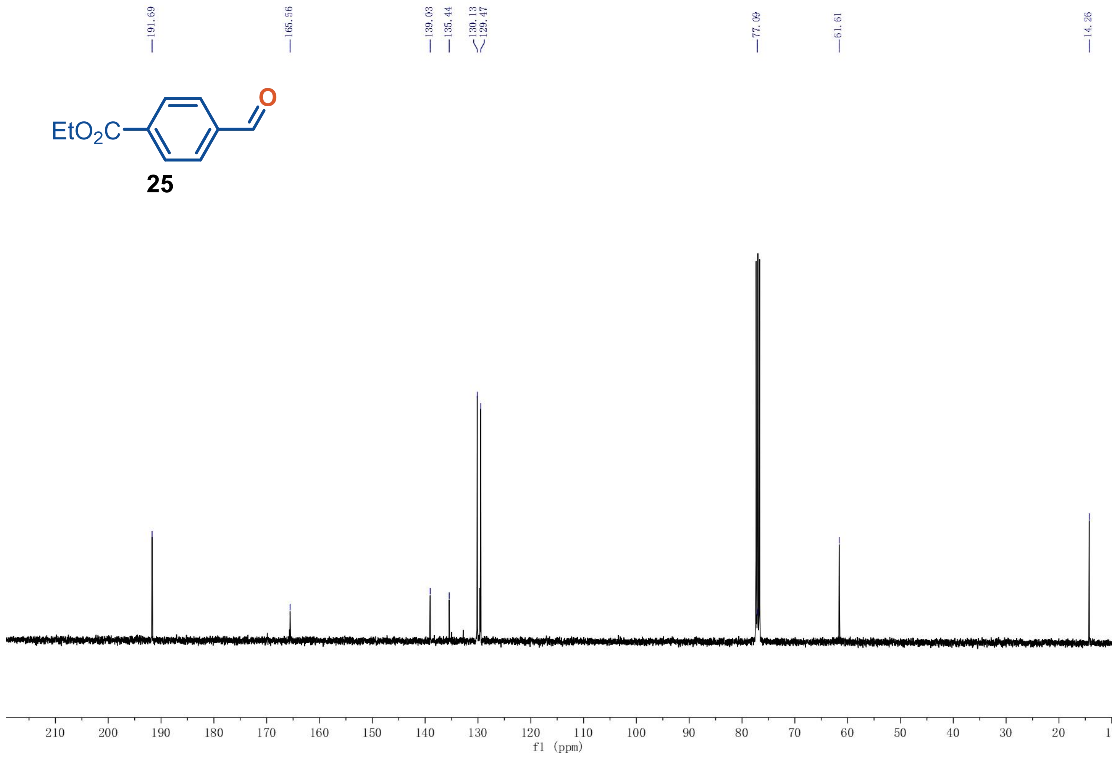

^{1}H NMR spectrum of **26**

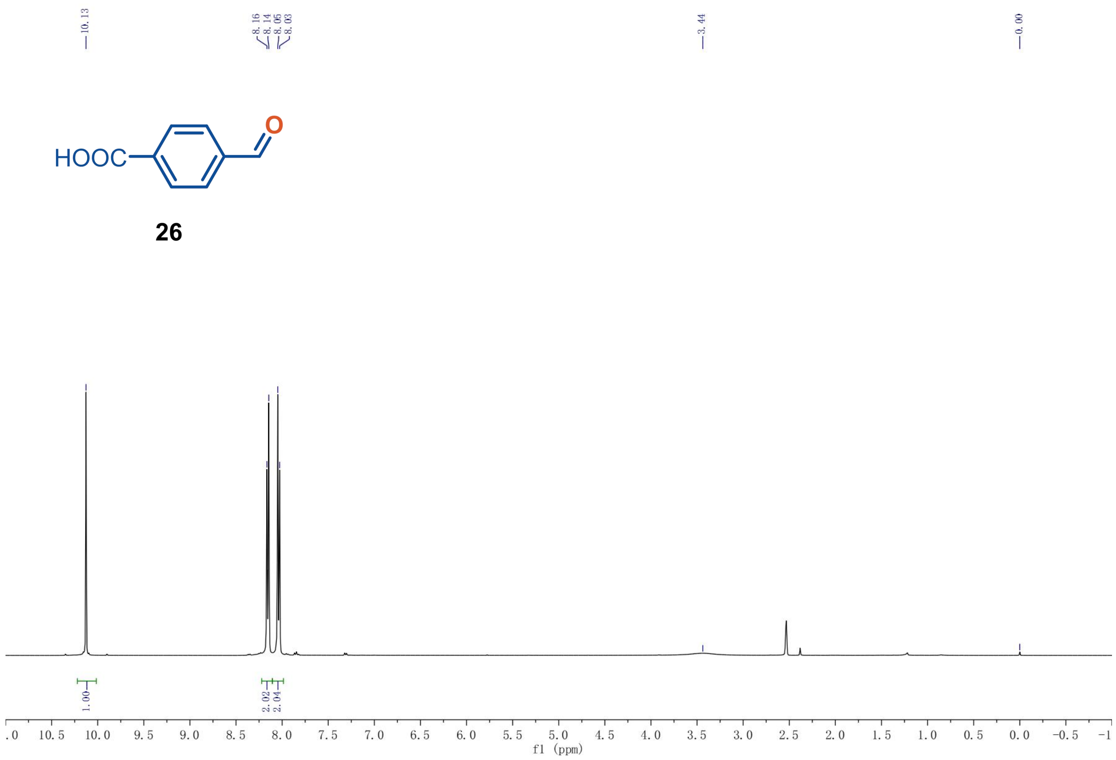

^{13}C NMR spectrum of **26**

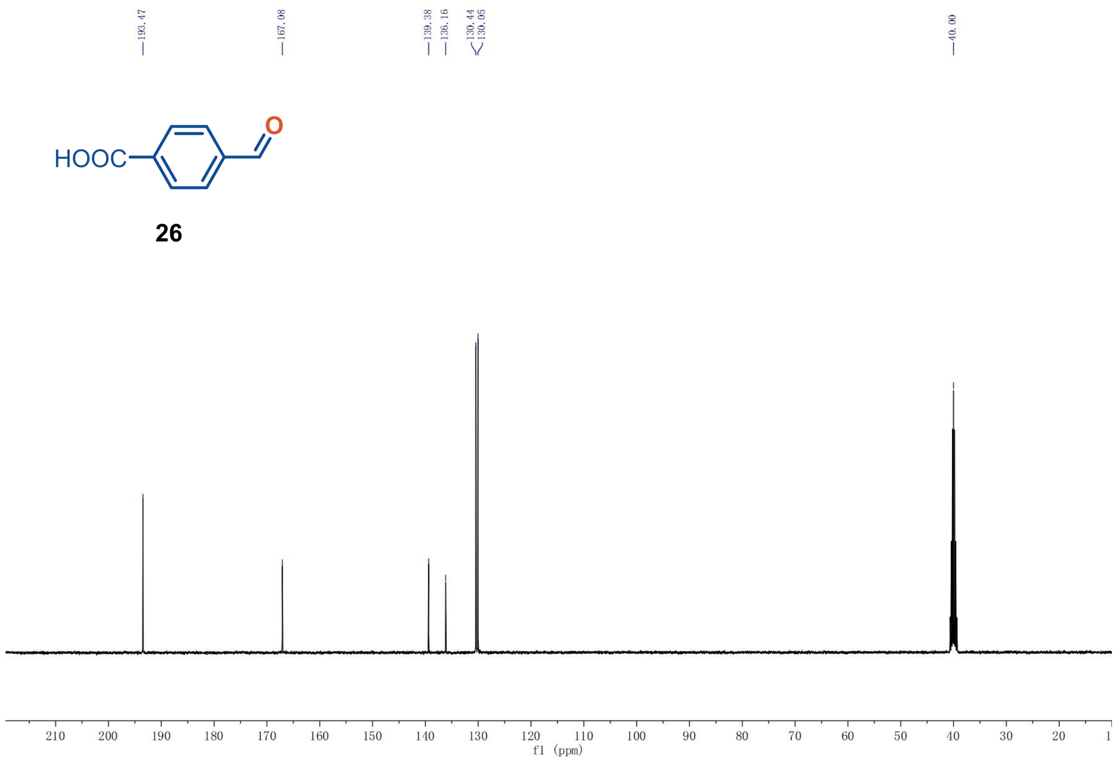

^{1}H NMR spectrum of **27**

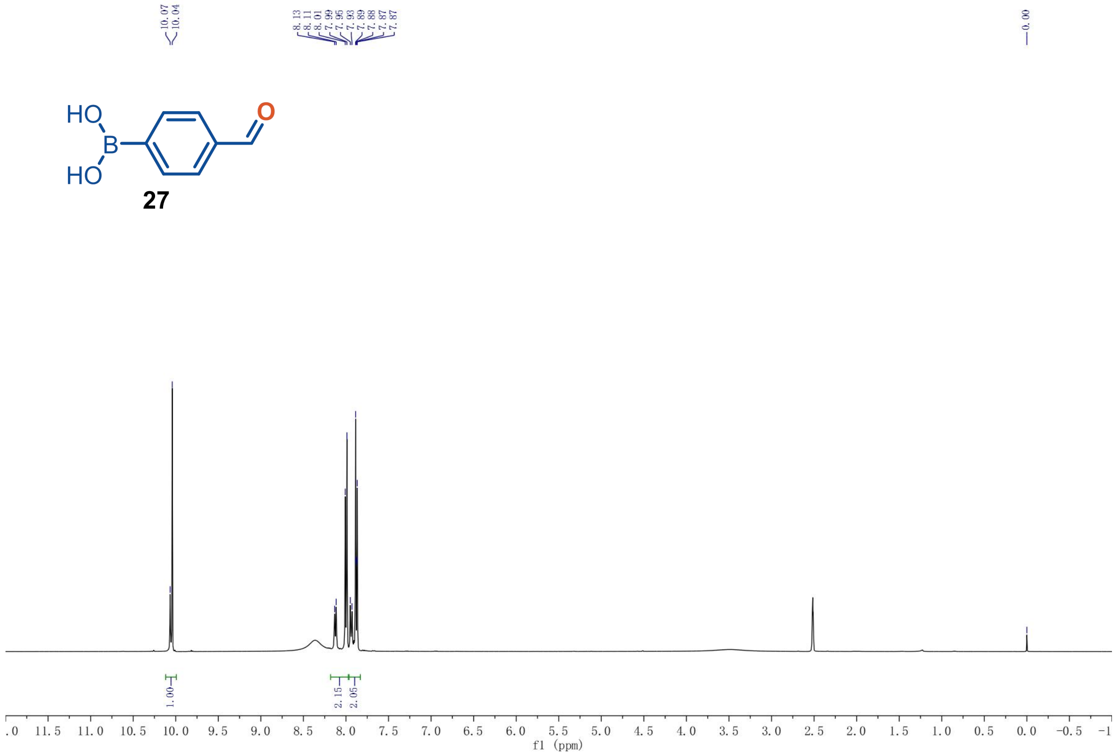

^{13}C NMR spectrum of **27**

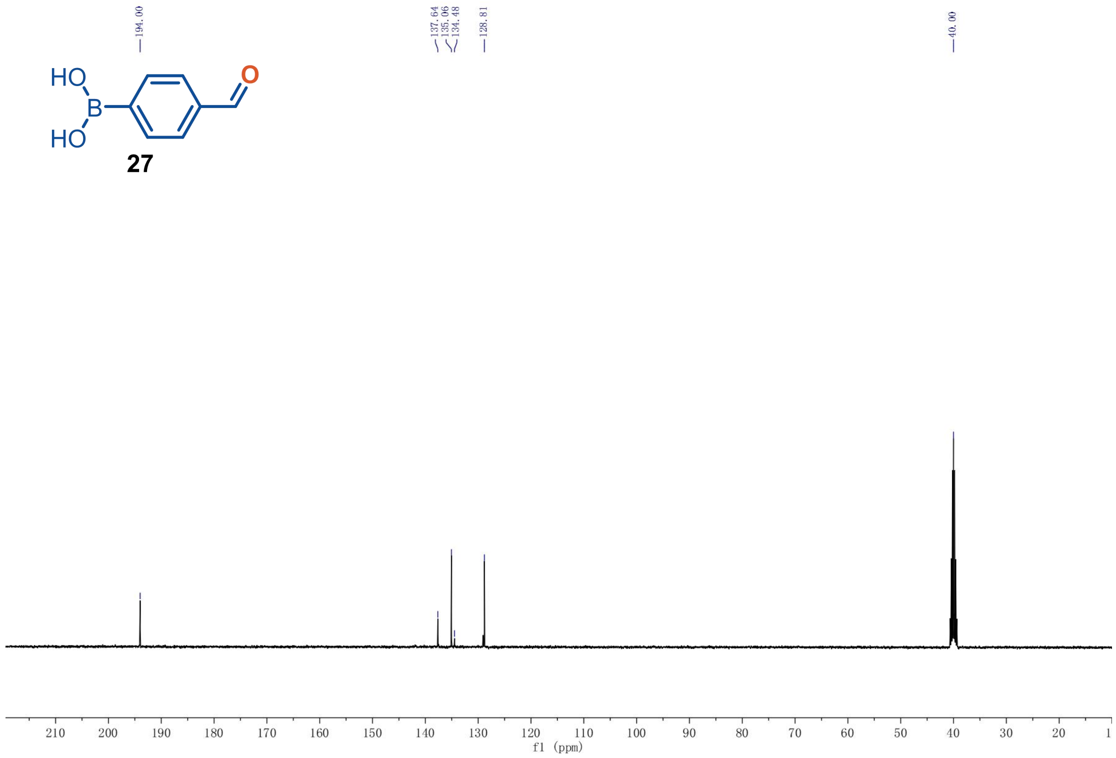

^{1}H NMR spectrum of **28**

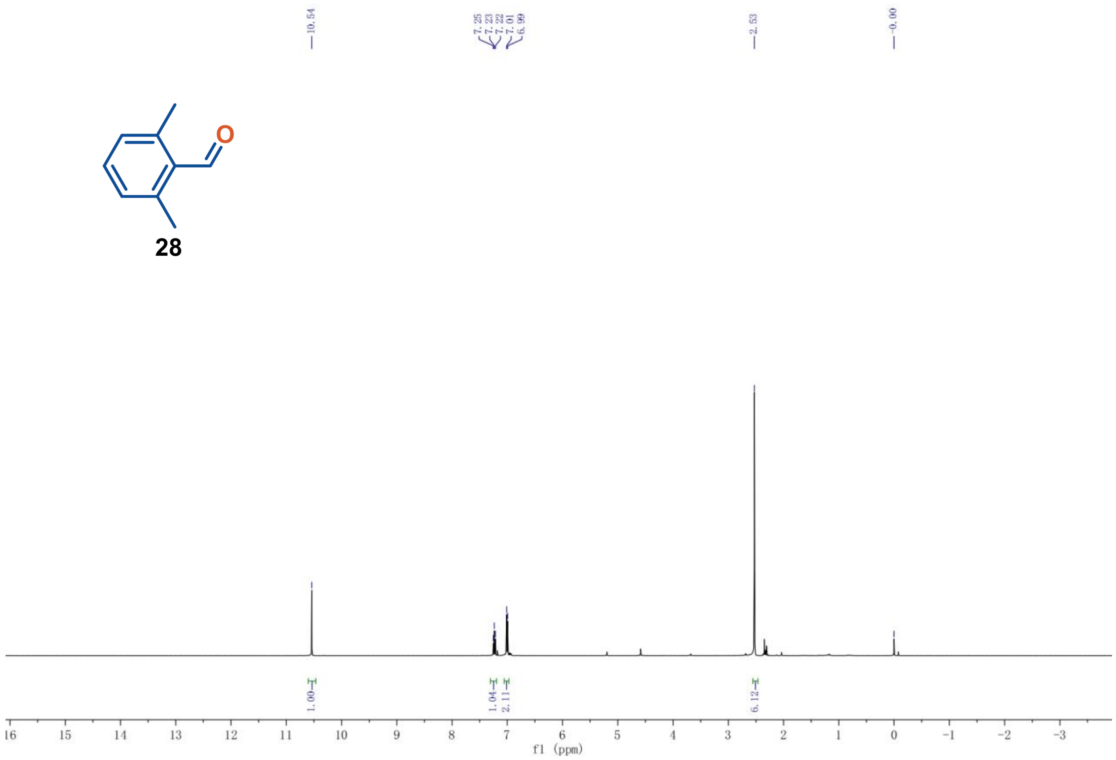

^{13}C NMR spectrum of **28**

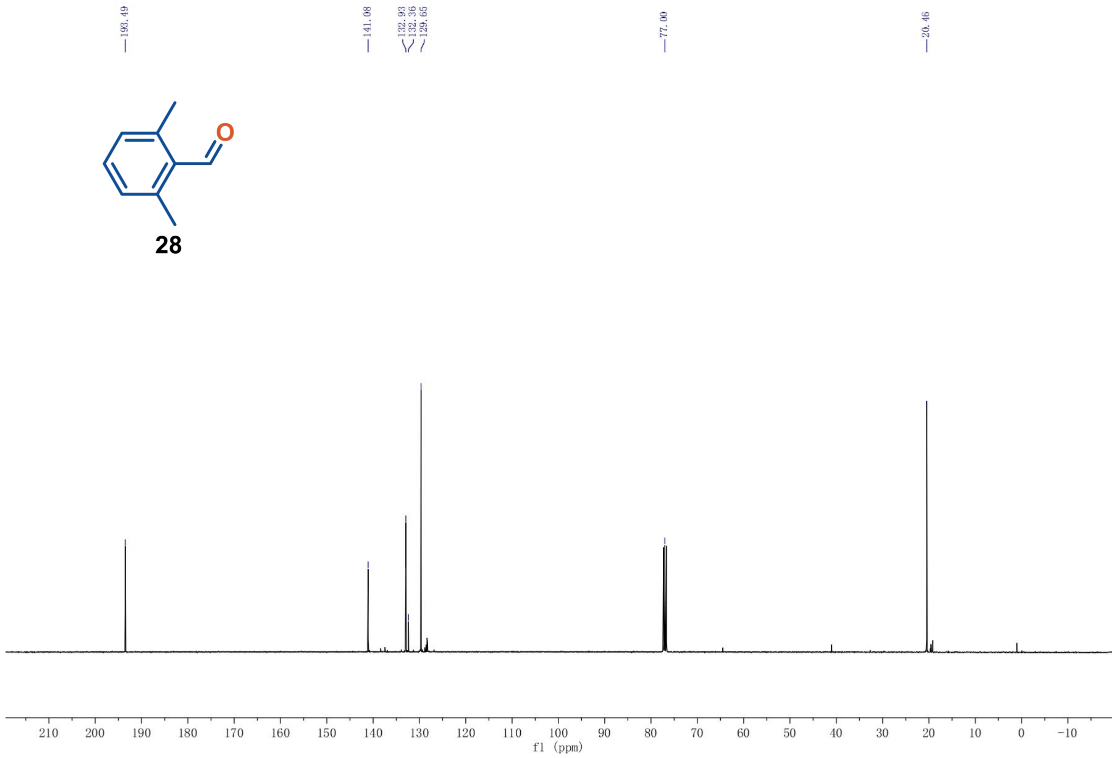

^{1}H NMR spectrum of **29**

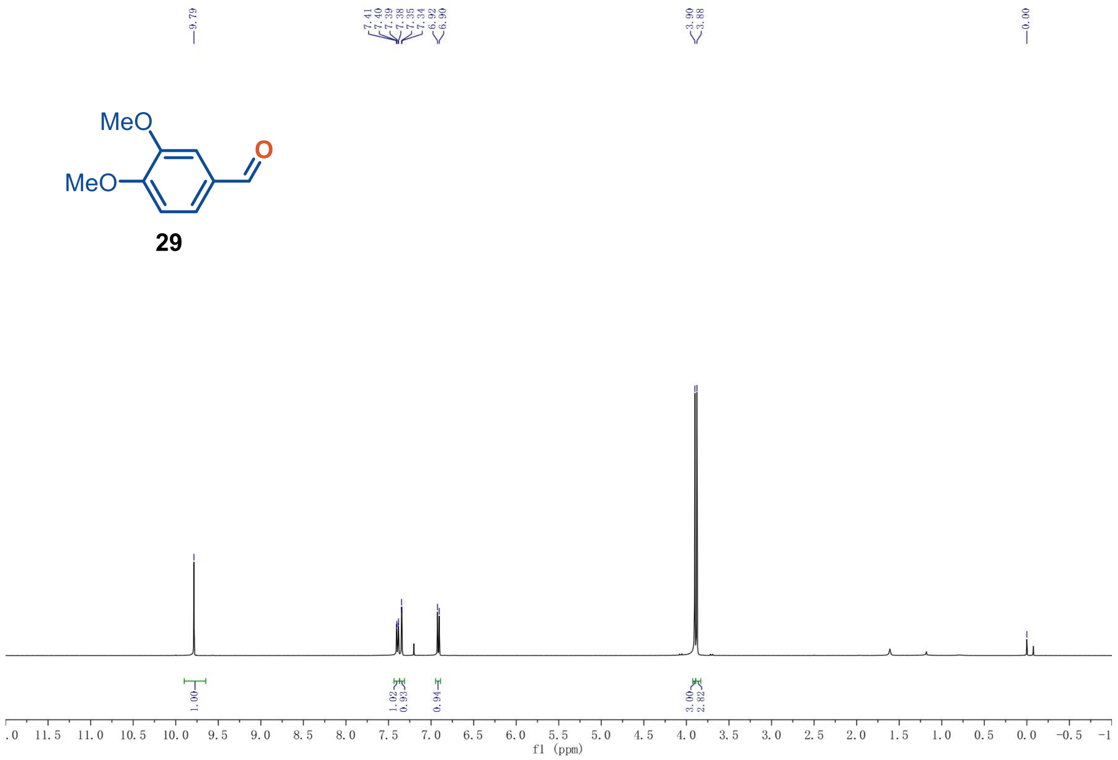

^{13}C NMR spectrum of **29**

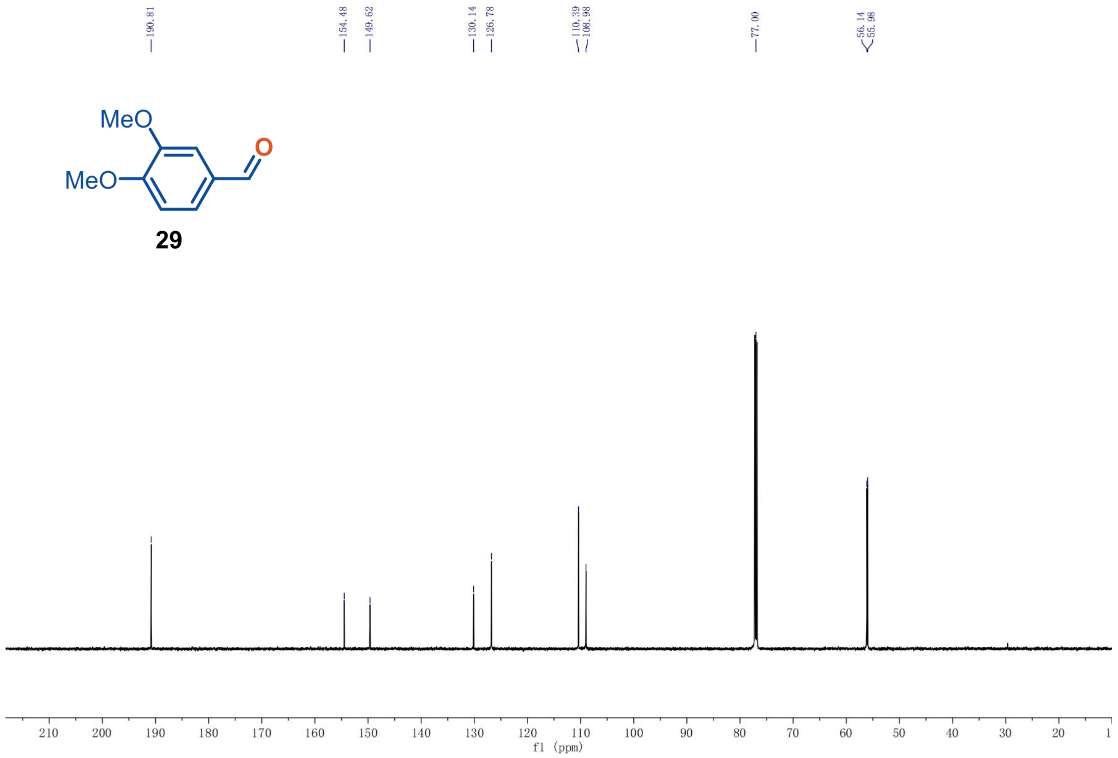

^{1}H NMR spectrum of **30**

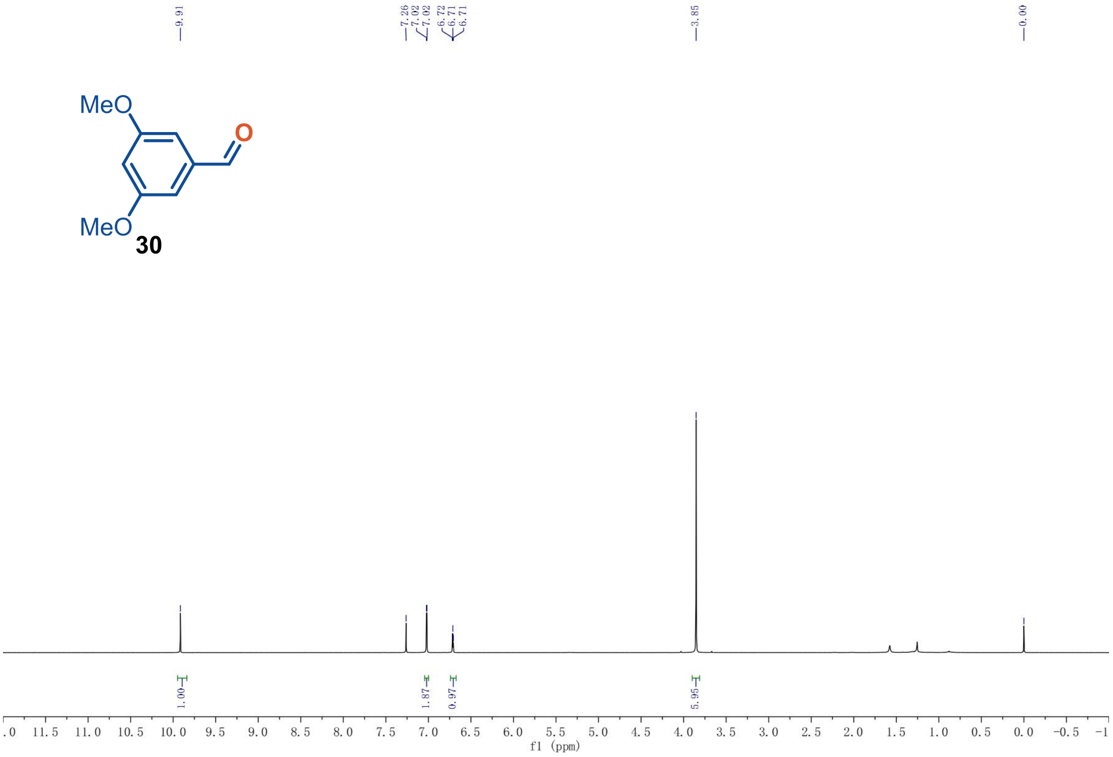

^{13}C NMR spectrum of **30**

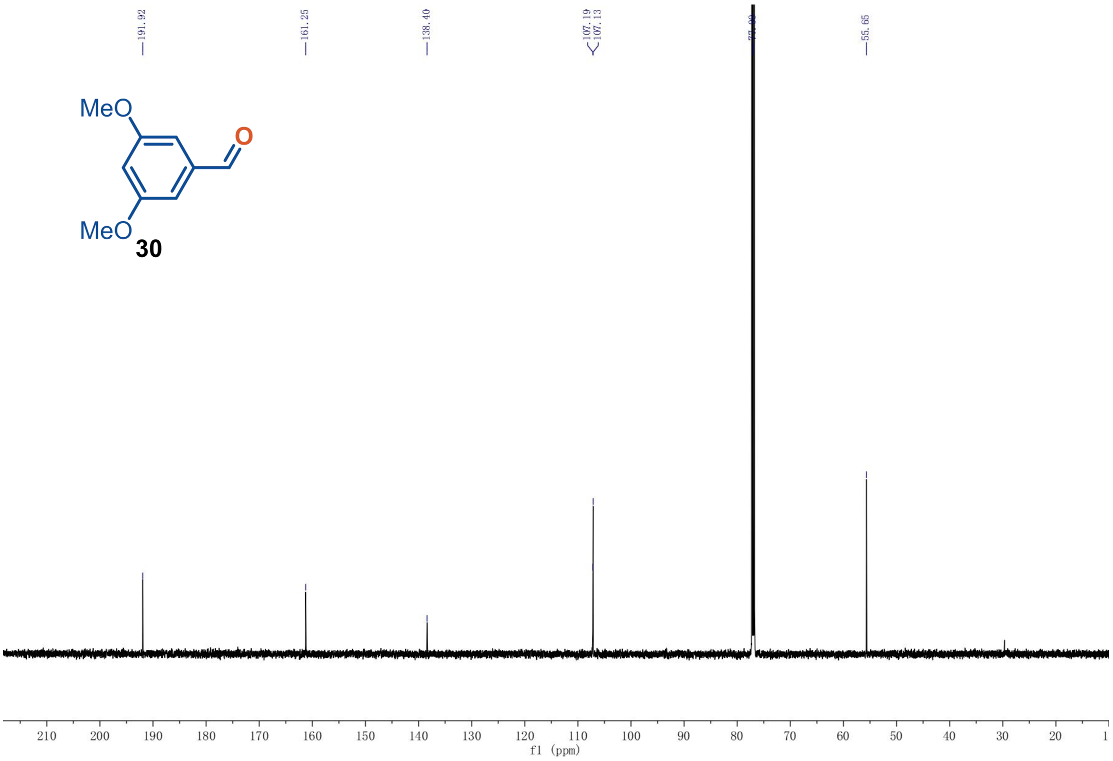

^{1}H NMR spectrum of **31**

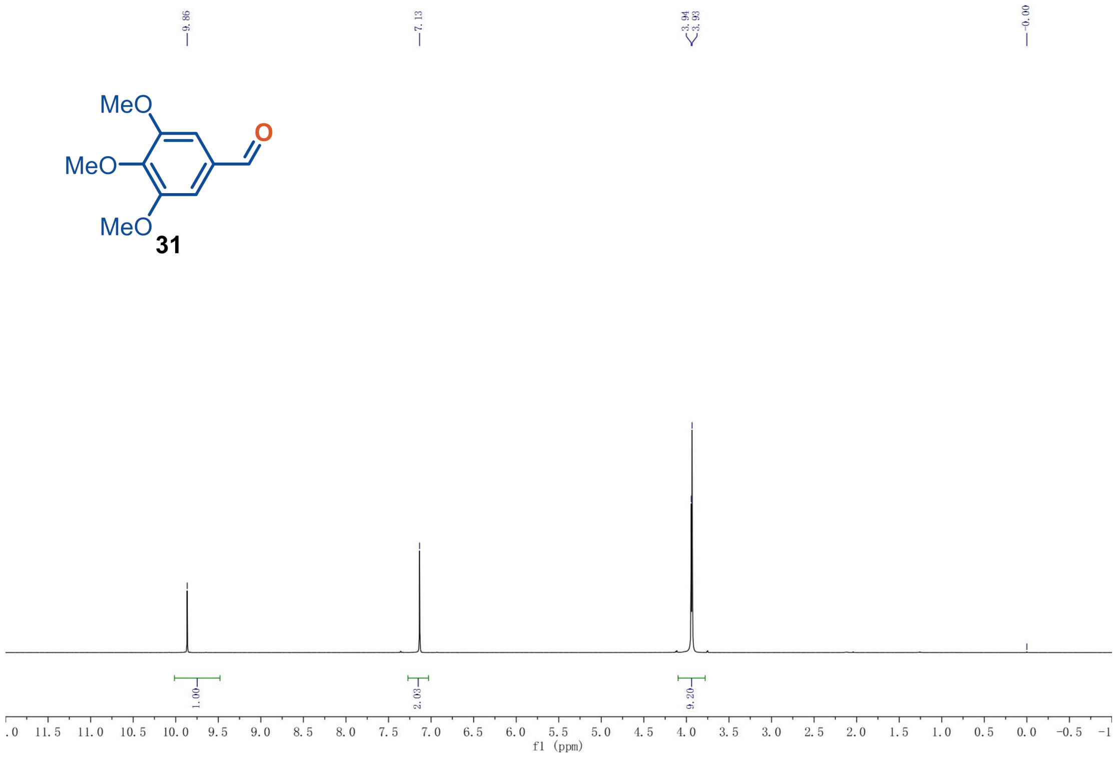

^{13}C NMR spectrum of **31**

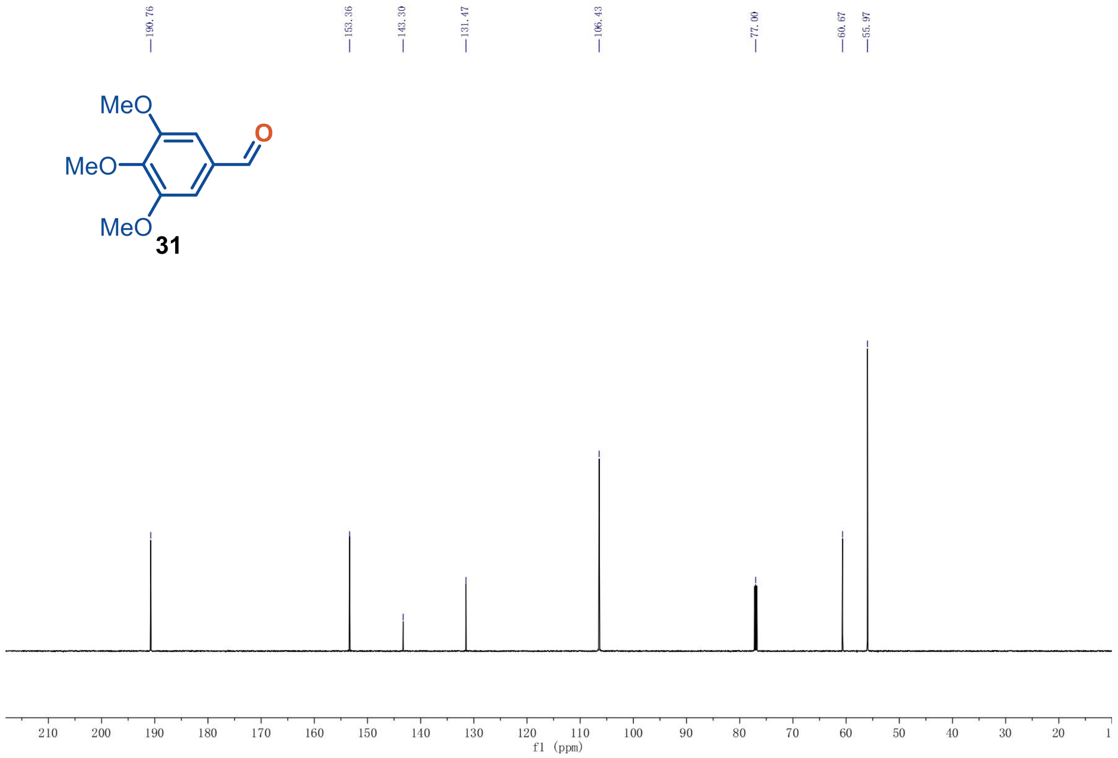

^{1}H NMR spectrum of **32**

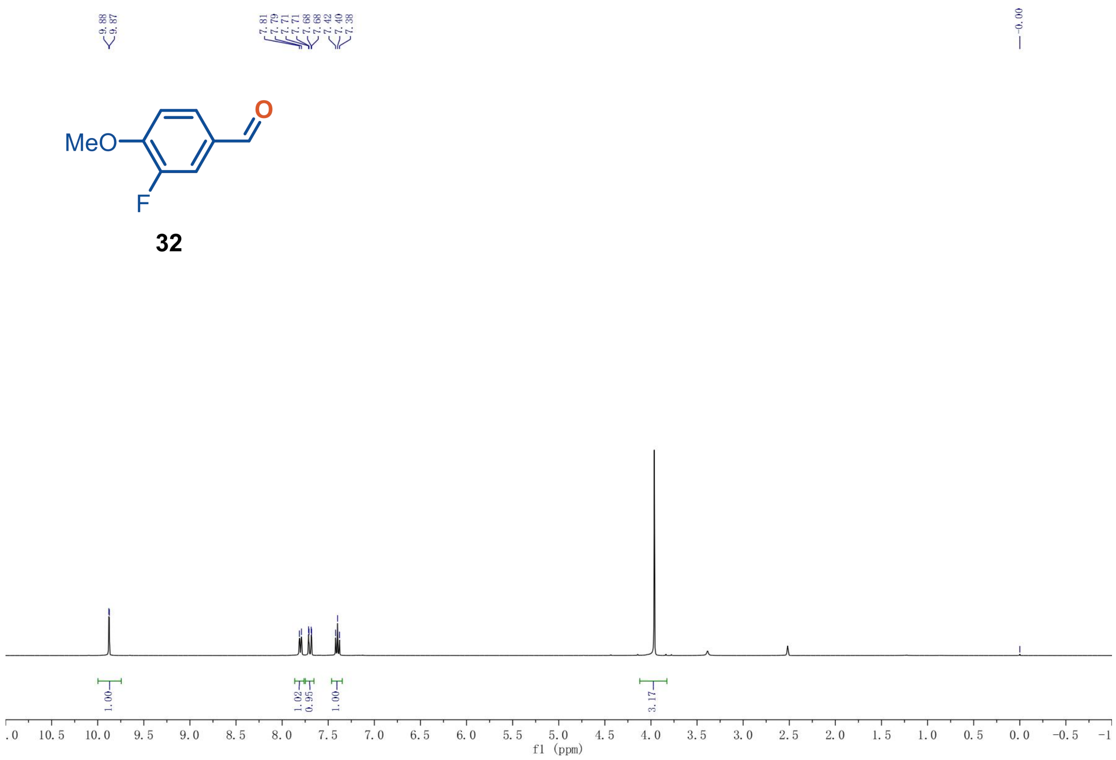

^{13}C NMR spectrum of **32**

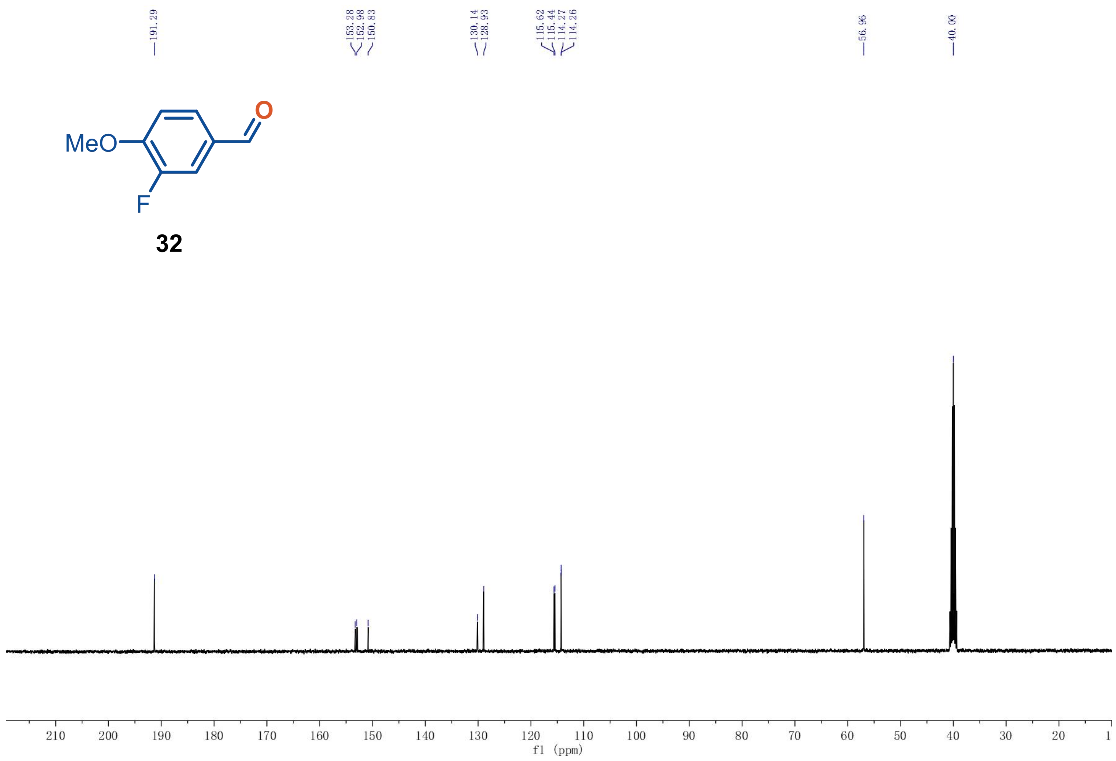

^{19}F NMR spectrum of **32**

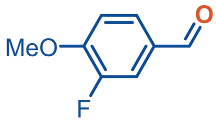

32

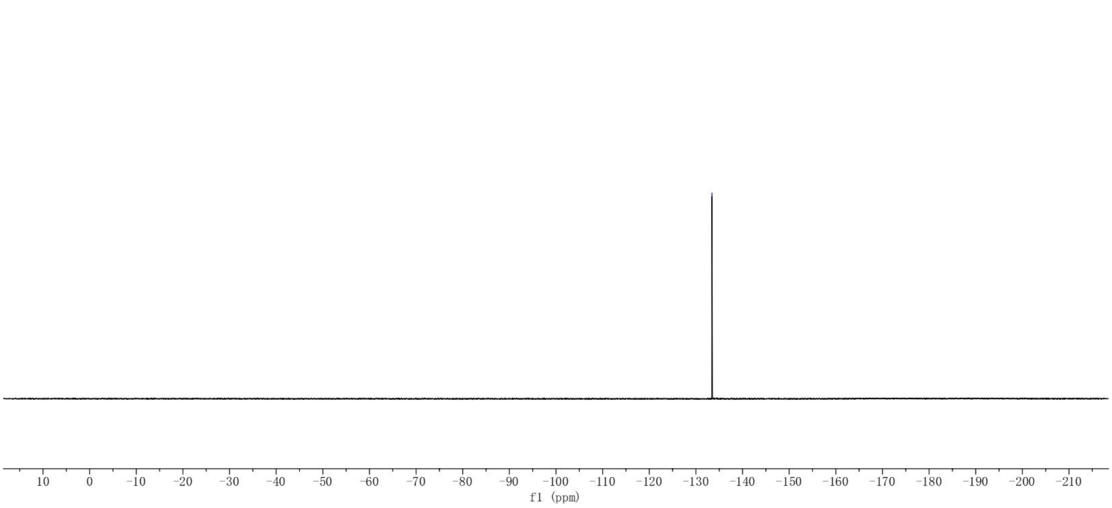

^{1}H NMR spectrum of **33**

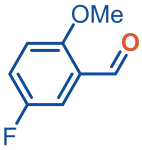

33

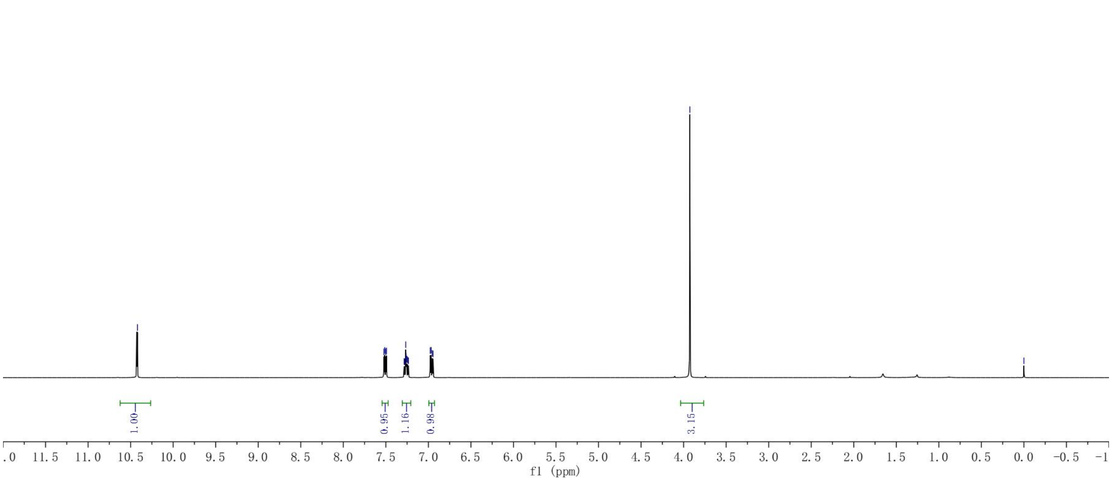

^{13}C NMR spectrum of **33**

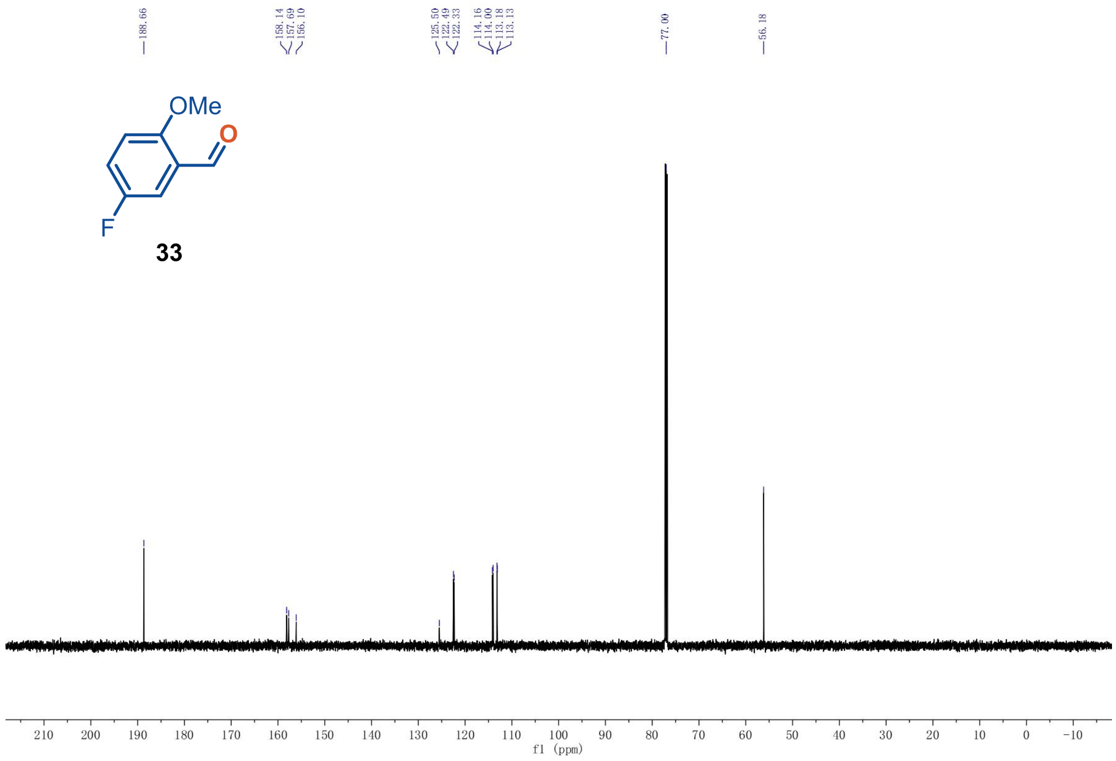

^{19}F NMR spectrum of **33**

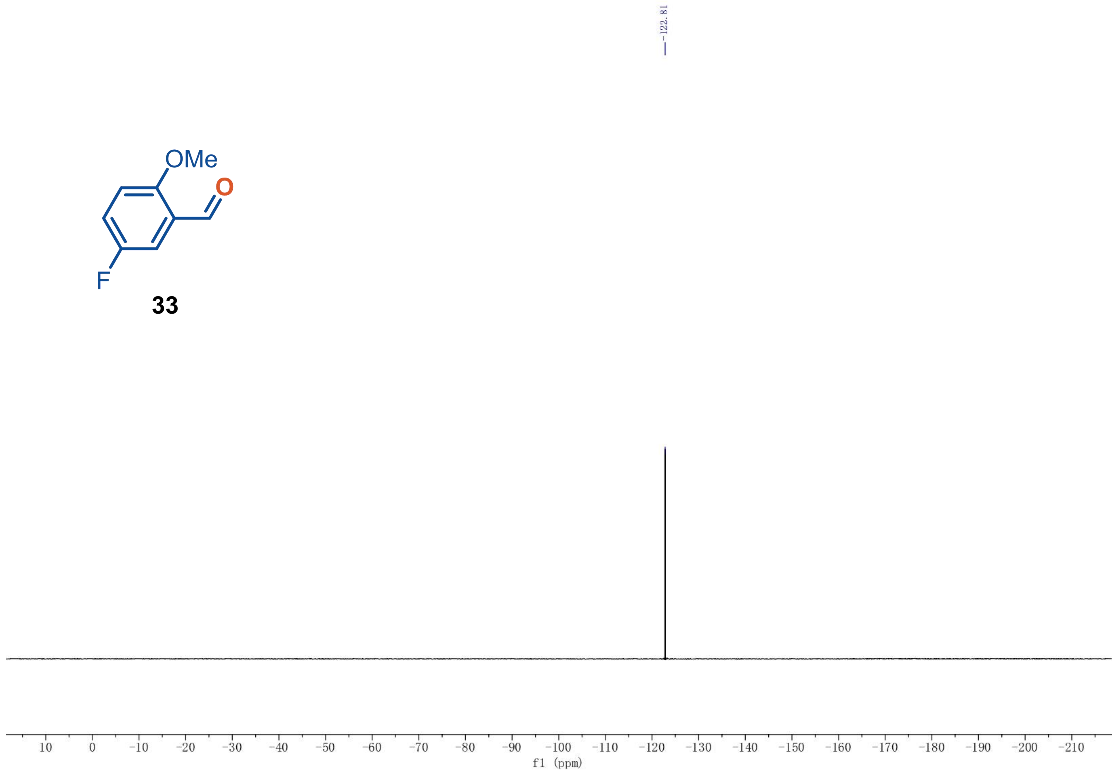

^{1}H NMR spectrum of **34**

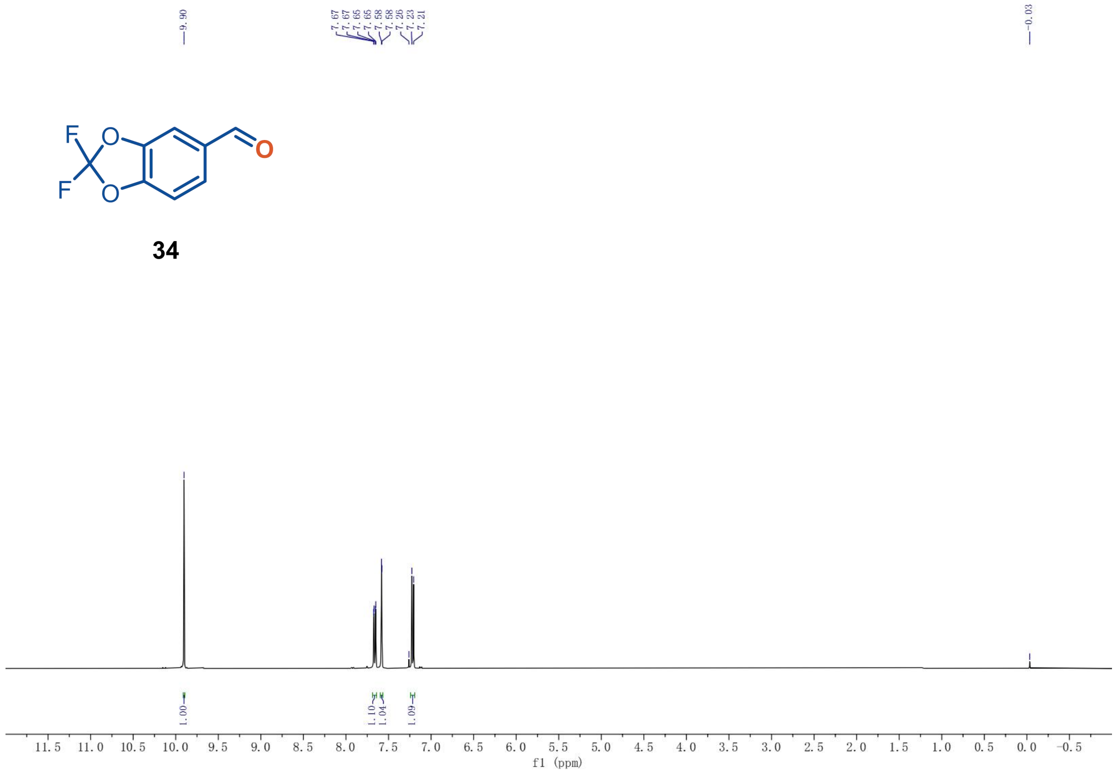

^{13}C NMR spectrum of **34**

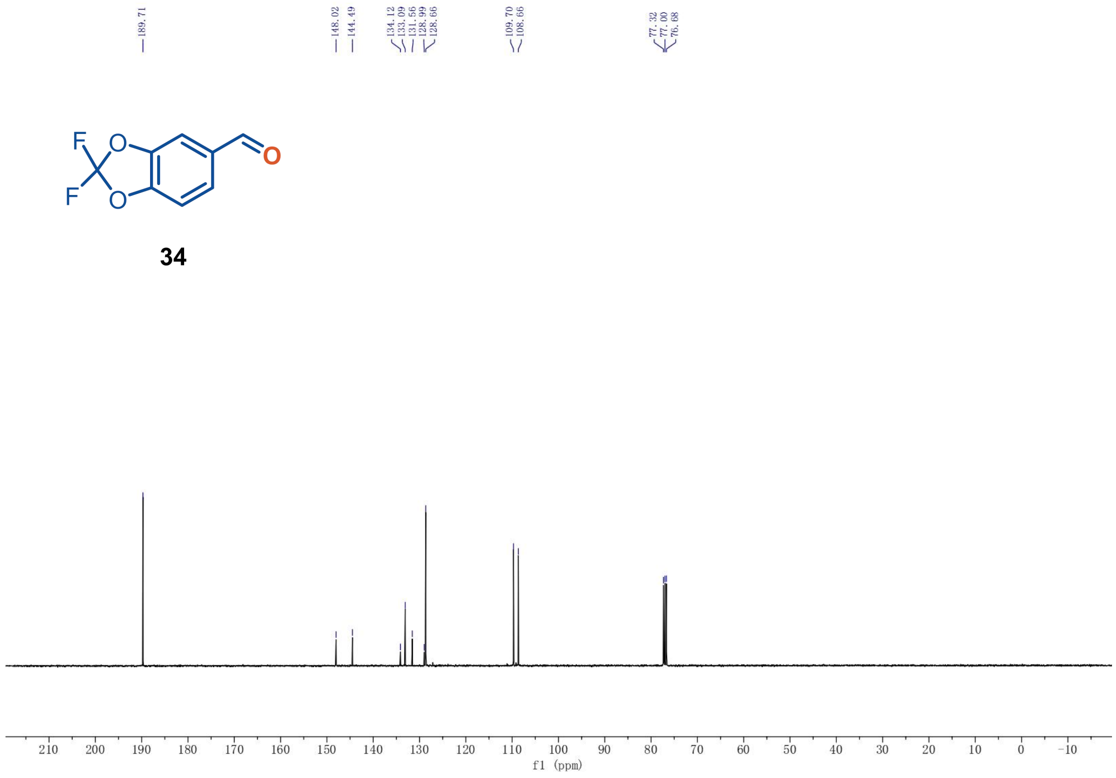

^{19}F NMR spectrum of **34**

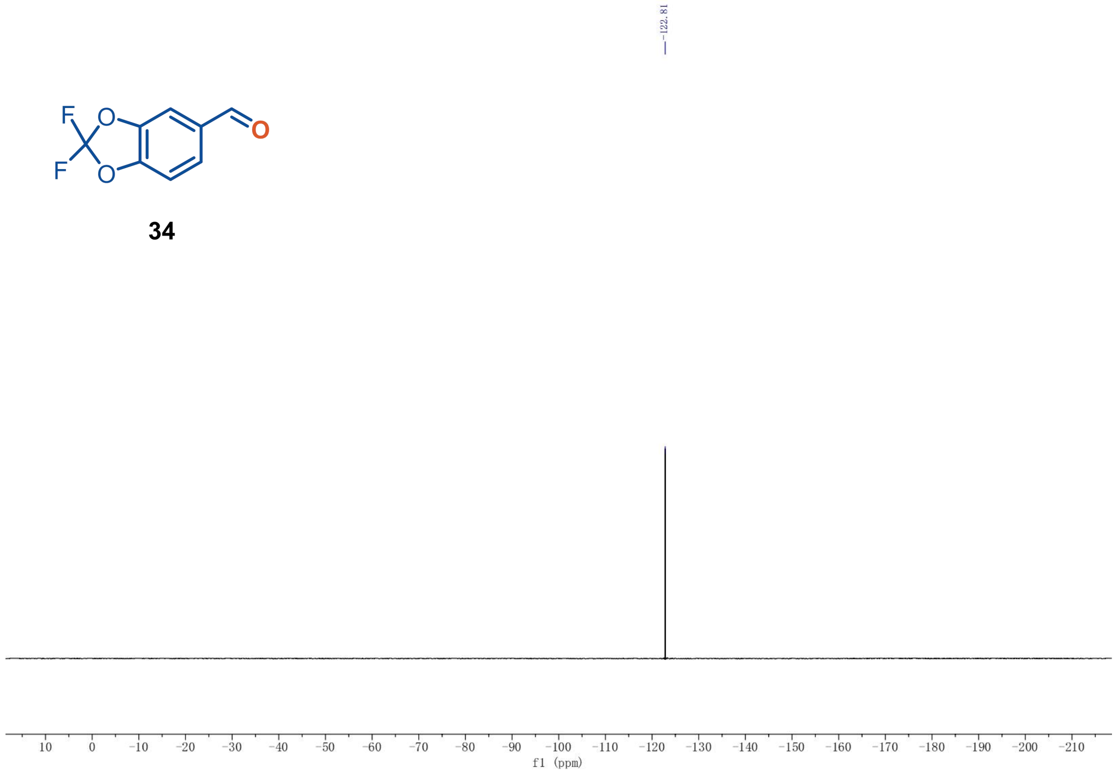

^{1}H NMR spectrum of **35**

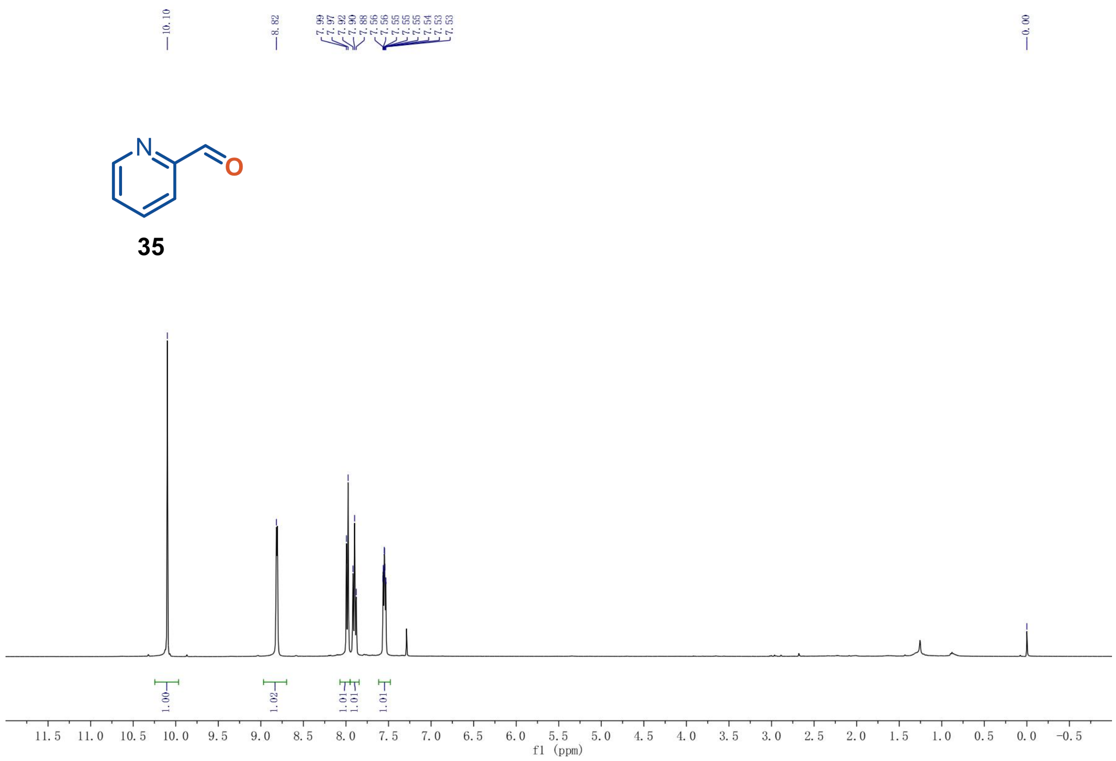

^{13}C NMR spectrum of **35**

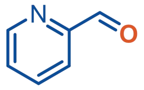

35

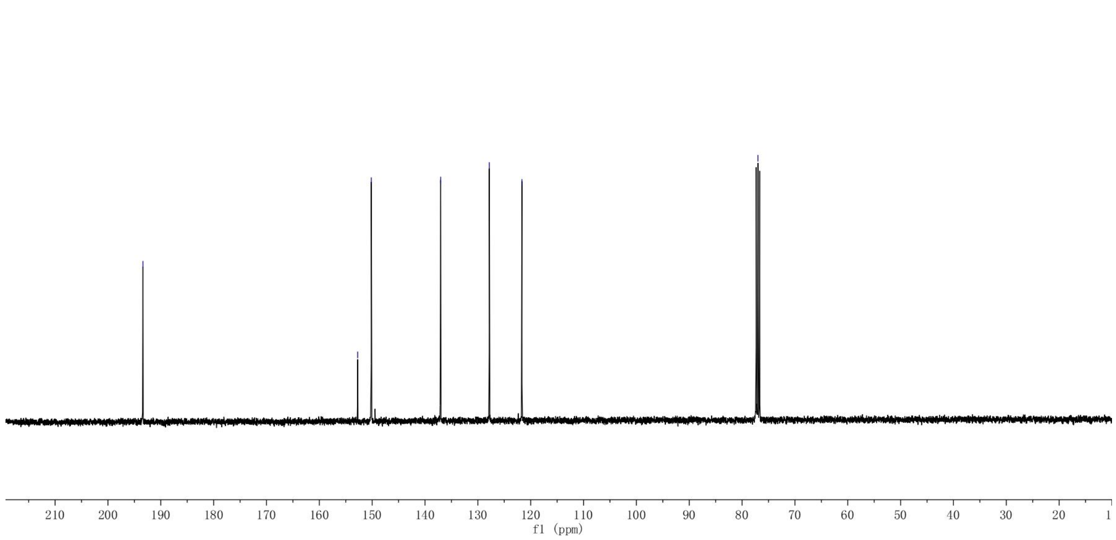

^{1}H NMR spectrum of **36**

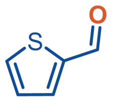

36

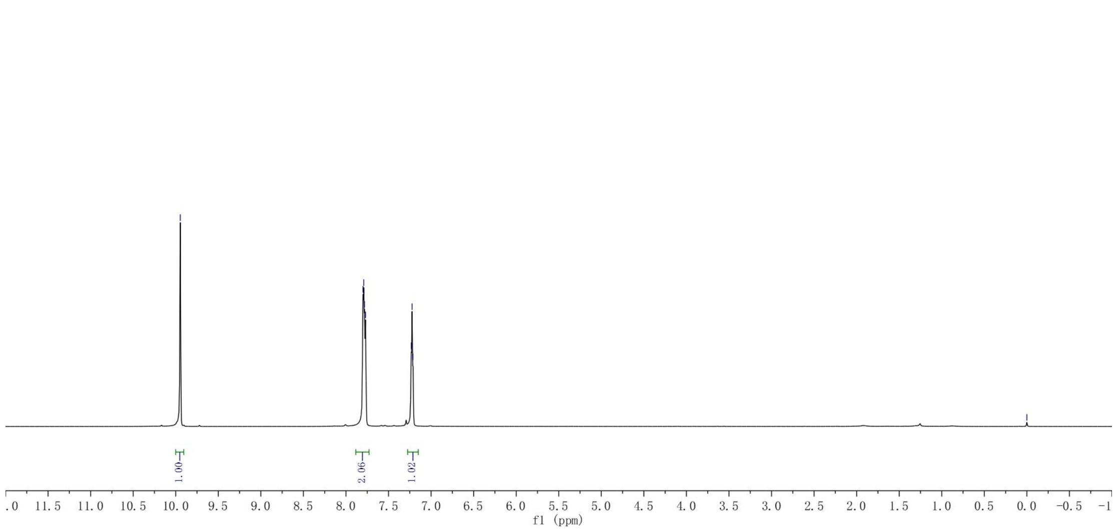

^{13}C NMR spectrum of **36**

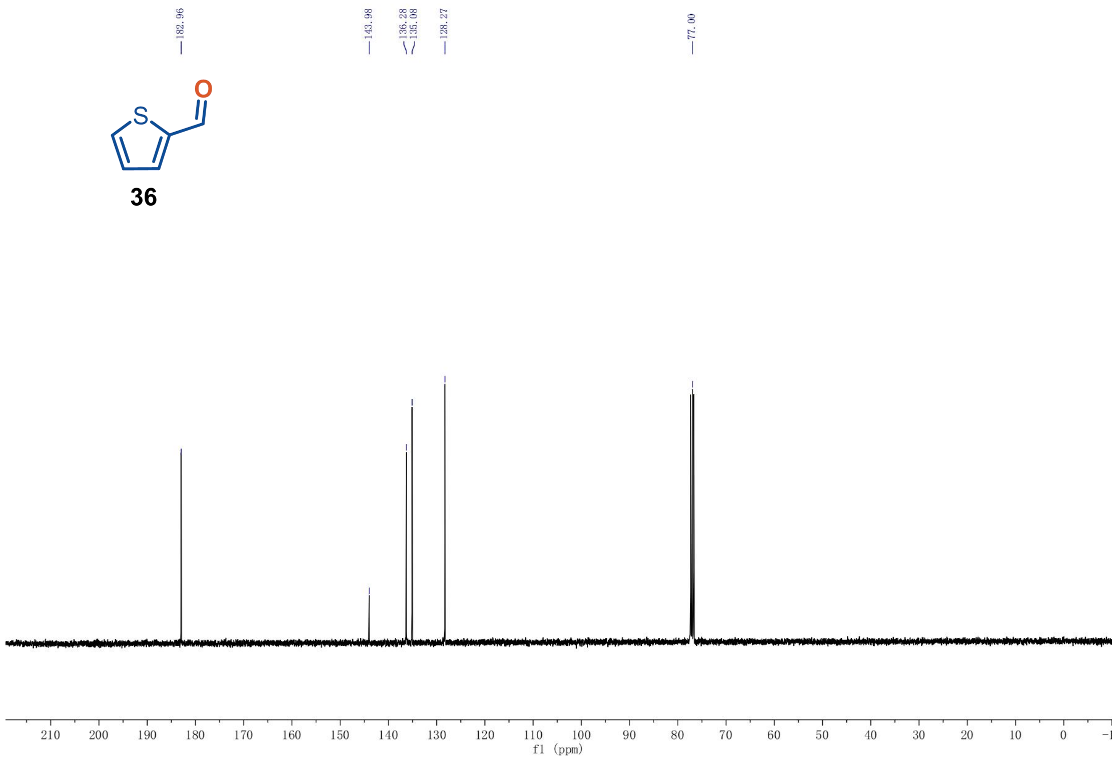

^{1}H NMR spectrum of **37**

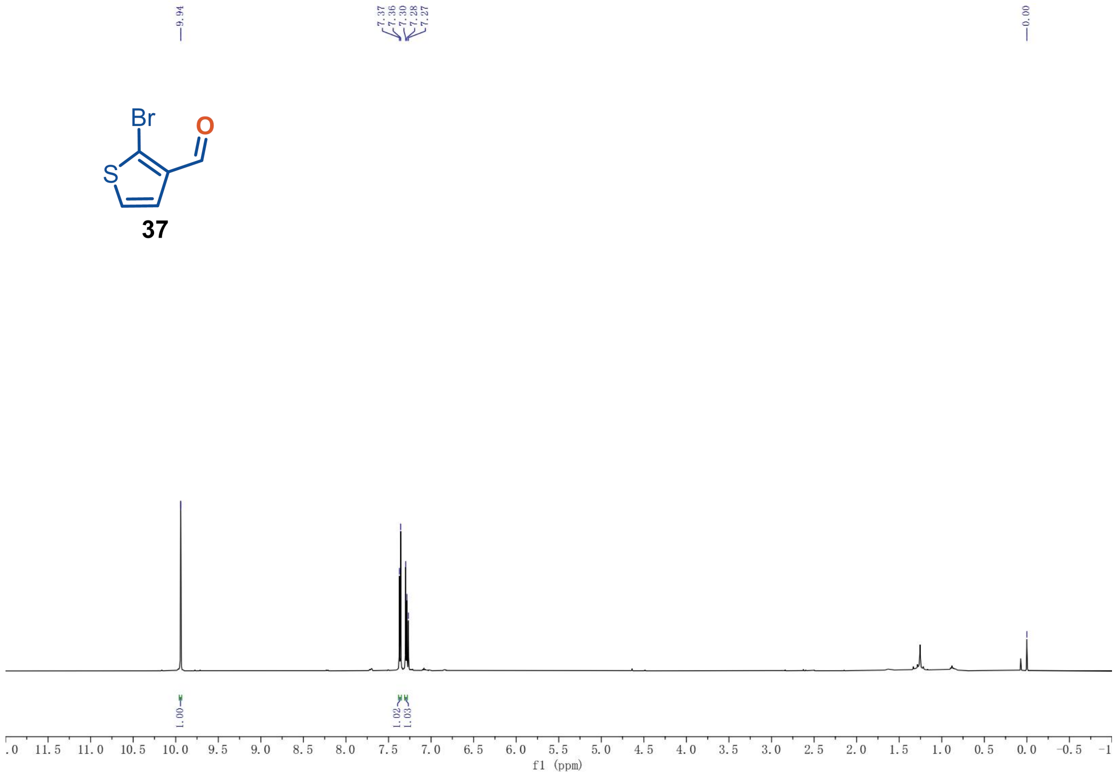

^{13}C NMR spectrum of **37**

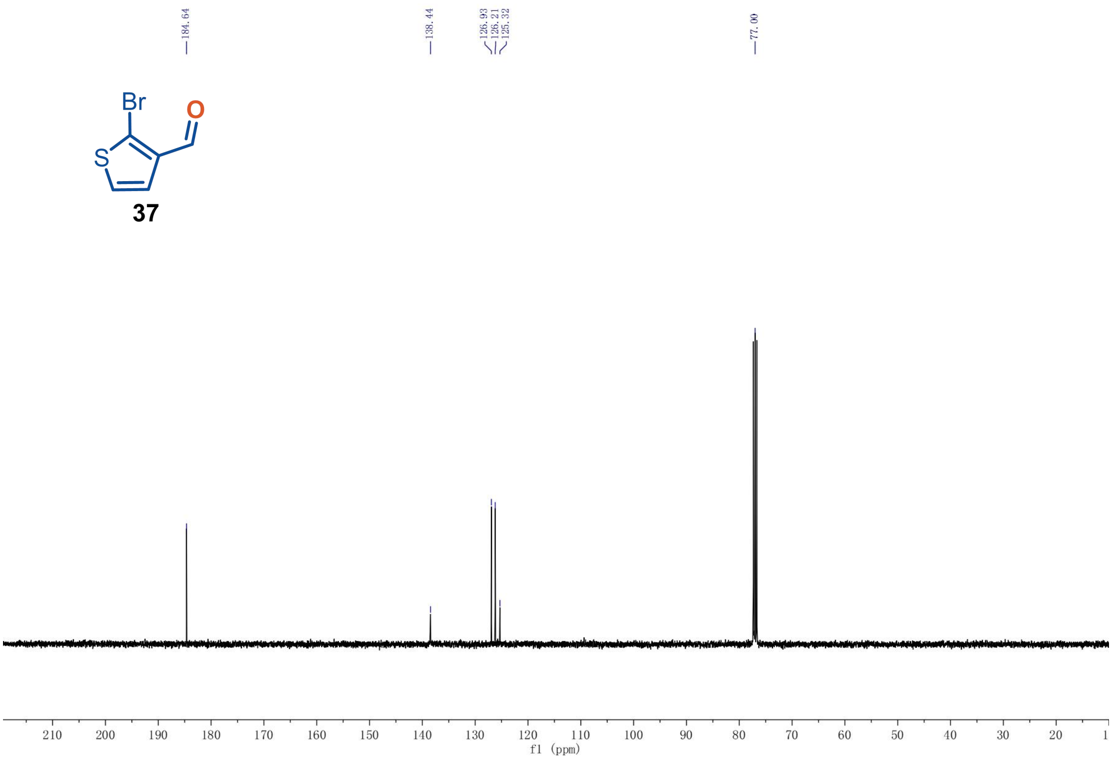

^{1}H NMR spectrum of **38**

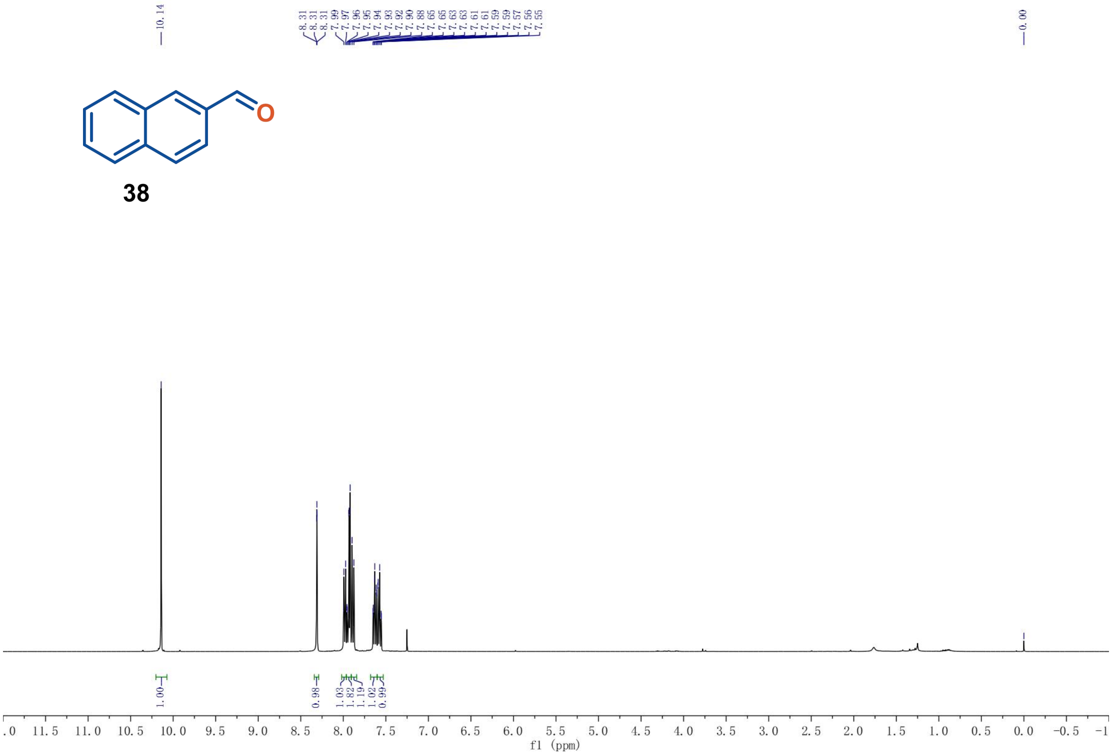

^{13}C NMR spectrum of **38**

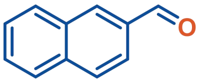

38

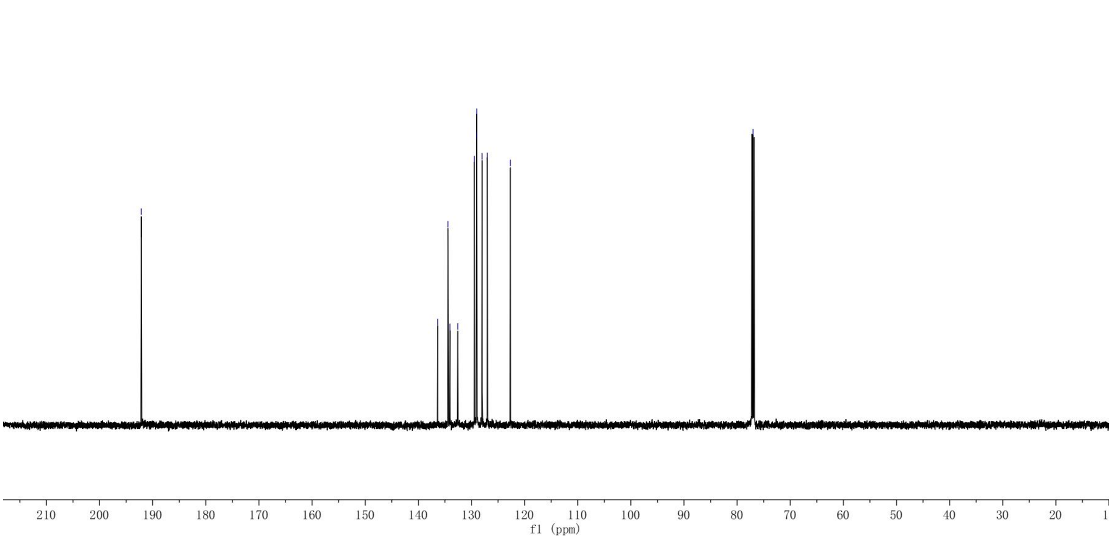

^{1}H NMR spectrum of **39**

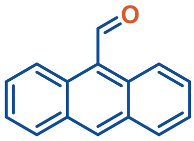

39

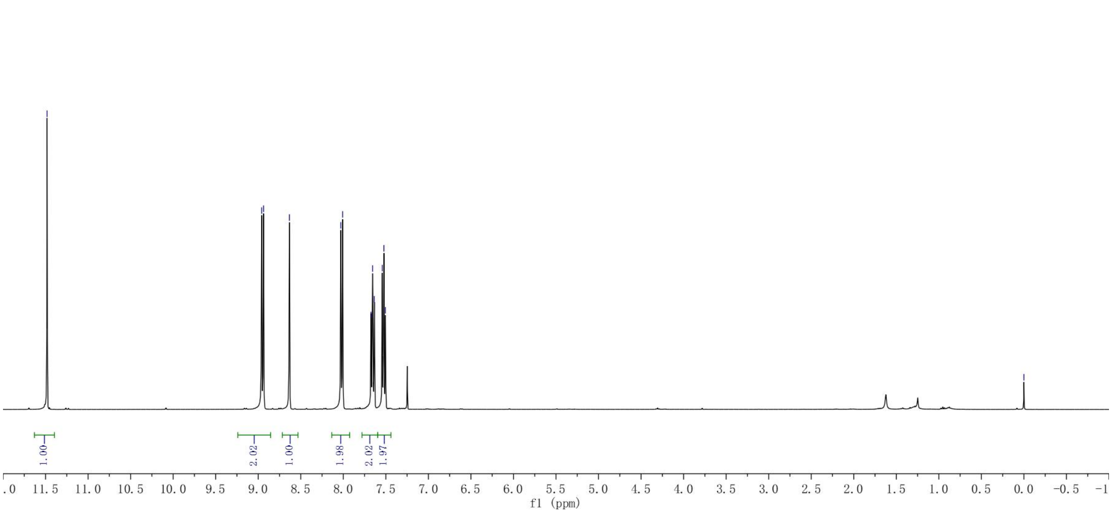

^{13}C NMR spectrum of **39**

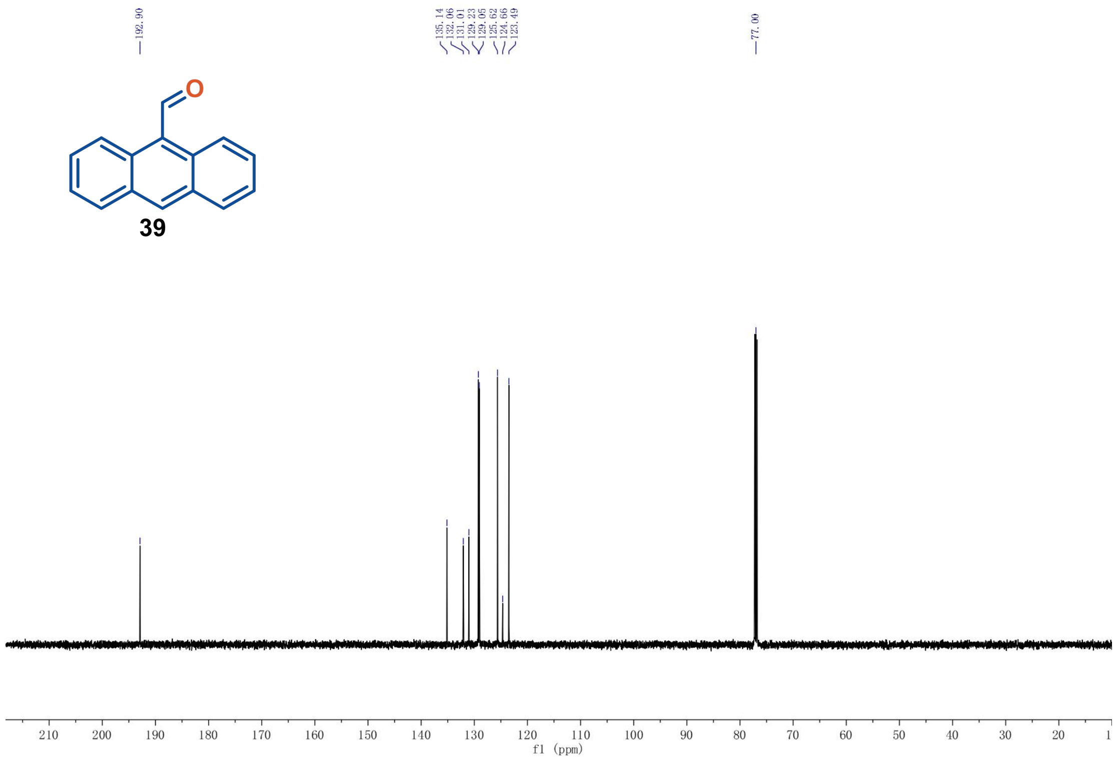

^{1}H NMR spectrum of **40**

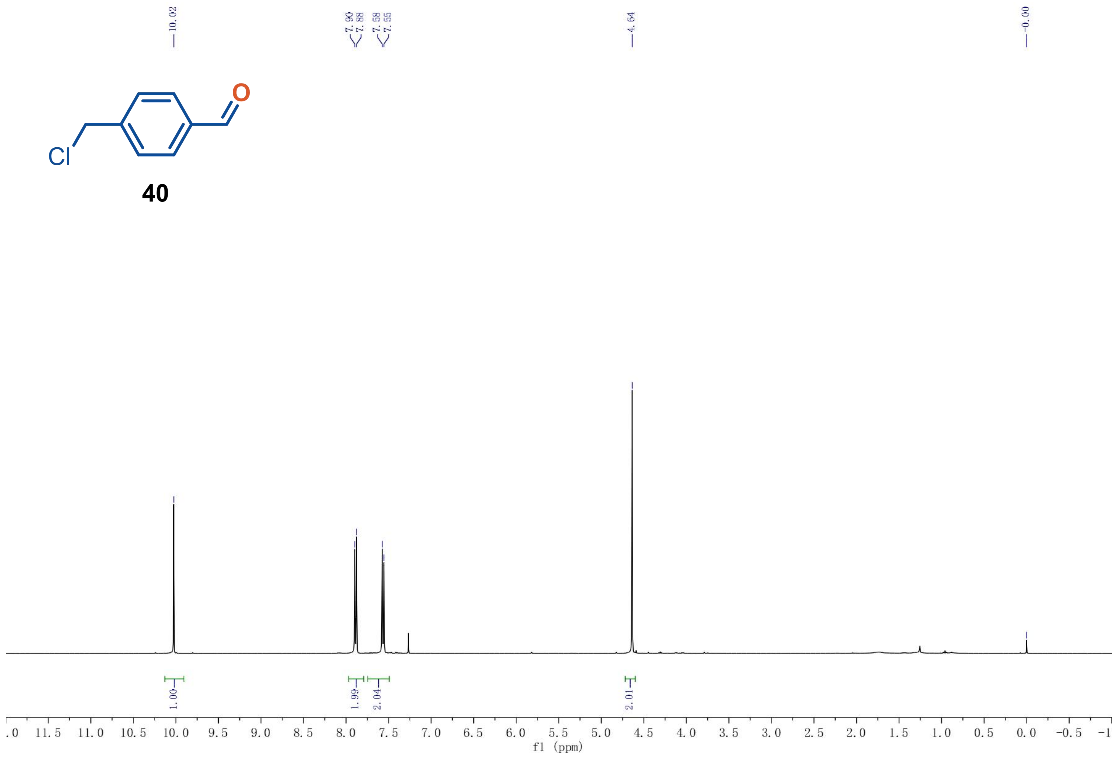

^{13}C NMR spectrum of **40**

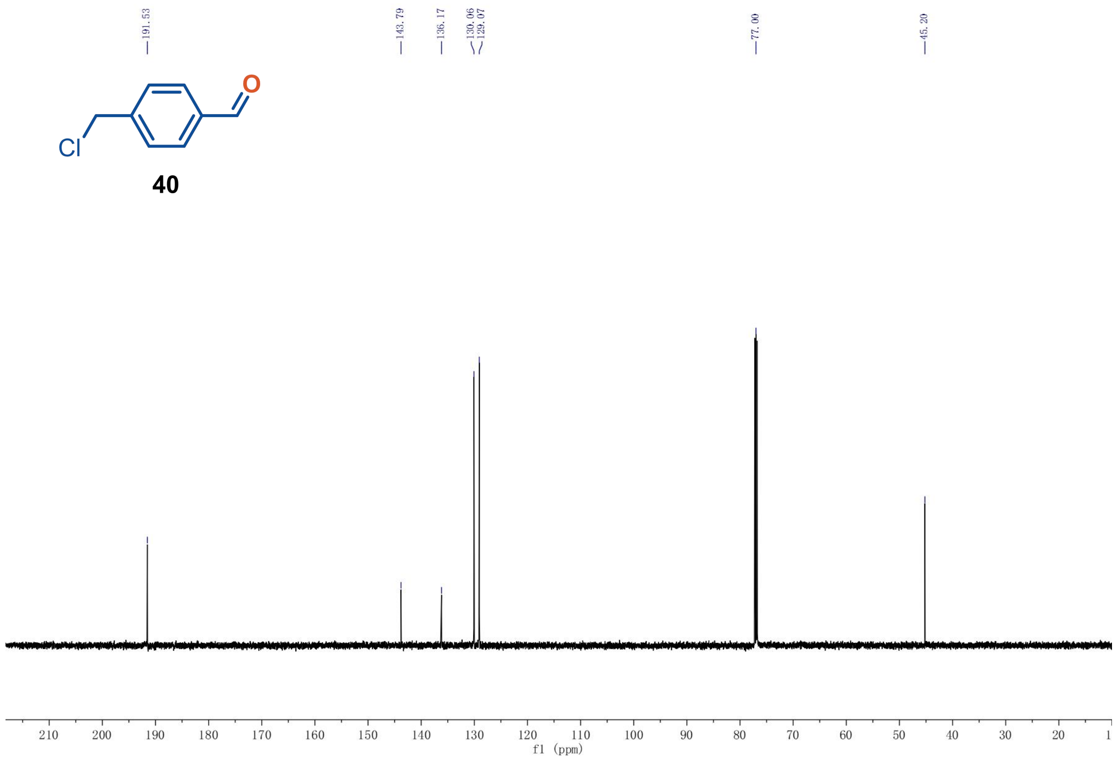

^{1}H NMR spectrum of **41**

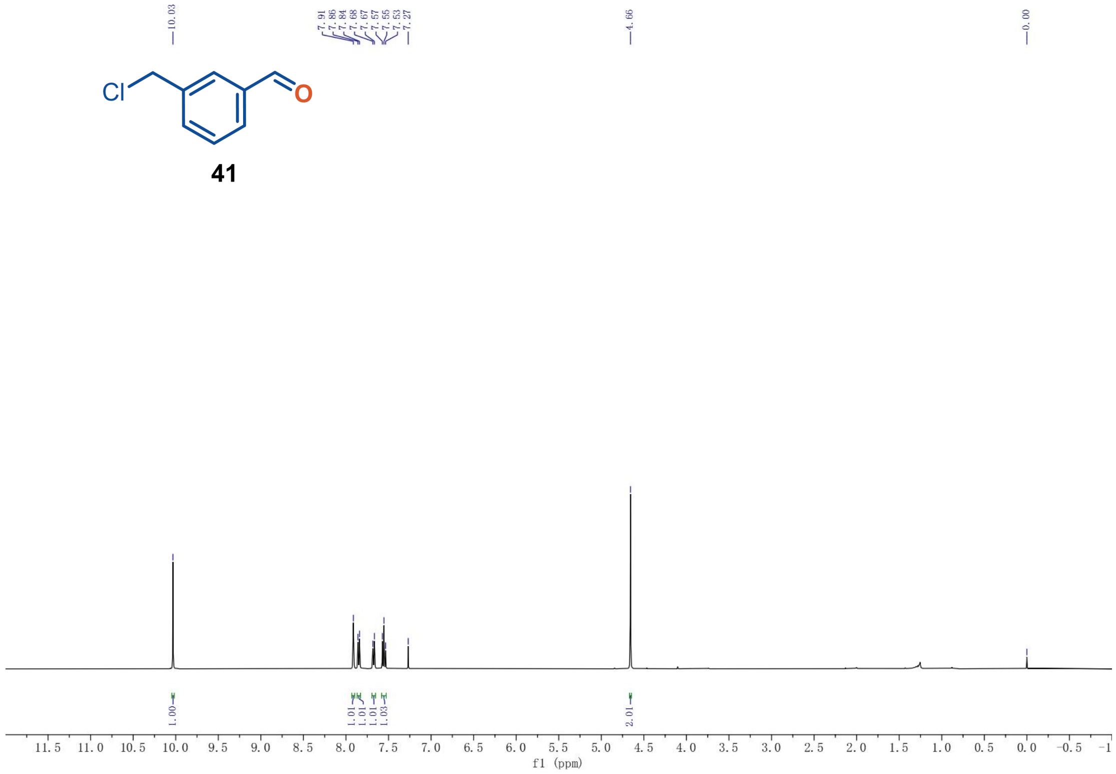

^{13}C NMR spectrum of **41**

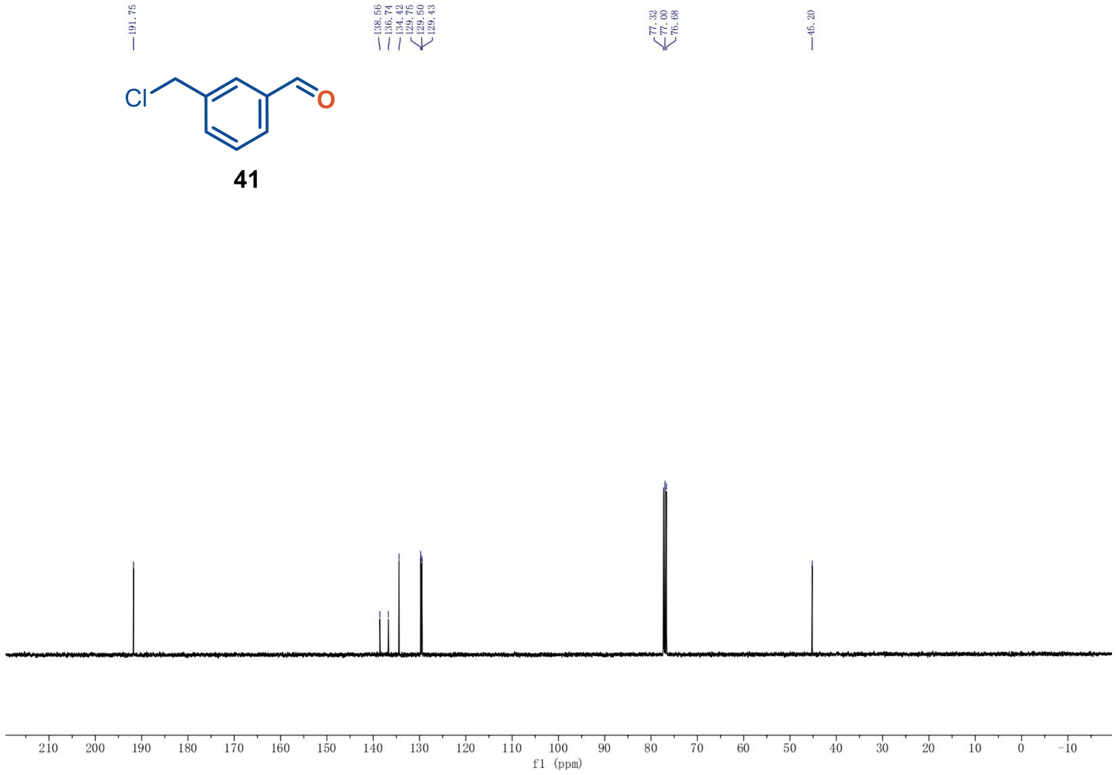

^{1}H NMR spectrum of **42**

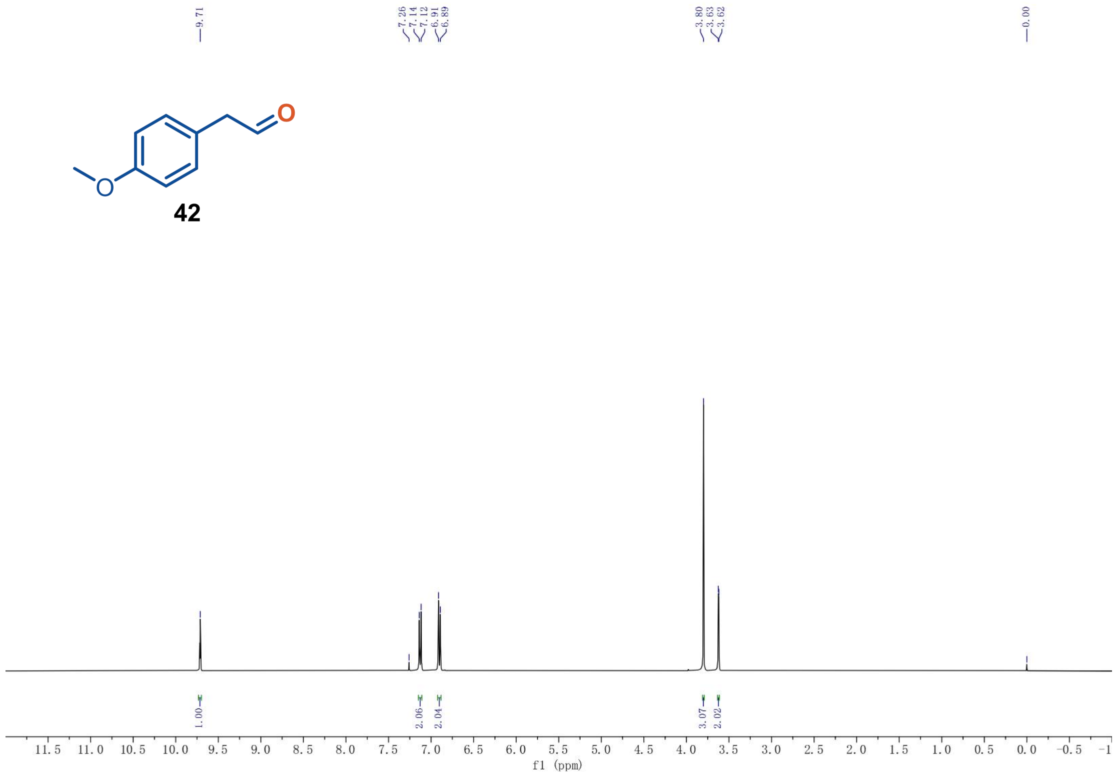

^{13}C NMR spectrum of **42**

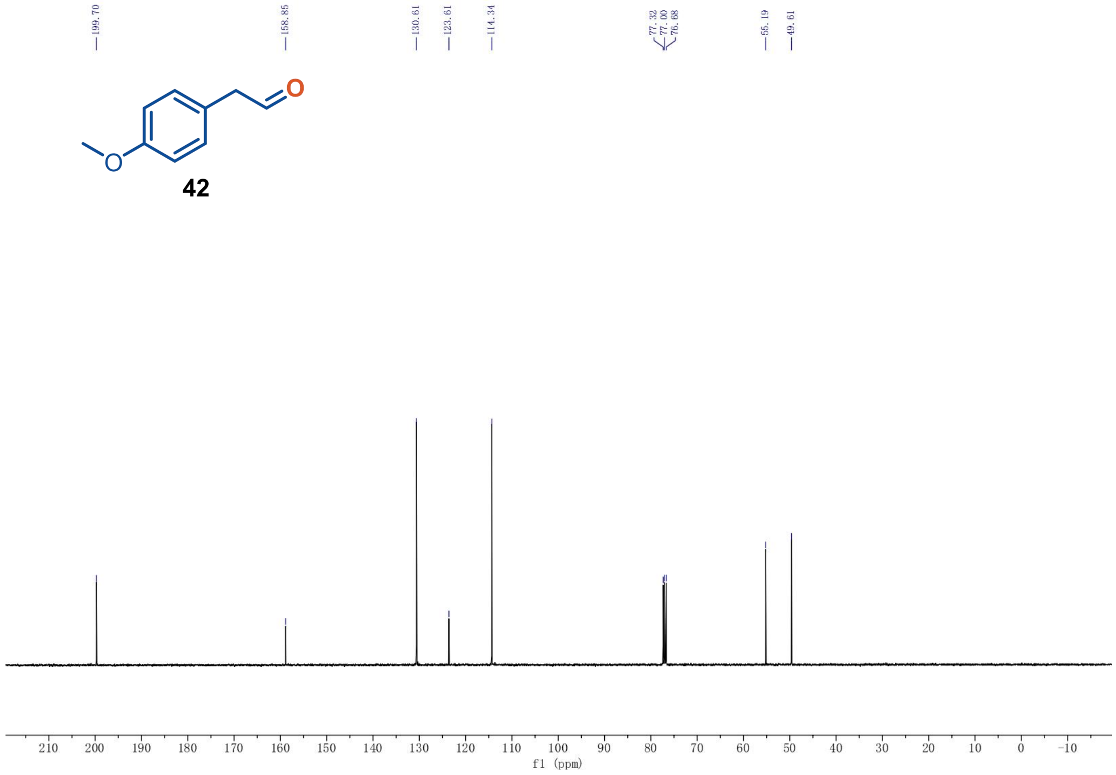

^{1}H NMR spectrum of **43**

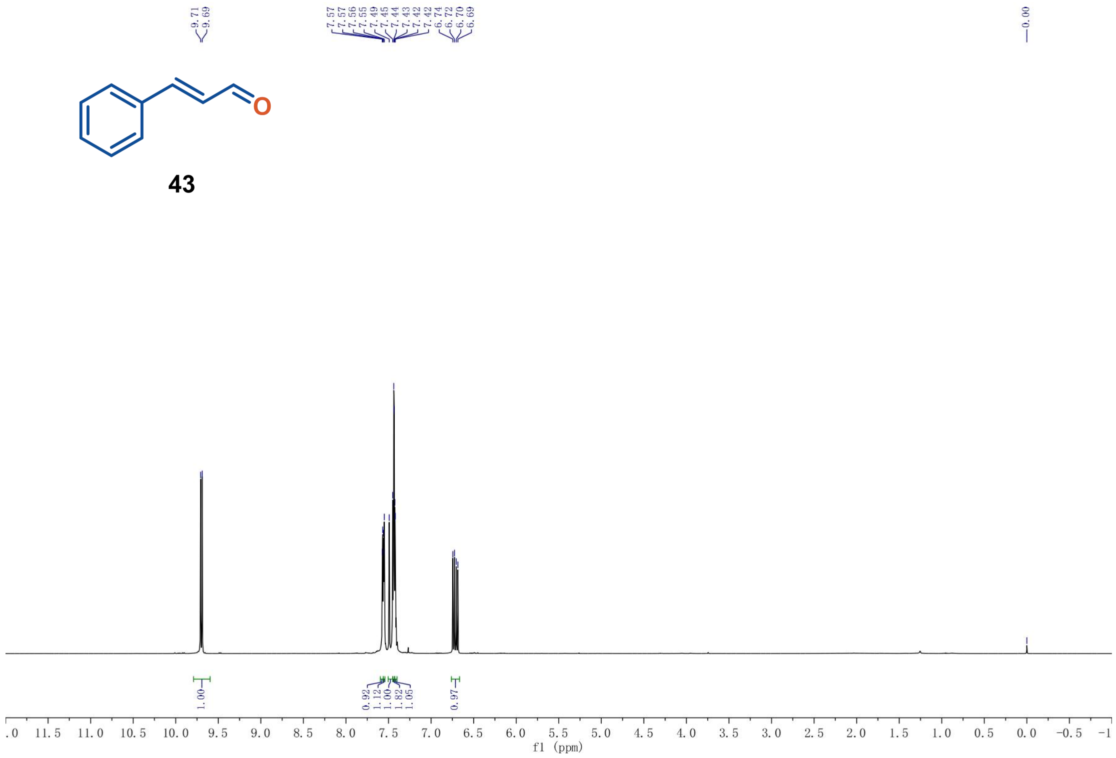

^{13}C NMR spectrum of **43**

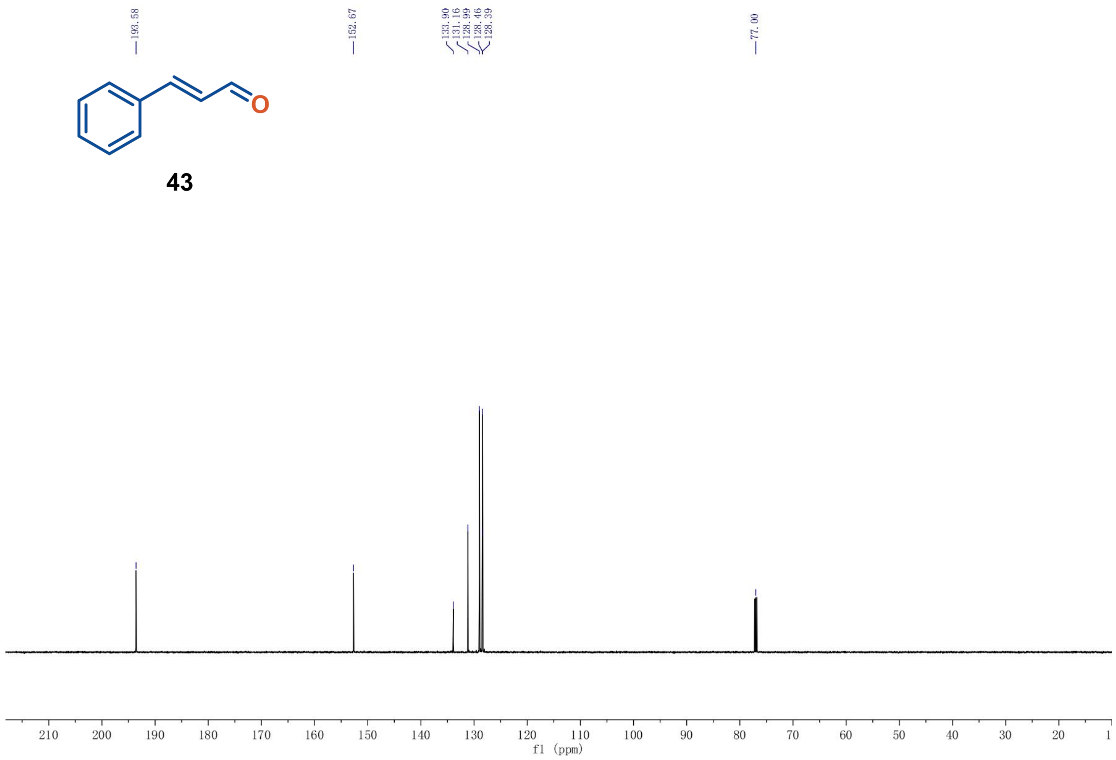

43

^{1}H NMR spectrum of **44**

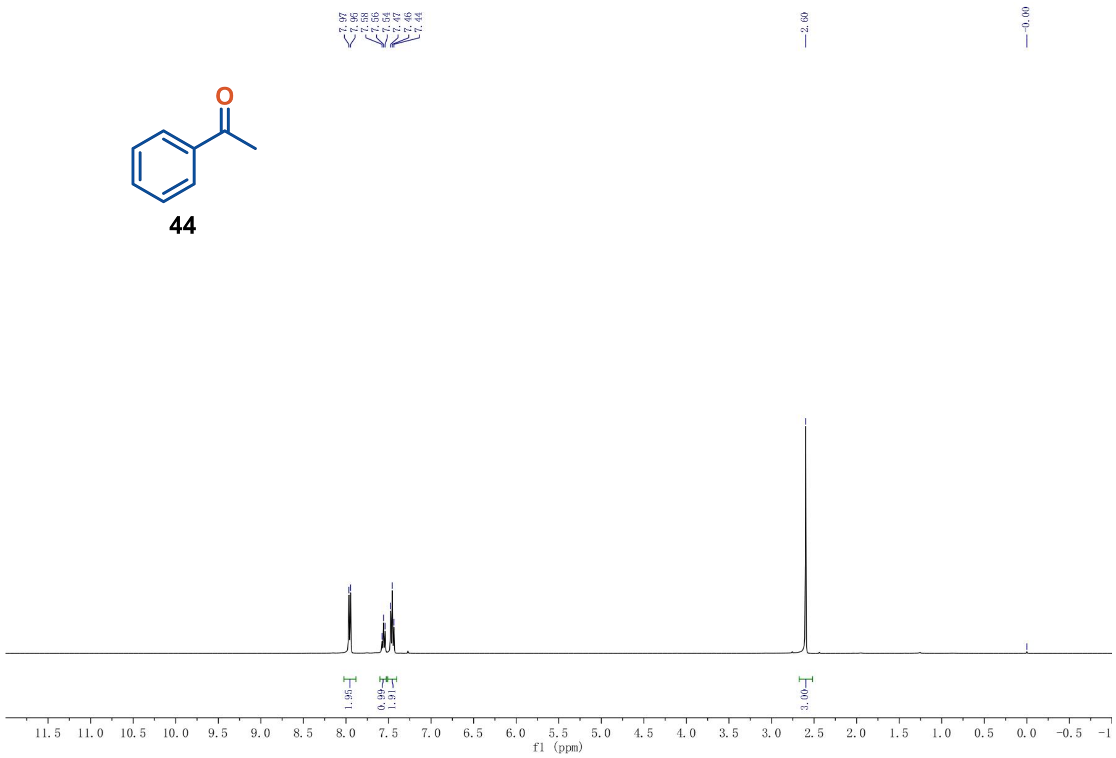

44

^{13}C NMR spectrum of **44**

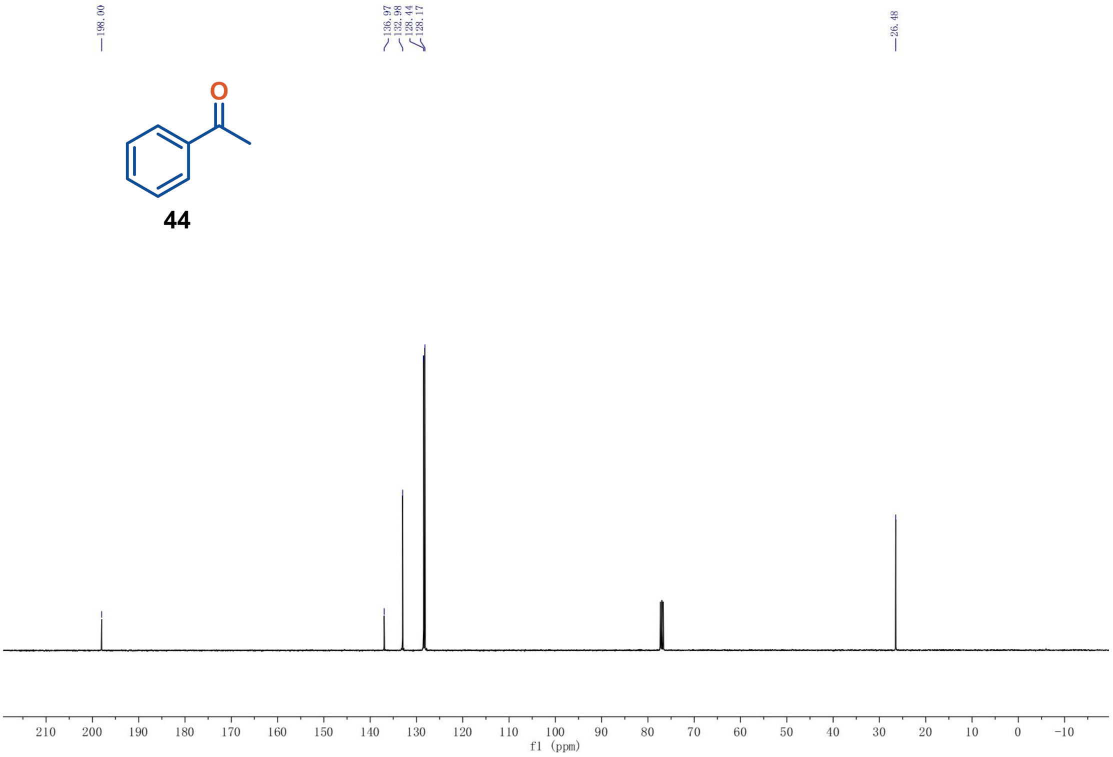

^{1}H NMR spectrum of **45**

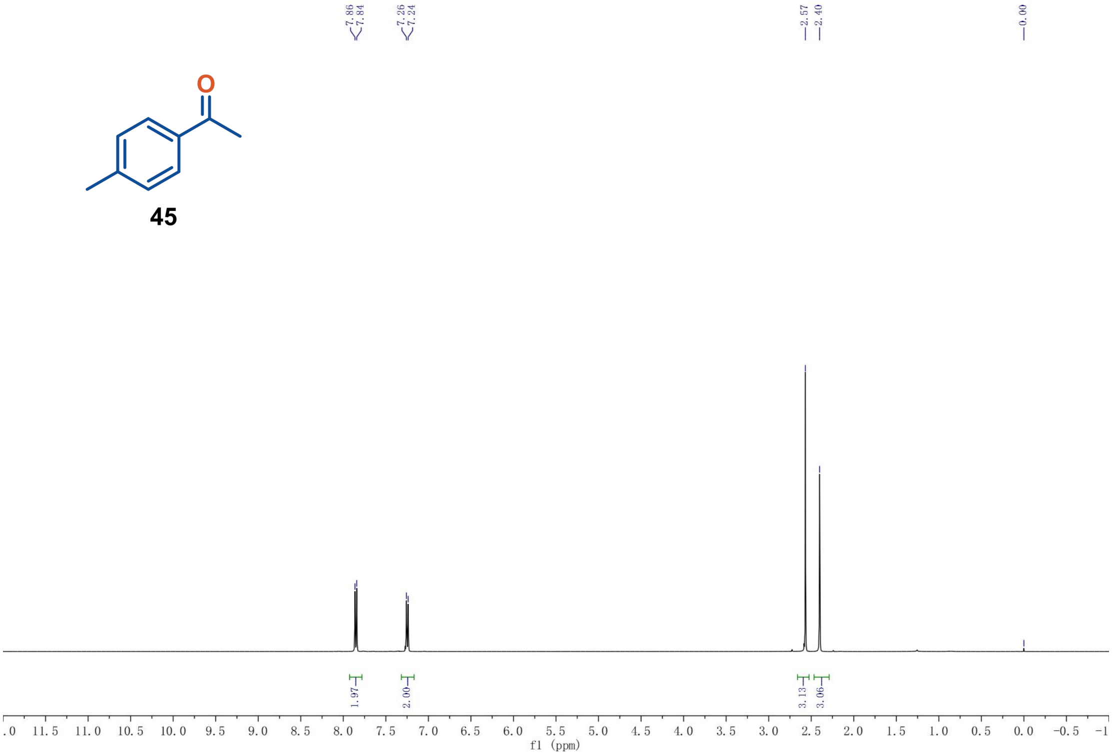

^{13}C NMR spectrum of **45**

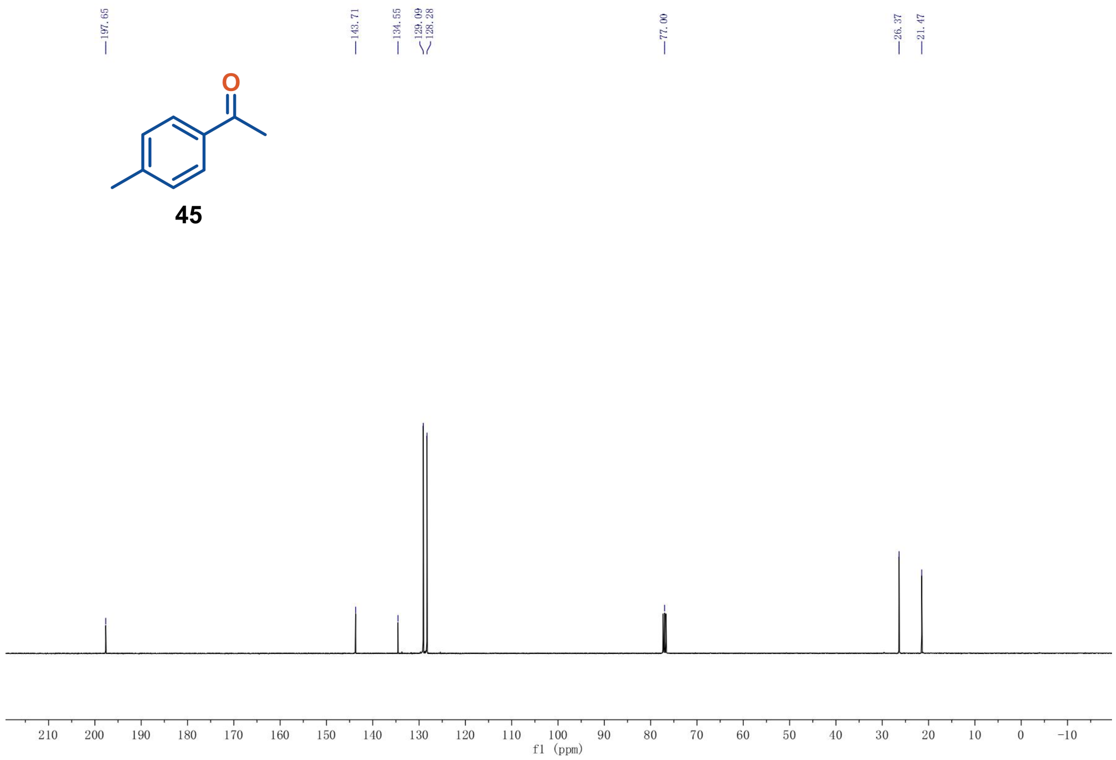

^{1}H NMR spectrum of **46**

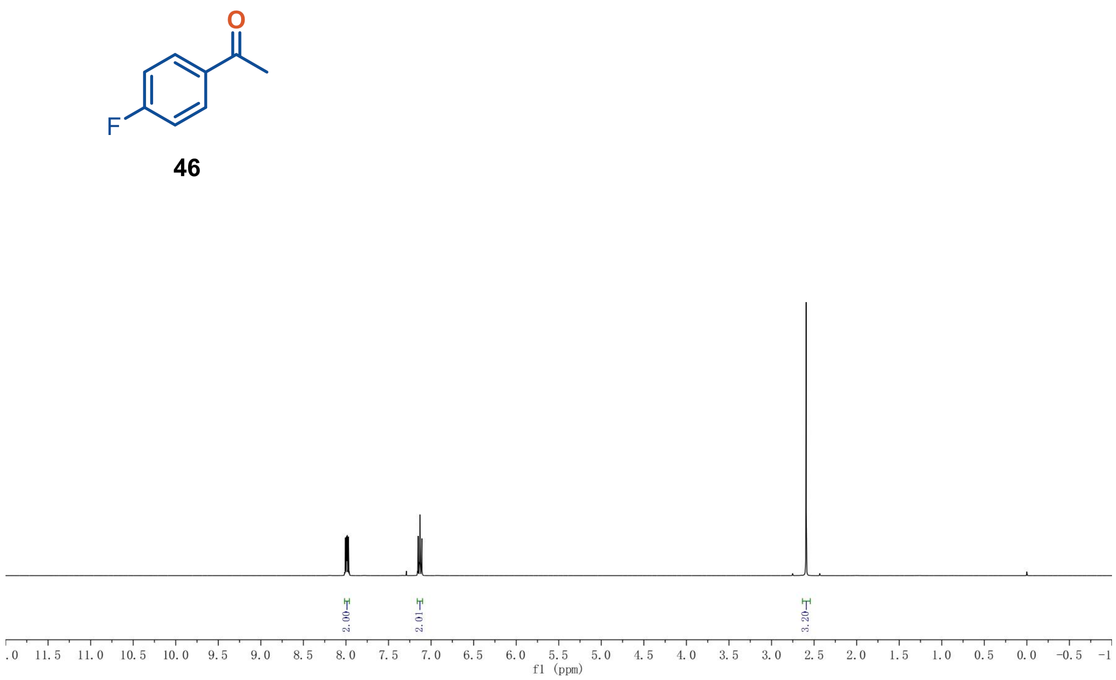

^{13}C NMR spectrum of **46**

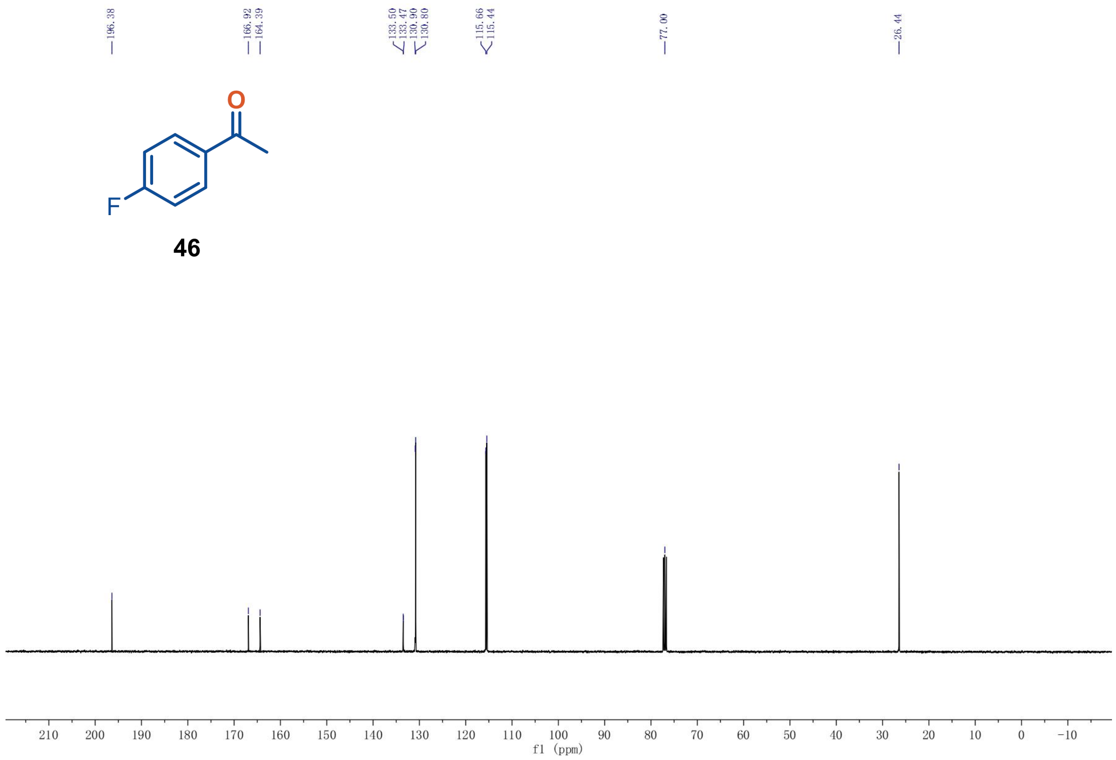

^{19}F NMR spectrum of **46**

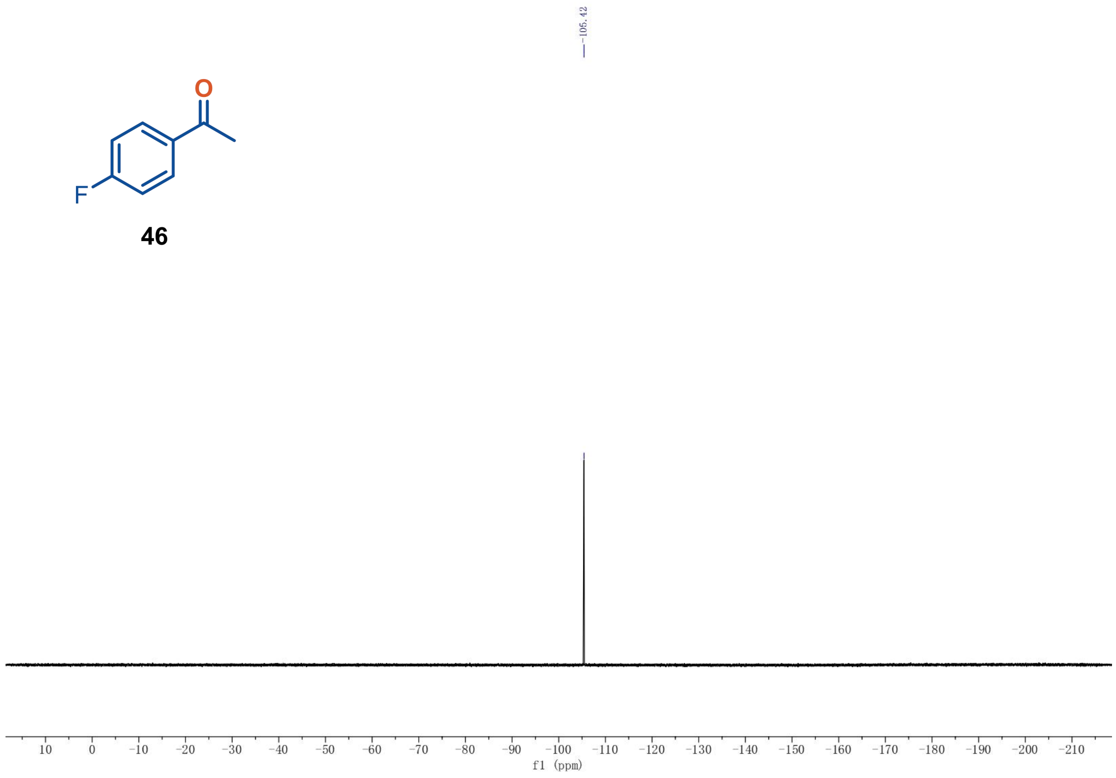

^{1}H NMR spectrum of **47**

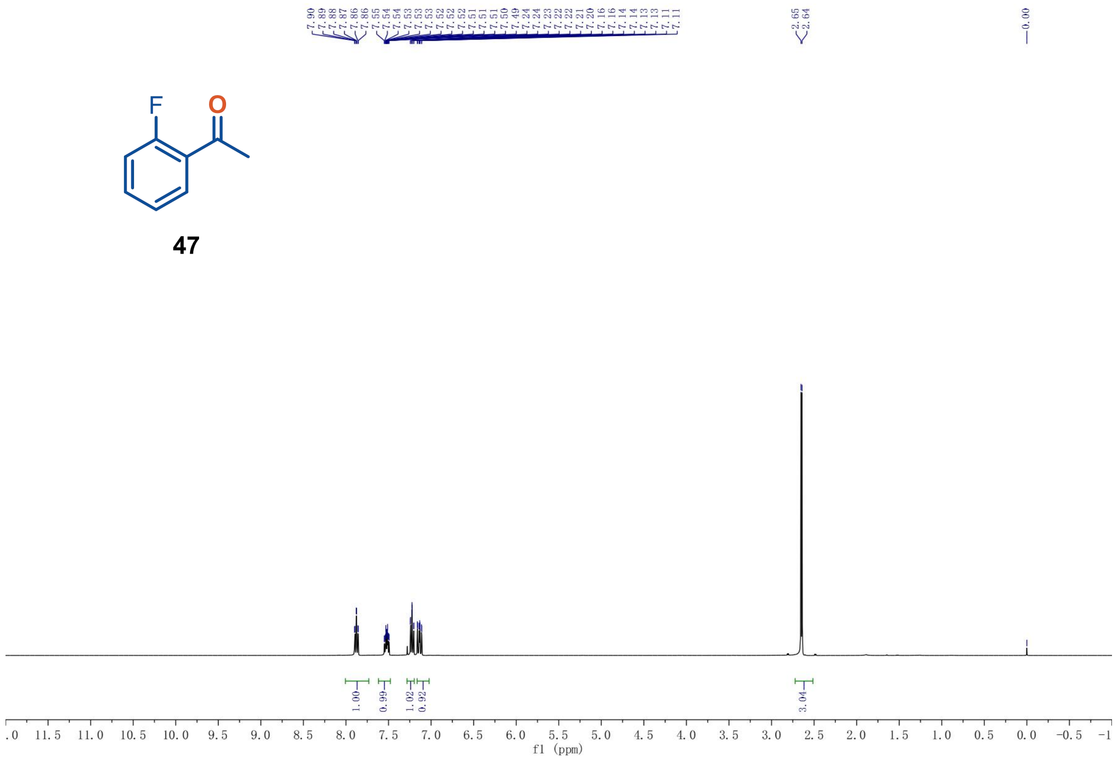

^{13}C NMR spectrum of **47**

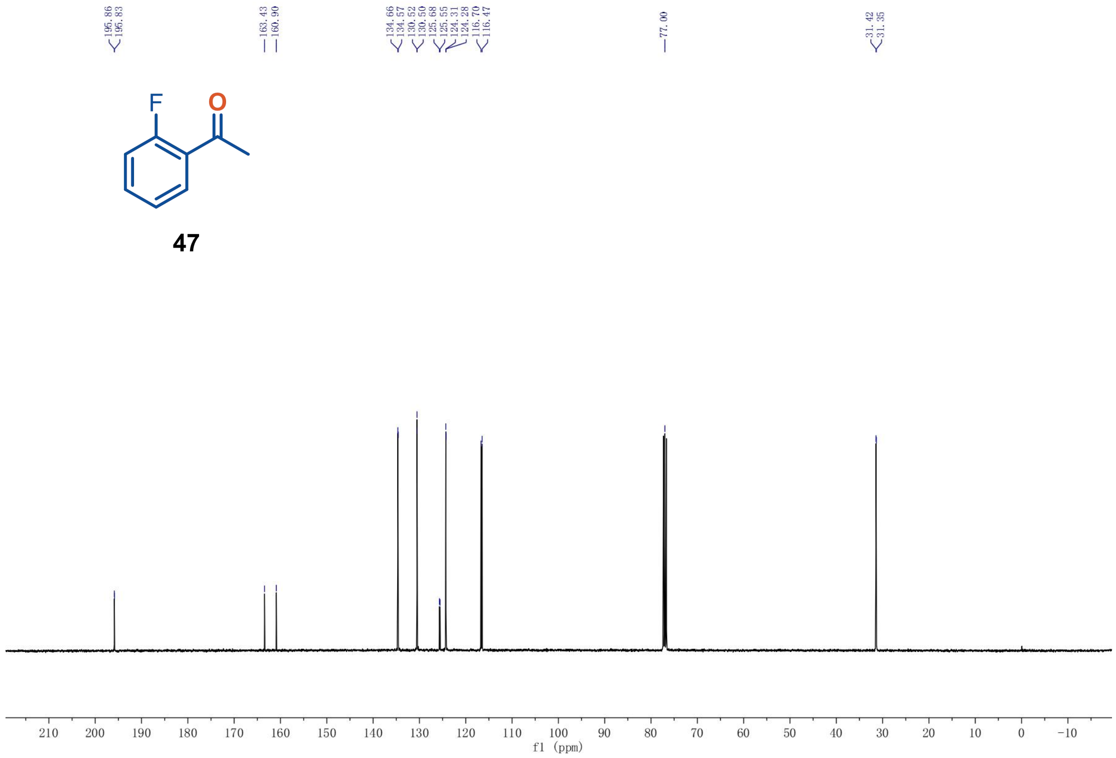

^{19}F NMR spectrum of **47**

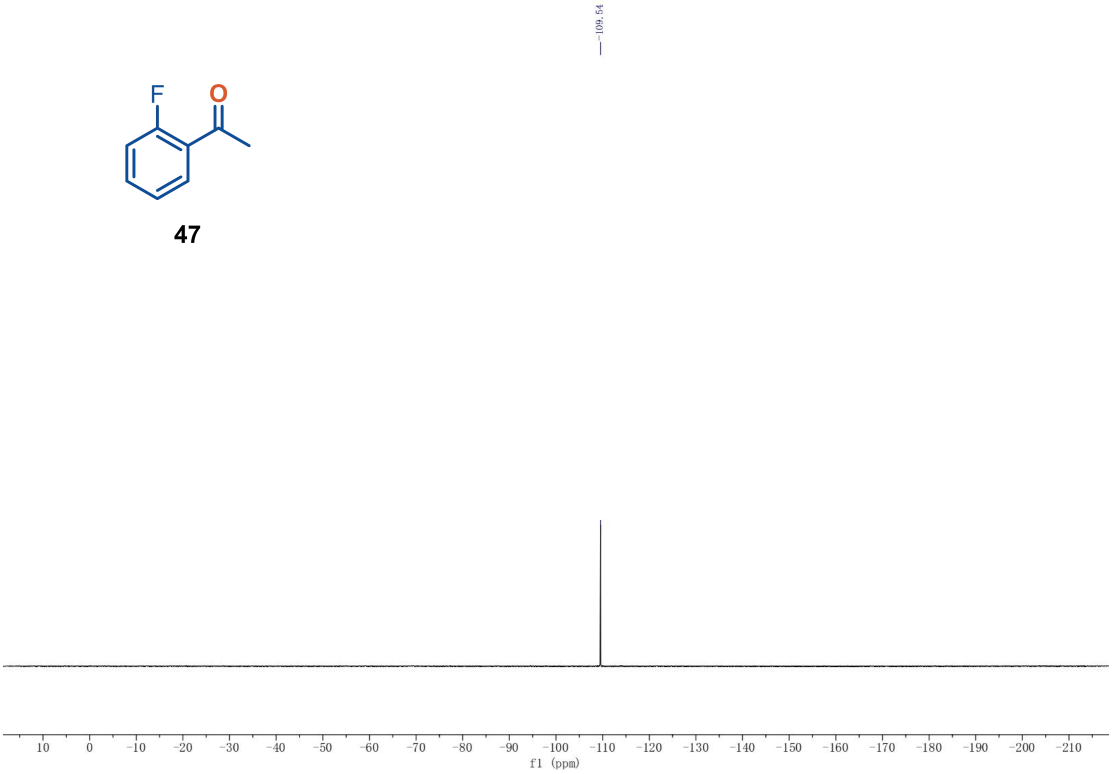

^{1}H NMR spectrum of **48**

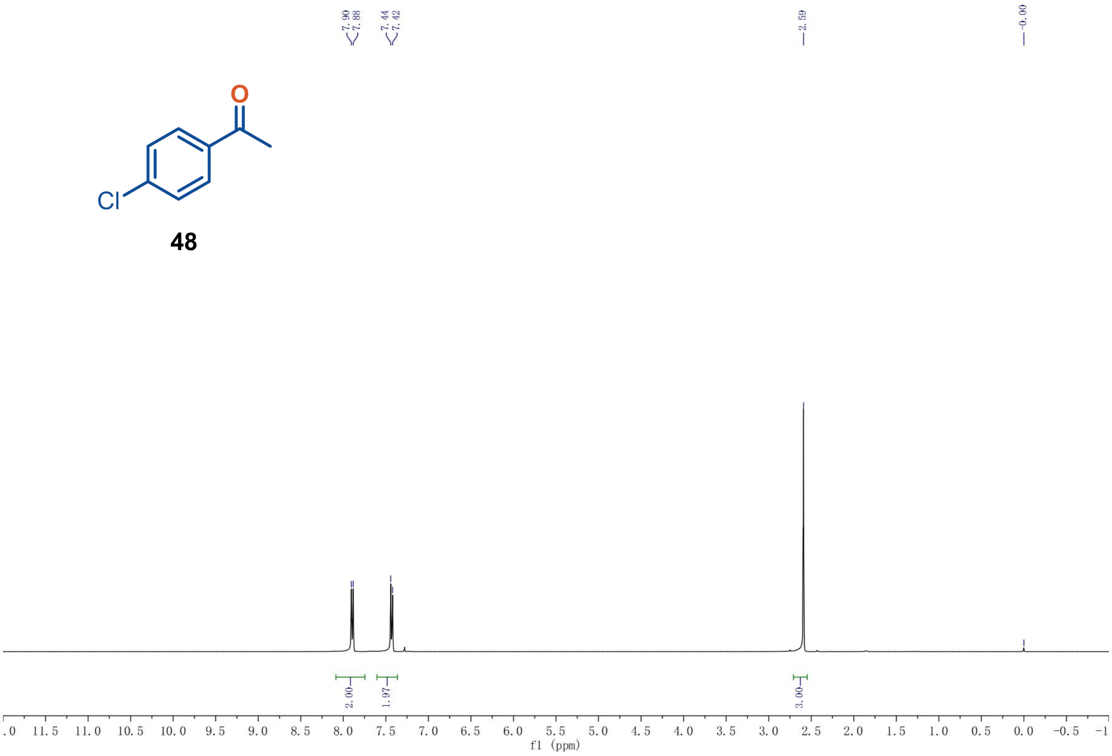

^{13}C NMR spectrum of **48**

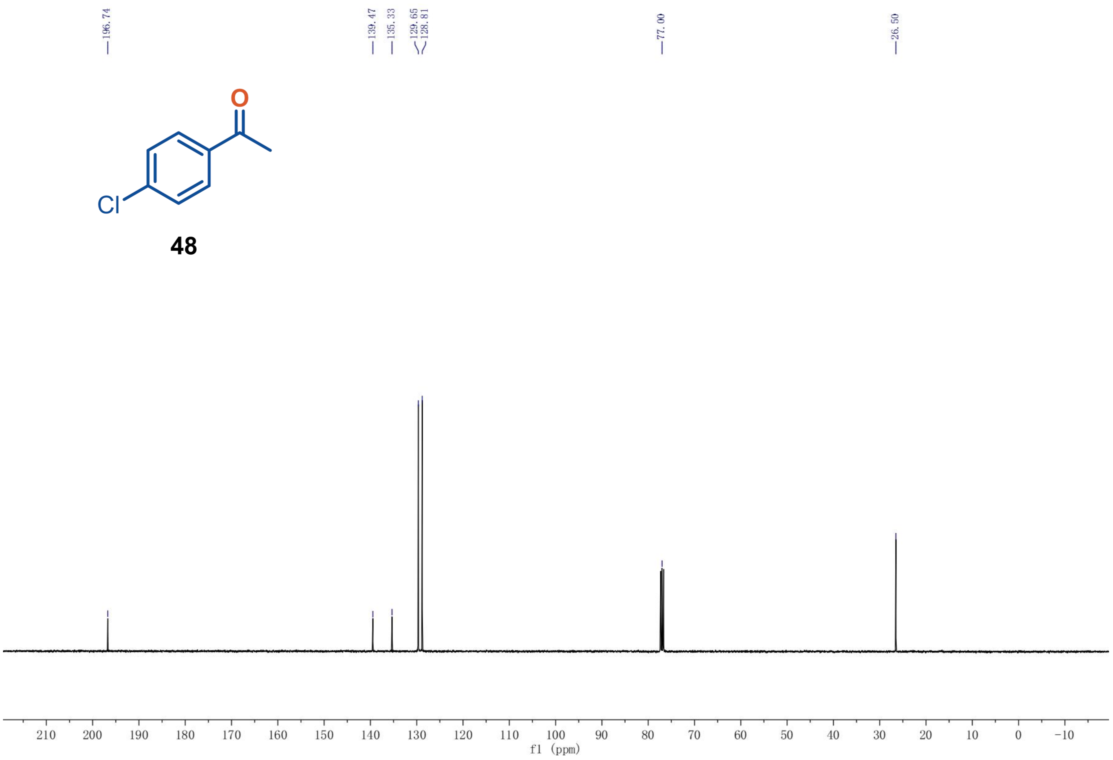

^{1}H NMR spectrum of **49**

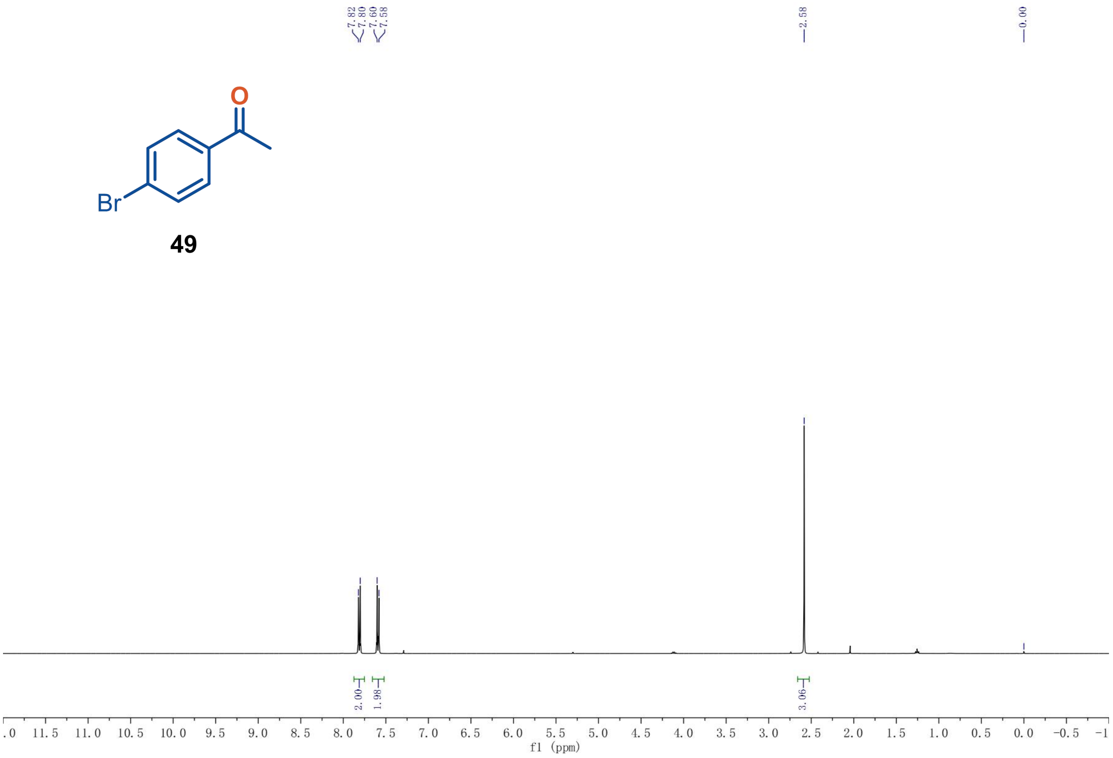

^{13}C NMR spectrum of **49**

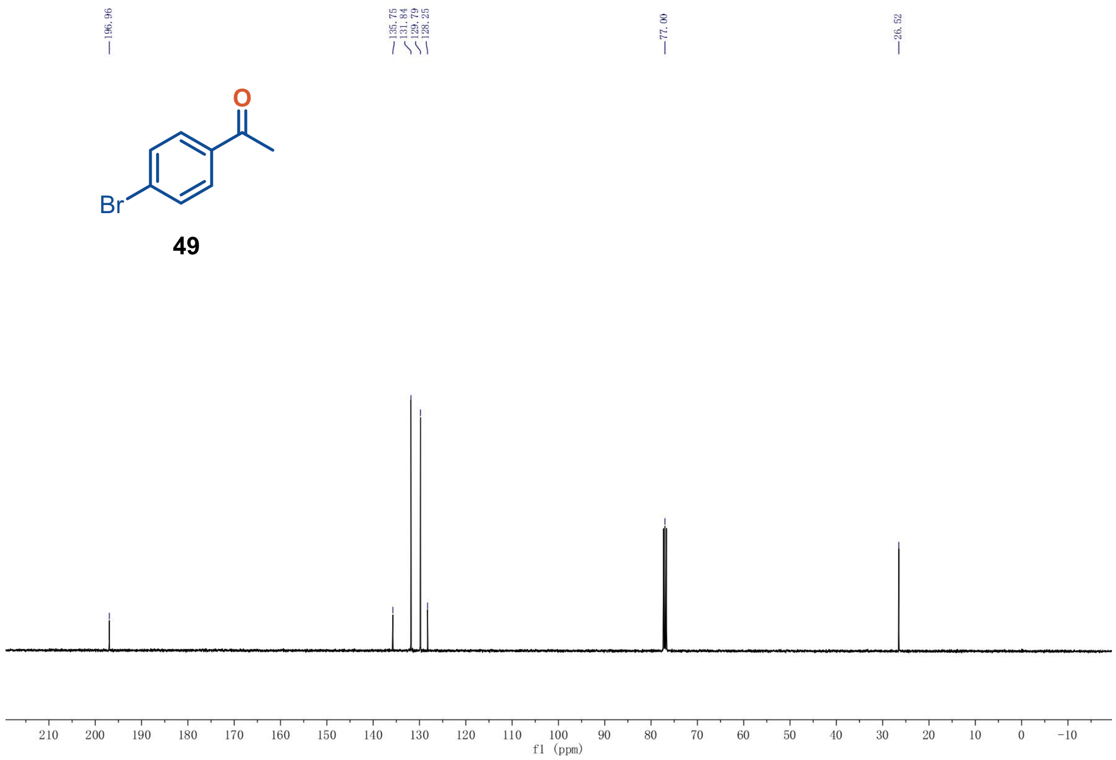

49

^{1}H NMR spectrum of **50**

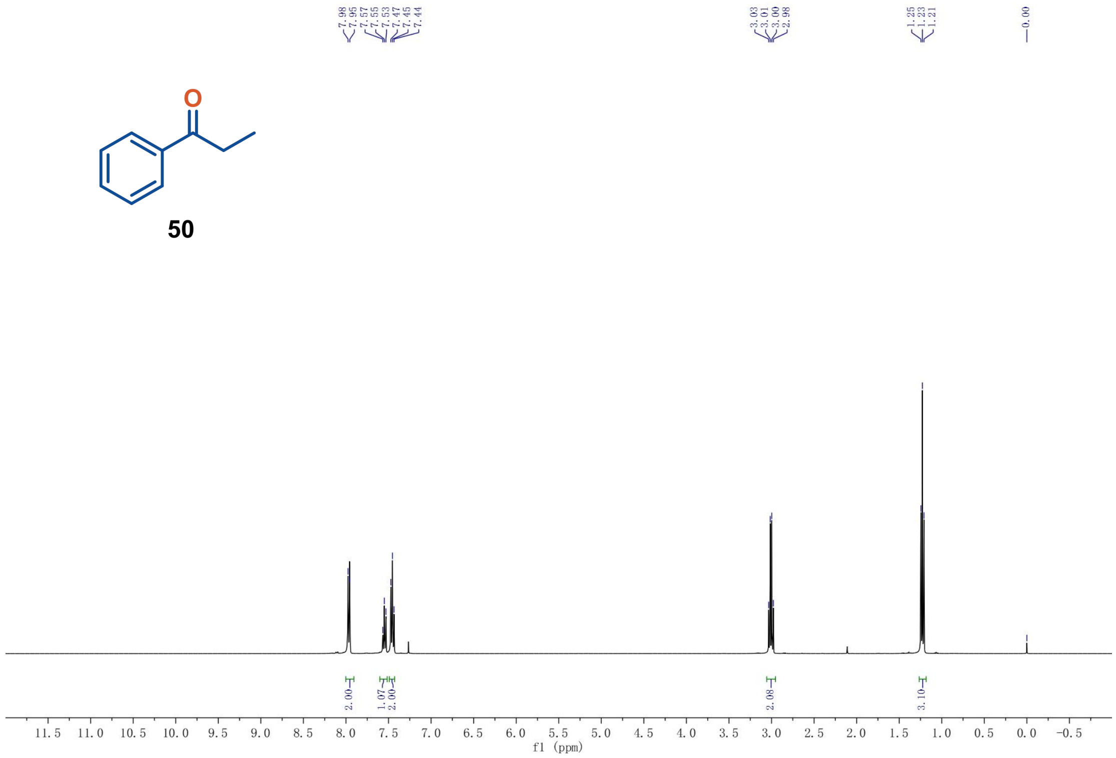

50

^{13}C NMR spectrum of **50**

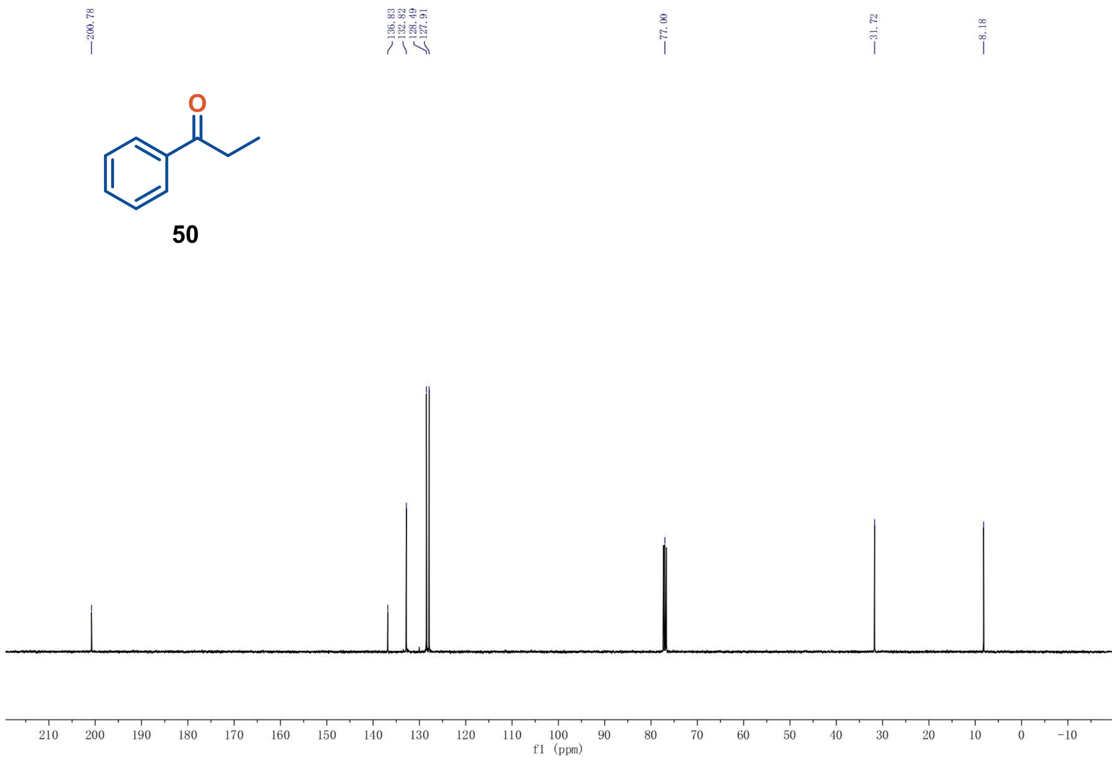

^{1}H NMR spectrum of **51**

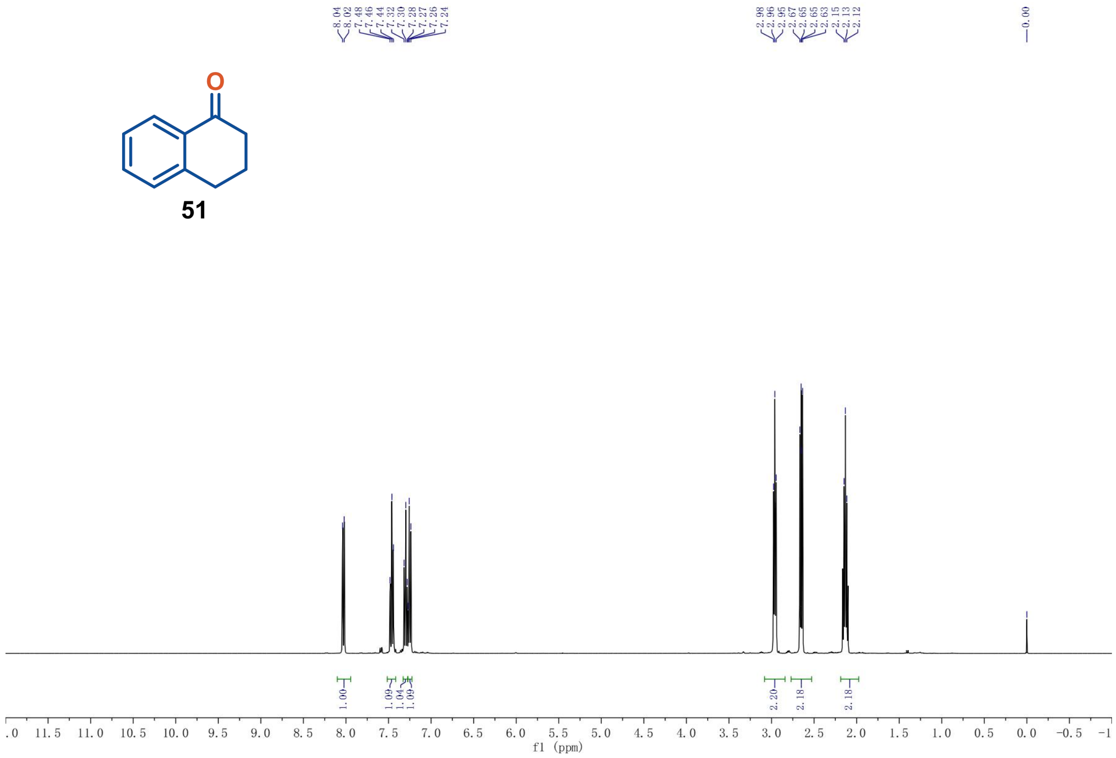

^{13}C NMR spectrum of **51**

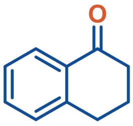

51

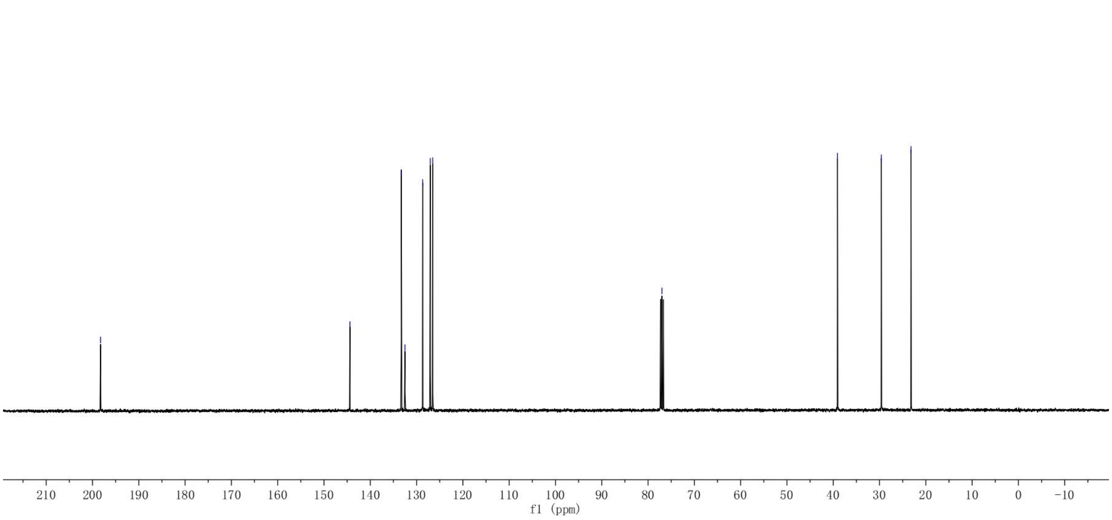

^{1}H NMR spectrum of **52**

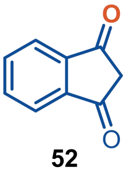

52

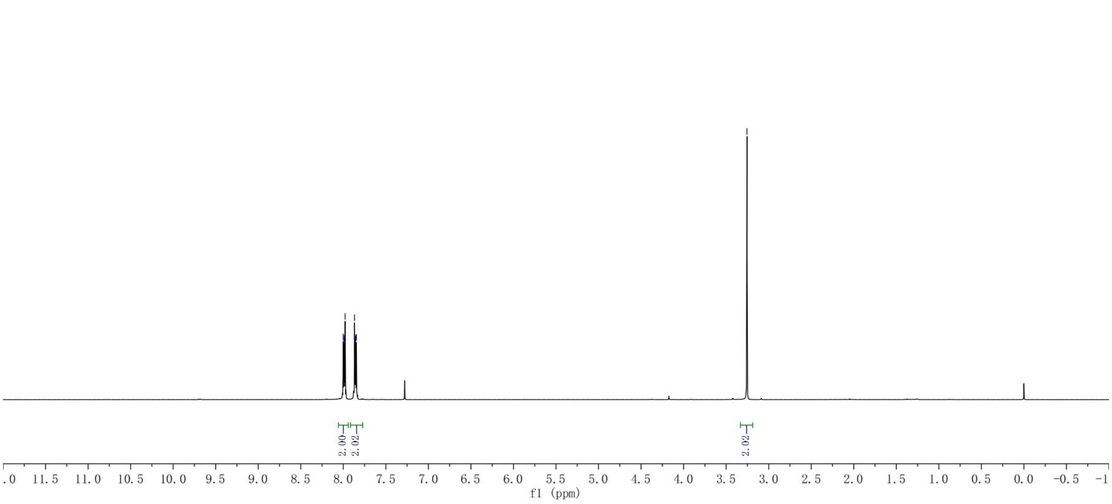

^{13}C NMR spectrum of **52**

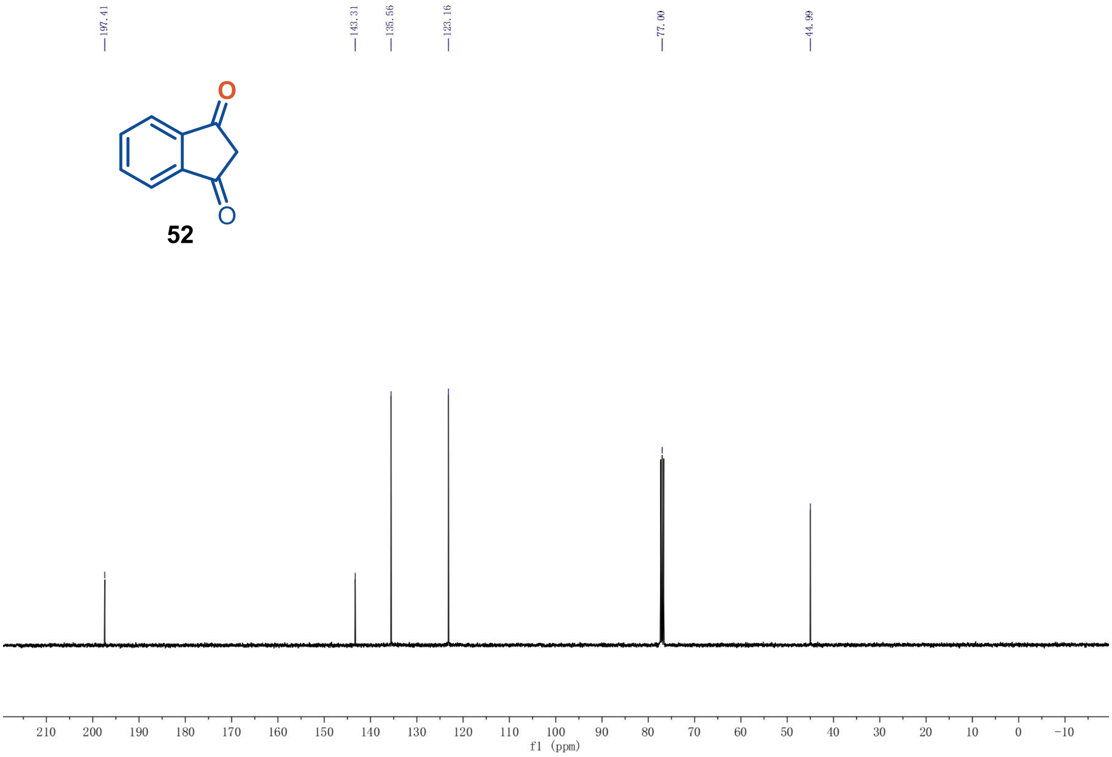

^{1}H NMR spectrum of **53**

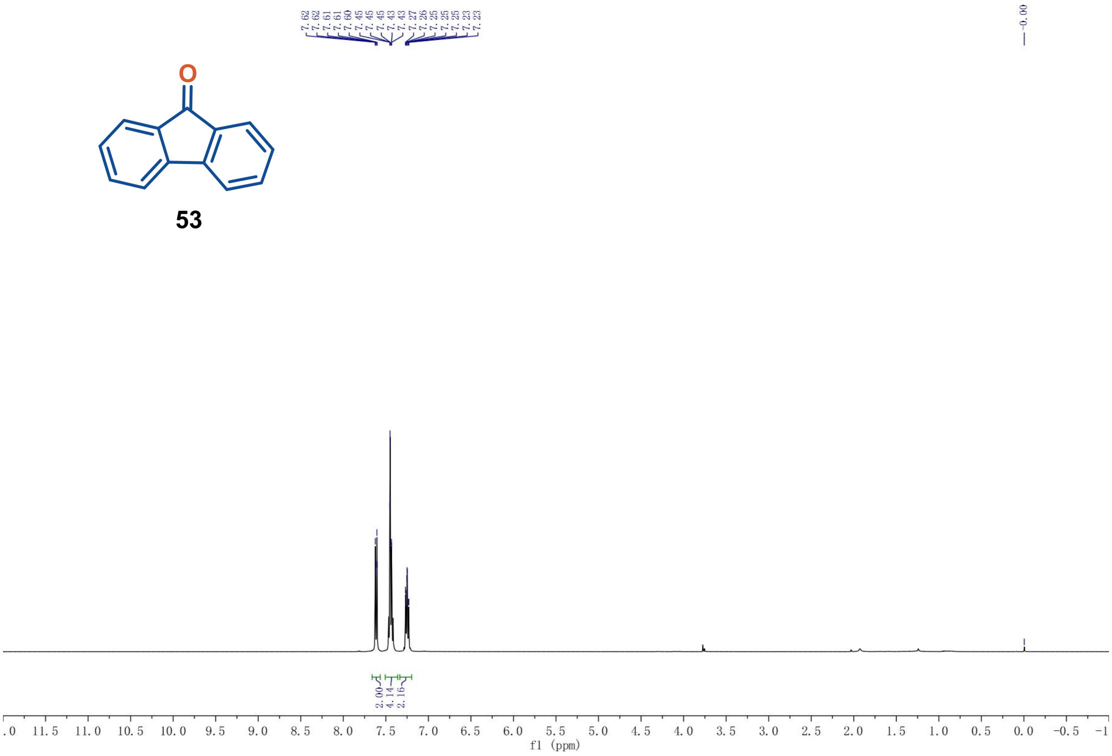

^{13}C NMR spectrum of **53**

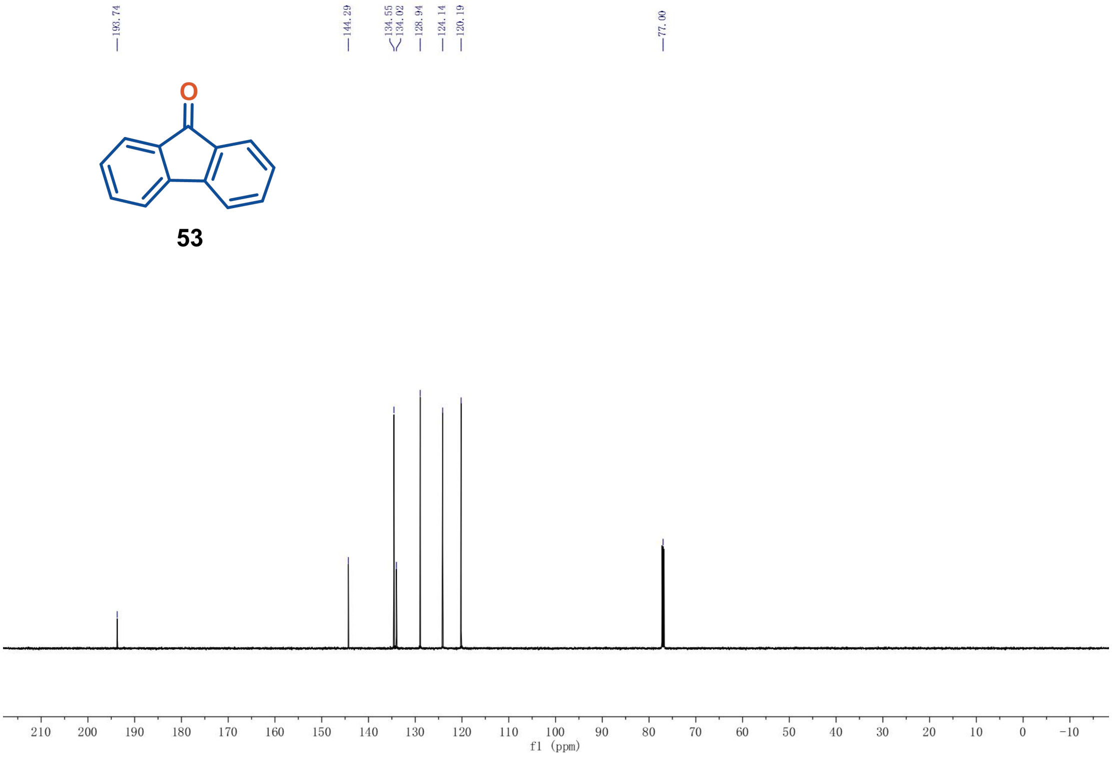

^{1}H NMR spectrum of **54**

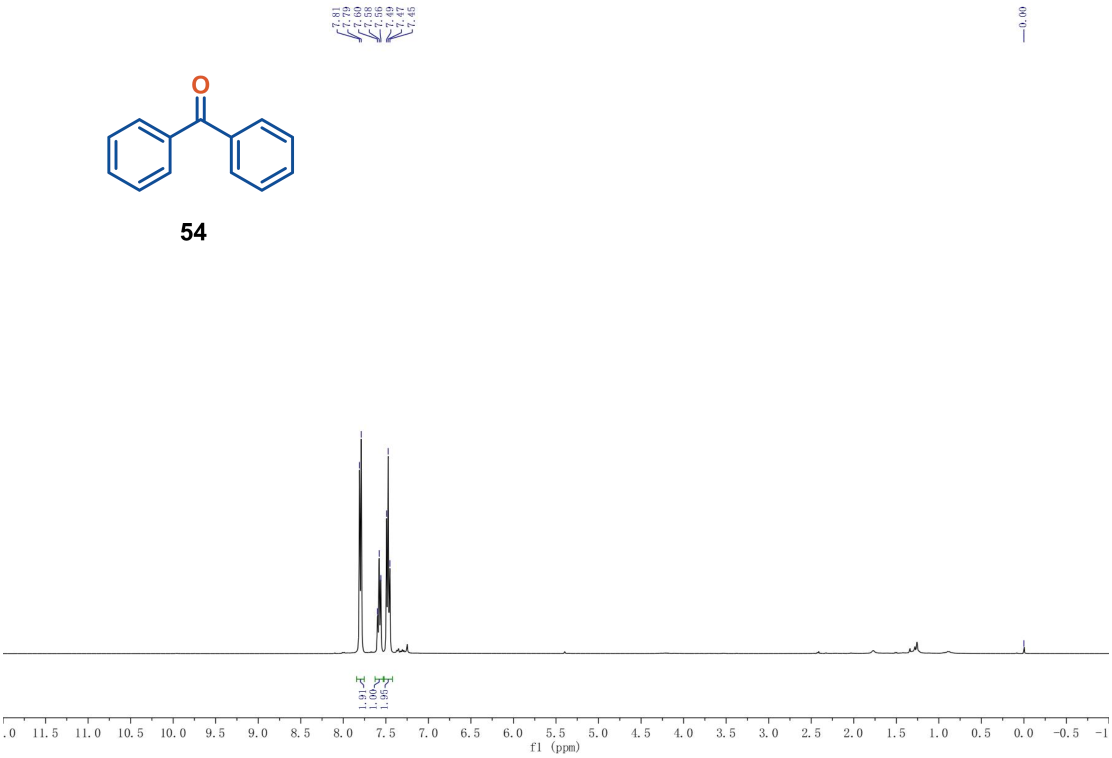

^{13}C NMR spectrum of **54**

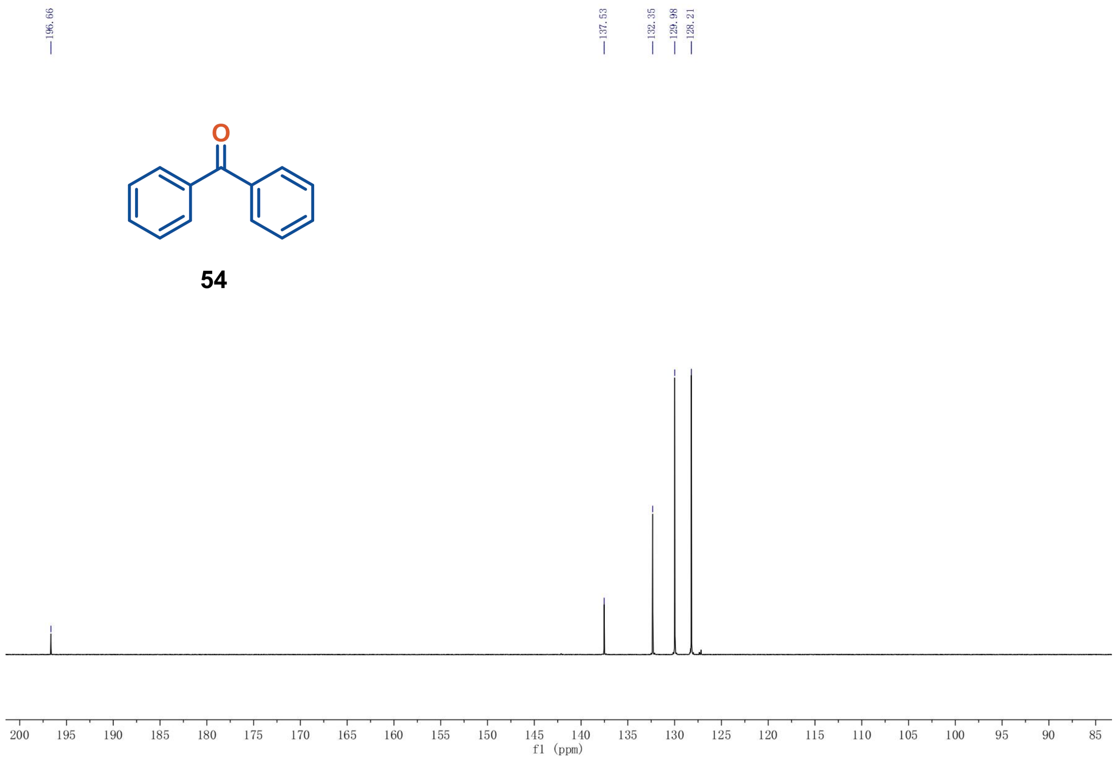

^{1}H NMR spectrum of **55**

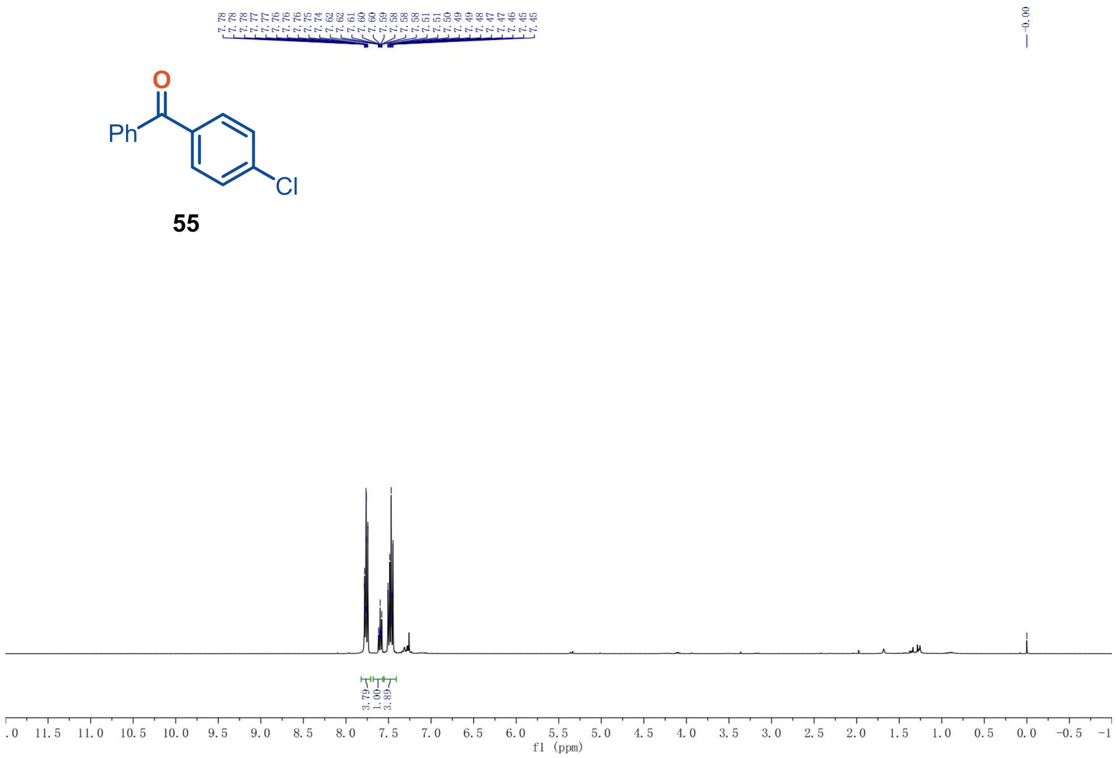

^{13}C NMR spectrum of **55**

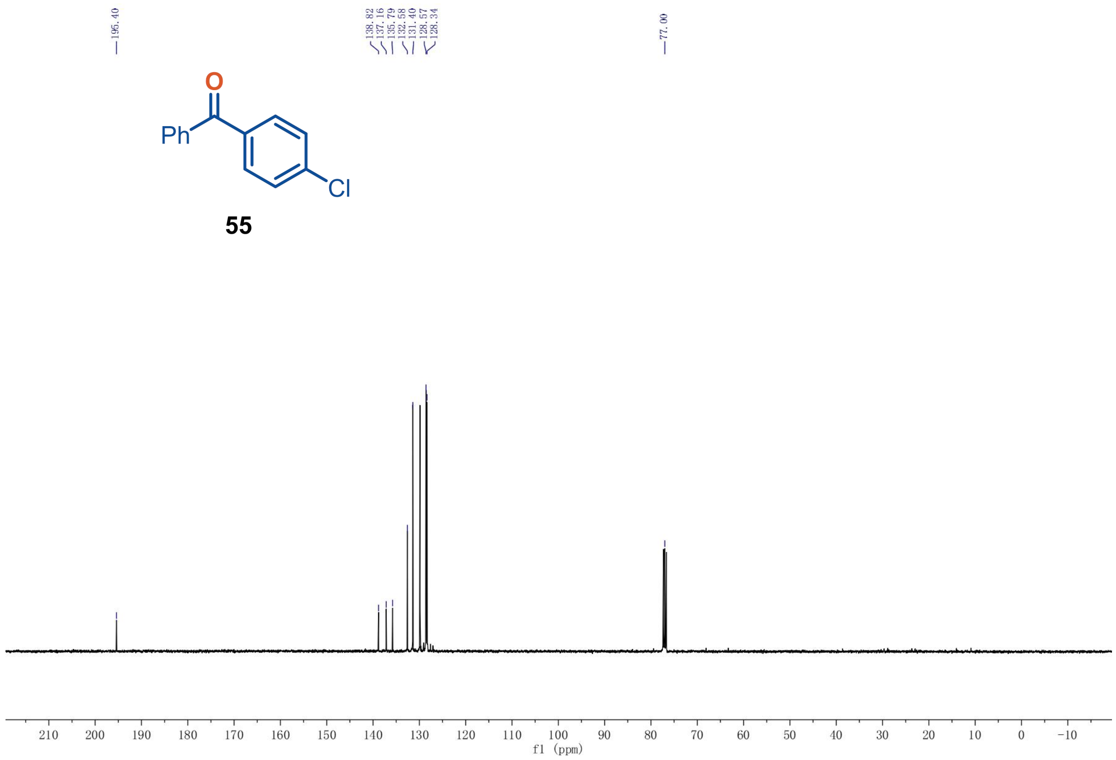

^{1}H NMR spectrum of **56**

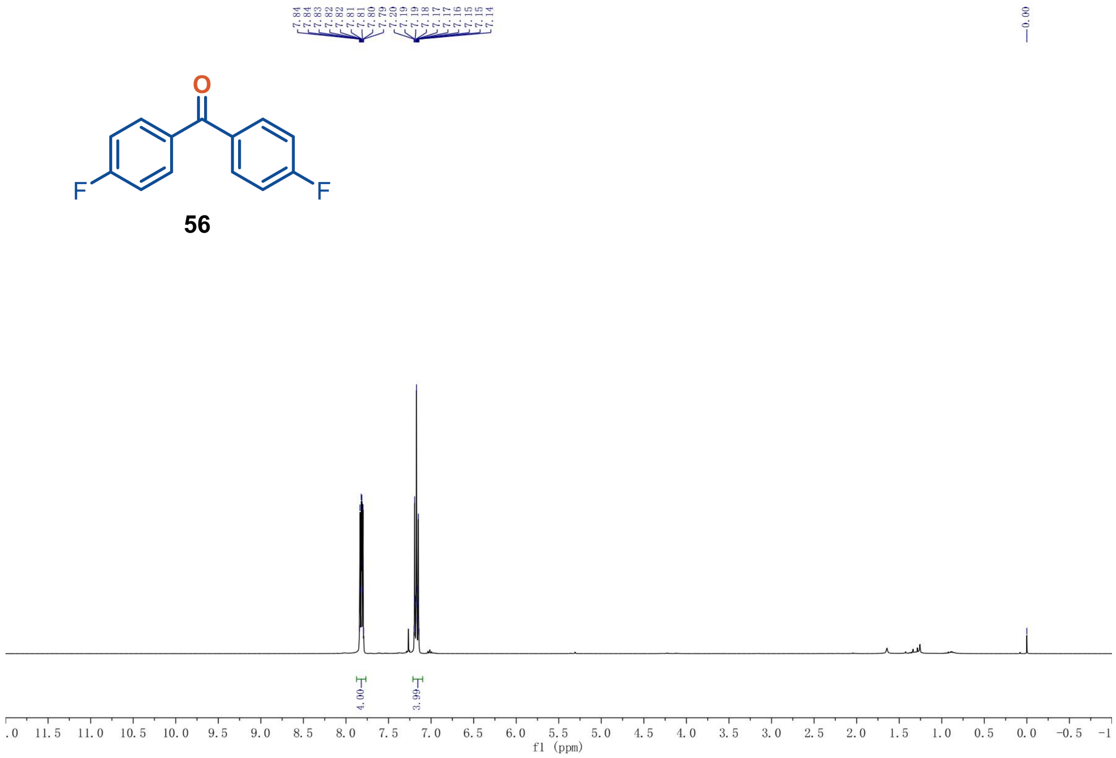

^{13}C NMR spectrum of **56**

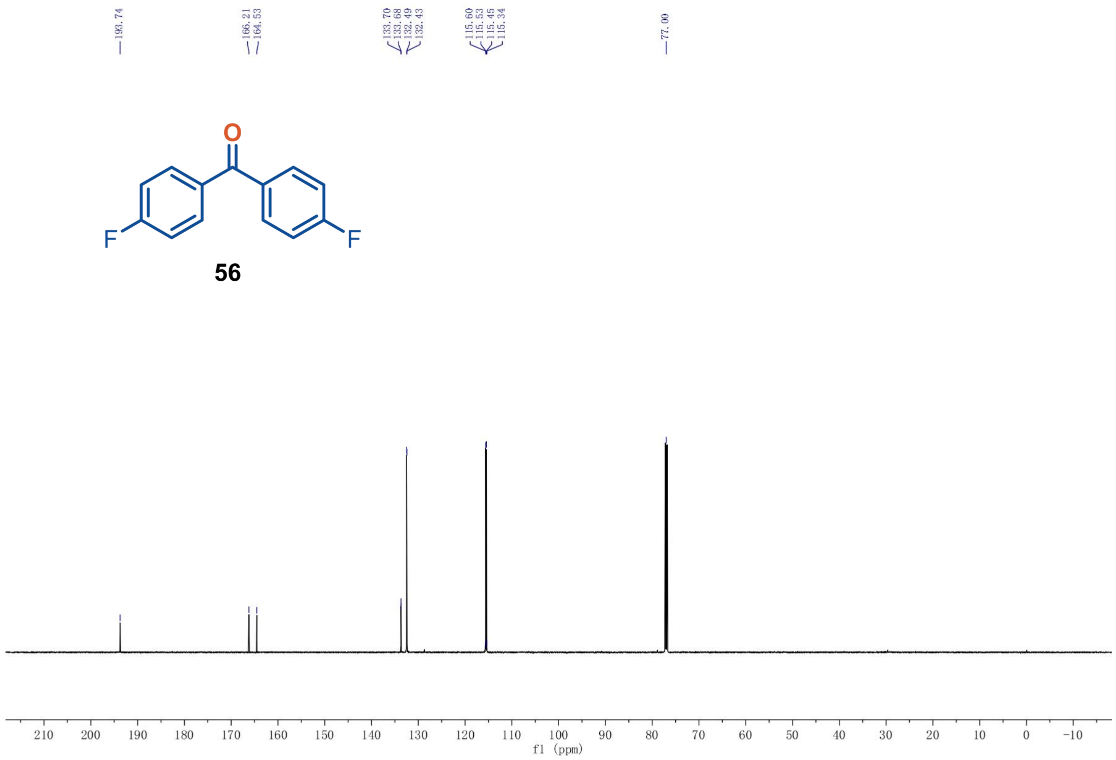

^{19}F NMR spectrum of **56**

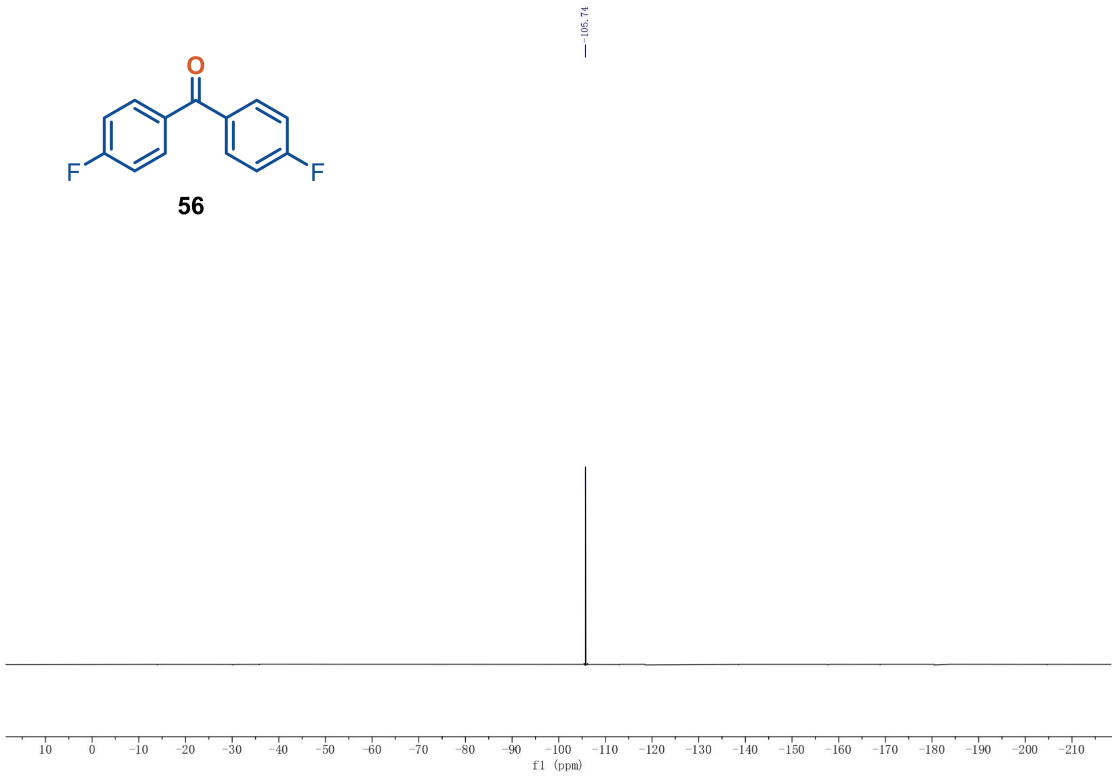

^{1}H NMR spectrum of **57**

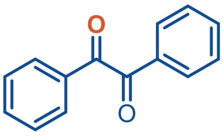

57

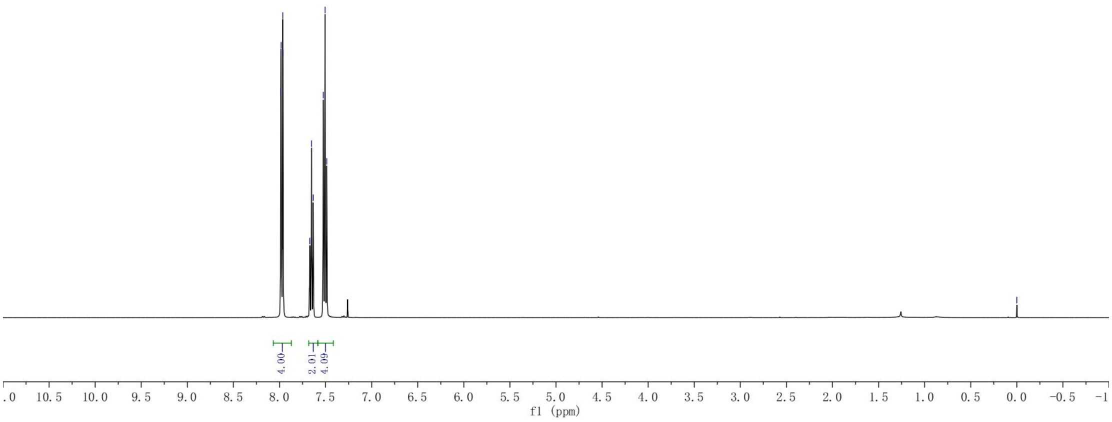

^{13}C NMR spectrum of **57**

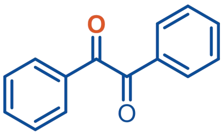

57

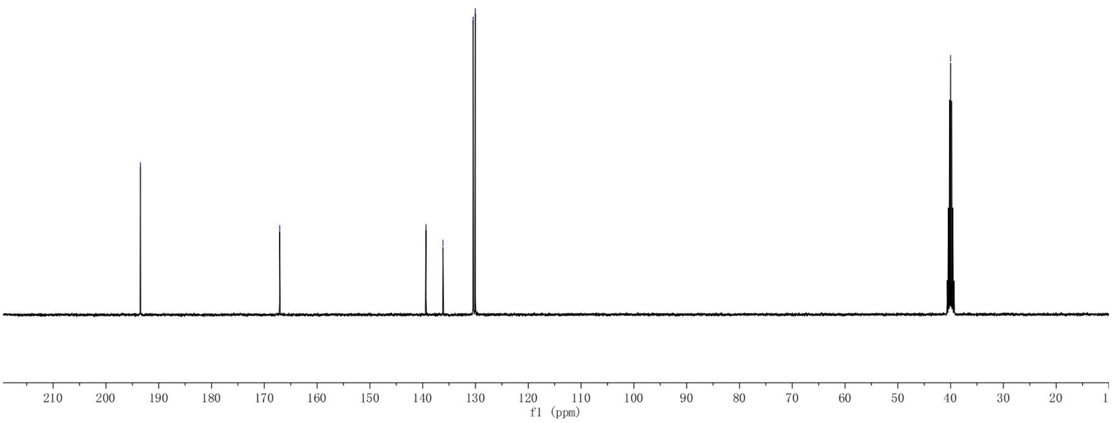

^{1}H NMR spectrum of **58**

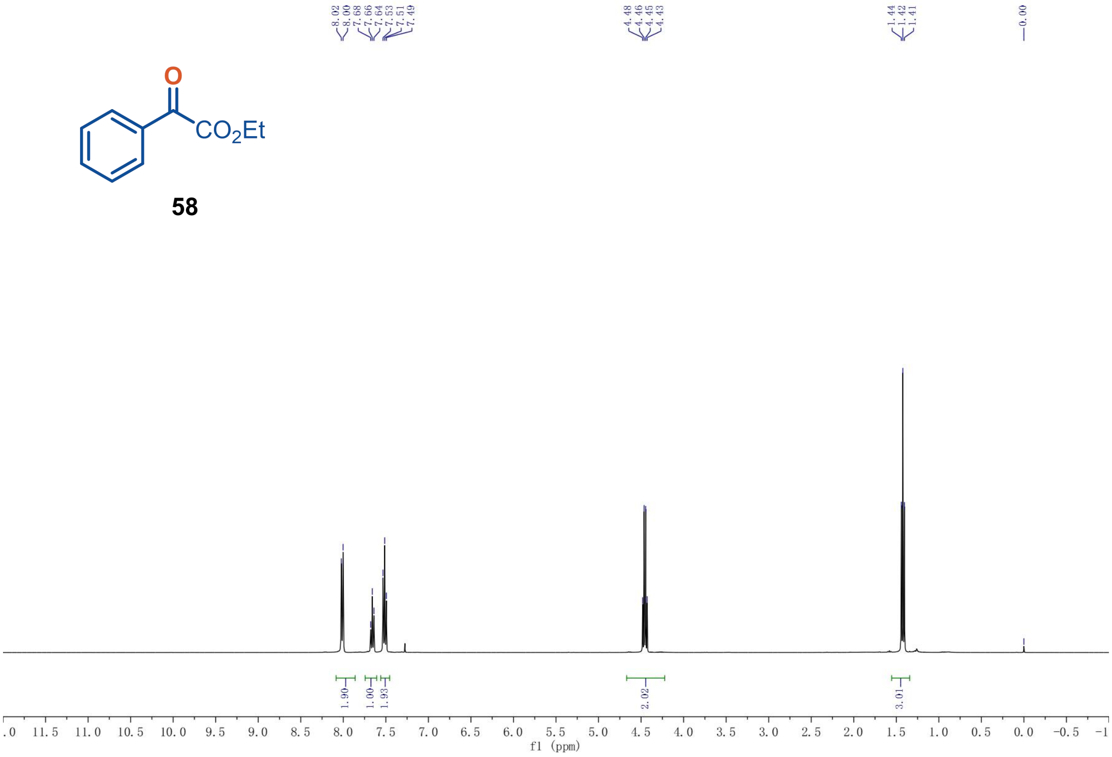

58

^{13}C NMR spectrum of **58**

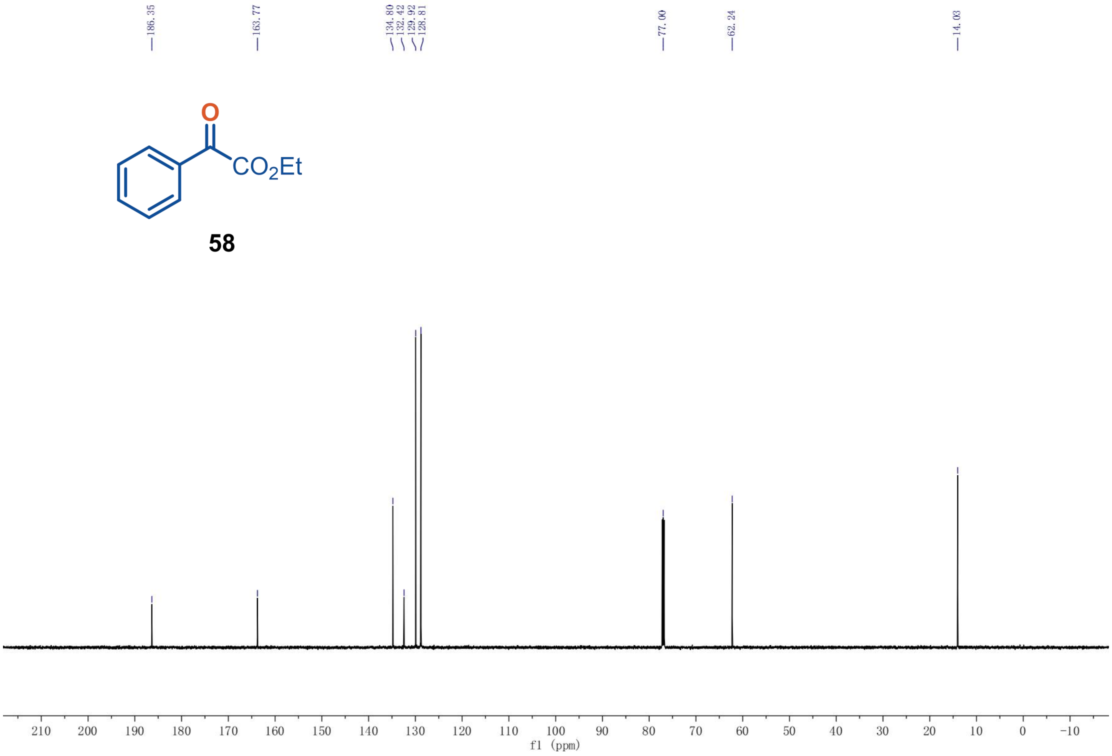

58

^{1}H NMR spectrum of **59**

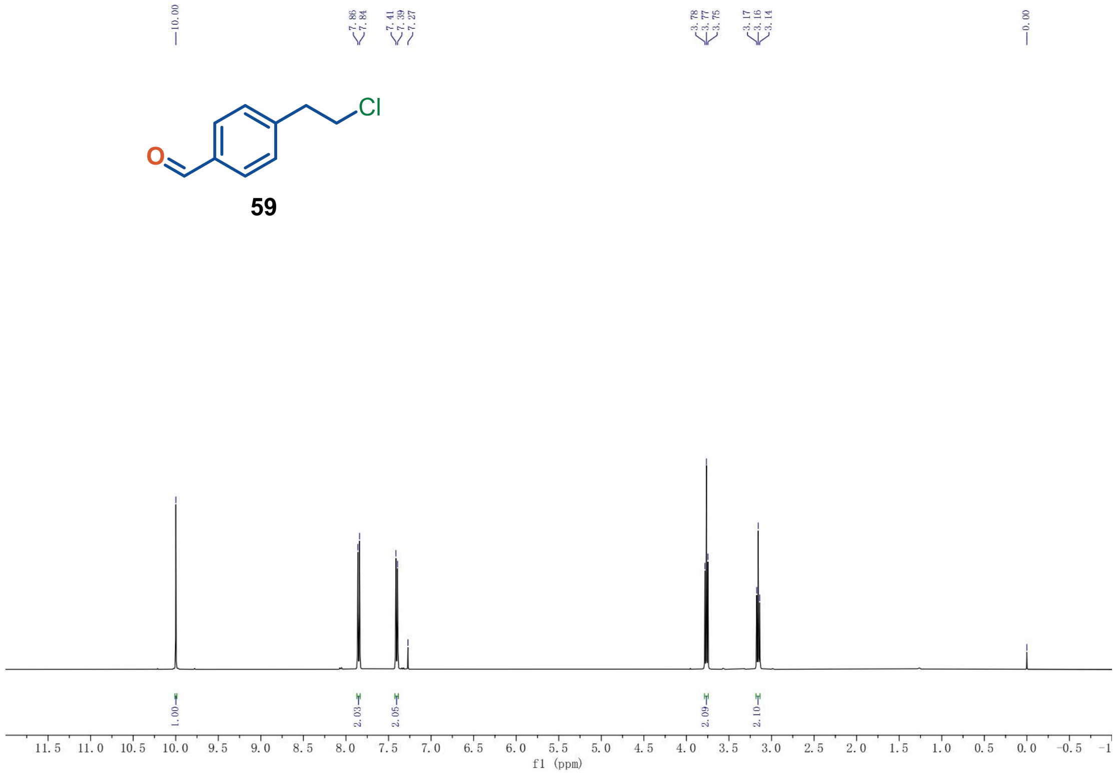

^{13}C NMR spectrum of **59**

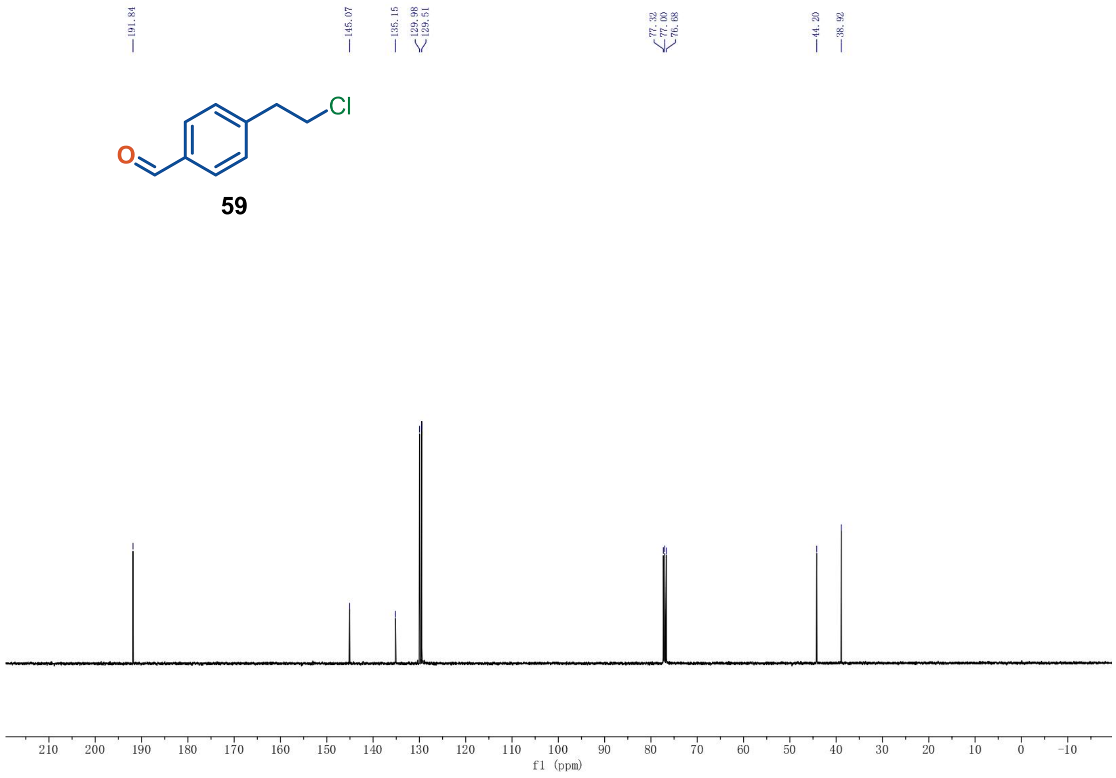

^{1}H NMR spectrum of **60**

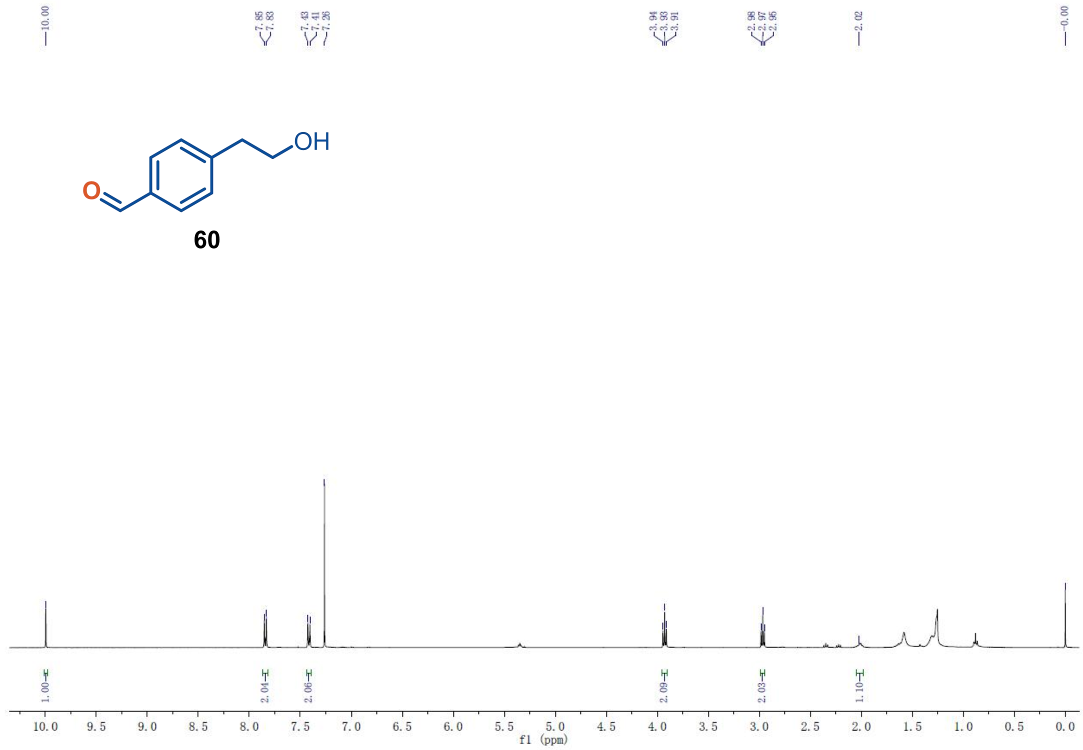

^{13}C NMR spectrum of **60**

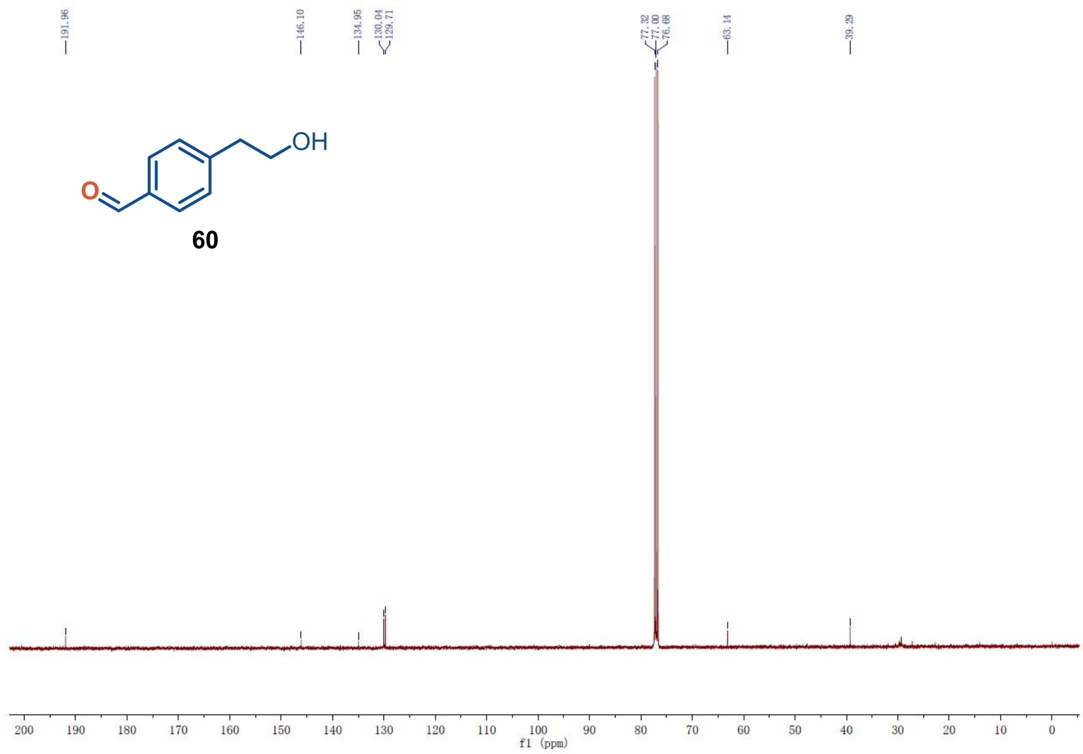

^{1}H NMR spectrum of **61**

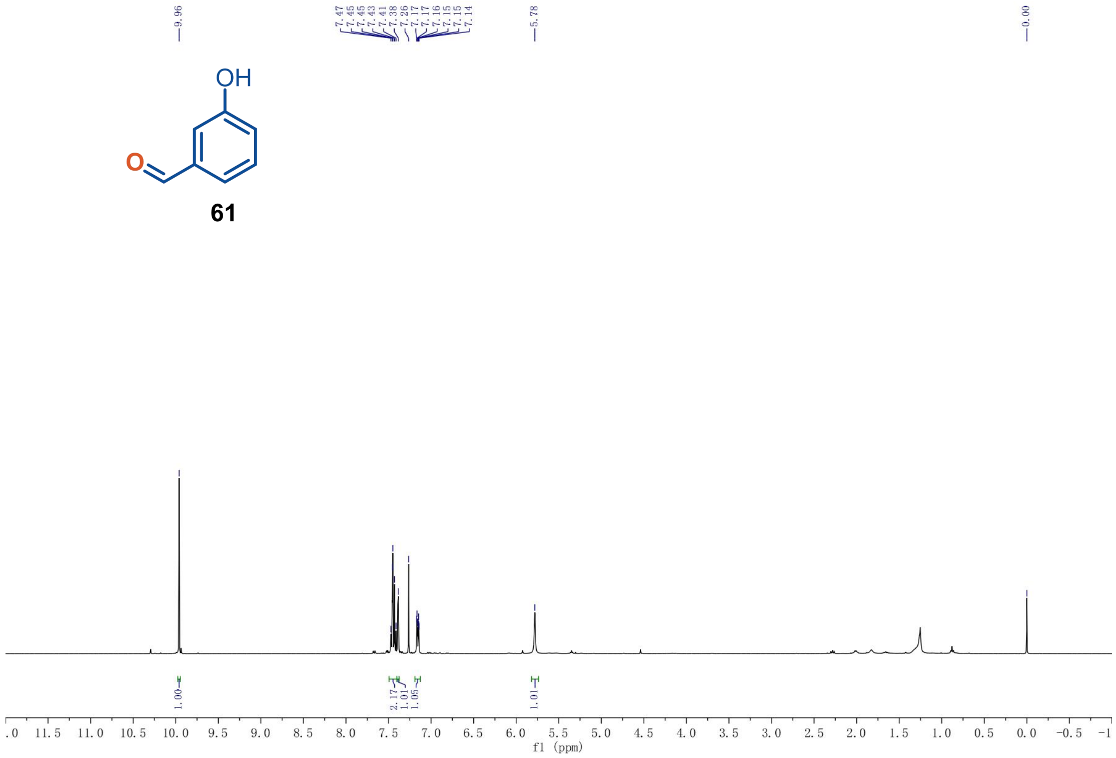

^{13}C NMR spectrum of **61**

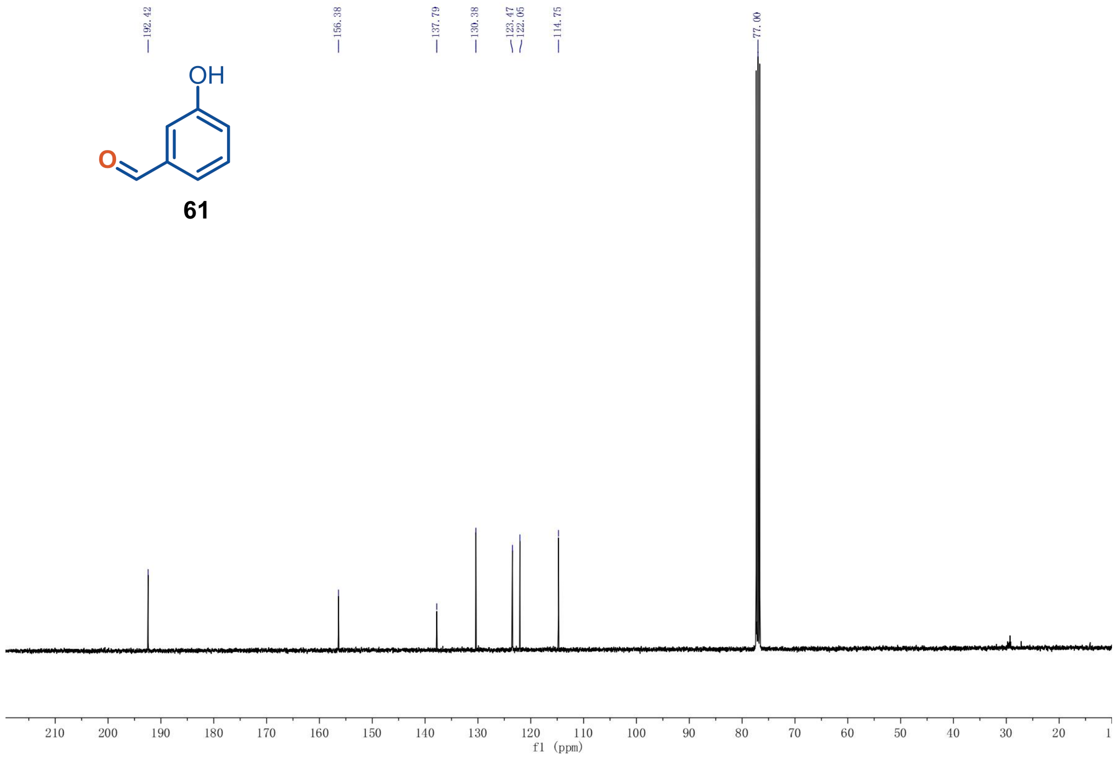

^{1}H NMR spectrum of **62**

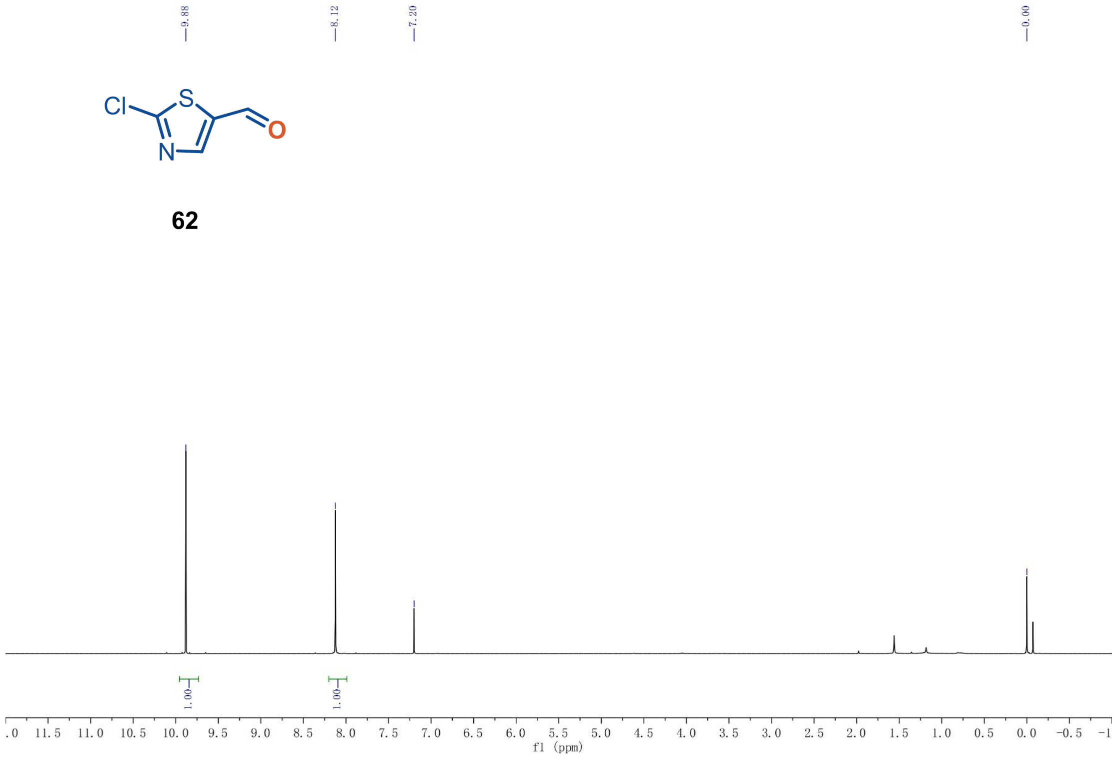

^{13}C NMR spectrum of **62**

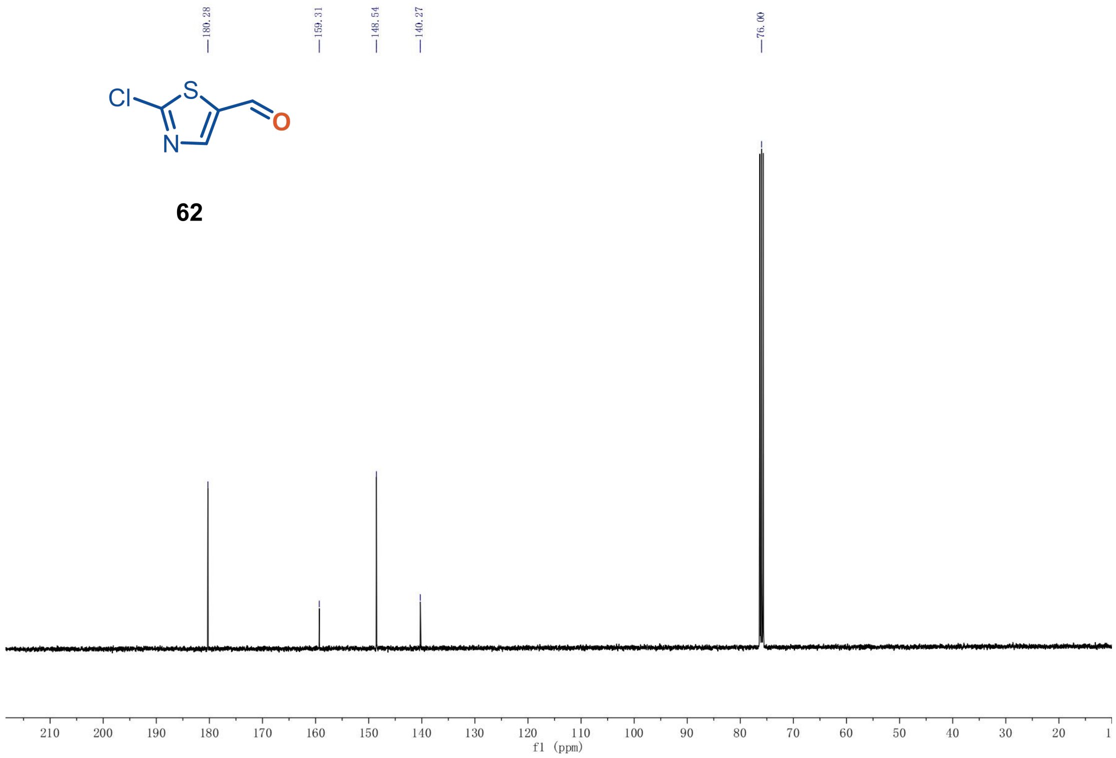

^{1}H NMR spectrum of **63**

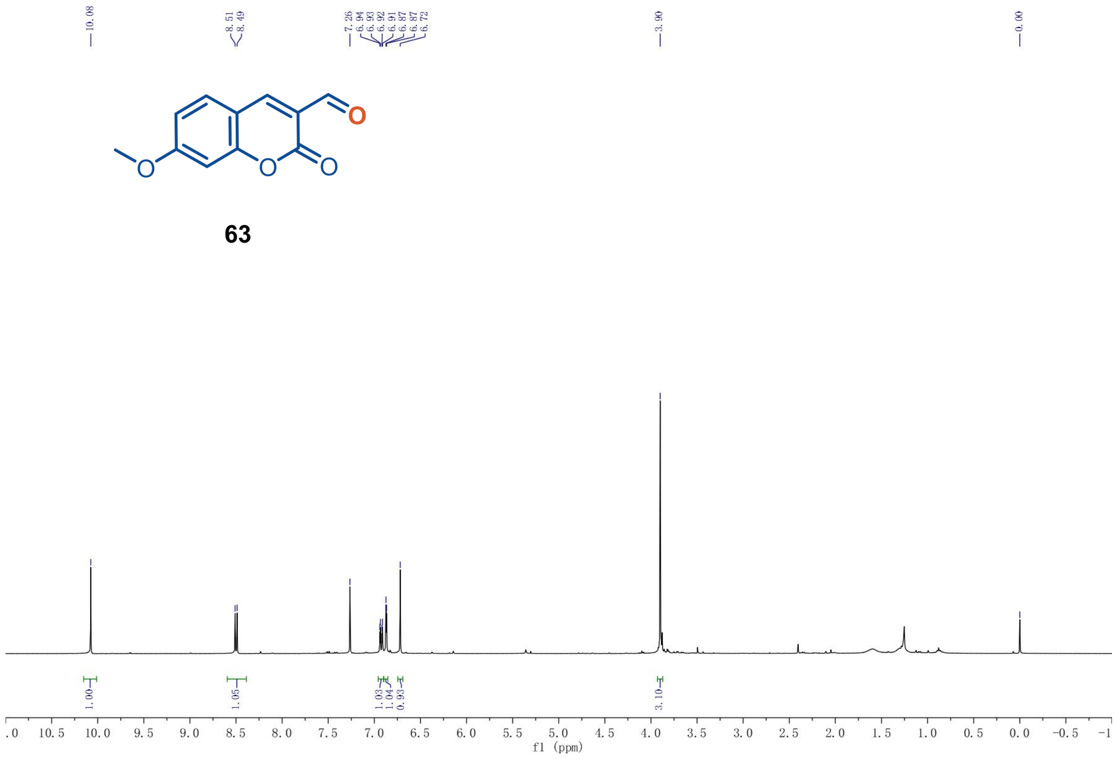

^{13}C NMR spectrum of **63**

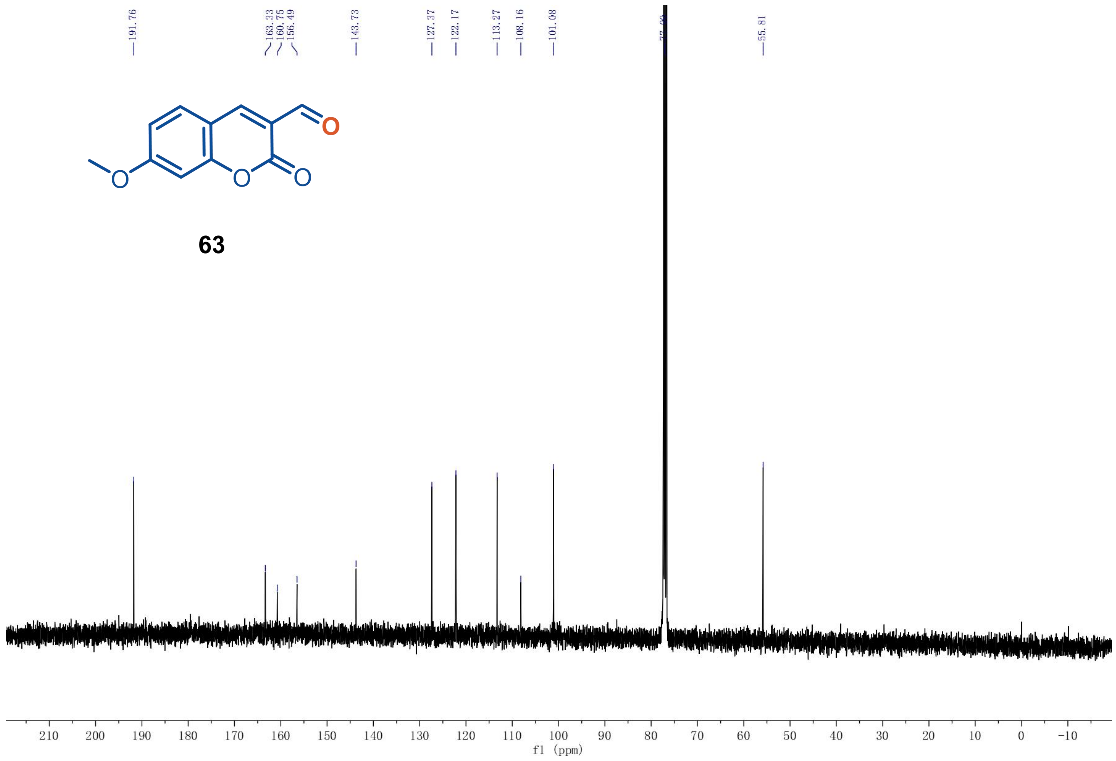

^{1}H NMR spectrum of **64**

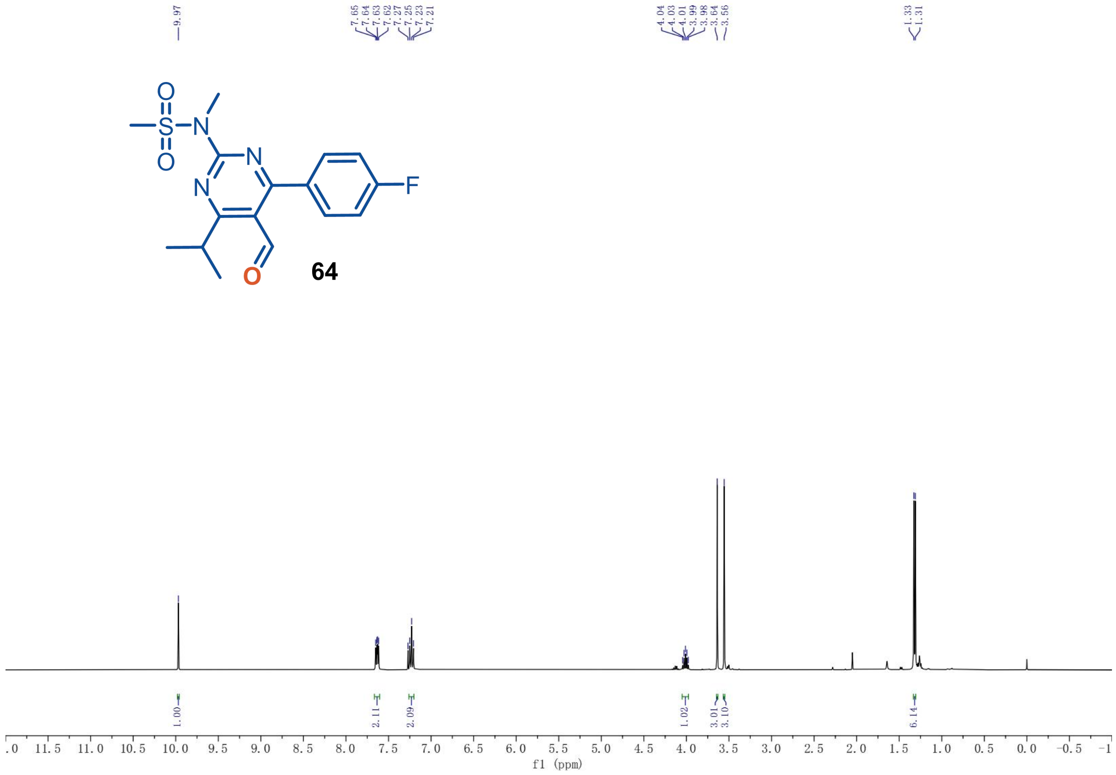

^{13}C NMR spectrum of **64**

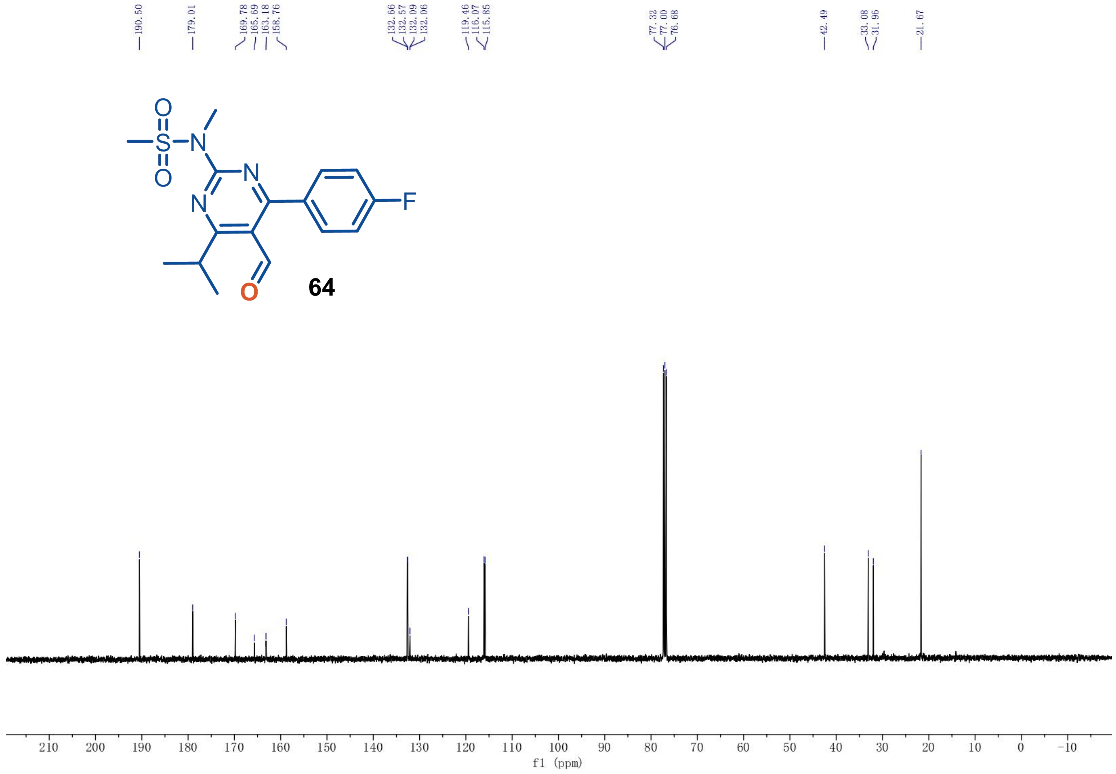

^{19}F NMR spectrum of 64

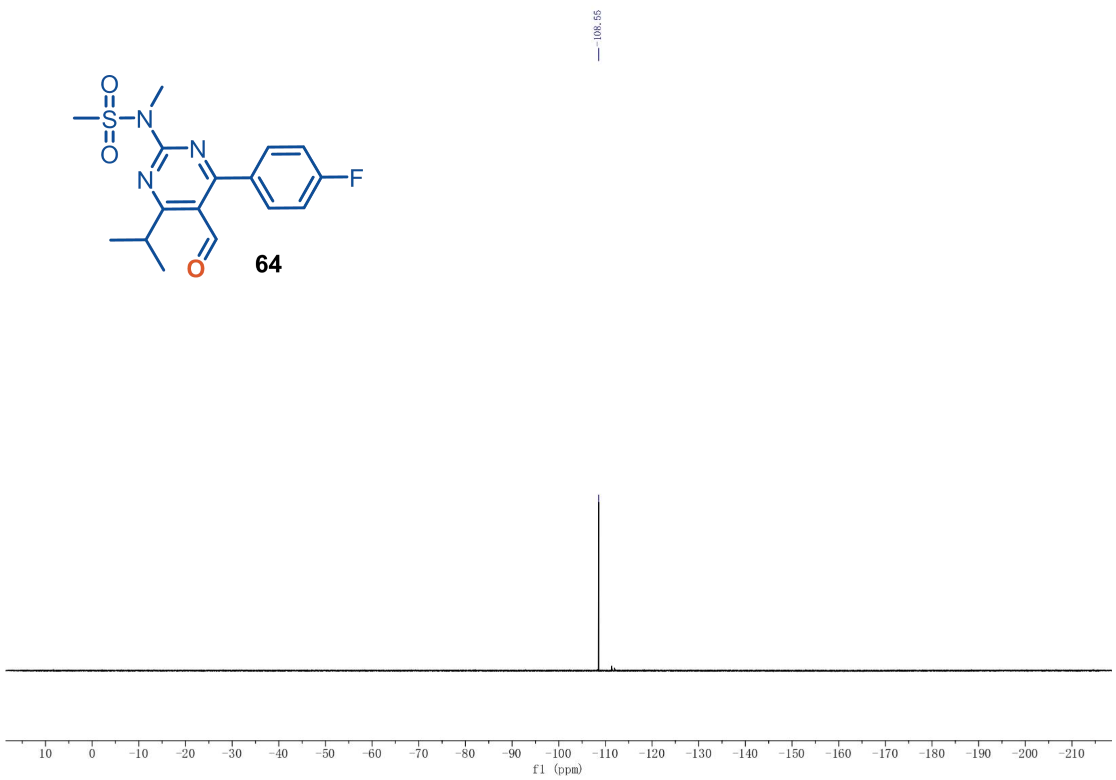

^{1}H NMR spectrum of **65**

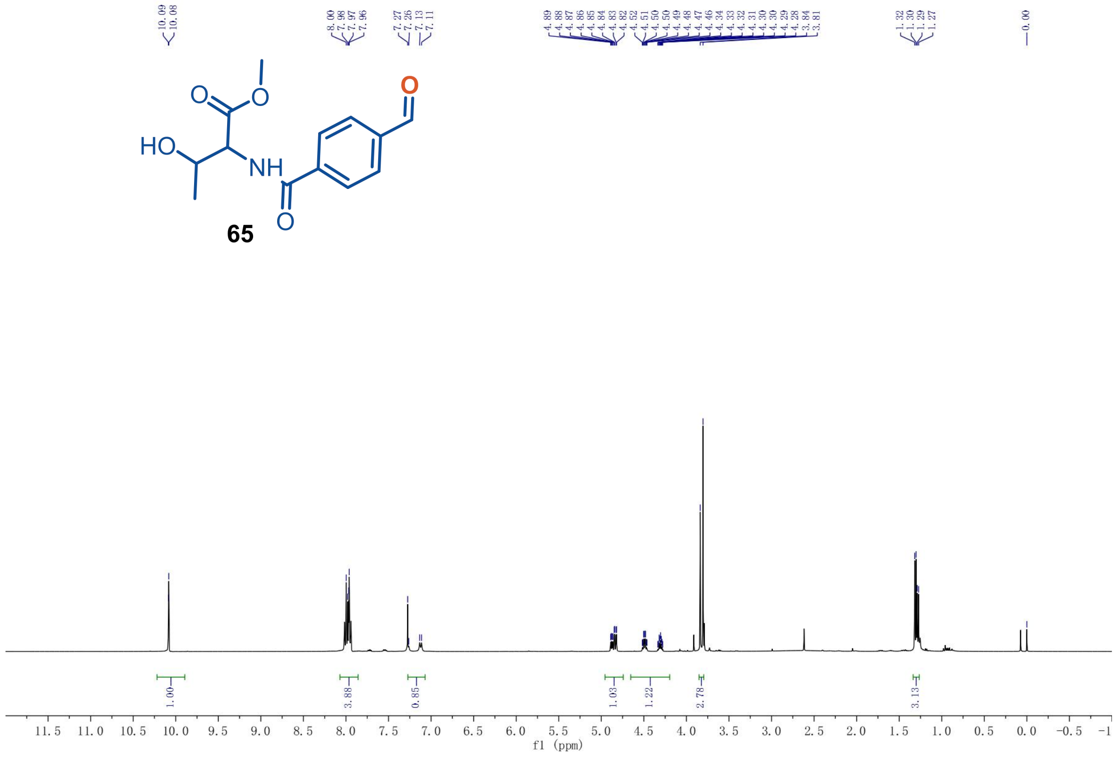

^{13}C NMR spectrum of **65**

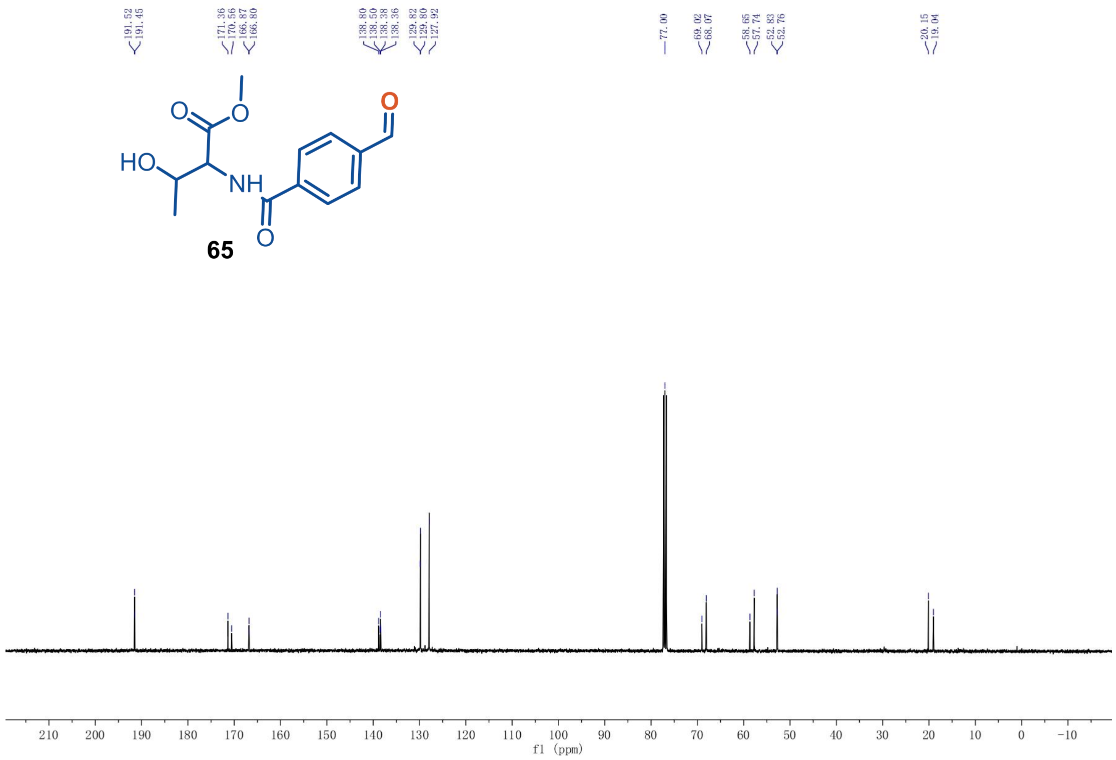

^{1}H NMR spectrum of **66**

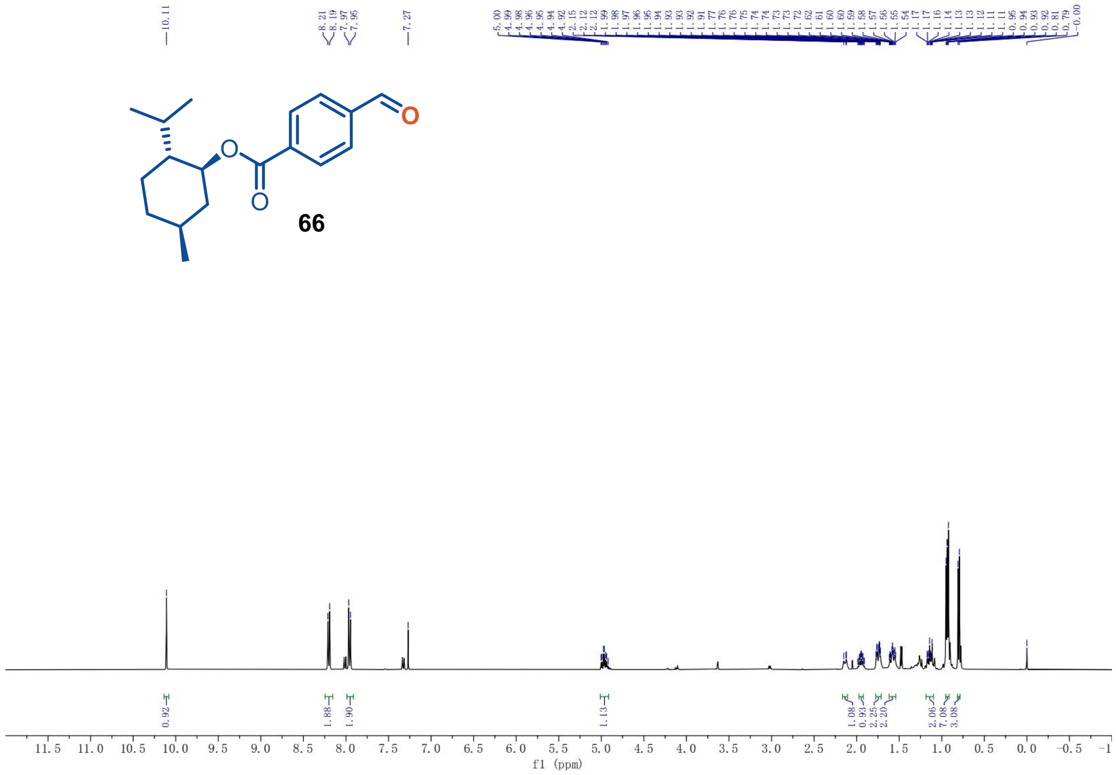

^{13}C NMR spectrum of **66**

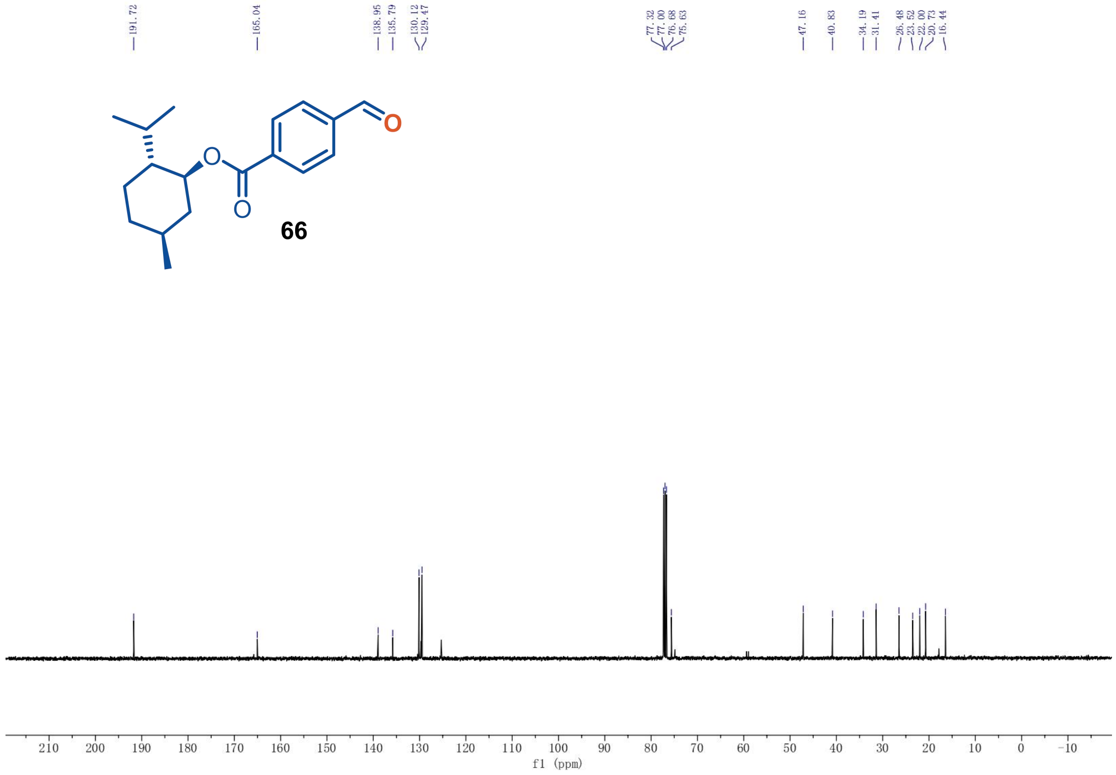

^{1}H NMR spectrum of **67**

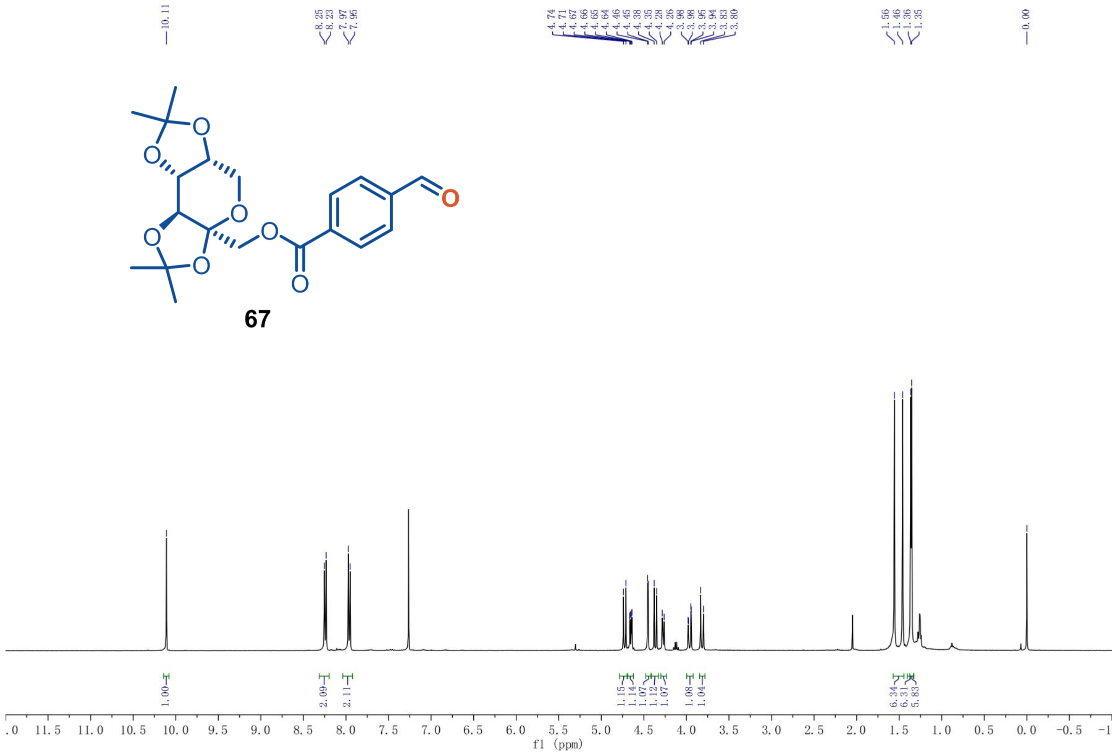

^{13}C NMR spectrum of **67**

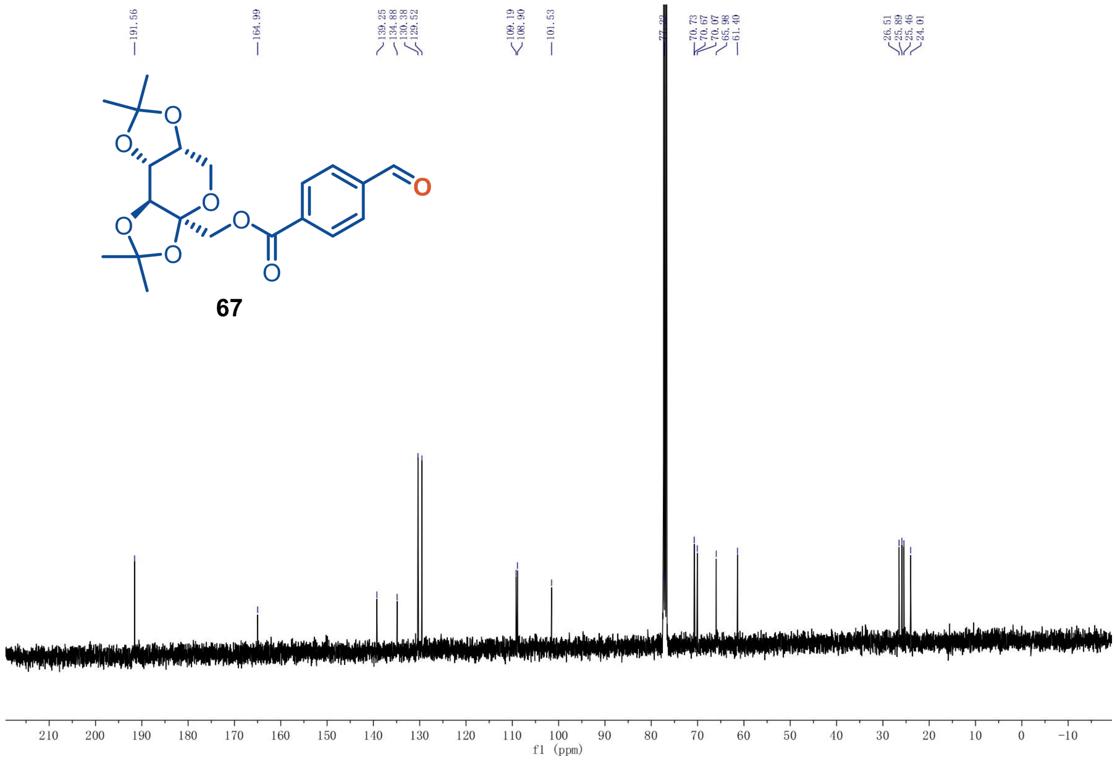

^{1}H NMR spectrum of **68**

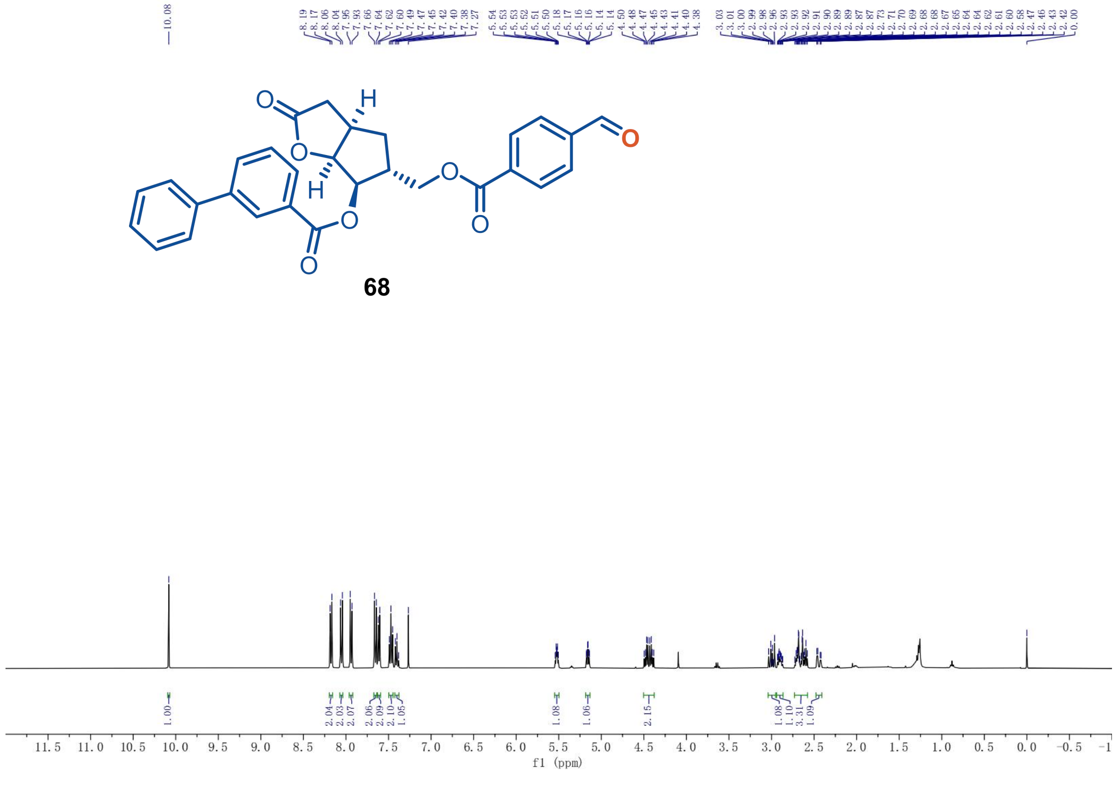

^{13}C NMR spectrum of **68**

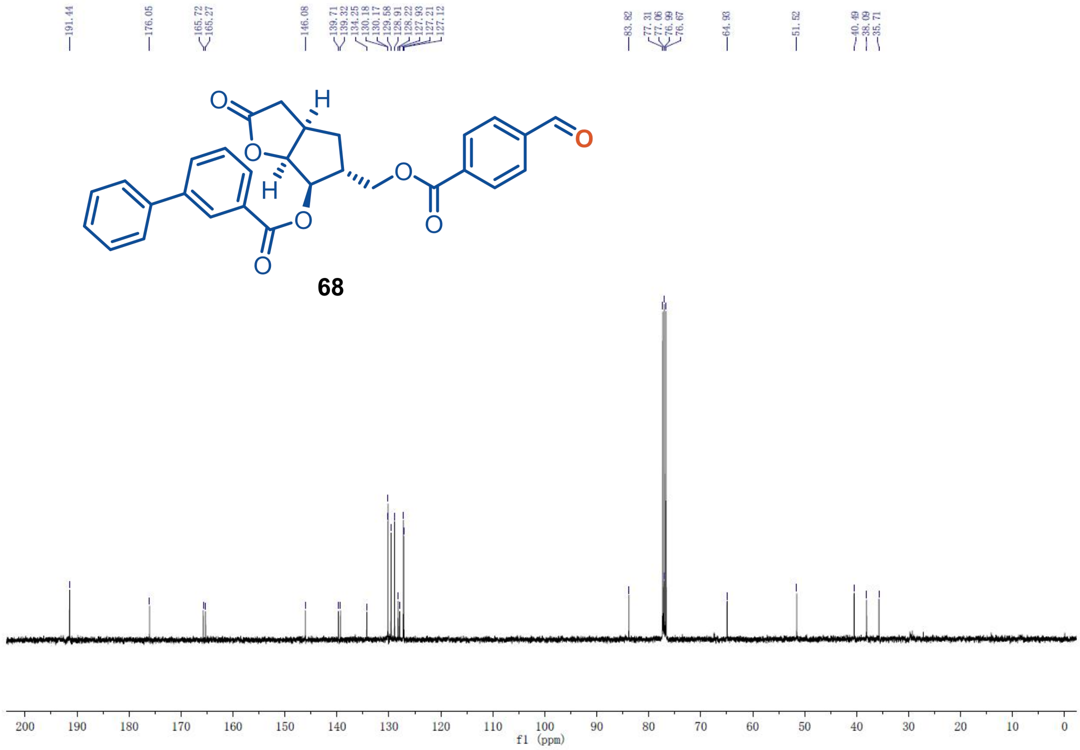

^{1}H NMR spectrum of **69**

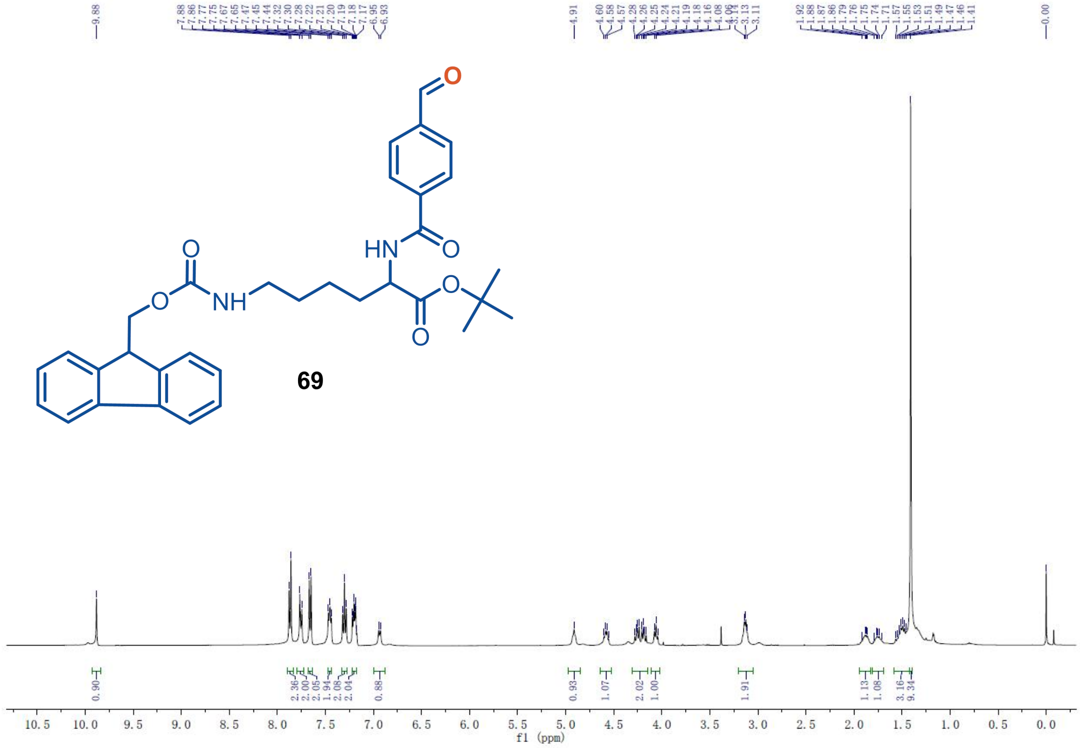

^{13}C NMR spectrum of **69**

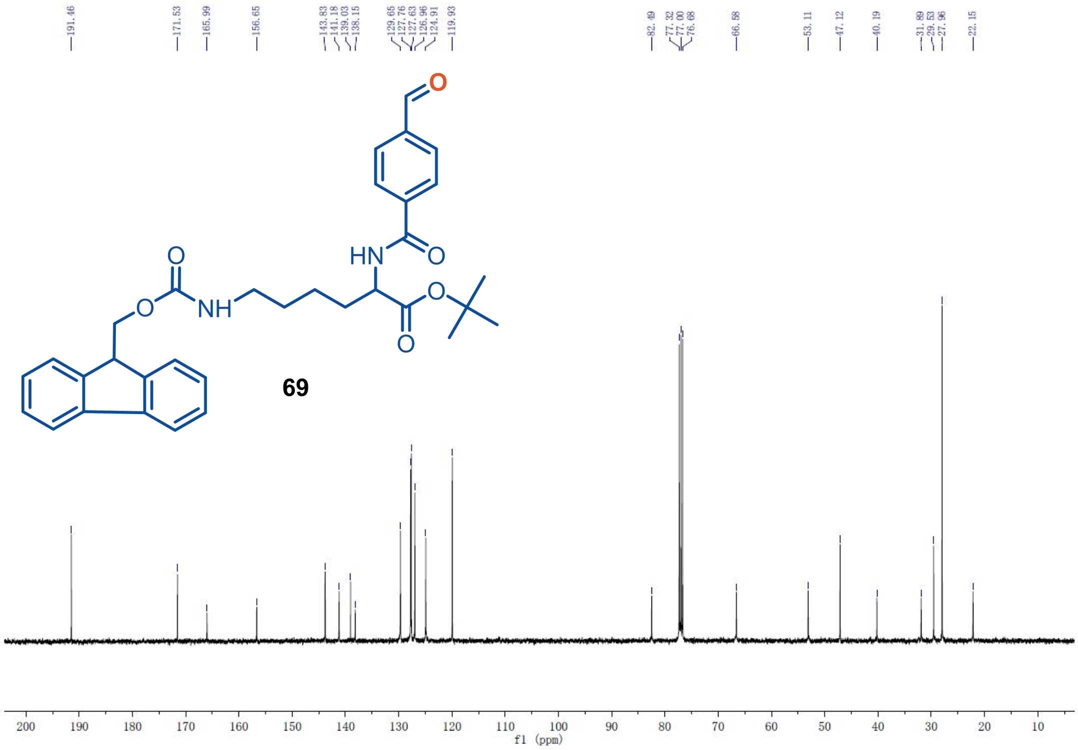

^{1}H NMR spectrum of **72**

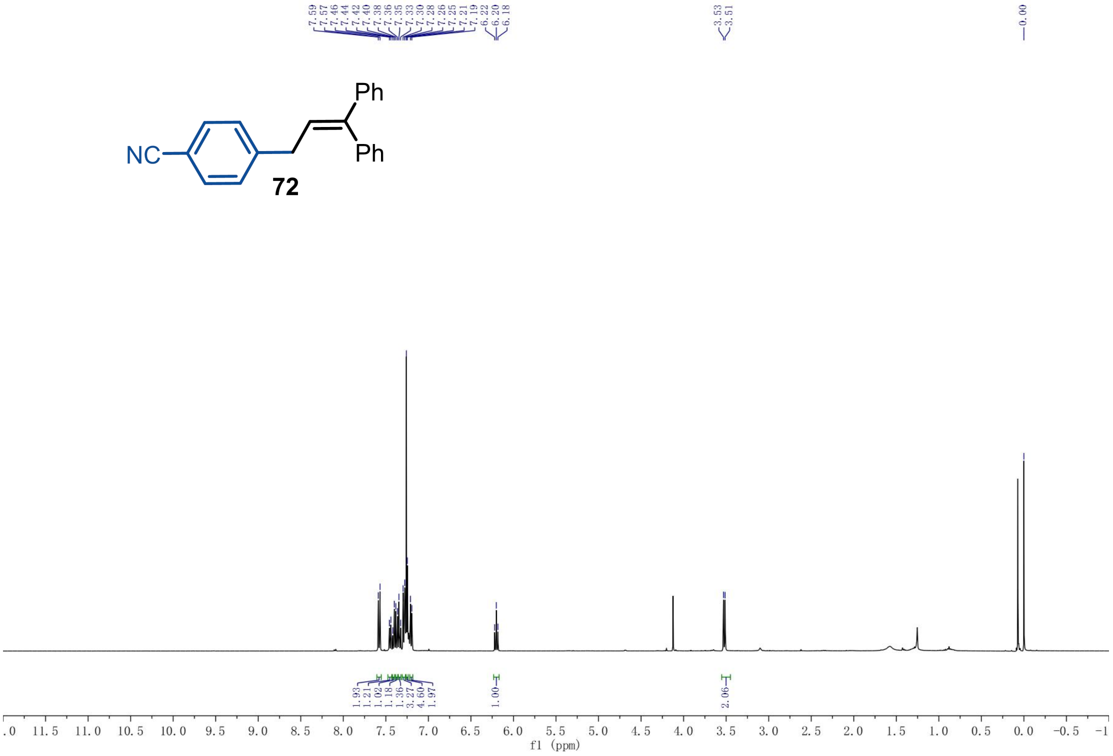

^{13}C NMR spectrum of **72**

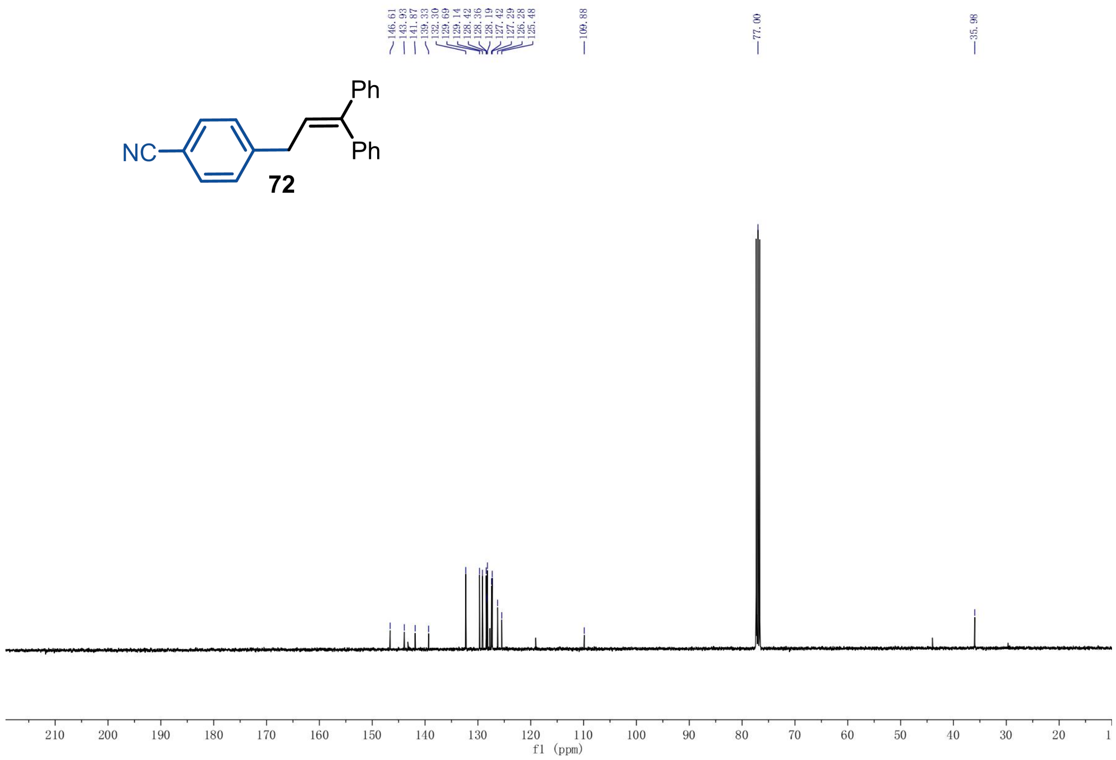

^{1}H NMR spectrum of **73**

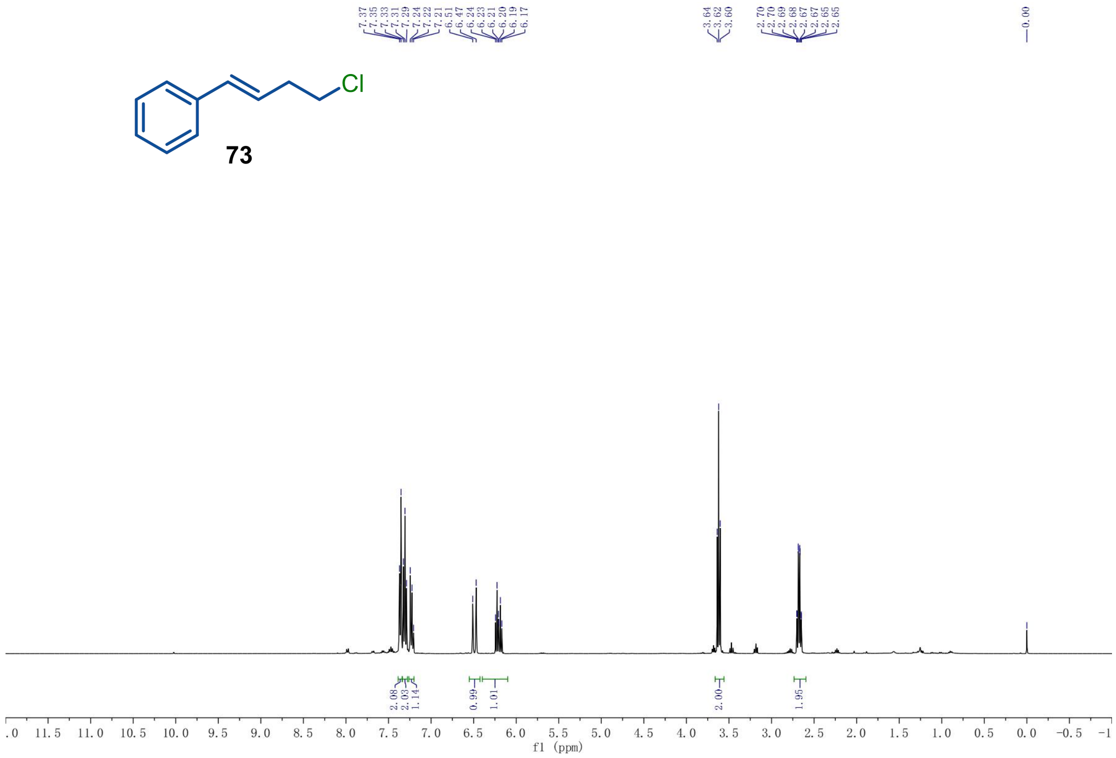

^{13}C NMR spectrum of **73**

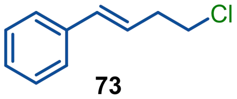

73

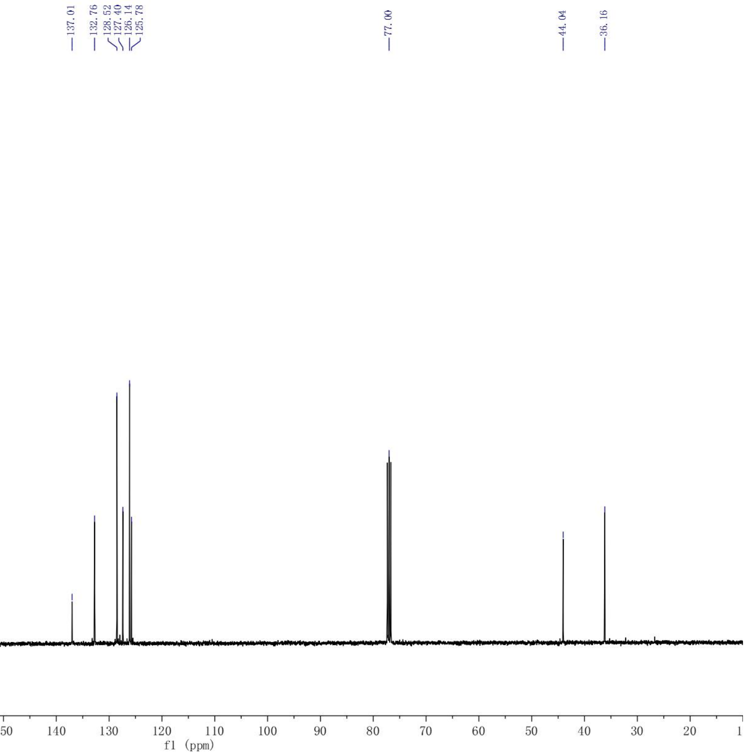

Supplement: SC-017-D5SC07057K-s001 [file SC-017-D5SC07057K-s001.pdf]
